# Ecdysteroid-Containing Squalenoylated Self-Assembling Nanoparticles Exert Tumor-Selective Sensitization to Reactive Oxygen Species (ROS)-Induced Oxidative Damage While Protecting Normal Cells: Implications for Selective Radiotherapy

**DOI:** 10.1021/acs.jmedchem.4c02758

**Published:** 2025-03-28

**Authors:** Máté Vágvölgyi, Endre Kocsis, Bizhar A. Tayeb, István Zupkó, Renáta Minorics, Ana Martins, Zsófia Hoyk, Gergő Ballai, Imre Szenti, Zoltán Kónya, Tamás Gáti, Dóra Bogdán, Gábor Tóth, Attila Hunyadi

**Affiliations:** † Institute of Pharmacognosy, University of Szeged, Eötvös str. 6, Szeged H-6720, Hungary; ‡ Institute of Pharmacodynamics and Biopharmacy, University of Szeged, Eötvös str. 6, Szeged H-6720, Hungary; § Institute of Biophysics, HUN-REN Biological Research Centre, Temesvári blvd. 62, Szeged H-6726, Hungary; ∥ Department of Applied and Environmental Chemistry, Interdisciplinary Excellence Centre, University of Szeged, Rerrich Béla sq. 1, Szeged H-6720, Hungary; ⊥ HUN-REN-SZTE Reaction Kinetics and Surface Chemistry Research Group, University of Szeged, Rerrich Béla sq. 1, Szeged H-6720, Hungary; # Servier Research Institute of Medicinal Chemistry (SRIMC), Záhony str. 7, Budapest H-1031, Hungary; ∇ Department of Organic Chemistry, Semmelweis University, Hőgyes Endre str. 7, Budapest H-1092, Hungary; ○ NMR Group, Department of Inorganic and Analytical Chemistry, Budapest University of Technology and Economics, Szt. Gellért sq. 4, Budapest H-1111, Hungary; ◆ HUN-REN-SZTE Biologically Active Natural Products Research Group, Eötvös str. 6, H-6720 Szeged, Hungary; ¶ Graduate Institute of Natural Products, Shih-Chuan first Rd. 100, Kaohsiung 807, Taiwan

## Abstract

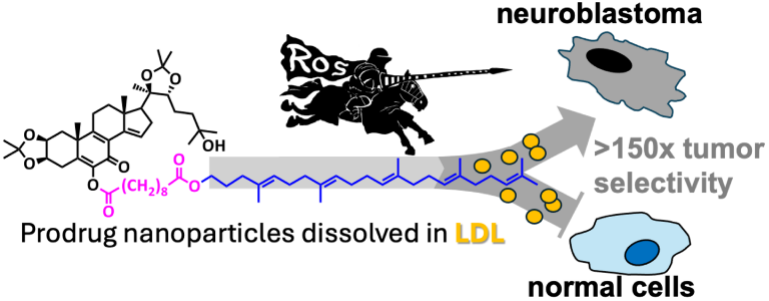

Central nervous system (CNS) tumors are exceptionally difficult to treat, and oxidative stress-inducing radiotherapy is an important treatment modality. In this study, we examined self-assembling pro-drug nanoconjugates of naturally derived antitumor ecdysteroids, which were designed to interfere with oxidative stress in brain tumor cells. Eight ecdysteroid-squalene conjugates were semi-synthesized and formulated into self-assembled aqueous nanosuspensions, which showed excellent stability for up to 16 weeks. The nanoassemblies demonstrated a strong dose-dependent sensitizing effect to *tert*-butyl hydroperoxide (tBHP)-induced oxidative damage in SH-SY5Y cells, while exerting a strong protective effect in MRC-5 fibroblast cells. In contrast, free ecdysteroids protected both cell lines from tBHP-induced damage. This suggests an important role for squalenoylation in the antitumor effect and indicates that our conjugates have potential as highly selective adjuvants in radiotherapy by sensitizing cancer cells and protecting surrounding tissues. Furthermore, our findings suggest a potential neuroprotective effect of nonconjugated ecdysteroids.

## Introduction

Cancer is among the diseases with the highest global mortality, while also sustaining an upsurging incidence.^1^ Although central nervous system (CNS) tumors constitute a moderate share of total neoplastic morbidity, they are of grave concern because of their occurrence in children and the elderly,^2^ and due to the poor prognosis associated with malignant variants.^3^ A multidisciplinary approach is generally needed for the treatment of CNS tumors. Depending on the tumor type, grade, histopathologic characteristics, patient preference, and posttreatment residue, a combination of surgery, chemotherapy, and radiotherapy may be administered for definitive treatment or local control, as advised by recent neuro-oncological guidelines.^4^ Ionizing radiation directly induces a proinflammatory response at both the cellular and tissue levels,^5^ which is further exacerbated by consequent reactive oxygen species (ROS) production.^6^ This mechanism is nonspecific to the tumor tissue and poses a high risk of parenchymal and vascular damage outside of the target, which can translate into posttreatment specific or systemic neurocognitive deficits.^7^ There are also challenges with respect to CNS tumor chemotherapy. These are primarily attributed to the limited penetration of anticancer agents through the blood–brain barrier (BBB) into the cerebrospinal fluid (CSF). This requires more invasive and/or high-dose therapeutic strategies,^8^ which further complicates treatment known to cause multiorgan toxicity.^9^

Targeted anticancer therapy research includes the identification of drugs that are effective against specific tumor targets,^10^ increasing tumor tissue-specific delivery of compounds using nanocarriers,^11,12^ or a combination of these two approaches.^13^ In addition to targeting tumors, nanoparticles can also improve the overall pharmacokinetic characteristics of a drug, resulting in reduced toxicity at higher doses.^14^ Although earlier methods of nanomedicine production involved the manufacture of polymeric or liposomal nanoparticles,^15^ a recently emerging approach is the preparation of self-assembling drug conjugates.^16^ This technique involves the covalent coupling of the drug to a self-assembly inducer molecule, usually a biocompatible polymeric chain, which forms a self-assembling drug conjugate. In an aqueous medium, the conjugate may form nanoparticles without the need for external emulsifiers; instead, they spontaneously self-assemble mediated by secondary molecular interactions.

Of the various manifestations of self-assembling drug conjugates, squalenoylation technology represents a novel approach to nanomedicine. Squalene is a precursor for cholesterol biosynthesis, which has the advantage of 1) avoiding synthetic challenges because of its natural occurrence, and 2) evading inherent contribution to toxicity due to its biodegradability and lack of immunogenicity. Squalenoylation was first introduced during the development of self-assembling nanostructures by Couvreur et al. through semisynthetic functionalization of the terminal double bond of squalene to enable its covalent conjugation with paclitaxel.^17^ This approach has garnered significant interest in the preparation of nanoassemblies, primarily for chemotherapeutic drug development.^18−23^ Squalenoylation improves the pharmacokinetics of anticancer agents and improves efficacy at equitoxic doses.^24^ Furthermore, squalenoylated nanoassemblies dissolve in plasma lipoproteins, primarily in the low-density lipoprotein (LDL) and very-low-density lipoprotein (VLDL) fractions, which offers a unique way of passively targeting LDL-receptor overexpressing tissues, such as solid tumors.^22^ Furthermore, LDL-receptor-mediated transcytosis, a mechanism that enables LDL to pass through the BBB, was successfully exploited for transporting cargo into the brain in vivo.^25^ This suggests that drug conjugates dissolved in LDL can bypass the BBB and target CNS tumors.

Ecdysteroids are a group of arthropod molting hormones that also naturally occur in several plant species. They are widely acknowledged for their beneficial bioactivities in mammals.^26,27^ We have previously identified less polar ecdysteroid derivatives with potent chemosensitizing effects on multidrug-resistant (MDR) and drug-sensitive cell lines. We have also described semisynthetic strategies, such as dioxolane formation on vicinal diols,^28−30^ and oxidative side-chain cleavage resulting in ecdysteroid derivatives with modified antitumor properties.^31^ We have also demonstrated good BBB penetration and marked sensitizing effect of semisynthetic ecdysteroids on SH-SY5Y neuroblastoma cells treated with vincristine.^32^ Recently, we prepared squalene-conjugated ecdysteroid prodrugs, which were formulated into stable nanoparticles that released 60–70% of their conjugates into horse serum lipoproteins within 24 h.^33^

Pharmacokinetics should be carefully considered in drug development at the lead discovery and optimization phase.^34−36^ Here, we exploit squalenoylation to prepare self-assembling ecdysteroid pro-drug nanoparticles as anticancer agents capable of CNS-specific delivery based on 1) extensive lipoprotein metabolism in the CNS^37,38^ and 2) the LDL-affinity of squalene-coupled compounds. Our approach is also mechanism-oriented and based on a recent discovery that the most abundant natural ecdysteroid 20-hydroxyecdysone modulates endothelial hyperresponsiveness to inflammatory stimuli.^39,40^ We hypothesize that ecdysteroids may be involved in this mechanism of proinflammatory, oxidative stress-inducing pathophysiology of radiotherapy-induced cellular toxicity. Potentiation of this mechanism has been demonstrated by several ROS-upregulating nanosystems.^41^ Therefore, we evaluated the effects of squalenoylated ecdysteroids on the oxidative stress tolerance of SH-SY5Y neuroblastoma cells.

## Results and Discussion

### Chemistry

**Scheme 1:**
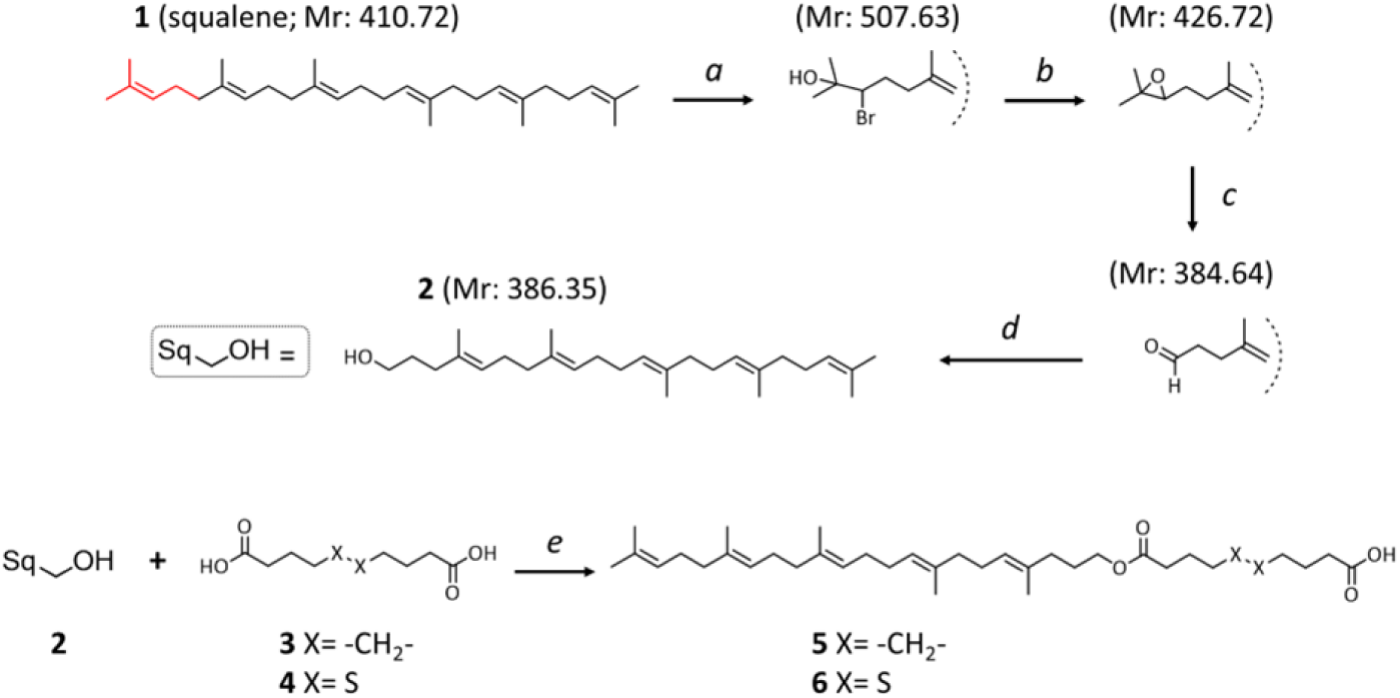
Preparation of Self-Assembly Inducer Squalene Derivatives Reaction conditions: *a*: *N*-bromosuccinimide (NBS) (1.5 equiv), H_2_O:THF – 1:5, RT, 30 min; *b*: K_2_CO_3_ (2 equiv), CH_3_OH, RT, 3 h; *c*: H_5_IO_6_ (1.8 equiv), 1,4-dioxane, RT, 3 h; *d*: NaBH_4_ (2 equiv), C_2_H_5_OH, RT, 24 h; *e*: sebacic acid or 4,4′-dithiodibutyric acid (2 equiv), DMAP (0.7 equiv), EDC·HCl (1.2 equiv), CH_2_Cl_2anh._, RT, Ar, 24 h

The terminal double bond of squalene (**1**) was functionalized based on a previously described multistep synthetic reaction sequence,^19,20^ which enabled the preparation of 1,1′,2-tris-norsqualenoyl alcohol (**2**) (see Scheme 1).

The terminal hydroxyl group of the resulting squalene derivative was conjugated (see Scheme 1 reaction *b*, detailed also below) with one of two linker^19^ compounds: sebacic acid (**3**) or 4,4′-dithiodibutyric acid (**4**), following a previously described esterification method^33^ that was performed with 4-dimethylaminopyridine (DMAP) and (3-(dimethylamino)propyl)-*N*′-ethylcarbodiimide hydrochloride (EDC·HCl) in anhydrous methylene chloride. The reaction yielded the corresponding squalene-coupled esters of sebacic acid and 4,4′-dithiodibutyric acid (**5** and **6** respectively), which were used as self-assembly inducers.

Two naturally occurring ecdysteroids were selected as the chemical starting point to this study. Calonysterone (**7**) was chosen because of its versatile pharmacological properties. It is a potent cytoprotective compound,^42^ whereas its less polar derivatives appear as promising antitumor derivatives. Likewise, ajugasterone C (**11**) was selected for the marked activity of its derivatives on drug-resistant cancer cells.^28,31^

The polarity of ecdysteroid-squalene conjugates can severely affect nanoparticle stability. Generally, less polar conjugates tend to form more stable nanoparticles. With this notion in mind, we designed a substrate sequence to prepare from compounds **7** and **11** so that we cover a range of lipophilicity for the substrates of subsequent conjugation reactions.

**Scheme 2:**
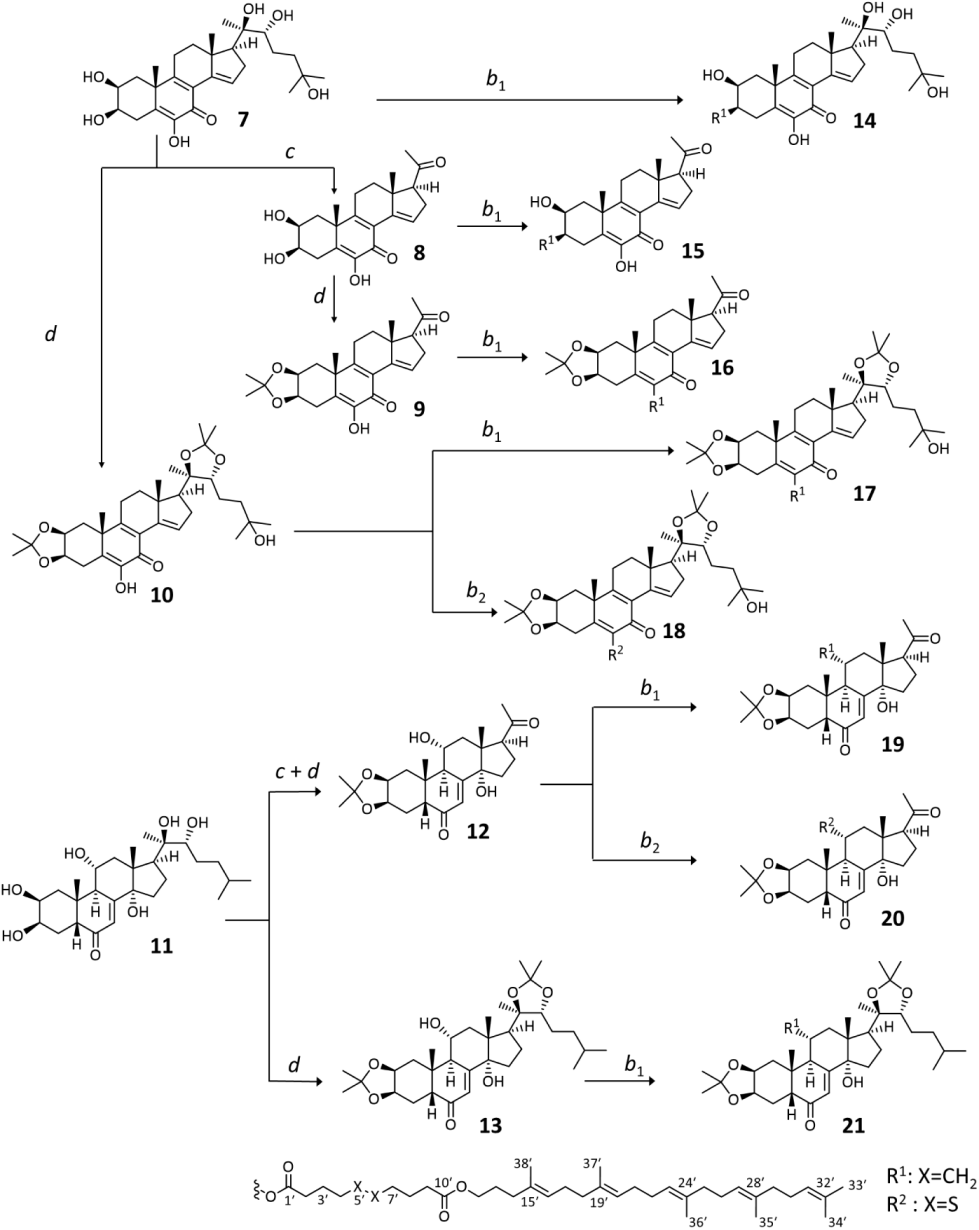
Preparation of Ecdysteroid Derivatives and Their self-Assembling Pro-drug Conjugates Reaction conditions: *b*: sebacic acid (*b*_1_) or 4,4′-dithiodibutyric acid (*b*_2_) (1.2 equiv), DMAP (2 equiv), EDC·HCl (2.5 equiv), CH_2_Cl_2anh_., RT, Ar, 2 h; *c*: PIDA (1 equiv), CH_3_OH, RT, 45 min; *d*: PMA, acetone, RT, 30 min. Atomic numbering of the R^1^ and R^2^ ester groups is shown to facilitate understanding of the NMR signal assignments

The compounds were subjected to oxidative side chain cleavage using hypervalent iodine reagent (diacetoxyiodo)benzene (PIDA) in methanol; we have previously found that this transformation may improve the antitumor properties of ecdysteroids.^31^ Acetonide formation on the vicinal diol(s) of the ecdysteroids was also performed; this moiety plays a key pharmacophore role in several antitumor ecdysteroids.^28^ This was achieved in acetone using phosphomolybdic acid (PMA), which yielded the 2,3-acetonide (**9**, **12**) or 2,3;20,22-diacetonide (**10**, **13**) derivatives depending on the starting materials. A combined strategy of these two transformations was used. First, the 2,3;20,22-diacetonide derivatives (**10** and **13**) of compounds **7** and **11** were synthesized. Then, the oxidative side-chain cleavage of calonysterone (**7**) was carried out to obtain compound **8** that was subjected to acetonide formation on its 2,3-diol to obtain compound **9**. A similar reaction sequence was performed with ajugasterone C (**11**), whose oxidative side-chain cleavage, followed by acetonide formation at the 2,3-diol produced compound **12** (Scheme 2).

### Preparation of Ecdysteroid Containing Self-Assembling Prodrug Conjugates

Conjugation of ecdysteroids (**7**, **8**, **9**, **10**, **12**, **13**) with self-assembly inducer side chains (**5** or **6**) was achieved following esterification.^33^ Each ecdysteroid was conjugated with the sebacic acid ester of 1,1′,2-tris-norsqualenoyl alcohol (**5**), which yielded compounds **14**–**17**, **19**, and **21**. The calonysterone diacetonide (**10**) and the side-chain-cleaved acetonide derivative of ajugasterone C (**12**) were also conjugated with the 4,4′-dithiodibutyric acid ester of 1,1′,2-tris-norsqualenoyl alcohol (**6**) to obtain compounds **18** and **20**, respectively (Scheme 2). The conjugates were purified by supercritical fluid chromatography (SFC); chromatographic conditions are provided in Table S72.

### Structure Elucidation

We recently reported the structure and complete ^1^H and ^13^C signal assignment of several 11α-squalenoylated ecdysteroids obtained from ajugasterone C 2,3;20,22-diacetonide (**21**) and the side chain-cleaved 11α-hydroxypoststerone 2,3-acetonide (**19**)^33^ as well as the characterization of squalenoylated C-20-oxime ecdysteroid 2,3-acetonides.^43^

Structure elucidation of the newly synthesized compounds was performed based on the exact molecular formulas obtained by HRMS and on detailed NMR studies. The location and identity of the newly formed functions and the NMR signals of the products were assigned by comprehensive one- and two-dimensional NMR methods using standard techniques.^44,45^ Most ^1^H assignments were made using general knowledge of the chemical shift dispersion along with the ^1^H–^1^H coupling pattern (^1^H NMR spectra). We previously used a similar NMR methodology for the ecdysteroid conjugates.^33,43^ The ^1^H, ^13^C, DEPTQ, APT, 1D sel-ROESY [τ_mix_: 300 ms], 1D sel-TOCSY, 1D sel-INEPT (^13^C), HSQC, edHSQC, HMBC and band-selective HSQC, and HMBC measurements were used to establish appropriate ^1^H and ^13^C signal assignments. Our previous NMR study on 11α-squalenoylated ecdysteroid compounds (**19**, **21**) revealed that esterification significantly increased the chemical shift of βH-11 by >1 ppm compared with the value measured in the starting steroid; however, it resulted in only a minimal change in the chemical shifts of the other signals in the spectrum. Therefore, we needed reliable and complete ^1^H and ^13^C signal assignments for all parent compounds (**7**–**13**). Among these, the previously not published ^1^H and ^13^C NMR data for compounds **9**, **10**, and **12** are listed in Table 1. The ^1^H and ^13^C chemical shifts of the steroid parts of the conjugated compounds **14**–**18,** and **20** are listed in Table 2, and the signals of the R-groups of the conjugated compounds **16**–**18**, and **20** are summarized in Table 3. The atomic numbering for the OR group is shown in Scheme 2. The appearance of five = CH signals for the R-groups in the ^1^H (δ 5.20–5.10 ppm) and ^13^C (δ 125.2–124.2 ppm) NMR spectra of compounds **14**–**18** and **20** justifies the connection of the long lipophilic side-chain R.

**Table 1:** ^1^H and ^13^C Chemical Shifts of Compounds **9** and **12** in CDCl_3_, and **10** in CD_3_OD,^a^500/125 MHz

	**9**^a^		**10**^a^		**12**^a^	
	H	C	H	C	H	C
1β α	2.67 1.58	38.6	2.66 1.57	39.8	1.20 2.56	40.0
2	4.26	73.5	4.27	77.0	4.53	72.6
3	4.21	75.6	4.14	75.3	4.30	71.5
4β α	2.36 3.39	27.7	2.33 3.32	28.6	2.15 1.78	27.1
5	-	129.3	-	130.8	2.35	52.1
6	-	143.0	-	144.9	-	202.9
7	-	179.4	-	181.2	5.85	122.7
8	-	123.7	-	125.2	-	159.6
9	-	163.4	-	164.9	2.88	41.9
10	-	40.5	-	41.7	-	38.7
11β α	∼2.7 ∼ 2.7	24.6	∼2.67 ∼ 2.67	25.6	4.15 -	67.9
12β α	2.32 1.73	35.4	2.28 1.51	37.6	2.17 2.32	41.0
13	-	46.8	-	47.9	-	47.2
14	-	138.5	-	142.6	-	84.1
15β α	6.91	127.2	6.89	127.8	2.07 1.65	32.1
16β α	3.05 2.47	32.8	2.75 2.33	33.6	2.30 1.98	21.2
17	2.88	62.8	1.90	56.5	3.30	58.2
18	0.84	17.2	1.04	18.0	0.62	17.8
19	1.51	25.3	1.49	26.1	1.05	23.6
20	-	208.8	-	84.9	-	209.0
21	2.23	31.3	1.26	22.0	2.17	31.3
22	-	108.9	3.81	83.4	-	108.2
Meβ–22	1.62	28.6	-	-	1.50	28.6
Meα–22	1.38	26.0	-	-	1.34	26.6
23			1.56–1.50	24.9		
24			1.73 1.50	42.2		
25			-	71.2		
26			1.19	29.0		
27			1.20	29.7		
28			-	109.1		
Meβ–28			1.57	29.0		
Meα–28			1.34	26.3		
29			-	106.9		
Meβ–29			1.32	27.3		
Meα–29			1.41	29.4		
HO-6	6.78	-				

a 500/125 MHz.

**Table 2:** ^1^H and ^13^C Chemical Shifts of the Steroid Moiety of Compounds **14**–**18** and **20** in CDCl_3_

	**14**^a^		**15**^a^		**16**^a^		**17**^c^		**18**^b^		**20**^b^	
	H	C	H	C	H	C	H	C	H	C	H	C
1β α	2.47 1.45	40.9	2.50 1.49	40.9	2.72 1.75	38.50	2.70 1.72	38.55	2.70 1.72	38.52	1.23 1.80	39.94
2	4.20	67.7	4.22	67.7	4.26	73.28	4.25	73.37	4.25	73.33	4.44	72.21
3	4.80	74.1	4.81	74.1	4.17	75.84	4.17	75.89	4.17	75.87	4.32	71.46
4β α	2.69 3.22	23.5	2.70 3.25	23.5	2.40 3.01	28.66	2.40 3.00	28.66	2.4 3.01	28.68	2.17 1.78	27.03
5	-	128.9	-	129.1	-	146.24	-	145.94	-	146.07	2.35	52.03
6	-	143.2	-	143.2	-	141.31	-	141.38	-	141.34	-	201.79
7	-	179.8	-	179.6	-	177.03	-	177.29	-	177.11	5.89	123.26
8	-	123.3	-	123.4	-	125.69	-	125.69	-	125.63	-	158.07
9	-	163.6	-	163.6	-	159.90	-	159.79	-	159.93	3.18	38.57
10	-	40.8	-	40.9	-	41.60	-	41.47	-	41.50	-	38.64
11β α	2.60 2.60	24.3	2.66 2.66	24.4	2.63 2.63	24.46	2.63 2.53	24.43	2.63 2.53	24.44	5.31 -	70.55
12β α	2.28 1.55	36.2	2.32 1.53	35.3	2.30 1.73	35.32	2.23 1.48	36.09	2.23 1.48	36.05	2.30 2.25	36.10
13	-	46.6	-	46.8	-	46.79	-	46.53	-	46.52	-	46.93
14	-	138.5	-	138.5	-	138.10	-	140.31	-	140.30	-	84.16
15β α	6.93	127.4	6.91	127.2	6.90	127.91	6.92	127.86	6.91	127.87	2.11 1.65	32.17
16β α	2.75 2.30	31.8	3.06 2.48	32.9	3.00 2.44	32.84	2.75 2.33	32.53	2.75 2.33	32.52	2.34 1.95	21.12
17	1.98	55.0	2.89	62.8	2.85	62.71	1.82	54.90	1.82	54.87	3.29	58.12
18	1.09	17.6	0.86	17.2	0.83	17.17	1.03	17.35	1.03	17.35	0.68	17.59
19	1.54	27.0	1.55	27.0	1.57	25.15	1.57	25.11	1.57	25.11	1.04	23.54
20	-	76.4	-	208.8	-	208.93	-	83.40	-	83.38	-	208.40
21	1.30	20.0	2.24	31.3	2.21	31.26	1.23	21.27	1.23	21.26	2.16	31.47
22	3.53	76.2	-	-	-	109.11	3.78	81.82	3.78	81.81	-	108.34
Meβ–22	-	-	-	-	1.60	28.57	-	-	-	-	1.48	28.54
Meα–22	-	-	-	-	1.37	25.96	-	-	-	-	1.35	26.55
23	1.59 1.41	25.9					1.63 1.47	23.71	1.63 1.47	23.69		
24	1.77 1.60	40.8					1.73 1.57	41.33	1.73 1.57	41.31		
25	-	70.8					-	70.33	-	70.33		
26	1.25	29.3					1.24	29.65	1.24	29.64		
27	1.26	30.0					1.25	29.24	1.25	29.14		
28	-						-	109.07	-	109.10		
Meβ–28	-						1.60	28.58	1.60	28.58		
Meα–28	-						1.37	25.97	1.37	25.97		
29	-						-	106.93	-	106.93		
Meβ–29	-						1.33	26.80	1.33	26.79		
Meα–29	-						1.44	28.91	1.44	28.90		

a 500/125.

b 800/200.

c 950/239 MHz.

**Table 3:** ^1^H and ^13^C Chemical Shifts of the R Group in Compounds **16**–**18** and **20** in CDCl_3_

	**16**^a^		**17**^c^		**18**^b^		**20**^b^	
	H	C	H	C	H	C	H	C
1′	-	171.65	-	171.66	-	170.88	-	171.96
2′	2.59	33.70	2.59	33.73	2.75	32.09	2.55 2.46	33.00
3′	1.75	24.83	1.75	24.85	2.17	24.26	2.08	23.96
4′	1.41	29.06	1.41	29.09	2.83	37.47	2.76	37.57
5′	1.36–1.32	29.07	1.36–1.32	29.09	-	-	-	-
6′		29.09		29.11	-	-	-	-
7′		29.11		29.13	2.75	37.73	2.74	37.77
8′	1.64	24.98	1.63	25.00	2.04	24.19	2.04	24.23
9′	2.30	34.35	2.30	34.37	2.45	32.64	2.45	32.64
10′	-	173.92	-	173.92	-	173.00	-	173.02
11′	-	-	-	-	-	-	-	-
12′	4.04	63.96	4.04	63.97	4.05	64.23	4.05	64.31
13′	1.73	26.87	1.73	26.91	1.73	26.85	1.73	26.84
14′	2.04	35.78	2.03	35.80	2.03	35.77	2.03	35.77
15′	-	133.69	-	133.71	-	133.63	-	133.59
16′	5.13	125.01	5.14	125.03	5.14	125.09	5.14	125.13
17′	2.08	26.63	2.08	26.66	2.08	26.66^d^	2.08	26.65
18′	1.99	39.65	1.99	39.67	1.99	39.72^d^	1.98	39.66
19′	-	135.09	-	134.99	-	134.98	-	135.12
20′	5.145	124.33	5.16	124.28	5.15	124.27	5.15	124.37
21′	2.03	28.24	2.02	28.26^d^	2.02	28.25^d^	2.02	28.25^d^
22′	2.03	28.24	2.02	28.27^d^	2.02	28.26^d^	2.02	28.26^d^
23′	5.15	124.26	5.15	124.36	5.15	124.35	5.15	124.25
24′	-	134.97	-	135.11	-	135.11	-	134.96
25′	1.99	39.73	2.00	39.75	1.99	39.66	1.98	39.74
26′	2.08	26.63	2.08	26.66	2.08	26.65^d^	2.08	26.66
27′	5.12	124.23	5.13	124.26	5.13	124.25	5.12	124.24
28′	-	134.88	-	134.90	-	134.90	-	134.91
29′	1.99	39.71	1.98	39.72	1.98	39. 74^d^	1.98	39.72
30′	2.08	26.74	2.07	26.76	2.07	26.75	2.07	26.75
31′	5.10	124.37	5.11	124.40	5.10	124.39	5.10	124.38
32′	-	131.24	-	131.24	-	131.25	-	131.25
33′	1.69	25.69	1.69	25.69	1.69	25.69	1.69	25.70
34′	1.61	17.67	1.61	17.68	1.61	17.68	1.61	17.68
35′	1.61	15.99	1.61	16.00	1.61	16.00	1.61	16.00
36′	1.61	16.03	1.61	16.03	1.61	16.04	1.61	16.05
37′	1.61	16.02	1.61	16.04	1.61	16.04	1.61	16.04
38′	1.61	15.86	1.61	15.87	1.61	15.87	1.61	15.87

a 500/125.

b 800/200.

c 950/239 MHz.

d tentative assignment.

The characteristic HRMS (Figures S1–S9) and NMR spectra (Figures S10–S71) of the compounds are presented as Supporting Information. To facilitate understanding of the ^1^H and ^13^C signal assignments, the stereostructures are also displayed in the spectra.

Compound **9**: HRMS data (Figure S1) revealed a molecular formula of C_24_H_30_O_5_. For structure and NMR signal assignments (Table 1), the following NMR spectra (Figures S10–S14) were used: ^1^H NMR; sel-ROE on δ 1.38, 0.84, and 1.51 ppm; ^13^C DEPTQ; edHSQC section; and HMBC.

Compound **10**: HRMS data (Figure S2) revealed a molecular formula of C_33_H_48_O_7_. The structure and NMR signal assignments (Table 1) were based on the following spectra (Figure S15–20): ^1^H NMR; sel-ROE on CH_3_-18; ^13^C DEPTQ; HSQC; edHSQC CH_2_ section; and HMBC.

Compound **12**: HRMS data (Figure S3) revealed a molecule formula of C_24_H_34_O_6_. The structure and NMR signal assignments (Table 1) were based on the following spectra (Figures S21–S24): ^1^H NMR; sel-ROE on Hα-2, CH_3_-19 and CH_3_-18; ^13^C DEPTQ; HSQC; edHSQC CH_2_ section; and HMBC.

Compound **14**: HRMS data (Figure S4) revealed a molecular formula of C_64_H_100_O_10_. The following NMR spectra (Figures S25–31) were used for its structure elucidation: ^1^H NMR; ^13^C APT; edHSQC CH and CH_3_ sections; edHSQC CH_2_ section; ROESY Me-section, and HMBC Me-section. The ^1^H and ^13^C chemical shifts of the steroid moiety are listed in Table 2. Characteristic δ ^1^H/^13^C values of the R group: δ HC′= 5.15–5,11 m (5H)/125.1–124.2; H_3_C-33′1.69/25.69, H_3_C-34′1.61/17.68, H_3_C-35′-38′1.62(12H)/16.05, 16.05, 16.01, 15.88; C-1′172.39, C-10′173.91, H_2_C-12′4.04/64.02 ppm.

Compound **15**: HRMS data (Figure S5) revealed a molecular formula of C_58_H_86_O_8_. ^1^H NMR; ^13^C APT; edHSQC; HMBC; and ROESY spectra were detected (Figures S32–36). The ^1^H and ^13^C chemical shifts of the steroid moiety are listed in Table 2. Characteristic δ ^1^H/^13^C values of the R group: δ HC′= 5.17–5,11m (5H)/125.1–124.2; H_3_C-33′1.70/25.69, H_3_C-34′1.61/17.68, H_3_C-35′-38′1.61(12H)/16.05, 16.04, 16.00, 15.88; C-1′172.38, C-10′173.90, H_2_C-12′4.06/64.02 ppm. The Hα-3/C-1′HMBC cross-peak (4.81/172.38 ppm in Figure S35) confirmed the selective esterification in the C-3 position.

Compound **16**: HRMS data (Figure S6) revealed the C_61_H_90_O_8_ molecular formula. ^1^H NMR; ^1^H NMR section; sel-TOCSY on (δ 4.04/6.90/4.26) t_mix_ = 120 ms; ^13^C DEPTQ; ^13^C DEPTQ section; HSQC; HSQC section; edHSQC CH_2_ section; HMBC; HMBC section; sel-INEPT (δ 4.04*t*/2.30*t*/2.59t); sel-INEPT (δ 4.04*t*/2.30*t*/2.59t) section (Figures S37–48) were the spectra used for the NMR assignment. ^1^H and ^13^C chemical shifts of the steroid moiety are listed in Table 2, whereas the signals of the R group are listed in Table 3. The disappearance of the characteristic δ HO-6 (6.78s ppm) signal as compared to the parent compound **9** confirms the formation of the 6-conjugated structure in **16**. Although the 1D sel-TOCSY (Figure S39) measurements allow separate observation of the ^1^H signals within the hydrogen spin system, the 1D sel-INEPT (Figures S47, S48) with extreme selectivity identified the ^13^C nuclei that are connected to this proton through heteronuclear *J*(^1^H,^13^C) spin–spin coupling.^46^

Compound **17**: HRMS data (Figure S7) revealed the C_70_H_108_O_10_ molecular formula. Taking advantage of the extreme sensitivity and spectral dispersion provided by the high field strength (950/239 MHz) as well as the advantages of selective and band selective methods, the applied measurements (Figures S49–56: ^1^H NMR; sel-TOCSY on 15, 12′and 22; ^13^C NMR; edHSQC and = CH section; edHSQC CH_2_ section; band-sel. HSQC sections (33–43 and 32–22 ppm); edHSQC and HMBC CH_3_ sections; HMBC) resulted in complete ^1^H and ^13^C assignments (Tables 2 and 3, respectively).

Compound **18**: HRMS data (Figure S8) revealed the C_68_H_104_O_10_S_2_ molecular formula. The NMR measurements (Figures S57–S62) ^1^H NMR; ^1^H NMR section 3.1–0.9 ppm; sel-TOCSY on 4.05, 1.69, and 2.83 ppm; ^13^C DEPTQ; edHSQC sections and band selective HSQC; HMBC and band selective HMBC assignment of C-4′and C-7′, resulted in chemical shifts very similar to those measured for compound **17**, except for the H_2_C-4′and H_2_C-7′methylenes in the immediate vicinity of the −S-S- atoms.

Compound **20**: HRMS data (Figure S9) revealed a molecular formula of C_59_H_90_O_9_S_2_. The NMR measurements (Figures S63–71) ^1^H NMR; sel-TOCSY on (δ 4.05/1.69/2.55) t_mix_ = 120 ms; ^13^C NMR; ^13^C DEPTQ; edHSQC and band selective HSQC = CH section; band selective HSQC section 3.4–0.6/40.5–15 ppm; HMBC; band selective HMBC sections 27.2–25.6 and 17.8–15.6 ppm; and band selective HMBC sections in the 41.6–36.2 and 136–121 ppm range resulted in chemical shifts very similar to those that we published for compound **19**,^33^ except for the H_2_C-4′and H_2_C-7′methylenes in the immediate vicinity of the −S-S- atoms.

### Preparation and Characterization of Nanoparticles

Following our preparative semisynthetic work, the resulting compounds **14**–**21** were subjected to nanoprecipitation, following a published procedure.^33^ This method enabled the self-assembly of the ecdysteroid conjugates in water. The resulting colloid suspensions were periodically characterized by dynamic light scattering (DLS), as it is a theoretically well understood, widely accessible and commonly used method for analyzing hydrodynamic characteristics of nanoparticles.^47^ The acquired data is closely related to particle morphology (particle size—average hydrodynamic diameter (Z-Average); dispersity – polydispersity index (PdI)), although the current research is more interested in expected long-term colloidal stability (zeta potential), and the actual long-term colloidal stability through repeated measurements. Nanosuspensions were studied for 10 or 16 weeks. Even though evaluation of the results indicated some statistically significant changes in the average hydrodynamic size after 10 weeks for compounds **18**–**21**, size increase (3.8%), indicating aggregation processes and therefore some degree of instability, was found only for compound **19** (Table S73). Plots of raw light scattering data are available as Figures S74–S95.

In general, we can conclude that 1) the average hydrodynamic diameter of the nanoparticles ranged from 135.4 to 268.5 nm with no major shifts in the size of the individual nanostructures over time; 2) the polydispersity index did not exceed 0.27, which suggests samples with considerably monodisperse particle arrangement; and 3) zeta potential values were comfortably above, or close to (compounds **20** and **21** at 10 weeks) the generally regarded lower limit of preferable potential colloidal stability (±30 mV).

### Biology

#### Experimental Design to Test the Effect of the Compounds on Oxidative Stress

The MTT assay provides insight into the impact of varying concentrations of tBHP on cell viability. Following exposure to tBHP concentrations ranging from 1.95 μM to 1,000 μM for 4 h, cell viability was reduced in a dose-dependent manner, with an IC_50_ value of 50.6 ± 2.5 μM (Figure S98). Because tBHP is widely used as a ROS inducer,^48^ tBHP-treated SH-SY5Y cells are useful for examining oxidative stress-induced cell damage.^49,50^

To determine the concentrations of each compound for the assay, a pilot study was performed. This involved a smaller subset of compounds that served as representative examples of typical chemical structures, i.e., a nonfunctionalized ecdysteroid (**7**), its side chain-cleaved derivative (**8**) and the 2,3-acetonide of the latter (**9**), together with their nanoassemblies **14**, **15**, and **16**. These compounds were tested in a range of 0.5–10 μM for their effect on tBHP-induced cellular damage. The results indicated that 0.5 μM of each compound exhibited enhanced activity in the cells (Figure S99). Therefore, this concentration was selected to test the remaining compounds in this assay. This experimental design was supported by our preliminary experiments showing that ecdysteroids did not show significant effects on cell viability at this dose when administered alone compared to the effect of vincristine applied as a positive control (Figures S96 and S97). Although 0.5 μM of some nanoassemblies (e.g., **14** and **16**) slightly decreased SH-SY5Y (but not MRC-5) cell viability, the effect was less than 40% (Figure S96). Therefore, a more complex evaluation of synergy (e.g., using combination indices or isobolograms) was considered irrelevant.

#### Effect of Free Ecdysteroids and Their Self-Assembled Nanoparticles on tBHP-Mediated Cellular Damage

**Figure 1:**
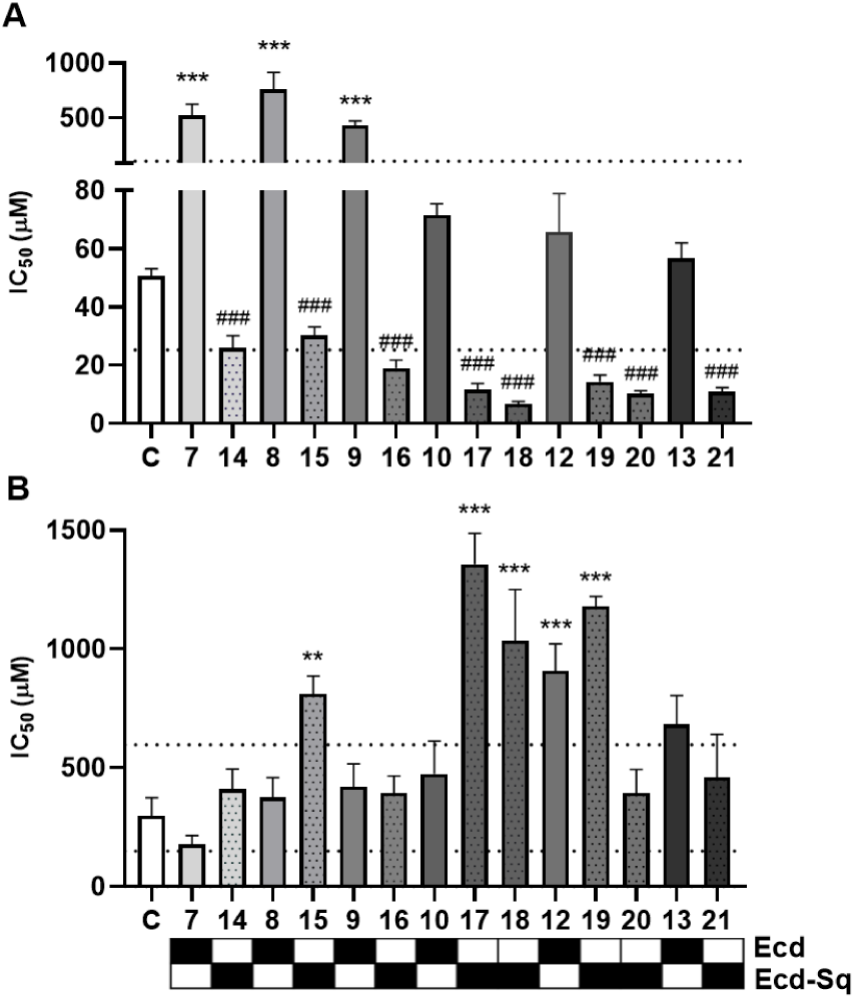
IC_50_ values of tBHP in the absence (C) or presence of ecdysteroids (Ecd; **7**–**10**, **12**, **13**) or their self-assembled nanodrugs (Ecd-Sq; **14**–**21**) in SH-SY5Y neuroblastoma cells (**A**) or MRC-5 fibroblasts (**B**). Cells were pretreated with 0.5 μM of the test compounds for 48 h before exposure to tBHP for 4 h at various doses (1.95–1,000 μM). Error bars show standard error of the mean (SEM). Statistical significance was evaluated by one-way ANOVA and Dunnett’s posthoc test separately for the free and squalenoylated ecdysteroids; **, and *** represent significant protection from tBHP cytotoxicity at *p* < 0.01, and *p* < 0.001, respectively; ###: significant sensitization to tBHP at *p* < 0.001 (*n* = 6–12). Horizontal dashed lines represent threshold values for twice and half as much as of the IC_50_ value of tBHP alone. Each ecdysteroid and its nanoformulated pro-drug(s) are shown next to each other to facilitate comparison.

Squalenoylated nanoparticles get dissolved in serum lipoproteins and enter cells through endocytosis. Furthermore, we have previously demonstrated that this may be exploited in vitro provided that enough time is available to preincubate the cells in culture medium.^33^ Therefore, the cells were pretreated with the ecdysteroids for 48 h prior to 4 h of tBHP treatment, and the IC_50_ values are shown in Figure 1. Numerical data are provided as in Table S100.

When evaluating the results, we performed statistical analysis but also defined a 2-fold difference compared with tBHP treatment alone as a relevant cutoff. A clear difference was observed in the behavior of free and nanoformulated ecdysteroids on neuroblastoma cells. Compounds **7**, **8**, and **9** (i.e., free calonysterone derivatives) increased oxidative stress resistance of the SH-SY5Y cell line by 10–15-fold. This is consistent with the previous results observed for 20-hydroxyecdysone that effectively protected SH-SY5Y cells from reactive oxygen species (ROS)-mediated apoptosis caused by the neurotoxin 6-hydroxydopamine (6-OHDA).^51^ A similar tendency was observed for ajugasterone C derivatives **12** and **13**. In terms of structure–activity relationships (SAR), it can be stated that the more unsaturated calonysterone derivatives (**7**, **8**, **9**) showed more potent cytoprotective effects than the classical 7-ene-6-one B-ring ajugasterone C derivatives (**12**, **13**). A notable exception to this trend was calonysterone 2,3,20,22-diacetonide (**10**), which showed activity of similar order of magnitude to that of ajugasterone C derivatives. In contrast to the free ecdysteroids, nearly all nanoconjugates exhibited strong sensitization of neuroblastoma cells to the cytotoxic effects of tBHP, with the sole exception of compound **15**, which was below our predefined cutoff. The most potent nanoconjugate **18** increased tBHP-induced cytotoxicity in SH-SY5Y cells by approximately 7.5-fold, which indicated a strong effect at decreasing oxidative stress resistance. In terms of SAR on the alkyl ester chain, it is worth noting that compounds containing a disulfide-bridge (**18**, **20**) acted as more potent sensitizers to tBHP than their counterparts without this moiety (**17**, **19**). This is consistent with previous observations on the incorporation of chemically more vulnerable linkers that further facilitate the release of an active substance from the conjugate, which also affects the strength of the bioactivity.

Nearly all compounds exerted protective effects on MRC-5 cells, and the nanoformulations showed a tendency to further increase this effect compared with their respective parent ecdysteroids. In some cases, the effect was highly significant (e.g., compound **10** vs **17** or **18)**. Compound **18** was not only the strongest sensitizer to oxidative stress in SH-SY5Y cells, but also the strongest protective agent in MRC-5 fibroblasts. Because of this, the antitumor selectivity of the oxidative stress inducer tBHP, calculated as the IC_50_^MRC-5^/IC_50_^SH-SY5Y^, markedly increased from 5.9 to 153.1 following treatment with 0.5 μM of the nanoformulated ecdysteroid conjugate **18**. A similar, though somewhat less potent effect was observed for compounds **17** and **19**, which also sensitized neuroblastoma cells to oxidative damage while protecting fibroblasts and therefore increased the tumor selectivity of tBHP to 117.1 and 82.2, respectively (Table S72).

#### Morphological Assessment of Cells

**Figure 2:**
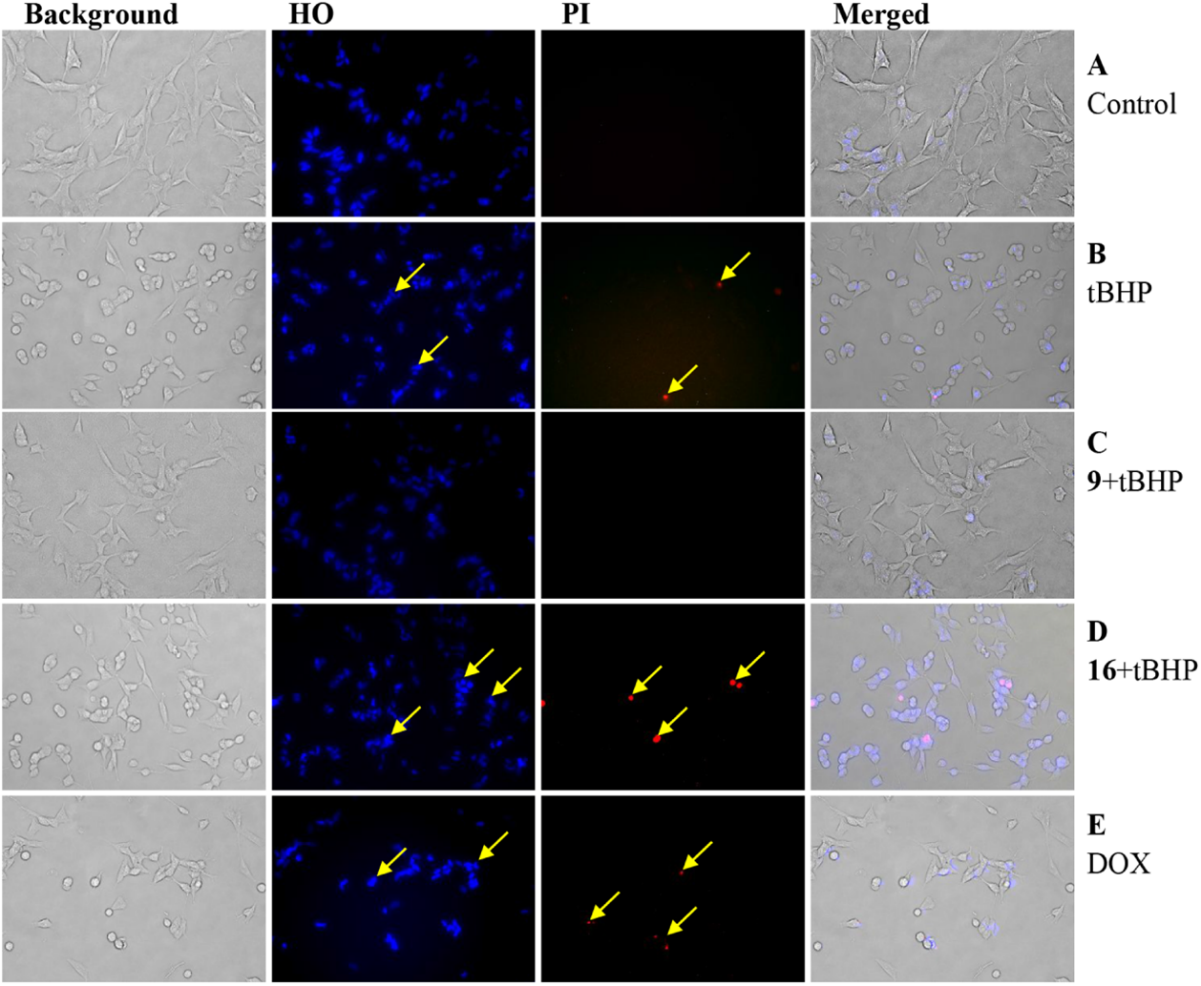
Representative fluorescent microscopic images (×10 objective) for apoptotic nuclear assessment using Hoechst 33258 staining of SH-SY5Y cells after a 48-h incubation as a control (**A**), in the presence of 50 μM tBHP (**B**), pretreated with compound **9** before exposure to 50 μM tBHP (**C**), pretreated with compound **16** (0.5 μM) before exposure to 50 μM tBHP (**D**), or treated with doxorubicin (2 μM) as a positive control (**E**). Apoptotic and necrotic cells are indicated with arrows in the HO and PI staining, respectively.

To assess the morphological effects on SH-SY5Y cells, compound **9** and its nanoconjugate **16** were selected as a representative compound pair exerting protective (**9**) and sensitizing effects (**16**) on this cell line (Figure 2). Compared with the control cells (Figure 2A), 50 μM tBHP treatment resulted in obvious signs of cellular damage, including reduced cell count, cell shrinkage, and cytoplasmic fragmentation and granularity. Furthermore, Hoechst 33258 and propidium iodide (PI) staining revealed oxidative stress-induced apoptosis and a very low number of necrotic cells, respectively (Figure 2B).

Pretreatment with compound **9** (0.5 μM) exerted a pronounced protective effect against tBHP-induced cytotoxicity. This was evidenced by the preservation of cell morphology, sustained growth, and the maintenance of a desirable cell confluence, along with a marked decrease in the apoptotic and necrotic cell population (Figure 2C). The opposite effect was observed when administering the squalenoylated nanoconjugate **16** (0.5 μM), and the morphological observations along with the increased population of both apoptotic and necrotic cells supported the sensitizing effect on oxidative damage (Figure 2D). On the other hand, when treated with doxorubicin (2 μM), SH-SY5Y cells showed a mix of apoptotic and necrotic features, with Hoechst staining highlighting apoptotic nuclear changes and PI staining marking necrotic cells (Figure 2E). Doxorubicin exerts its cytotoxic effects on SH-SY5Y neuroblastoma cells primarily through DNA adamage, ROS generation, and the induction of apoptosis.^52^ This oxidative damage triggers mitochondrial dysfunction, leading to the activation of intrinsic apoptotic pathways.^53^ This results in the release of cytochrome c from mitochondria and the activation of caspases, which ultimately drive programmed cell death, contributing to the killing effect of doxorubicin in SH-SY5Y cells.

#### Effect of Compound 9 and 16 on ROS Levels and AKT Phosphorylation in SH-SY5Y Cells

**Figure 3:**
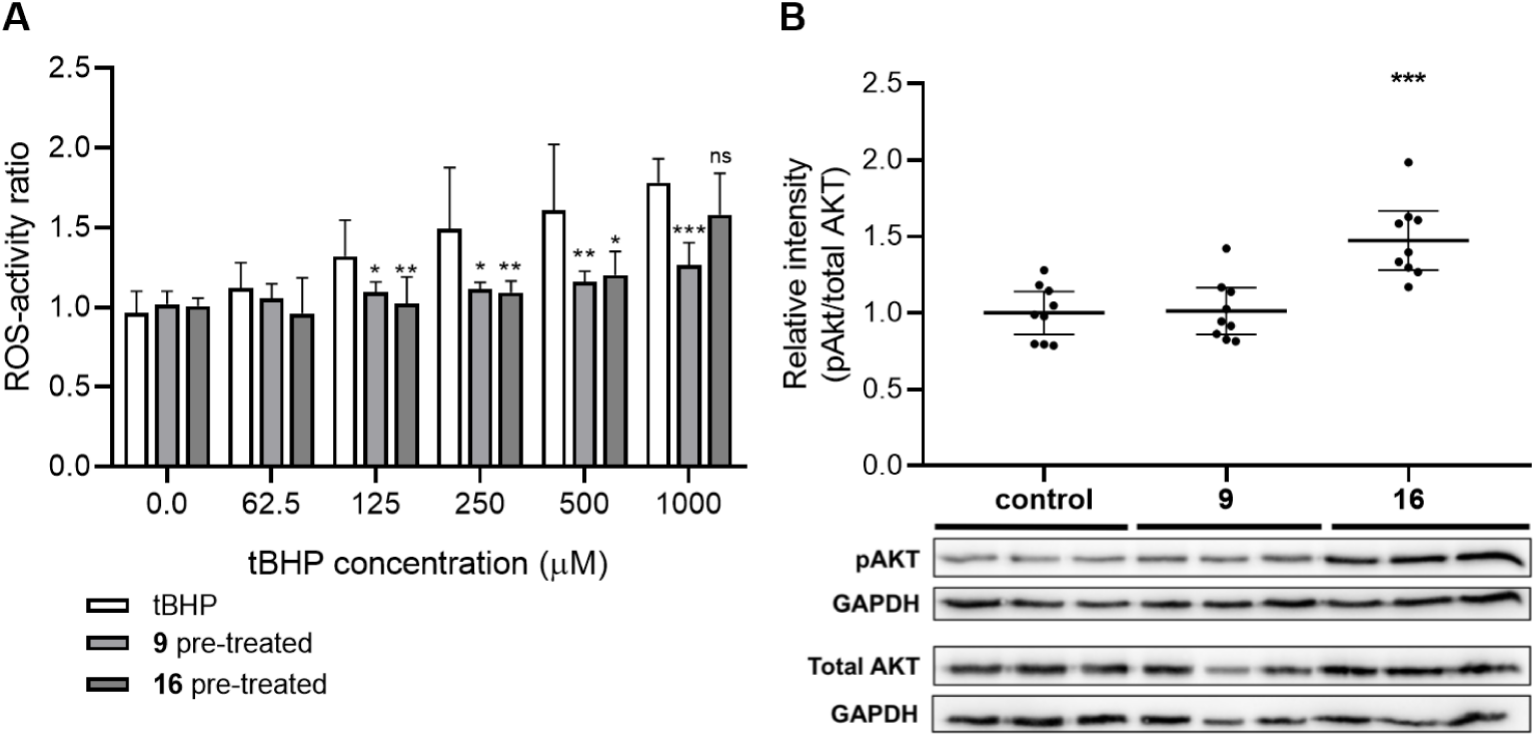
**A**: Effect of compound **9** and **16** on tBHP-induced increase in ROS levels. SH-SY5Y cells were pretreated with 0.5 μM of compound **9** or **16** for 48 h, and then oxidative stress was induced by a 1 h treatment with tBHP (62.5–1000 μM). The ROS-activity of pretreated samples were compared to that of the corresponding sample treated with tBHP-alone using one-way ANOVA with Tukey posthoc test. Results are expressed as mean values ± SEM of the data on two separate measurements with triplicates, ns indicates *p* > 0.05, * and ** indicate *p* < 0.05 and *p* < 0.01, respectively. **B**: Western blot of pAKT and AKT in SH-SY5Y lysates nontreated (control) or treated with compounds **9** or **16** for 48 h. Representative bands (3 per treatment) are shown. Relative intensity of pAKT was compared to that of total AKT for each sample (*n* = 9). Values were normalized to those of the average of the control samples. Data points represent a biological replicate for each treatment. The line shows the median values with whiskers showing the 95% confidence interval. ^***^ indicates *p* ≤ 0.001 by one-way ANOVA followed by Dunnett’s multiple comparisons correction.

To evaluate the effect of the two selected representative compounds, **9** and its nanoconjugate **16**, on tBHP-induced oxidative stress in SH-SY5Y cells, we performed intracellular ROS-activity measurements. tBHP treatment induced a dose-dependent increase in ROS-activity of the cells (Figure 3A). Pretreatment with either of the compounds could counteract oxidative stress induced by 125–500 μM tBHP. This was, however, different when tBHP was administered at the highest, 1000 μM dose; in this case only the free ecdysteroid (**9**) decreased ROS activity and the nanoconjugate **16** did not. While this is in line with our observation that compound **9** protects the cells from tBHP-induced oxidative stress, it is important to mention that compound **16** did not increase ROS levels in SH-SY5Y cells even though it clearly promotes tBHP-induced cell death (Figure 2). Accordingly, our results suggest that squalenoylated ecdysteroid nanoparticles do not act via increasing oxidative stress, but they somehow render the cells more sensitive to it.

An intriguing aspect of the studied compounds on SH-SY5Y neuroblastoma cells was that squalenoylation switched the protective effect of nonconjugated ecdysteroids into oxidative stress-potentiating effects of the ecdysteroid nanoparticles. A reasonable explanation may be offered by the well-known protein kinase B (Akt) activating effect of ecdysteroids in skeletal muscle cells, as reported for 20E, calonysterone (**7**), and their side-chain-cleaved derivatives, including compound **8**.^42,54^ Akt kinase activation promotes protein synthesis, cell growth, and survival;^55^ however, Akt hyperphosphorylation is associated with quite opposite outcomes: protein degradation, cell death, and a general increase in ROS-susceptibility.^56,57^ Therefore, we hypothesized that while the nonconjugated ecdysteroids induce Akt phosphorylation and increase cell viability, at the same time, squalenoylated derivatives promote Akt hyperphosphorylation-mediated ROS-sensitization. To test this hypothesis, we investigated the effect of compounds **9** and **16** on Akt phosphorylation using the same treatment conditions (0.5 μM, 48 h) as before; results are shown in Figure 3B. Interestingly, treatment with compound **9** did not result in detectable activation of Akt in our experimental setup, while its squalenoylated derivative **16** significantly increased Akt phosphorylation and consequent activation.

Akt requires phosphatidylinositol-triphosphate (PIP3), a cell membrane-associated source for phosphorylation;^55^ therefore, its operation is restricted to the proximity of cellular membranes.^58^ The squalene moiety of the conjugated ecdysteroid derivatives, such as **16**, confers a higher lipophilicity to these compounds as compared to their nonsqualenoylated counterparts, such as **9**. This should result in different accumulation rate within phospholipid bilayers, spatially coinciding with the PIP3 source for Akt. We hypothesize that such accumulation must significantly alter the intracellular distribution of ecdysteroids upon being released from their squalene-conjugated prodrugs (e.g., **16**) compared with those entering the cell in free form (e.g., **9**). Consequently, the increased concentration of ecdysteroids near the operational sites of Akt may explain the difference between the effects of free vs conjugated ecdysteroids on Akt phosphorylation, and therefore on ROS-induced cell death. At the same time, compound **9** could counteract higher amounts of tBHP, therefore it acted as a more potent antioxidant than **16** (Figure 3A). Altogether, these differences between the bioactivity profiles of compound **9** and its prodrug **16** concerning the ROS-Akt interplay provide a reasonable mechanistic background to their different effect on cell death.

The selectivity of squalenoylated ecdysteroids toward tumor cells may also be associated with the same target mechanism. Akt kinases are considered oncoproteins whose dysregulation and/or the overexpression contribute to the high survival capacity of cancer cells. In neuroblastoma, high metastatic potential and poor prognosis are associated with hyperactivation of Akt2.^59^ It is tempting to speculate that our nanoconjugates activate Akt at a level that still protects normal cells from oxidative stress, but this activation is already enough to manifest as ROS hypersensitivity in cancer cells. Further studies will be needed to verify this effect.

Our results suggest that compounds **16**–**18** are potential adjuvant agents to improve the selectivity of oxidative stress-related antitumor therapeutic regimens, including radiotherapy, that are used to treat neuroblastoma. Squalenoylated ecdysteroid nanoparticle prodrugs have the potential to sensitize tumors, while protecting nearby healthy tissues against ROS that form during irradiation.

Finally, considering that the neuroblastoma cell line used in our study also serves as an attractive pharmacological model for neuroprotection, the free ecdysteroids **7**–**9** may have the potential to protect cells from oxidative stress- or injury-induced neurotoxicity, with implications for various central nervous system pathologies. Future studies using neurons differentiated from SH-SY5Y cells will be necessary to evaluate the neuroprotective potential of these compounds. Furthermore, such studies will be necessary to determine whether there is any potential risk for neurotoxicity in the case of nanoconjugates **16**–**18**.

## Experimental Section

### Preparation of Self-Assembly Inducer Side Chains

The self-assembly inducer squalene (**1**) was functionalized and conjugated with sebacic acid (**3**) or 4,4′-dithiodibutyric acid (**4**) following as previously described.,^19,20,33^

### Multi-Step Synthesis of 1,1′,2-Tris-norsqualenoyl Alcohol (2)

#### Step 1: Synthesis of 2-Hydroxy-3-bromosqualene

Squalene was obtained from Sigma (Merck KGaA, Darmstadt, Germany) and used without further purification. Squalene (10 g) was dissolved in 70 mL of tetrahydrofuran (THF). The solution was supplemented with 20 mL of HPLC-grade distilled water. After thorough manual stirring, 30 mL of THF was added. Subsequently, 6.5 g of *N*-bromosuccinimide (NBS) was added in small portions and the mixture was stirred at room temperature for 30 min. THF was then evaporated, and brine (35 mL) was added to the aqueous residue and the resulting compounds were extracted with 4 × 30 mL of ethyl-acetate. The combined organic fractions were dried over Na_2_SO_4_ and after filtration, ethyl-acetate was evaporated using a rotary evaporator. The dry product was dissolved in *n*-hexane (50 mL) and NBS was removed by filtration. Following the evaporation of *n*-hexane, approximately 11 g of oily residue was obtained.

#### Step 2: Synthesis of 2,3-Oxidosqualene

The product mixture from Step 1 (approximately 11 g) was, without any further purification, dissolved in 100 mL of methanol. Then, 2 equiv of K_2_CO_3_ (6 g) was added and the mixture was stirred at room temperature for 3 h. Afterward, methanol was evaporated by a rotary evaporator, brine (50 mL) was added, and the products were extracted with 4 × 30 mL ethyl-acetate. The combined organic solution was dried using Na_2_SO_4_, filtered, and ethyl-acetate was evaporated under reduced pressure. Approximately 10.5 g of the products were obtained.

#### Step 3: Synthesis of 1,1′,2-Tris-norsqualenoyl Aldehyde

The product mixture from Step 2 (approximately 10.5 g) was used without chromatographic purification. The dry compounds were dissolved in 50 mL of 1,4-dioxane. Then, 1.8 equiv of H_5_IO_6_ (10 g) was dissolved in 20 mL of water. The aqueous and organic solutions were combined and stirred for 3 h at room temperature. The 1,4-dioxane was evaporated, brine (50 mL) was added to the aqueous residue, and the products were extracted with 4 × 30 mL of ethyl-acetate. The combined organic fractions were dried over Na_2_SO_4_, filtered, and supplemented with silica gel (approximately 10 g). Evaporation of ethyl-acetate under reduced pressure enabled dry sample loading for the subsequent flash chromatographic purification of 1,1′,2-tris-norsqualenoyl aldehyde. The chromatographic separation was done using a 330 g silica column (RediSep Gold, TELEDYNE Isco, Lincoln, NE, USA) with *n*-hexane as the mobile phase (flow rate: 200 mL/min). Thus, 2.1 g of 1,1′,2-tris-norsqualenoyl aldehyde was obtained (combined yield of Steps 1–3:21.9%).

#### Step 4: Synthesis of 1,1′,2-Tris-norsqualenoyl Alcohol

1,1′,2-tris-norsqualenoyl aldehyde (2.1 g) was dissolved in 50 mL of ethanol. Two equiv of NaBH_4_ (0.413 g) were added to the solution and the reaction mixture was stirred at room temperature for 24 h. The reaction was terminated by adding 4 equiv of CH_3_COOH (4 mL) to the mixture. After the evaporation of ethanol in a rotary evaporator, brine (200 mL) was added to the aqueous residue, and the compounds were extracted with 3 × 65 mL of *n*-hexane. The combined organic fractions were dried using Na_2_SO_4_, and following filtration, silica gel was added (approximately 8 g) to the solution. The evaporation of *n*-hexane under reduced pressure enabled dry sample loading for flash chromatographic purification of 1,1′,2-tris-norsqualenoyl alcohol (see Table S72). As a result of our efforts, a total of 1.29 g of 1,1′,2-tris-norsqualenoyl alcohol was obtained (isolated yield of Step 4:61.1%, combined yield of Step 1–4:13.7%).

### General Procedure for the Preparation of 1,1′,2-Tris-norsqualenoyl Alcohol-sebacic Acid- (5) and 1,1′,2-Tris-norsqualenoyl Alcohol-4,4′-dithiodibutyric Acid Esters (6)

1,1′,2-tris-norsqualenoyl alcohol was dissolved in anhydrous methylene chloride at a 1 mmol/mL concentration. Subsequently, 2 equiv of the corresponding dicarboxylic acid (sebacic acid for product **3**, and 4,4′-dithiodibutyric acid for product **4**), 0.7 equiv of 4-dimethylaminopyridine (DMAP), and 1.2 equiv of 1-(3-(dimethylamino)propyl)-3-ethylcarbodiimide hydrochloride (EDC·HCl) was added. The mixtures were stirred at room temperature under an argon atmosphere for 24 h. The mixtures were neutralized with 10% aq. NaHCO_3_ solution. Following the evaporation of methylene chloride under reduced pressure, brine was added to the aqueous residue, and the compounds were extracted with methylene chloride. The resulting organic fractions were combined, dried over Na_2_SO_4_, and filtered. Silica gel was added to the reaction mixture, and the compounds were adsorbed on its surface through solvent evaporation under reduced pressure. The samples were subjected to flash chromatographic purification (see Table S72). After successful separation, the yield was 24.3% for compound **5** and 36.4% for compound **6**.

### Preparation of Ecdysteroid Substrates as Starting Materials for the Preparation of Self-Assembling Drug Conjugates

#### Chromatographic Isolation and Semi-Synthesis of Naturally Occurring Ecdysteroids

The natural ecdysteroids calonysterone (**7**) and ajugasterone C (**11**) were isolated from *Cyanotis arachnoidea*(60) and *Serratula wolffii*,^61^ respectively. The pure compounds were subsequently used as starting materials for our semisynthetic work.

#### General Procedure for the Preparation of Side-Chain Cleaved Ecdysteroid Derivatives

Calonysterone (**7**) or ajugasterone C (**11**) was dissolved in methanol at a 12.5 mg/mL concentration. One equiv of (diacetoxyiodo)benzene (PIDA) was added. The mixtures were stirred for 45 min at room temperature, and subsequently neutralized with 5% aqueous NaHCO_3_ solution. After evaporation under reduced pressure, the dry residue was redissolved in methylene chloride and silica gel was added. After evaporation, the dry residue was subjected to flash chromatographic separation as detailed in Table S72. The yield for the side-chain-cleaved derivatives of calonysterone and ajugasterone C were 48.8% (compound **8**) and 71.9% (11α-hydroxypoststerone), respectively.

### General Procedure for the Preparation of Ecdysteroid Acetonides

Calonysterone, ajugasterone C, or their side-chain-cleaved derivatives were dissolved in acetone at 1 g/100 mL. The solutions were supplemented with 1 g of phosphomolybdic acid for each gram of ecdysteroid substrate. The mixtures were sonicated at room temperature for 30 min, and then neutralized with 5% aqueous NaHCO_3_ solution. After the evaporation of acetone using a rotary evaporator, methylene chloride (100 mL) was added to the aqueous residue and liquid–liquid extraction was performed (3 × 100 mL). The combined organic fractions were dried over Na_2_SO_4_, filtered, and after evaporation of methylene chloride under reduced pressure, the dry residue from each reaction was subjected to flash chromatographic separation (see Table S72). The yields of compounds **9**, **10**, **12,** and **13** were 49.8%, 55.1%, 67.6%, and 65.7%, respectively.

### Preparation of Ecdysteroid Containing Self-Assembling Drug Conjugates

The selected ecdysteroid starting material (**7**, **8**, **9**, **10**, **11**, **12**, or **13**) was dissolved in anhydrous methylene chloride at a 10 mg/mL concentration. Then, 1.2 equiv of the self-assembly inducer side-chain entity (**5** or **6**), 2 equiv of DMAP, and 2.5 equiv of EDC·HCl were added to the solution. For the ecdysteroid derivatives **7** and **11**, the reaction mixture was stirred at room temperature under argon atmosphere for 2 h, whereas for compounds **8**, **9**, **10**, **12,** and **13,** the stirring was allowed to proceed for 24 h. Each reaction was terminated with 5% aqueous NaHCO_3_ and subjected to liquid–liquid extraction with 3 × 50 mL of methylene chloride. Combined organic fractions were dried over Na_2_SO_4_ and following the evaporation of methylene chloride with a rotary evaporator, supercritical fluid chromatography (SFC) was used for the isolation of the products (see Table S72), which resulted in ecdysteroid conjugates **14**, **15**, **16**, **17**, **18**, **19**, **20**, and **21** in yields of 21.8%, 21.3%, 17.4%, 60.1%, 46.4%, 56.6%, 72.5%, and 66.8%, respectively.

### Chromatography Conditions

The conditions for chromatographic purification of the intermediate and target compounds are summarized in Table S72. Synthetic reactions were monitored by thin-layer chromatography. Kieselgel 60F_254_ silica plates were purchased from Merck (Merck KGaA, Darmstadt, Germany) and the characteristic spots of materials were examined under UV light at 254 and 366 nm.

Flash chromatography was carried out on a CombiFlash Rf+ Lumen instrument (TELEDYNE Isco, Lincoln, NE, USA) equipped with ELS and diode array detectors. Isolation of the products was done using commercially acquired RediSep NP-silica gel flash columns (TELEDYNE Isco, Lincoln, NE, USA).

Preparative HPLC was accomplished using the Armen Spot Prep II integrated HPLC purification system (Gilson, Middleton, WI, USA) equipped with dual-wavelength detection. Purity evaluation of the isolated nonconjugated ecdysteroids (compounds **8**, **9**, **10**, **12**, **13**) was performed on a Jasco HPLC instrument (Jasco International Co. Ltd., Hachioji, Tokyo, Japan) equipped with a diode array detector. The analysis was done using a Kinetex, 5 μm, XB-C18, 100 Å, 250 mm × 4.6 mm column (Phenomenex Inc., Torrance, CA, USA) by applying a 1 mL/min flow rate, using the peak area % data of the PDA chromatogram recorded between 210 and 410 nm.

Supercritical fluid chromatographic purification was carried out on a Jasco SFC instrument (Jasco International Co. Ltd., Hachioji, Tokyo, Japan) equipped with a PDA detector. The instrument was used with a Phenomenex Luna 5 μm, Silica (2), 100 Å, 250 mm × 21.2 mm HPLC column (Phenomenex Inc., Torrance, CA, USA) with a 15 mL/min flow rate for preparative purposes. Purity analysis of the isolated compounds **14**–**21** was performed on a Phenomenex Luna 5 μm, Silica (2), 100 Å, 250 mm × 4.6 mm HPLC column (Phenomenex Inc., Torrance, CA, USA).

All compounds possessed a purity of ≥ 95% by means of HPLC (**8**–**10**, **12**, and **13**) or SFC (**14**–**21**) analysis.

### Preparation and Examination of Self-Assembled Ecdysteroid Nanoparticles

A 16 mg aliquot of each ecdysteroid conjugate (**14**, **15**, **16**, **17**, **18**, **19**, **20**, or **21**) was dissolved in 2 mL of freshly distilled acetone (8 mg/mL). Next, 1 mL of these solutions was added to 2 mL of Milli-Q (Merck KGaA, Darmstadt, Germany) ultrapure-grade water dropwise using Hamilton syringes. The procedure was carried out slowly at RT with mild stirring (350 rpm). The self-assembly of the conjugates and the formation of nanoparticles occurred immediately because of secondary interactions between the squalene chains and the aqueous medium. The organic solvent (acetone) was evaporated at RT under reduced pressure, and the resulting aqueous nanosuspensions (4 mg/mL) were stored at 4 °C.

The colloid chemical characteristics of the self-assembled nanoparticles in ultrapure water were evaluated by a dynamic light scattering (DLS) technique on a Malvern Zetasizer Nano ZS instrument (Malvern Instruments, Malvern, UK). Prior to the analysis, samples were diluted to a 150 μg/mL concentration with ultrapure water and were subsequently aliquoted into disposable folded capillary cells. The measurements were conducted at a consistent 25 °C temperature, and the capillary cells were washed using distilled water between measurements of each sample. The average hydrodynamic diameter (Z-Average), polydispersity index (PdI), and zeta potential of the nanosuspensions were determined.

The nanoassemblies of compound **18** were also subjected for transmission electron microscopy. The sample was prepared using negative staining. Briefly, a drop from the self-assembled nanosuspension (4 mg/mL) was placed onto a copper grid (CF200-Cu) for 1 min and fixed with 2.5% glutaraldehyde for 2 min. The excess fixing agent was removed, followed by the application of a 0.5% aqueous solution of uranyl acetate (UA), which was left to settle for 1 min. Excess UA was then absorbed, and the grid was air-dried at room temperature. The sample was visualized using an FEI Tecnai G2 20 X Twin instrument (Thermo Fisher Scientific Inc., Waltham, MA, USA).

### Structure Elucidation

The mass spectra of the compounds were recorded on an Agilent 1,100 LC-MS instrument (Agilent Technologies, Santa Clara, CA, USA) coupled with a Thermo Q-Exactive Plus orbitrap analyzer (Thermo Fisher Scientific, Waltham, MA, USA) in positive mode.

NMR spectroscopy: ^1^H (950, 800, and 500 MHz) and ^13^C (239, 200, and 125 MHz) NMR spectra were recorded at room temperature on a Bruker Avance III spectrometer equipped with cryo probe heads. The NMR experiments were performed at 500/125 MHz (**9**, **10**, and **14**–**16**), 800/200 MHz (**12**, **18**, and **20**), or 950/239 MHz (**17**). Approximately 1–5 mg of **9**, **12**, **14**–**18,** and **20** were dissolved in 0.6 mL of chloroform-*d*, compound **10** was dissolved in methanol-*d*_4_, and the solutions were transferred to 5 mm NMR sample tubes. The chemical shifts are presented on the δ-scale and referenced to the solvent chloroform-*d*: δC = 77.00 and δH = 7.27 ppm, and δC = 49.1 and δH = 3.31 ppm for methanol-*d*_4_. The pulse programs for all experiments [^1^H, ^13^C, DEPTQ, APT, 1D sel-ROESY (τmix: 300 ms), 1D sel-TOCSY, 1D sel-INEPT (^13^C), HSQC, edHSQC, HMBC and band-selective-HSQC and -HMBC] were taken from the Bruker software library. For 1D measurements, 64 K data points were used to yield the FID.

### Cell Culture

The SH-SY5Y human neuroblastoma cell line, used as an in vitro model to study neurodegenerative diseases,^62^ was purchased from the ATCC (Manassas, Virginia, USA). The cells were grown in *T*-75 flasks and incubated at 37 °C and 5% CO_2_ in a humidified incubator. The basic growth medium was used and composed of Eagle’s Minimum Essential Medium (EMEM) supplemented with 15% heat-inactivated fetal bovine serum (FBS), 1% penicillin/streptomycin (1x Pen/Strep) and 1% l-glutamine (2 mM Glutamine). After reaching a confluency of 80–90%, the adherent cells were detached from the flask and collected using a TrypLE Express solution (Thermo Scientific, Waltham, Massachusetts, USA). The cells were subsequently recultured in fresh growth medium. The intact, human fetal lung fibroblast cell line, MRC-5 was used to test selectivity of the compounds toward cancer cells. MRC-5 cells were maintained in low-glucose Dulbecco’s Modified Eagle’s Medium supplemented with 20% FBS, 1% 1x Pen/Strep and 2% l-glutamine. All media and supplements, if not otherwise specified, were obtained from Capricorn Scientific Ltd. (Ebsdorfergrund, Germany). Quantification of viable cells was done using a LUNA II Automated Cell Counter (Logos Biosystem Ltd., Anyang-si, Gyeonggi-do, South Korea) following the addition of a 10% trypan blue solution.

### Cell Seeding and Treatment

SH-SY5Y cells were seeded into 96-well cell culture plates at a density of 5 × 10^3^ cells per well. The plates were incubated for 24 h in a humidified incubator at 37 °C and 5% CO_2_. Ecdysteroid derivatives were dissolved in dimethyl sulfoxide (DMSO) to prepare stock solutions at 10 mM concentration. The nanosuspensions of ecdysteroid containing self-assembling drug conjugates were diluted by ultrapure water to reach a concentration of 3.5 mM, and these were taken as the stock solutions to be further diluted with medium. The effect of DMSO or ultrapure water on cell viability was determined at their highest concentration (i.e., 0.1% DMSO, and 0.3% ultrapure water), and no effect of either was observed as compared to the untreated cell controls. Cells were treated with concentrations of 10, 5.0, 2.5, 1.0, or 0.5 μM of compound **7**, **8**, **9**, **14**, **15**, or **16**. All other compounds were tested at a single dose of 0.5 μM. The plates were incubated for 48 h under the same culture conditions. Subsequently, freshly prepared tBHP (1,000, 500, 250, 125, 62.5, 31.25, 15.62, 7.81, 3.90, and 1.95 μM), diluted in PBS, was added to the plates and incubated for 4 h. Compound selectivity was assessed by performing the same experiment on MRC-5 cells, a noncancerous lung fibroblast cell line (ATCC, Manassas, Virginia, USA). The experimental setup also included negative controls, in which cells were subjected only to EMEM treatment, and vincristine was used as a positive control on both SH-SY5Y and MRC-5 cells.

### Cell Viability Assay

Cell viability was determined by the 3-(4,5-dimethylthiazol-2-yl)-2,5-diphenyltetrazolium bromide [MTT Duchefa Biochemie BV, Haarlem, The Netherlands] assay.^63^ After treating the cells with both the compound and tBHP, a 20 μL volume of MTT solution (5 mg/mL in PBS) was added to each well and incubated for 4 h. The liquid medium was carefully aspirated, and 100 μL of DMSO was added to each well. The plates were placed on a shaker for 30 min to dissolve the purple formazan crystals. Subsequently, the optical density was measured at 545 nm using a microplate UV–vis reader (SPECTROstar Nano, BMG Labtech GmbH, Offenburg, Germany).

Data was collected from two separate experiments, each conducted in triplicate, to determine the antiproliferative effect of the compounds. IC_50_ values (half-maximal inhibitory concentration) were calculated using a logarithmic inhibitor vs normalized response nonlinear regression model in the software. The results incorporated error bars denoting the standard error of the mean (SEM) encompassing the IC_50_ values. Considering the large differences between many of the IC_50_ values in either direction, a 2-fold difference was considered a relevant threshold instead of statistical significance.

### Hoechst 33258/Propidium Iodide (HOPI) Fluorescent Staining

Fluorescence staining was performed to observe the morphological changes associated with necrosis and apoptosis induced by the compounds.^64^ Specifically, Hoechst 33258 was used to observe DNA condensation, nuclear fragmentation, and the distinctive characteristics associated with apoptosis. Because propidium iodide cannot penetrate live cells, it was used to identify necrotic cells within a cell population. Initially, SH-SY5Y cells were seeded into a 6-well plate at a density of 1 × 10^5^ cells per well and allowed to attach overnight. The cells were pretreated with the desired concentrations of the test compound and incubated for 48 h under the specified cell culture conditions. The cells were treated and divided into untreated (control), 50 μM tBHP alone, and tested compound at 0.5 μM + 50 μM tBHP. Since doxorubicin (DOX) exhibited cytotoxic effects on SH-SY5Y neuroblastoma cells,^65^ it was used as a positive control at a concentration of 2 μM. tBHP was added to the plates after 48 h of pretreatment with the compounds. After washing the cells with PBS, they were subjected to staining using a medium containing Hoechst 33258 (HO, 5 μg/mL) and propidium iodide (PI, 1 μg/mL). Staining was carried out in the dark for 90 min, and the medium was replaced. Images (five for each condition) were directly captured using a Nikon Eclipse TS100 fluorescent microscope (Nikon Instruments, Amstelveen, Netherlands) equipped with suitable filters.

### Intracellular ROS Measurement

Intracellular ROS levels were analyzed using the fluorometric intracellular ROS kit (Sigma-Aldrich, product number MAK143), following the manufacturer’s instructions. This kit uses a sensor that reacts with ROS, producing a fluorescent signal proportional to the amount of ROS in exposed cells. For the experiment, cells were seeded in a 96-well black plate with clear bottoms at a density of 10,000 cells per well in 90 μL of EMEM with 15% FBS. After overnight incubation under the same culturing conditions, the cells were treated with compound **9** or **16** at a concentration of 0.5 μM in 10 μL for 48 h. To induce ROS, the cells were then exposed to tBHP at various concentrations for 1 h in 5% CO_2_, 37 °C incubation. The master reaction mix was prepared following the manufacturer’s instructions, and 100 μL of this mixture was added to each well and incubated for 1 h. Finally, the fluorescence intensity was measured using a FluoStar Optima (BMG Labtech GmbH, Offenburg, Germany) fluorescence spectrometer, with an excitation wavelength set at 490 nm and an emission wavelength at 525 nm. Each treatment was conducted with 6 replicates to ensure consistency and accuracy of the results.

### Western Blot Analysis

Western blot analysis was performed as described before, with minor modifications.^66^ Briefly, SH-SY5Y cells were seeded into 6-well plates at a density of 1 × 10^6^ cells per well and allowed to adhere for 24 h at 37 °C and 5% CO_2_. Cells were then treated with 0.5 μM of compounds **9** or **16** for 48 h, while untreated cells served as the control. Following treatment, cells were washed once with cold PBS (Capricorn Scientific GmbH, Ebsdorfergrund, Germany) to remove residual media and compounds. For cell lysis, 100 μL of cold RIPA buffer (SantaCruz Biotechnology, Dallas, TX, USA) containing 1% phosphatase and protease inhibitor cocktail (sodium orthovanadate, PMSF and protease inhibitor cocktail; SantaCruz Biotechnology, Dallas, TX, USA) was added to each well on ice, and cells were collected using a cold cell scraper. Lysates were transferred into 2 mL Eppendorf tubes and kept on ice. Samples were then centrifuged at 12,000*g* for 20 min at 4 °C to separate cell debris. After centrifugation, supernatant containing the total protein was carefully collected into new tubes and kept on ice or −80 °C for further analysis. Before proceeding with Western blot analysis, protein content of the supernatant was quantified using the Pierce BCA (Bicinchoninic Acid) protein assay kit (Thermo Fisher Scientific Inc., Waltham, MA, USA). According to the manufacturer’s instructions, standard of known concentrations (0 – 2000 μg/mL) of bovine serum albumin (BSA) diluted in RIPA buffer was used to generate a standard curve. Absorbances of standard and unknown samples were measured at 562 nm by SpectroStar Nano spectrophotometer (BMG Labtech GmbH, Ortenberg, Germany), and the total protein concentration of unknown samples was interpolated from the standard curve. Thirty μg of protein were separated using 8% sodium dodecyl sulfate-polyacrylamide gel. SDS-PAGE was carried out at 110 V for 2 h. Proteins were then transferred to nitrocellulose membranes (Protran) at 260 mA for 2 h. Membranes were washed with 20 mM Tris-HCl, pH 7.5, 150 mM NaCl, 0.05% Tween-20 (TTBS, Merck). Nonspecific protein binding was blocked with 5% nonfat dry milk in TTBS for 1 h, then membranes were incubated overnight at 4 °C with phospho-Akt (Ser^473^) antibody (1:1000, Cell Signaling, cat#9271S) or total Akt antibody (1:1000, Cell Signaling, cat#9272S) diluted in TTBS. Membranes were rinsed and probed with antirabbit IgG, HRP-linked antibody (1:10000, Cell Signaling, cat#7074S) in TTBS. For loading control, the membranes were probed with GAPDH antibody (Merck, cat#MAB374, 1:500, overnight at 4 °C), followed by HRP-linked antimouse IgG (1:10000, Jackson, cat#115–035–146). Membranes were analyzed with the Image Lab software version 6.0 (Bio-Rad Laboratories). Densities of the bands of interest from each membrane were normalized to the corresponding GAPDH values.

Relative intensity of pAKT was compared to that of total AKT for each sample (*n* = 9). The two treated groups were compared to the untreated control using one-way ANOVA followed by Dunnet’s posthoc test.

### Statistical Analysis

All data and statistical analysis were performed using GraphPad Prism 9 Software (www.graphpad.com). Unless otherwise stated, all statistical tests were two-tailed, and results were considered significant when *p* < 0.05.

## Supporting Information Available

HRMS spectra of compounds **9**, **10**, **12**, **14**–**18**, and **20** (S1–S9), characteristic NMR spectra of compounds **9**, **10**, **12**, **14**–**18**, and **20** (S10–S71), chromatographic conditions for compound purification (S72), summarized DLS data (S73), particle size distribution plots of the nanosuspensions of compounds **14–20** at different storage times (S74–S95), antiproliferative effect of compounds **7**–**10** and **12**–**21** on SH-SY5Y and MRC-5 cells (S96), effect of tBHP on the viability of SH-SY5Y cells alone and after pretreatment with different concentrations of compounds **7**–**9** or **14**–**16** (S98–S100), TEM images of the nanoassemblies of compound **18** (S101), RP-HPLC-PDA max plot chromatogram of ecdysteroid lead compound **9** (S102), and SFC-PDA max plot chromatogram of ecdysteroid lead compound **16** (S103) (PDF)
Molecular formula strings of the compounds along with their pharmacological activity (CSV)

## List of abbreviations

20E: – 20-hydroxyecdysone
6-OHDA: – 6-hydroxydopamine,
ANOVA: – analysis of variance
APT: – attached proton test
BBB: – blood-brain barrier
CNS: – central nervous system
CSF: – cerebrospinal fluid
DEPTQ: – distorsionless enhancement by polarization transfer including the detection of quaternary nuclei
DLS: – dynamic light scattering
DMAP: – 4-dimethylaminopyridine
DMSO: – dimethyl-sulfoxide
EDC·HCl: – 1-(3-(dimethylamino)propyl)-3-ethylcarbodiimide hydrochloride;
ELS: – evaporative light scettering
EMEM: – Eagle’s Minimal Essential Medium;
FBS: – fetal bovine serum
FID: – free induction decay
HMBC: – heteronuclear multiple-bond correlation
HO: – Hoechst 33258
HPLC: – high-preformance liquid chromatography
HRMS: – high resolution mass spectrometry
(ed)HSQC: – (edited) heteronuclear correlation quantum coherence
IC_50_: – half maximal inhibitory concentration
(sel-)INEPT: – (selective) insensitive nuclei enhanced by polarization transfer
LDL: – low-density lipoprotein
MDR: – multidrug-resistance
MTT: 3-(4,5-dimethyl-2-thiazolyl)-2,5-diphenyl-2H-tetrazolium-bromide;
NBS: – N-bromosuccidimide
NMR: – nuclear magnetic resonance
PDA: – photodiode array
PdI: – polydispersity index
PI: – propidium-iodide
PIDA: – (diacetoxyiodo)benzene
PIP3: – phosphatidylinositole-3-phosphate
PMA: – phosphomolybdic acid
(sel-)ROE(SY): – (selective) rotating-frame nuclear Overhauser effect (spectroscopy)
ROS: – reactive oxygen species
RT: – room temperature
SARs: – structure–activity relationships
SEM: – standard error of mean
SFC: – supercritical fluid chromatography
tBHP: – *tert*-butyl hydroperoxide;
THF: – tetrahydrofuran;
(sel-)TOCSY: – (selective) total correlation spectroscopy
UA: – uranyl acetate
UV–vis: – ultraviolet–visible
VLDL: – very-low-density lipoprotein.
