# Supplementary material for: Ecdysteroid-Containing Squalenoylated Self-Assembling Nanoparticles Exert Tumor-Selective Sensitization to Reactive Oxygen Species (ROS)-Induced Oxidative Damage While Protecting Normal Cells: Implications for Selective Radiotherapy
Source: J Med Chem. 2025 Mar 28;68(7):7197–212. doi: 10.1021/acs.jmedchem.4c02758 (PMC11997992; doi:10.1021/acs.jmedchem.4c02758)
Supplement: Supplementary file 1 — jm4c02758_si_001.pdf [file jm4c02758_si_001.pdf]

Supporting information

Ecdysteroid-containing squalenoylated self-assembling nanoparticles exert tumor-selective sensitization to reactive oxygen species (ROS)-induced oxidative damage while protecting normal cells: implications to selective radiotherapy

Máté Vágvölgyi,[†,◆] Endre Kocsis,[†,◆] Bizhar A. Tayeb,[‡,◆] István Zupkó,[‡] Renáta Minorics,[‡] Ana Martins,[§] Zsófia Hoyk,[§] Gergő Ballai,[‖] Imre Szenti,[‖] Zoltán Kónya,[‖,⊥] Tamás Gáti,[††] Dóra Bogdán,[#] Gábor Tóth,[¶] Attila Hunyadi*,[†,∇,○]

[†] Institute of Pharmacognosy, University of Szeged, Eötvös str. 6, H-6720 Szeged, Hungary

[‡] Institute of Pharmacodynamics and Biopharmacy, University of Szeged, Eötvös str. 6, H-6720 Szeged, Hungary

[§] Institute of Biophysics, Biological Research Centre, Hungarian Research Network, Temesvári blvd. 62, Szeged H-6726, Hungary

[‖] Department of Applied and Environmental Chemistry, Interdisciplinary Excellence Centre, University of Szeged, Rerrich Béla sq. 1, H-6720 Szeged, Hungary,

[⊥] HUN-REN-SZTE Reaction Kinetics and Surface Chemistry Research Group, University of Szeged, Rerrich Béla sq. 1, H-6720 Szeged, Hungary

[††] Servier Research Institute of Medicinal Chemistry (SRIMC), Záhony str. 7, H-1031 Budapest, Hungary

[#] Department of Organic Chemistry, Semmelweis University, Hőgyes Endre str. 7, H-1092 Budapest, Hungary,

[¶] NMR Group, Department of Inorganic and Analytical Chemistry, Budapest University of Technology and Economics, Szt. Gellért sq. 4, H-1111 Budapest, Hungary

[∇] HUN-REN-SZTE Biologically Active Natural Products Research Group, Eötvös str. 6, H-6720 Szeged, Hungary

[○] Graduate Institute of Natural Products, Shih-Chuan 1st Rd. 100, Kaohsiung 807, Taiwan

*Correspondence: hunyadi.attila@szte.hu; Tel.: +3662546456

Table of contents

S1. Compound **9** HRMS
S2. Compound **10** HRMS
S3. Compound **12** HRMS
S4. Compound **14** HRMS
S5. Compound **15** HRMS
S6. Compound **16** HRMS
S7. Compound **17** HRMS
S8. Compound **18** HRMS
S9. Compound **20** HRMS
S10. Compound **9** ^{1}H NMR
S11. Compound **9** ^{1}H NMR + selROE on d 1.38, 0.84 and 1.51 ppm
S12. Compound **9** ^{13}C DEPTQ
S13. Compound **9** edHSQC section
S14. Compound **9** HMBC
S15. Compound **10** ^{1}H NMR
S16. Compound **10** ^{1}H NMR + sel-Roesy (t_{mix}: 300 ms) on CH_3-18
S17. Compound **10** ^{13}C DEPTQ
S18. Compound **10** HSQC
S19. Compound **10** edHSQC CH_2 section
S20. Compound **10** HMBC
S21. Compound **12** ^{1}H NMR
S22. Compound **12** ^{1}H NMR + sel-Roesy (t_{mix}: 300 ms) on Ha-2, CH_3-19 and CH_3-18
S23. Compound **12** ^{13}C DEPTQ
S24. Compound **12** HSQC
S25. Compound **12** edHSQC CH_2 section
S26. Compound **12** HMBC
S27. Compound **14** ^{1}H NMR
S28. Compound **14** ^{13}C APT
S29. Compound **14** edHSQC CH+CH_3 sections
S30. Compound **14** edHSQC CH_2 section
S31. Compound **14** ROESY Me-section + HMBC Me-section
S32. Compound **15** ^{1}H NMR
S33. Compound **15** ^{13}C APT
S34. Compound **15** edHSQC
S35. Compound **15** HMBC
S36. Compound **15** ROESY
S37. Compound **16** ^{1}H NMR
S38. Compound **16** ^{1}H NMR section
S39. Compound **16** ^{1}H NMR + selTOCSY on (4.04/6.90/4.26) t_{mix}=120ms
S40. Compound **16** ^{13}C DEPTQ
S41. Compound **16** ^{13}C DEPTQ section
S42. Compound **16** HSQC

S43. Compound **16** HSQC section
S44. Compound **16** edHSQC CH_2 section
S45. Compound **16** HMBC
S46. Compound **16** HMBC section
S47. Compound **16** ^{13}C DEPTQ + selINEPT (d4.04t/2.30m/2.59t)
S48. Compound **16** ^{13}C DEPTQ + selINEPT (d4.04t/2.30t/2.59t) section
S49. Compound **17** ^{1}H NMR
S50. Compound **17** ^{1}H + selTOCSY 15, 12’ and 22
S51. Compound **17** ^{13}C NMR
S52. Compound **17** edHSQC + =CH section
S53. Compound **17** edHSQC CH_2 section
S54. Compound **17** sel. HSQC sections (33–43 and 32–22 ppm)
S55. Compound **17** edHSQC and HMBC CH_3 sections
S56. Compound **17** HMBC
S57. Compound **18** ^{1}H NMR
S58. Compound **18** ^{1}H NMR section 3.1–0.9 ppm
S59. Compound **18** ^{1}H NMR + selTOCSY at 4.05; 1.69 and 2.83 ppm
S60. Compound **18** ^{13}C DEPTQ
S61. Compound **18** edHSQC sections + band sel. HSQC
S62. Compound **18** HMBC + band sel. HMBC assignment of C-4’ and C-7’
S63. Compound **20** ^{1}H NMR
S64. Compound **20** ^{1}H NMR + selTOCSY on (4.05/1.69/2.55) t_{mix}=120ms
S65. Compound **20** ^{13}C NMR
S66. Compound **20** ^{13}C DEPTQ
S67. Compound **20** edHSQC + band sel. HSQC =CH section
S68. Compound **20** band sel. HSQC section 3.4-0.6/40.5-15 ppm
S69. Compound **20** HMBC
S70. Compound **20** band sel. HMBC 27.2–25.6 and 17.8–15.6 ppm sections
S71. Compound **20** band sel. HMBC 41.6–36.2 and 136–121 ppm sections
S72. Chromatographic conditions for the separation of semi-synthesized compounds
S73. Summarized DLS data of compounds **14–21**
S74. Particle size distribution plots of the nanosuspension of compound **14** (2 h)
S75. Particle size distribution plots of the nanosuspension of compound **14** (2 weeks)
S76. Particle size distribution plots of the nanosuspension of compound **14** (4 weeks)
S77. Particle size distribution plots of the nanosuspension of compound **14** (16 weeks)
S78. Particle size distribution plots of the nanosuspension of compound **15** (2 h)
S79. Particle size distribution plots of the nanosuspension of compound **15** (2 weeks)
S80. Particle size distribution plots of the nanosuspension of compound **15** (4 weeks)
S81. Particle size distribution plots of the nanosuspension of compound **15** (16 weeks)
S82. Particle size distribution plots of the nanosuspension of compound **16** (2 h)
S83. Particle size distribution plots of the nanosuspension of compound **16** (2 weeks)
S84. Particle size distribution plots of the nanosuspension of compound **16** (4 weeks)
S85. Particle size distribution plots of the nanosuspension of compound **16** (16 weeks)
S86. Particle size distribution plots of the nanosuspension of compound **17** (2 h)

S87. Particle size distribution plots of the nanosuspension of compound **17** (10 weeks)
S88. Particle size distribution plots of the nanosuspension of compound **18** (2 h)
S89. Particle size distribution plots of the nanosuspension of compound **18** (10 weeks)
S90. Particle size distribution plots of the nanosuspension of compound **19** (2 h)
S91. Particle size distribution plots of the nanosuspension of compound **19** (10 weeks)
S92. Particle size distribution plots of the nanosuspension of compound **20** (2 h)
S93. Particle size distribution plots of the nanosuspension of compound **20** (10 weeks)
S94. Particle size distribution plots of the nanosuspension of compound **21** (2 h)
S95. Particle size distribution plots of the nanosuspension of compound **21** (10 weeks)
S96. Antiproliferative effect of compounds on SH-SY5Y and MRC-5 cells at 0.5 µM
S97. Antiproliferative effect of vincristine on SH-SY5Y and MRC-5 cells
S98. Dose-response curve of tBHP treatment on SH-SY5Y cells after 4h
S99. Effect of compounds at 0.5–10 µM on the IC_{50} value of tBHP on SH-SY5Y cells
S100. Calculated IC_{50} values and selectivity indices (SI) of tBHP on SH-SY5Y and MRC-5 cell
S101. TEM images of the nanoassemblies of compound **18**.
S102. RP-HPLC-PDA max plot chromatogram of ecdysteroid lead compound **9**.
S103. SFC-PDA max plot chromatogram of ecdysteroid lead compound **16**.

S1. Compound **9** HRMS

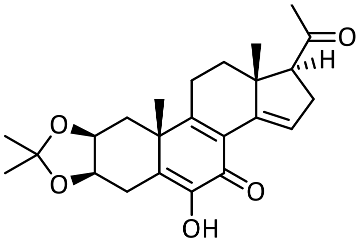

Chemical Formula: $C_{24}H_{30}O_5$

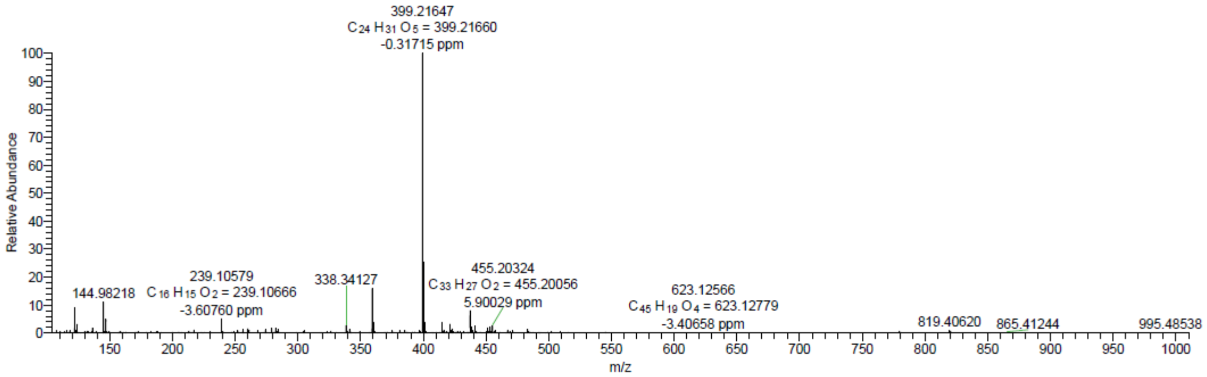

S2. Compound **10** HRMS

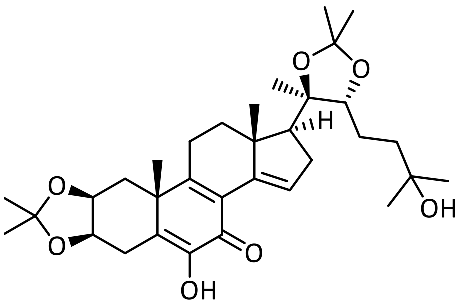

Chemical Formula: $C_{33}H_{48}O_7$

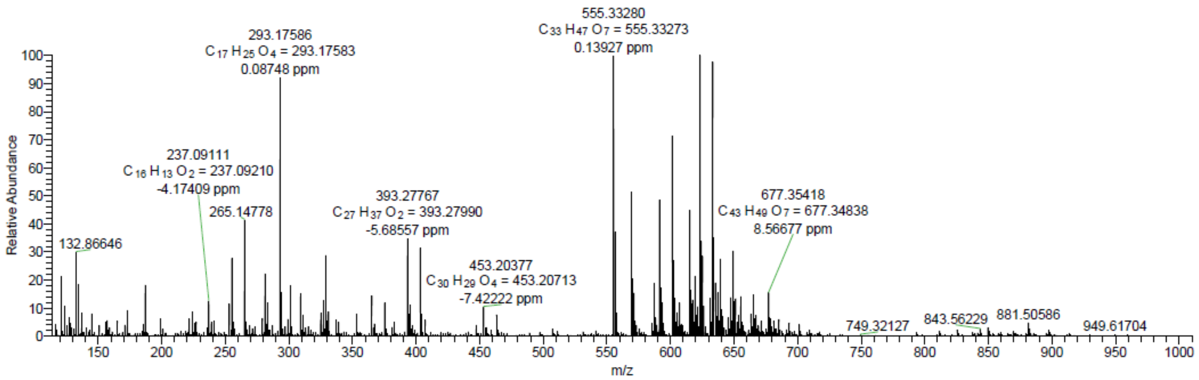

S3. Compound **12** HRMS

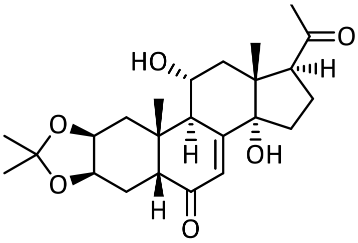

Chemical Formula: $C_{24}H_{34}O_6$

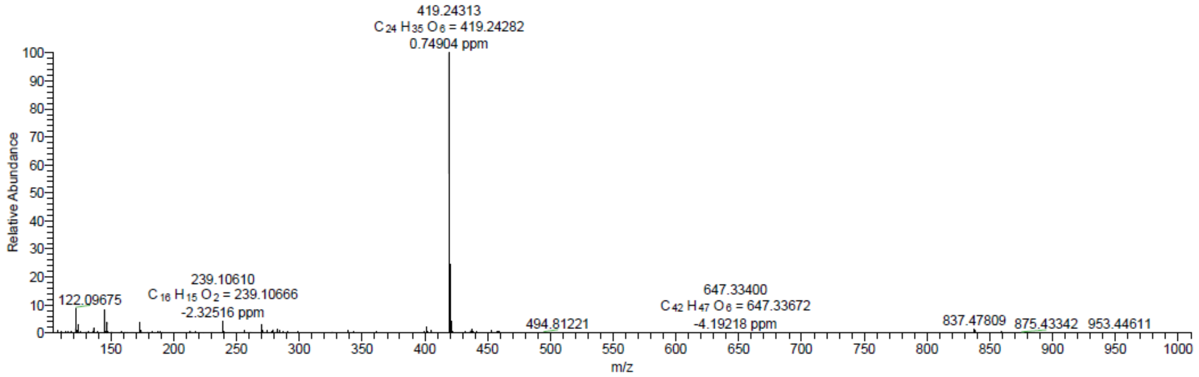

S4. Compound **14** HRMS

Chemical Formula: $C_{64}H_{100}O_{10}$

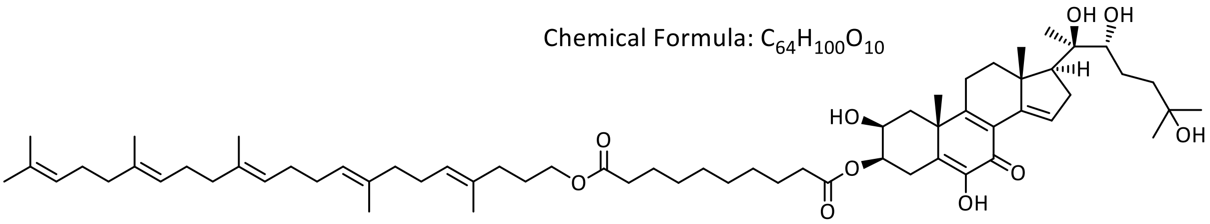

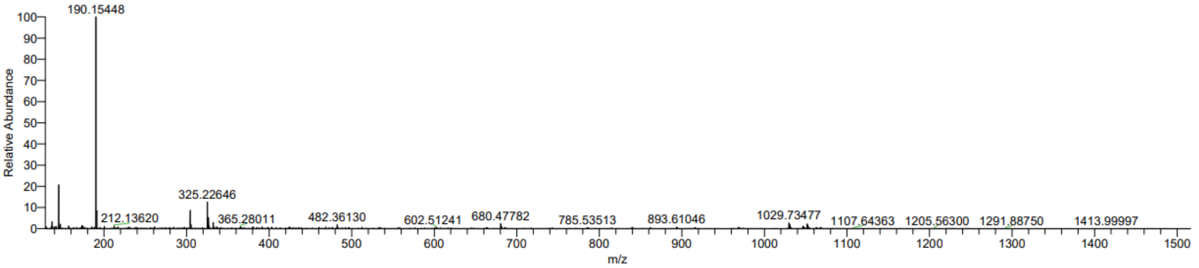

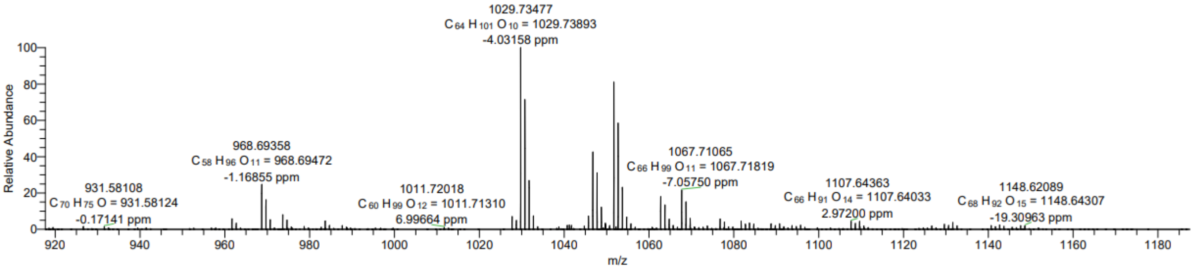

S5. Compound **15** HRMS

Chemical Formula: $C_{58}H_{86}O_8$

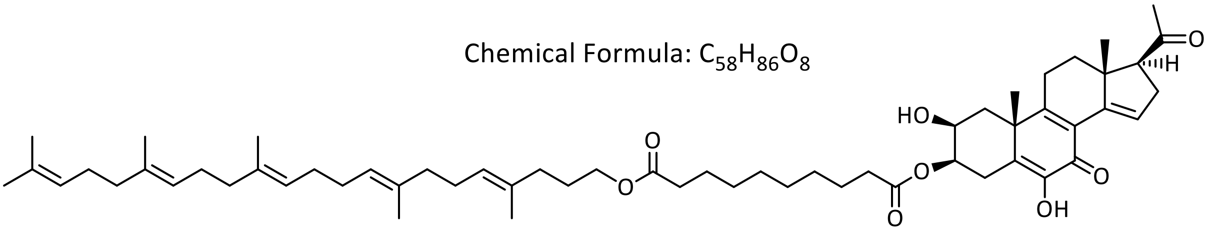

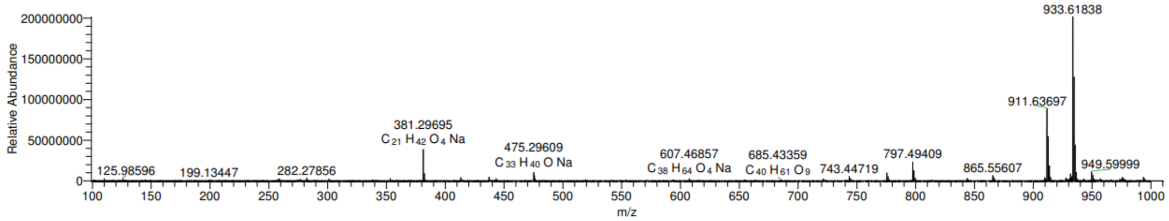

S6. Compound **16** HRMS

Chemical Formula: $C_{61}H_{90}O_8$

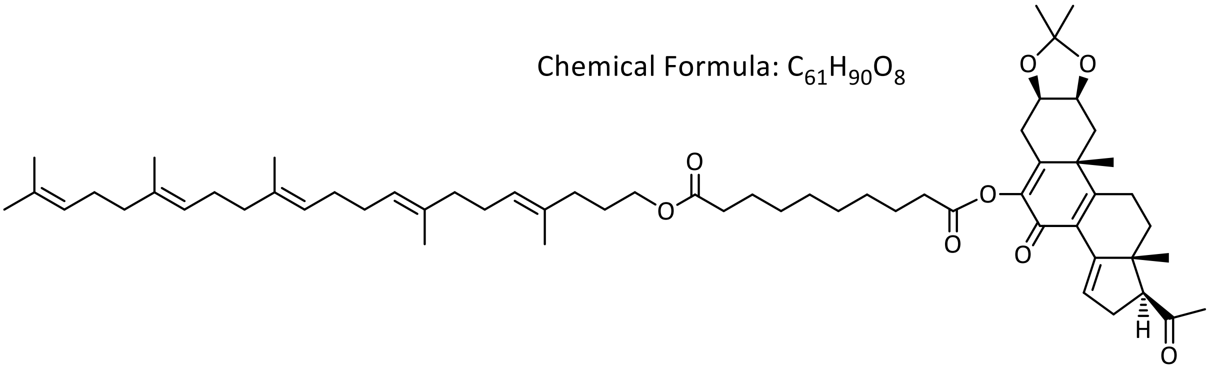

data15 #5 RT: 0.06 AV: 1 NL: 6.62E7
T: FTMS + p ESI Full ms [100.0000-1500.0000]

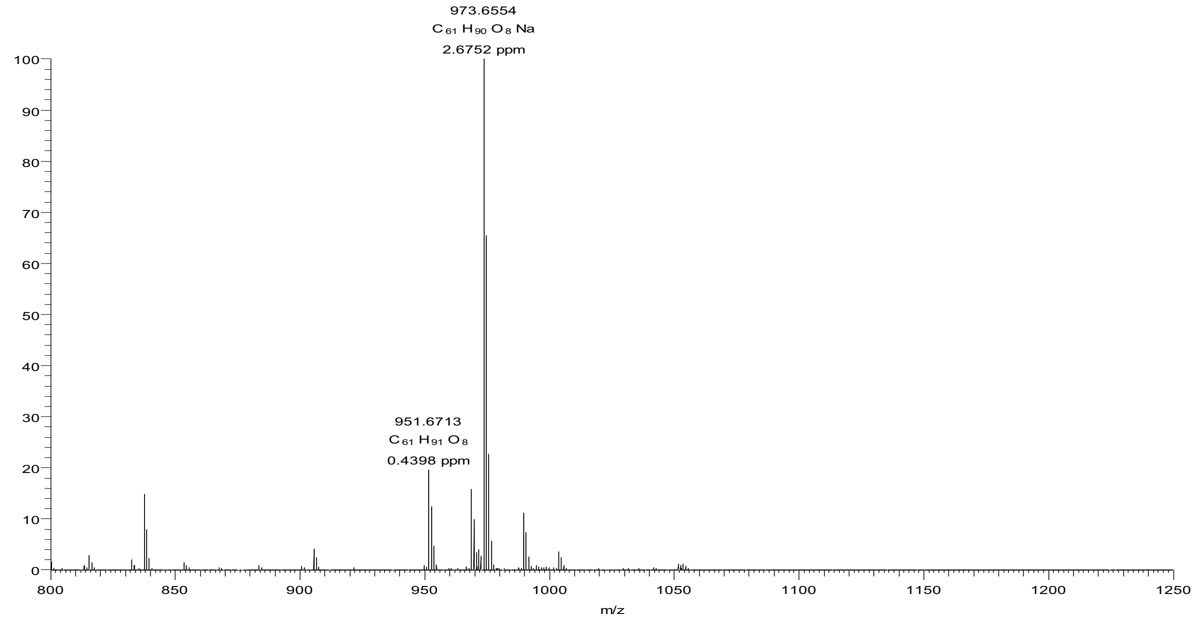

S7. Compound **17** HRMS

Chemical Formula: $C_{70}H_{108}O_{10}$

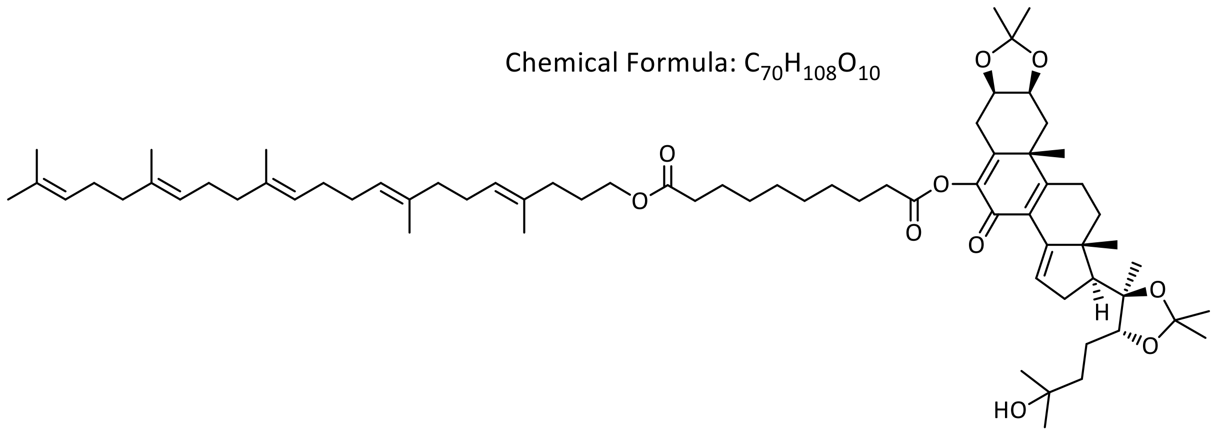

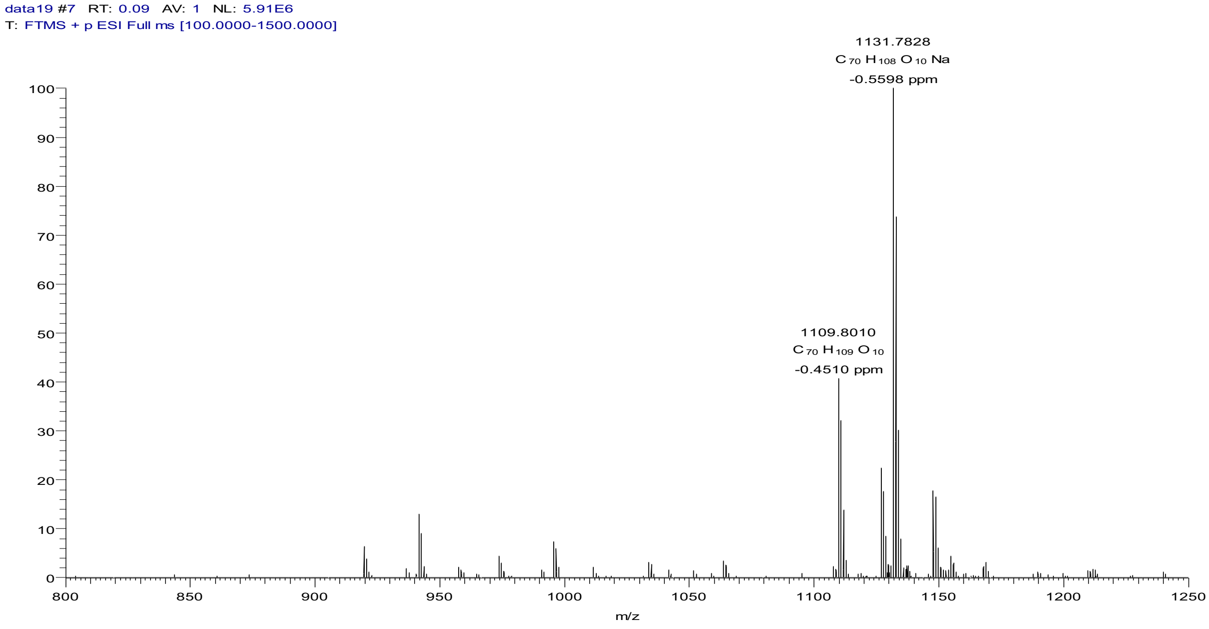

S8. Compound **18** HRMS

Chemical Formula: $C_{68}H_{104}O_{10}S_2$

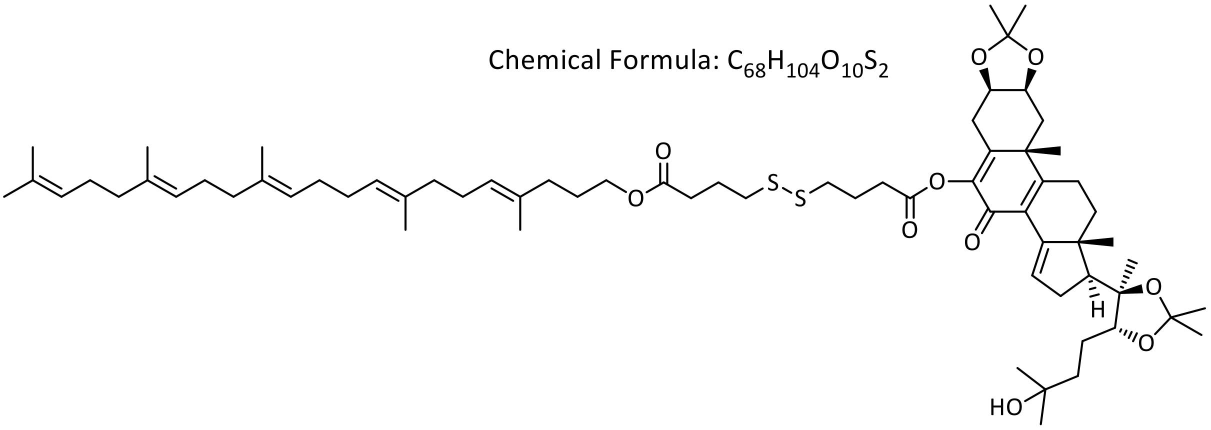

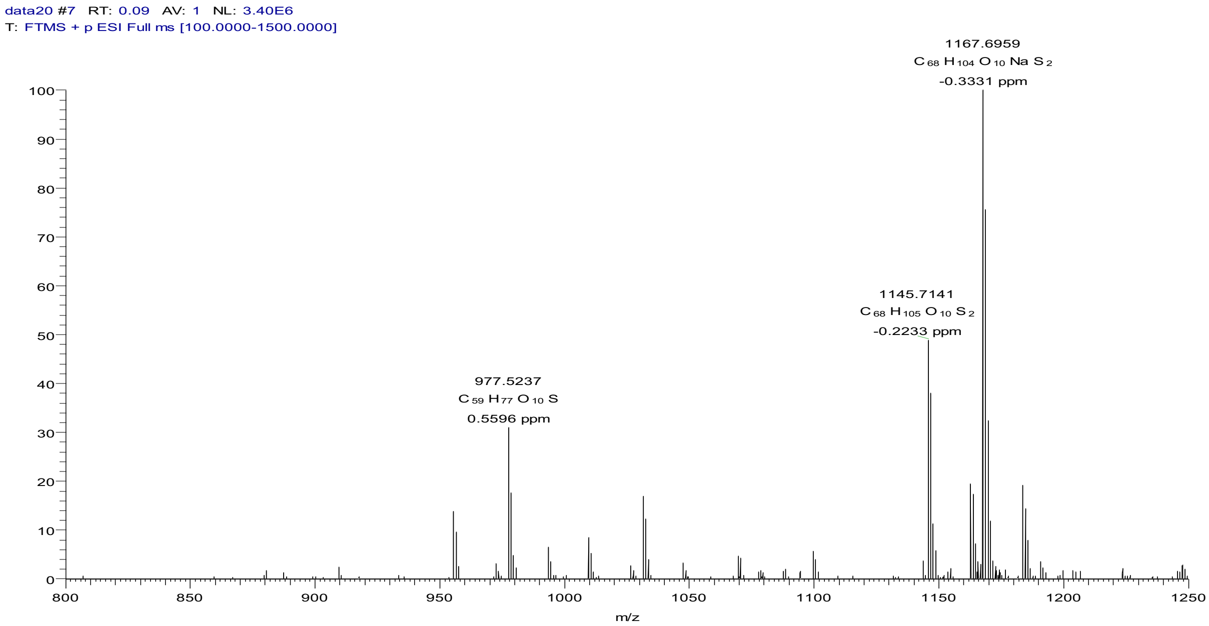

S9. Compound **20** HRMS

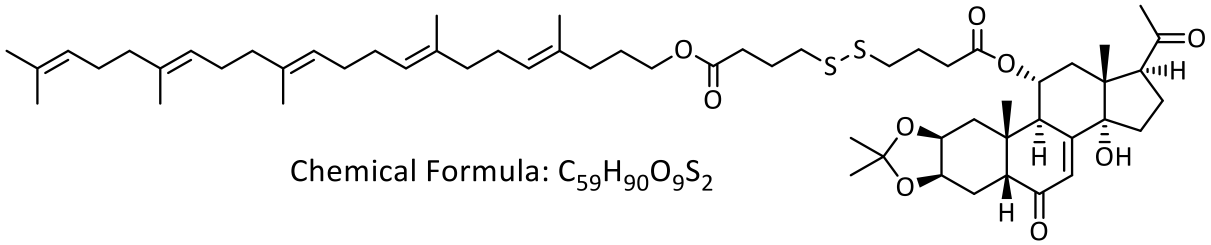

Chemical Formula: $C_{59}H_{90}O_9S_2$

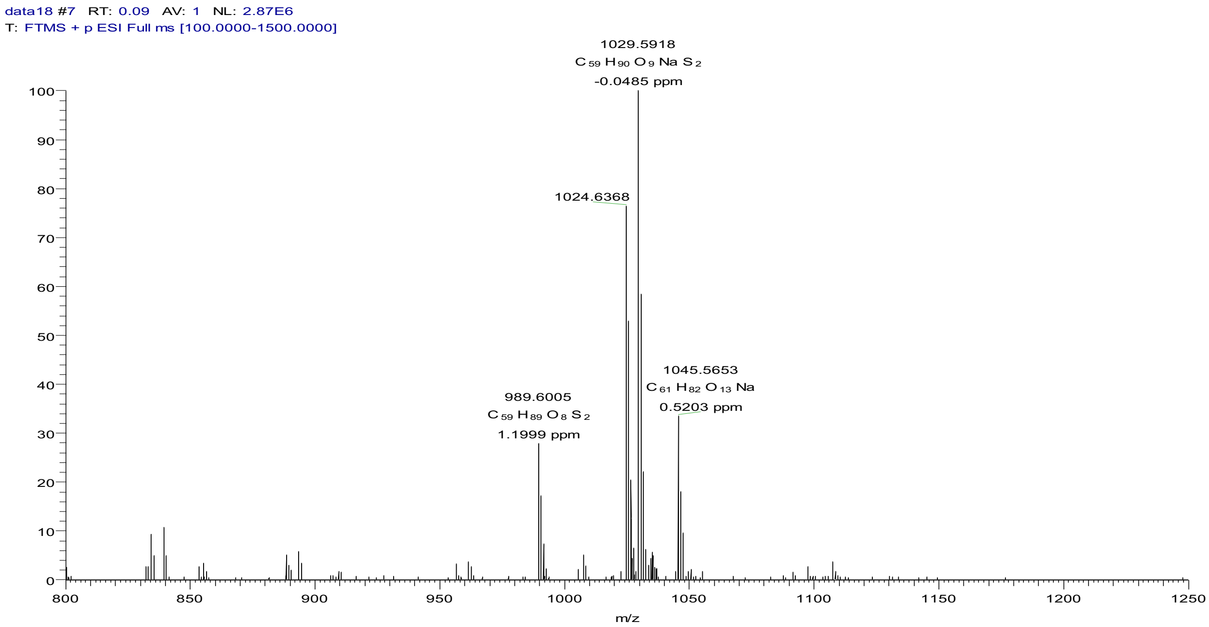

S10. Compound **9** ^{1}H NMR

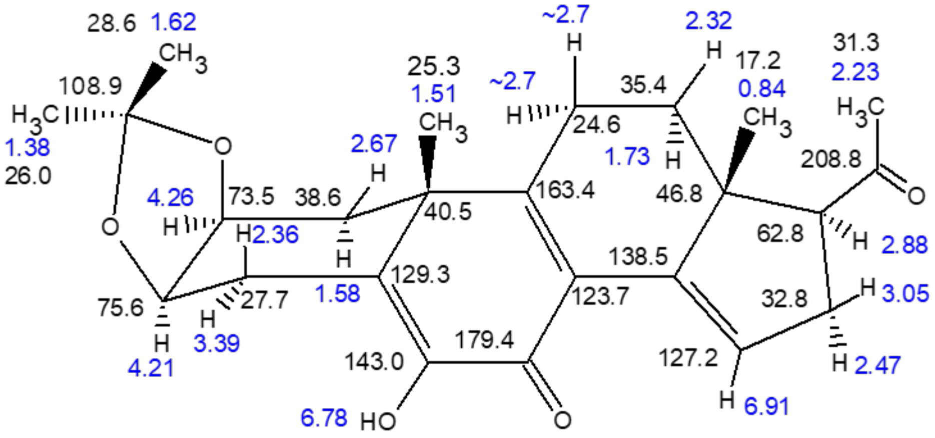

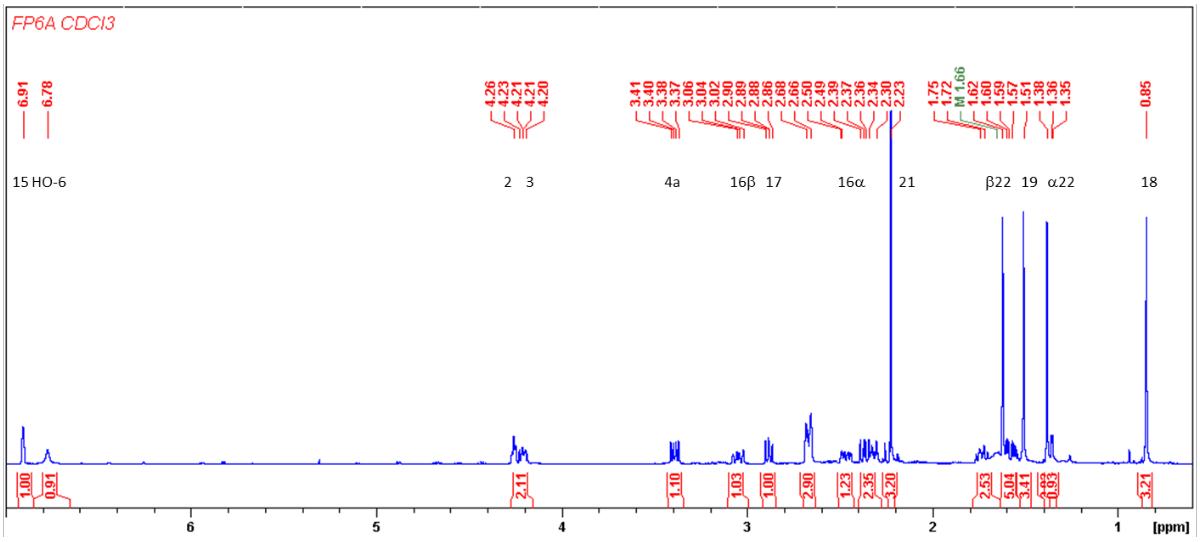

S11. Compound **9** ^{1}H NMR + selROE on d 1.38, 0.84 and 1.51 ppm

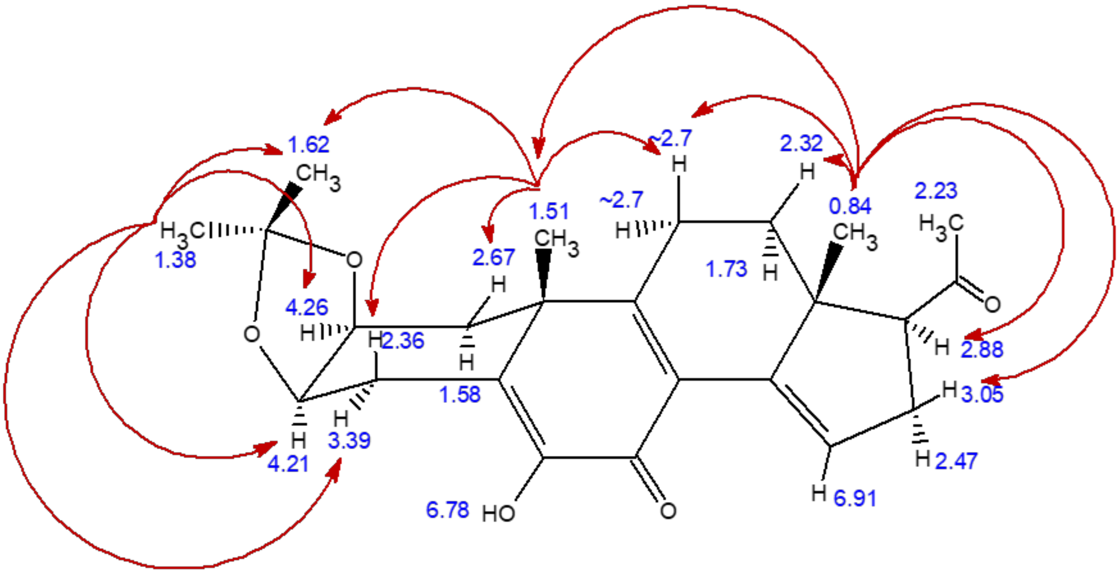

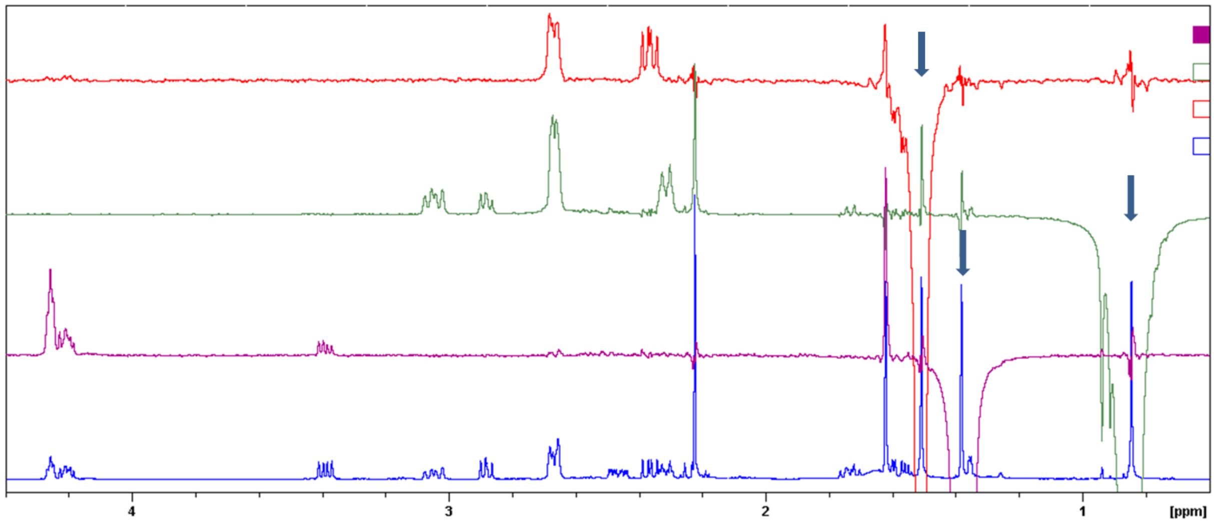

S12. Compound **9** ^{13}C DEPTQ

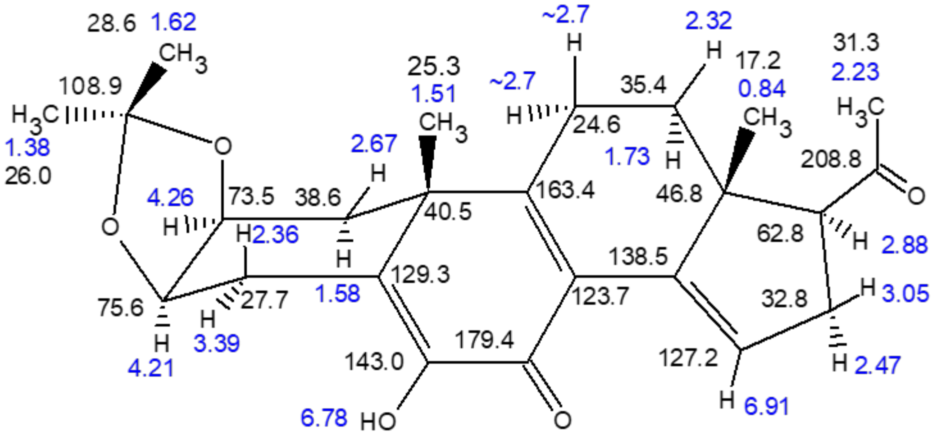

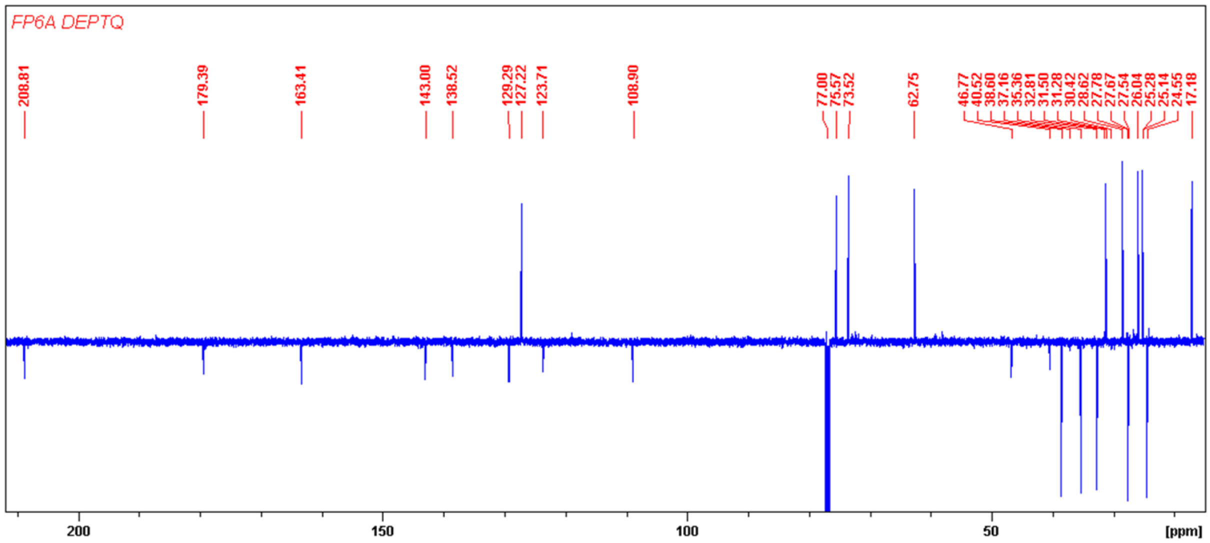

S13. Compound **9** edHSQC section

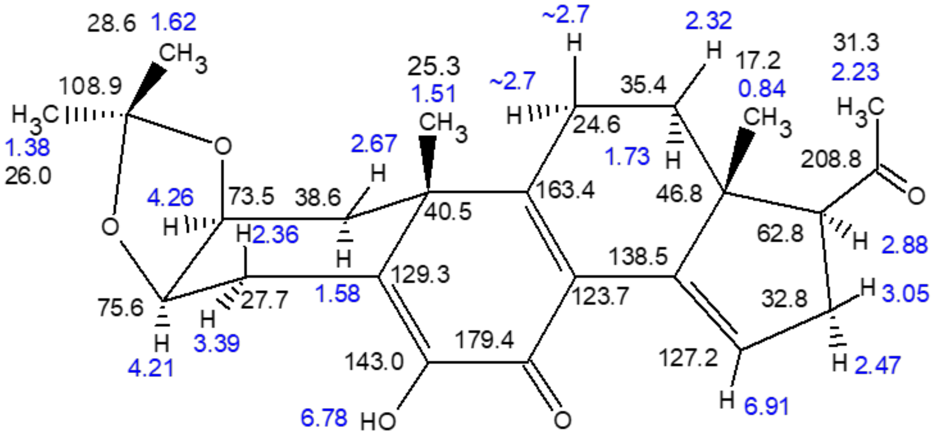

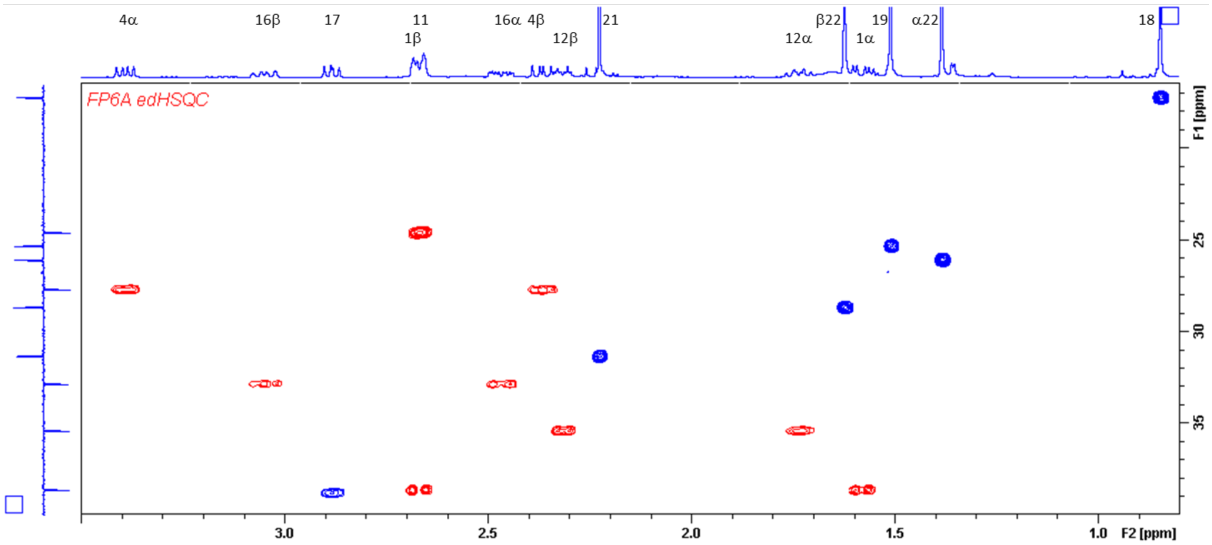

S14. Compound **9** HMBC

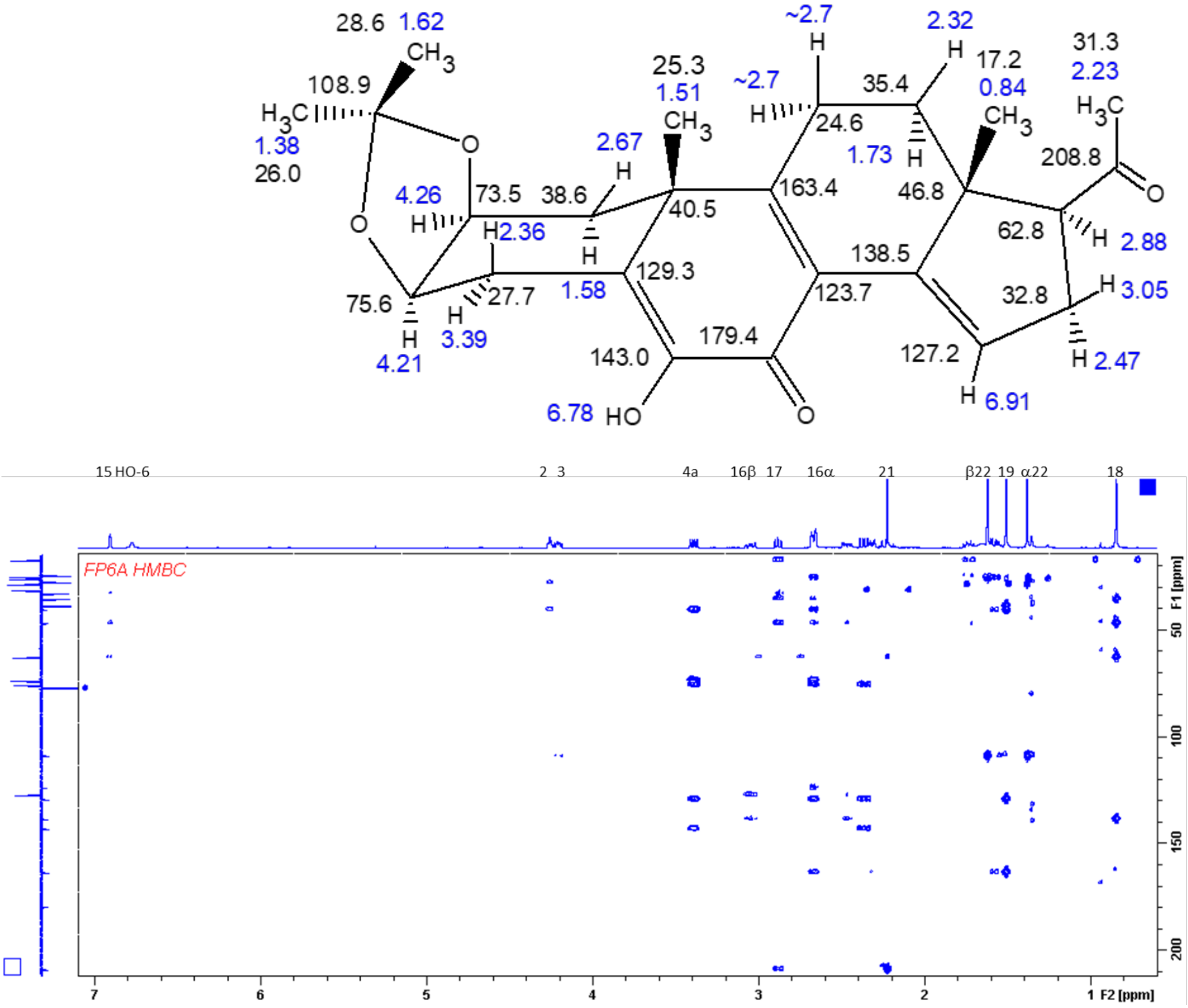

S15. Compound **10** ^{1}H NMR

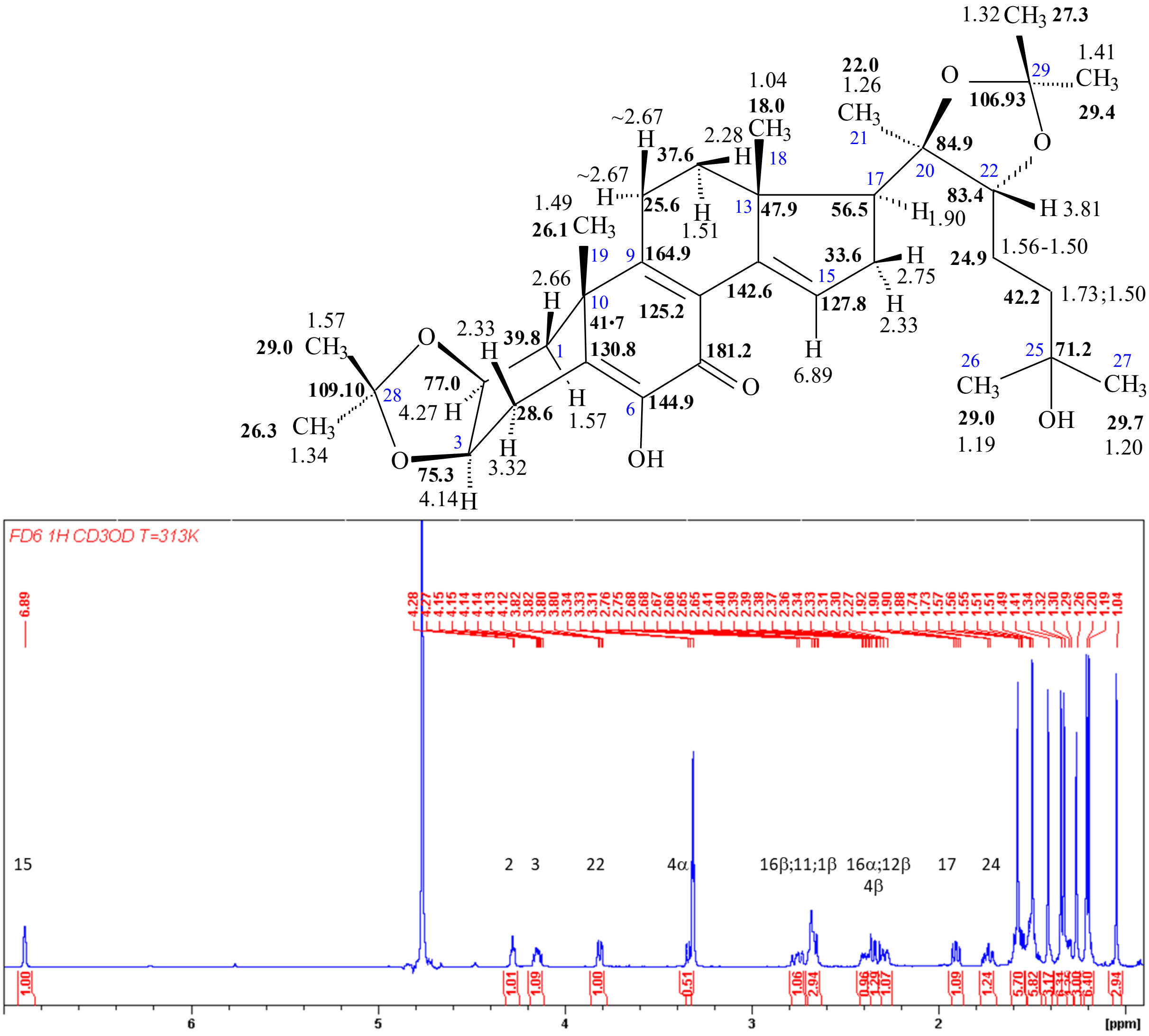

S16. Compound **10** ^{1}H NMR + sel-Roesy (t_{mix}: 300 ms) on CH_3-18

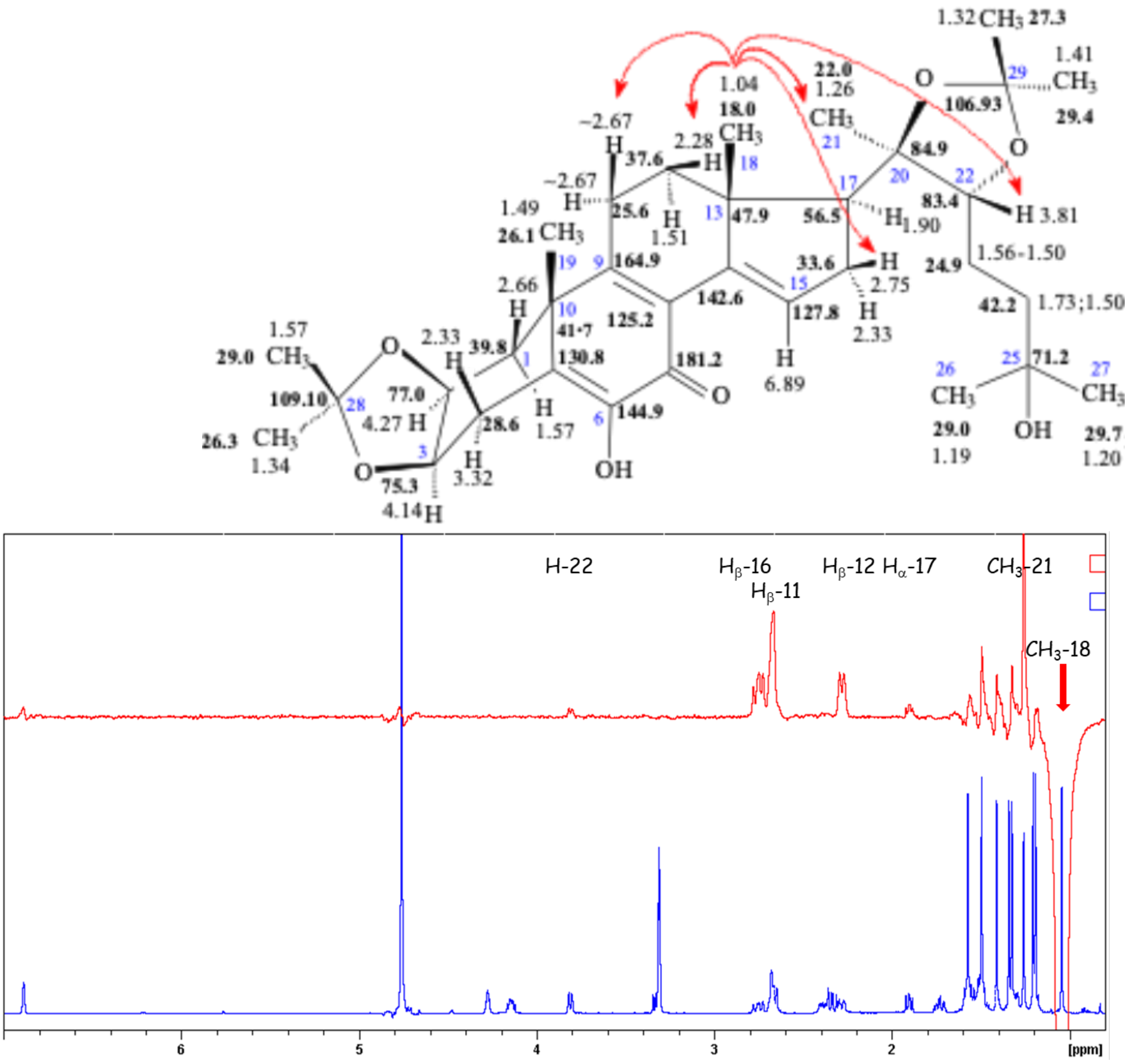

S17. Compound **10** ^{13}C DEPTQ

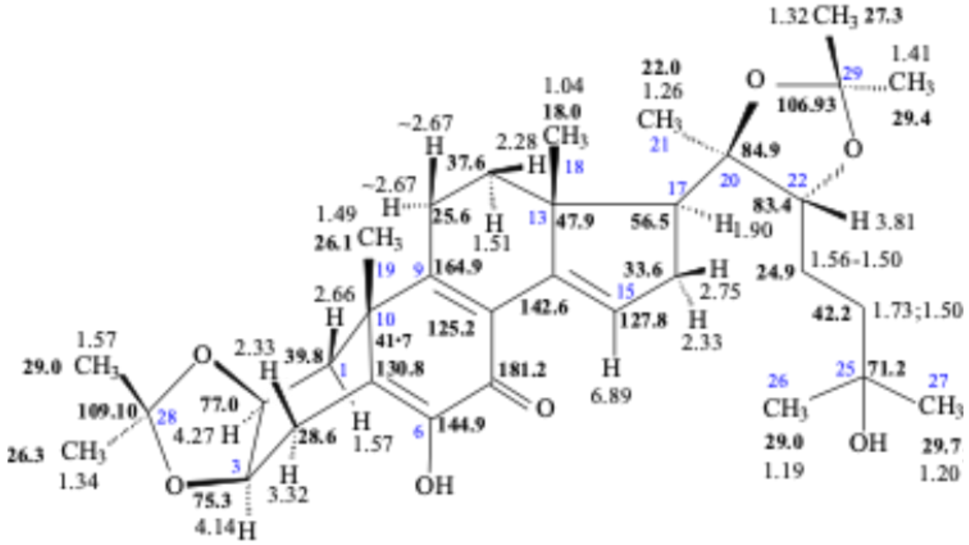

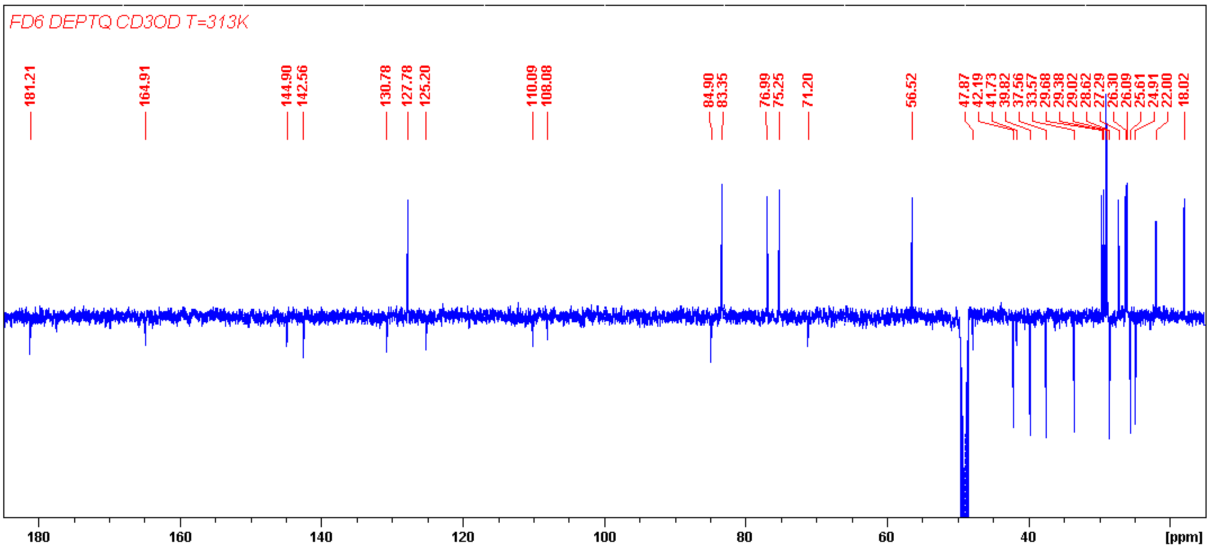

S18. Compound **10** HSQC

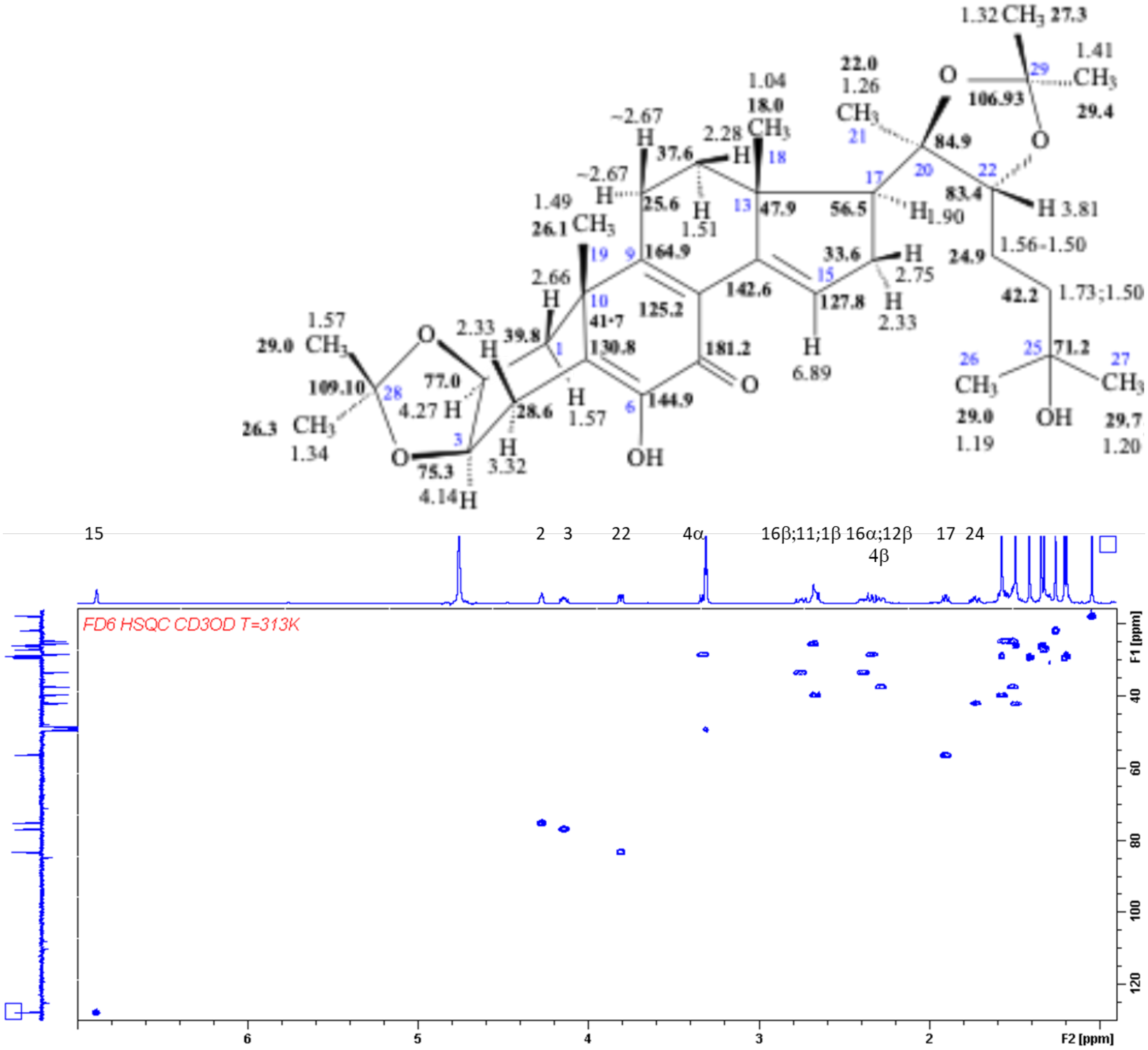

S19. Compound **10** edHSQC CH_2 section

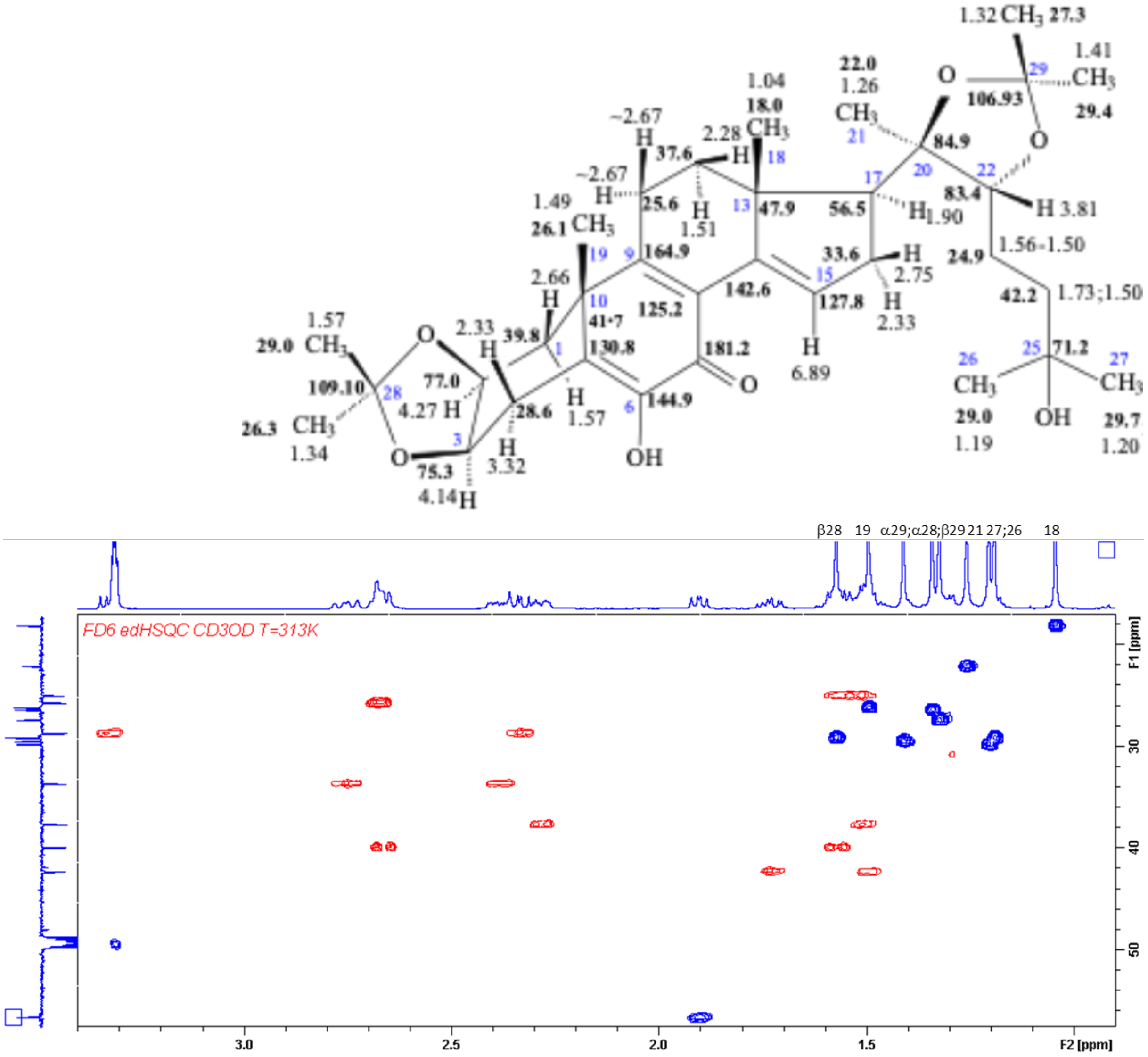

S20. Compound **10** HMBC

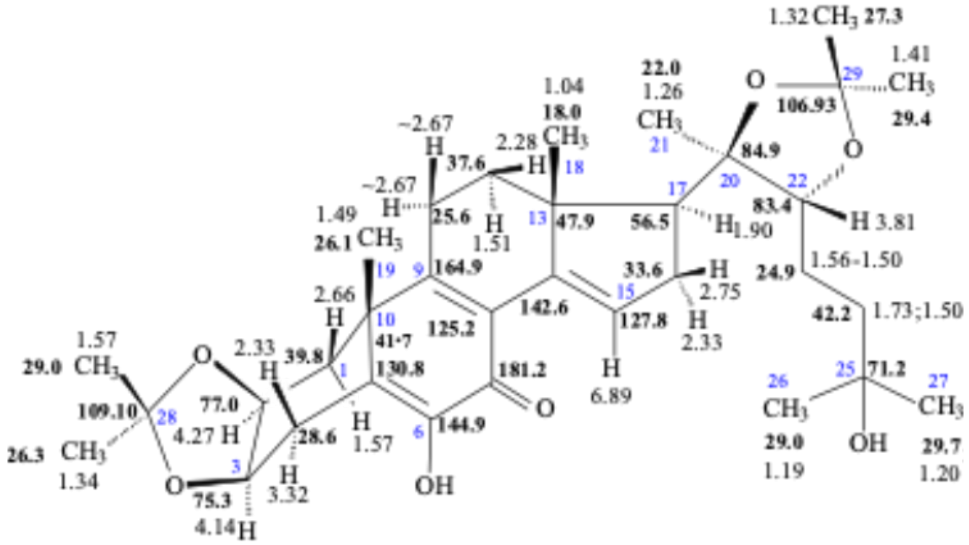

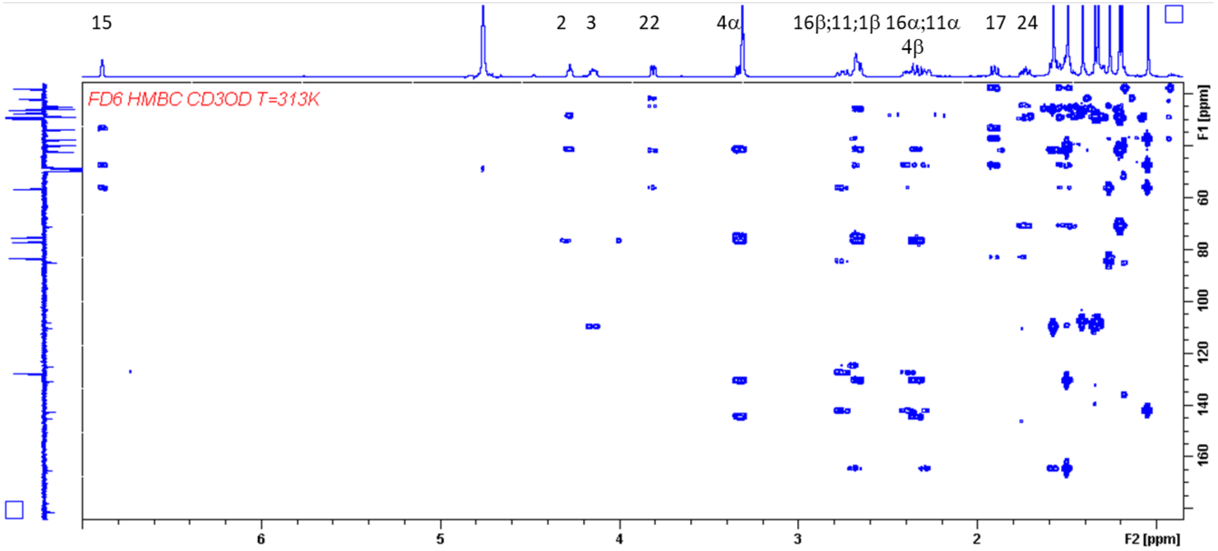

S21. Compound **12** ^{1}H NMR

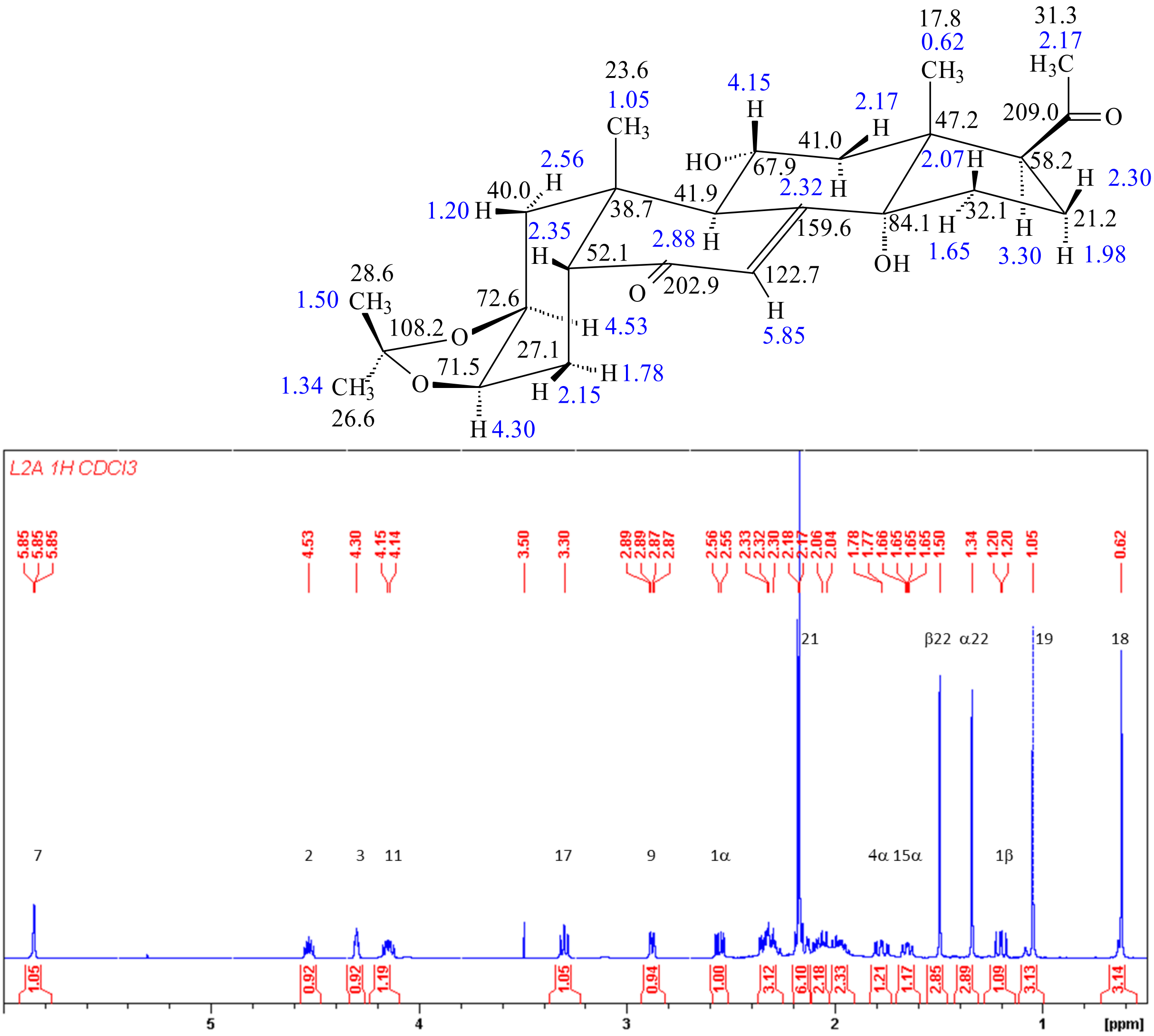

S22. Compound **12** ^{1}H NMR + sel-Roesy (t_{mix}: 300 ms) on Ha-2, CH_3-19 and CH_3-18

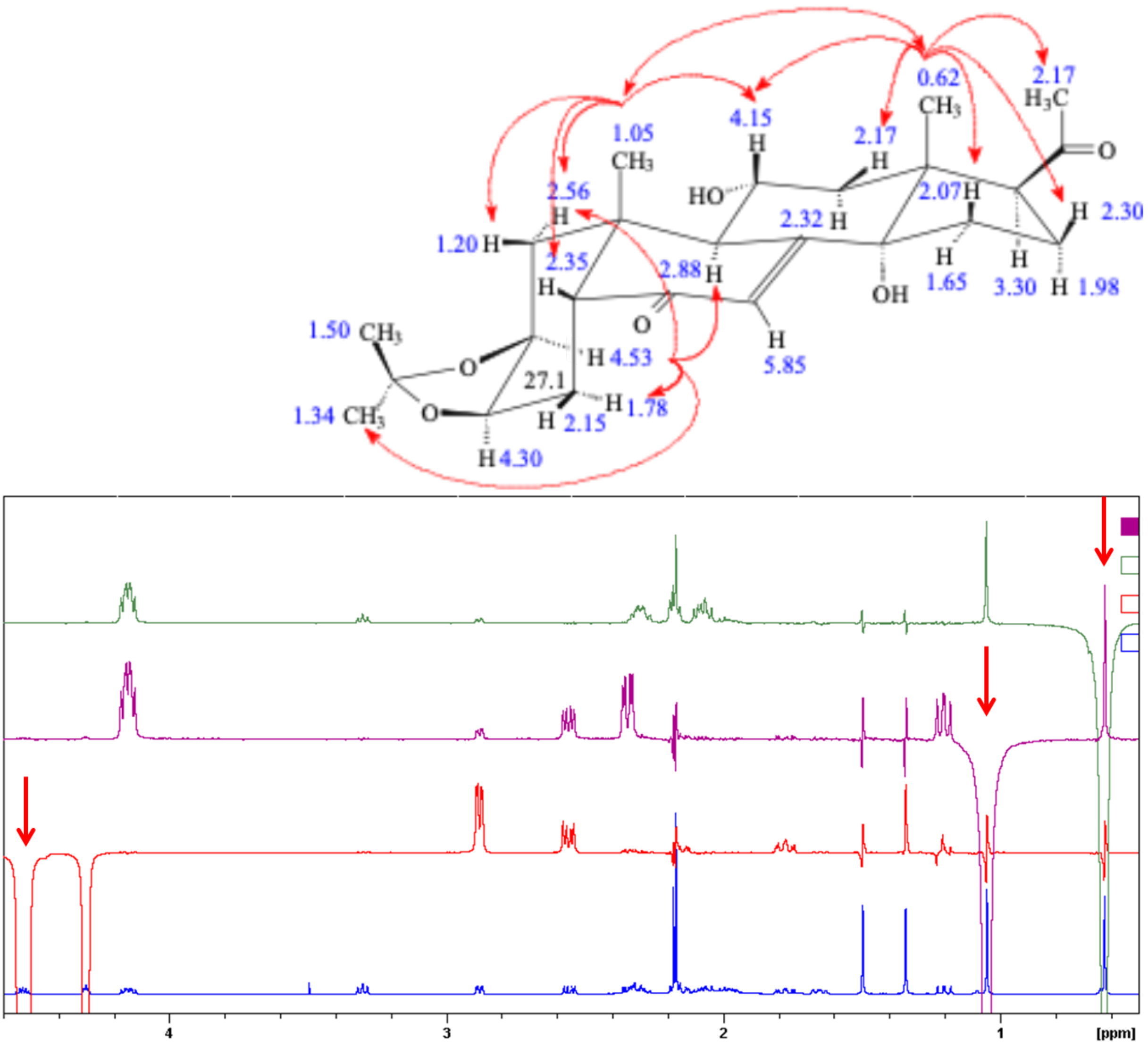

S23. Compound **12** ^{13}C DEPTQ

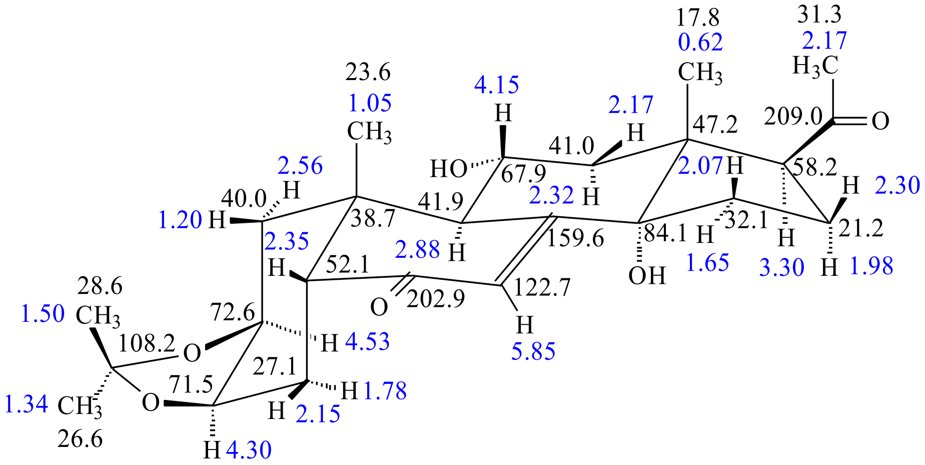

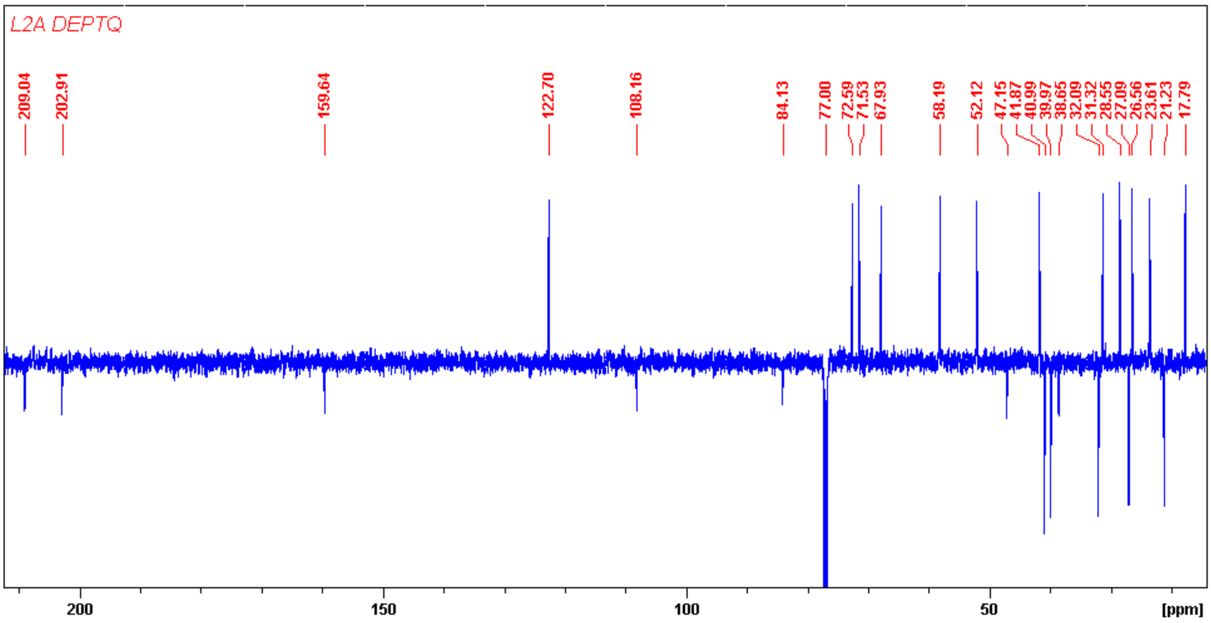

S24. Compound **12** HSQC

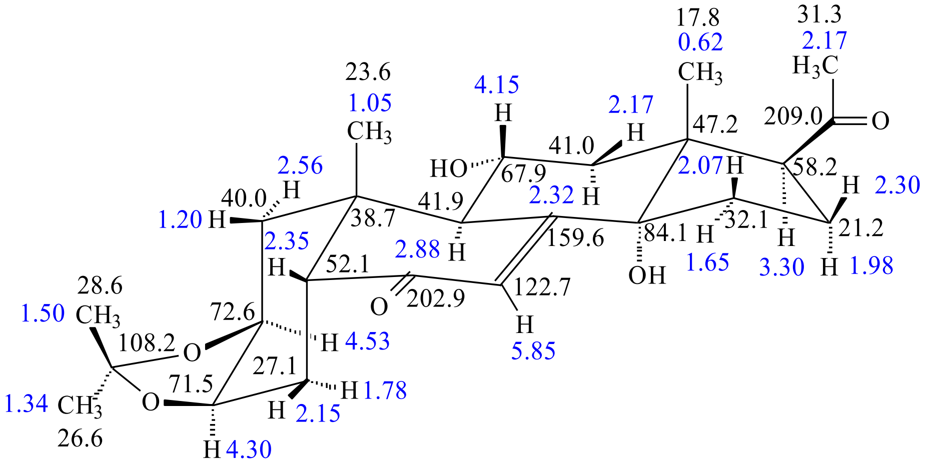

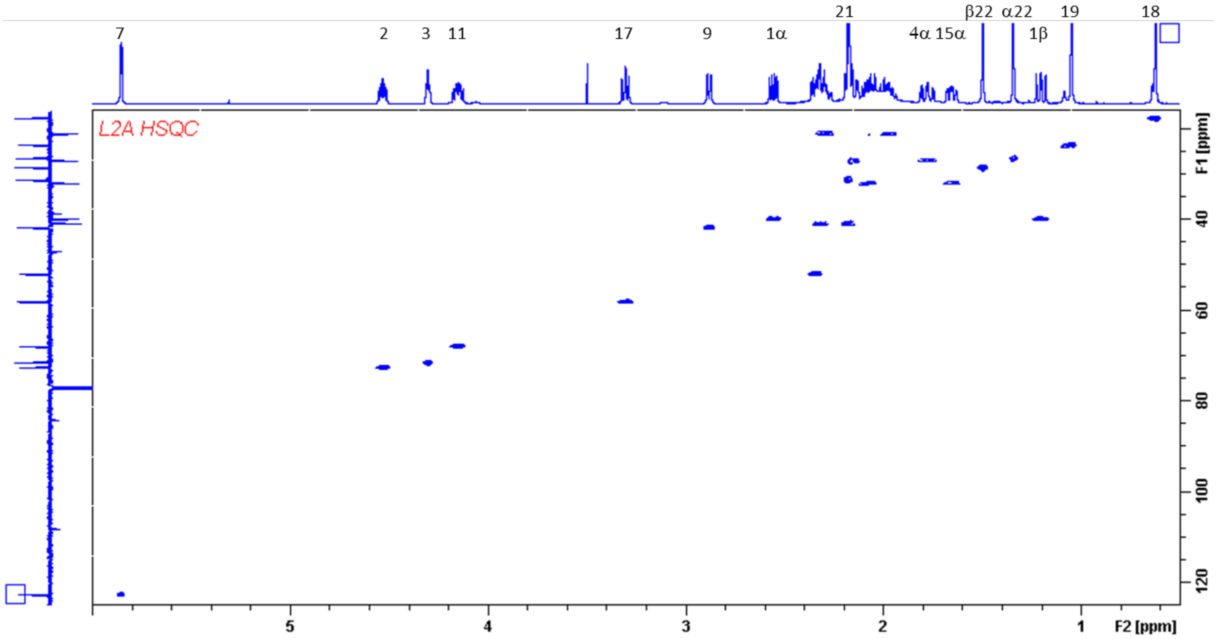

S25. Compound **12** edHSQC CH$_2$ section

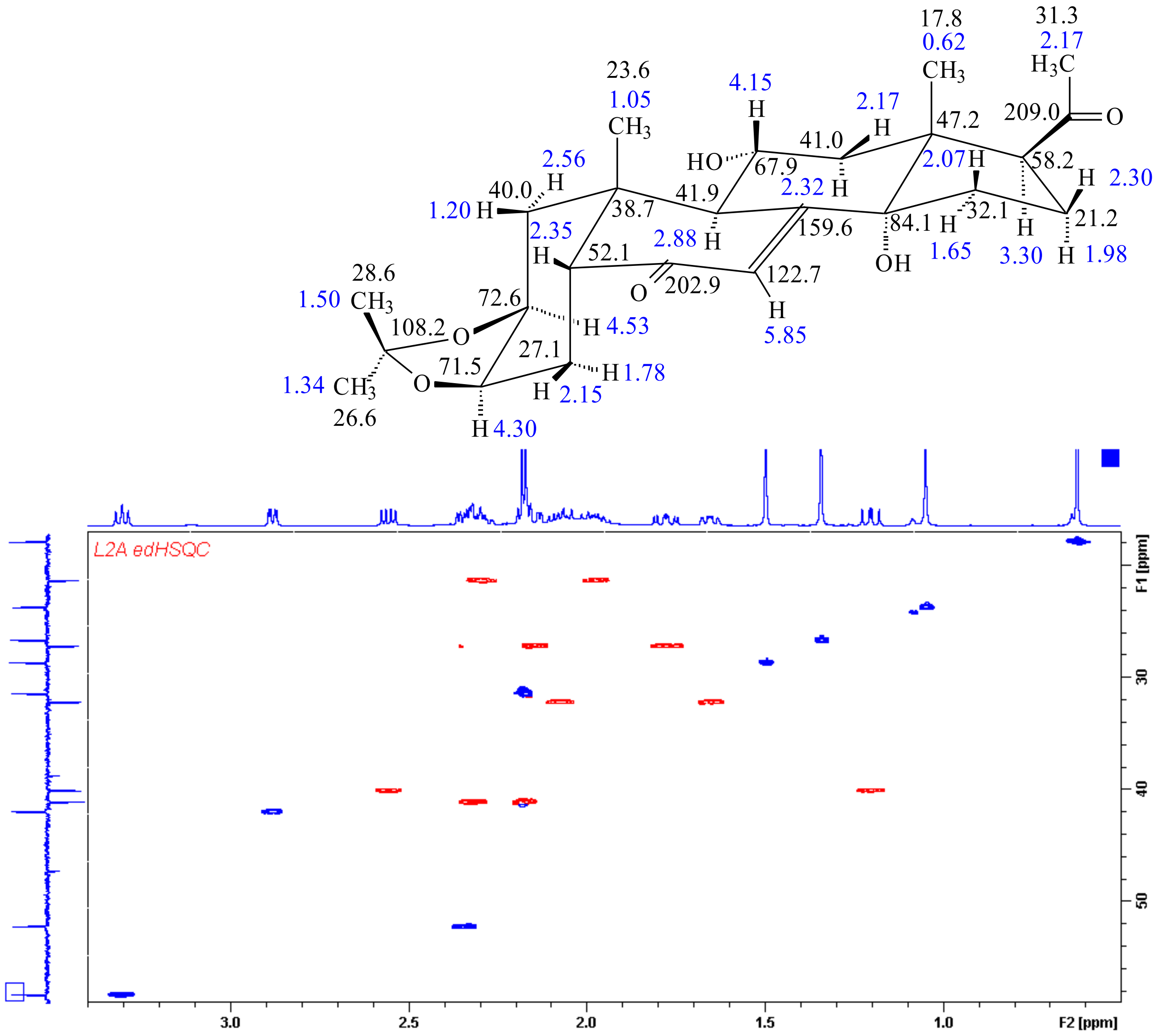

S26. Compound **12** HMBC

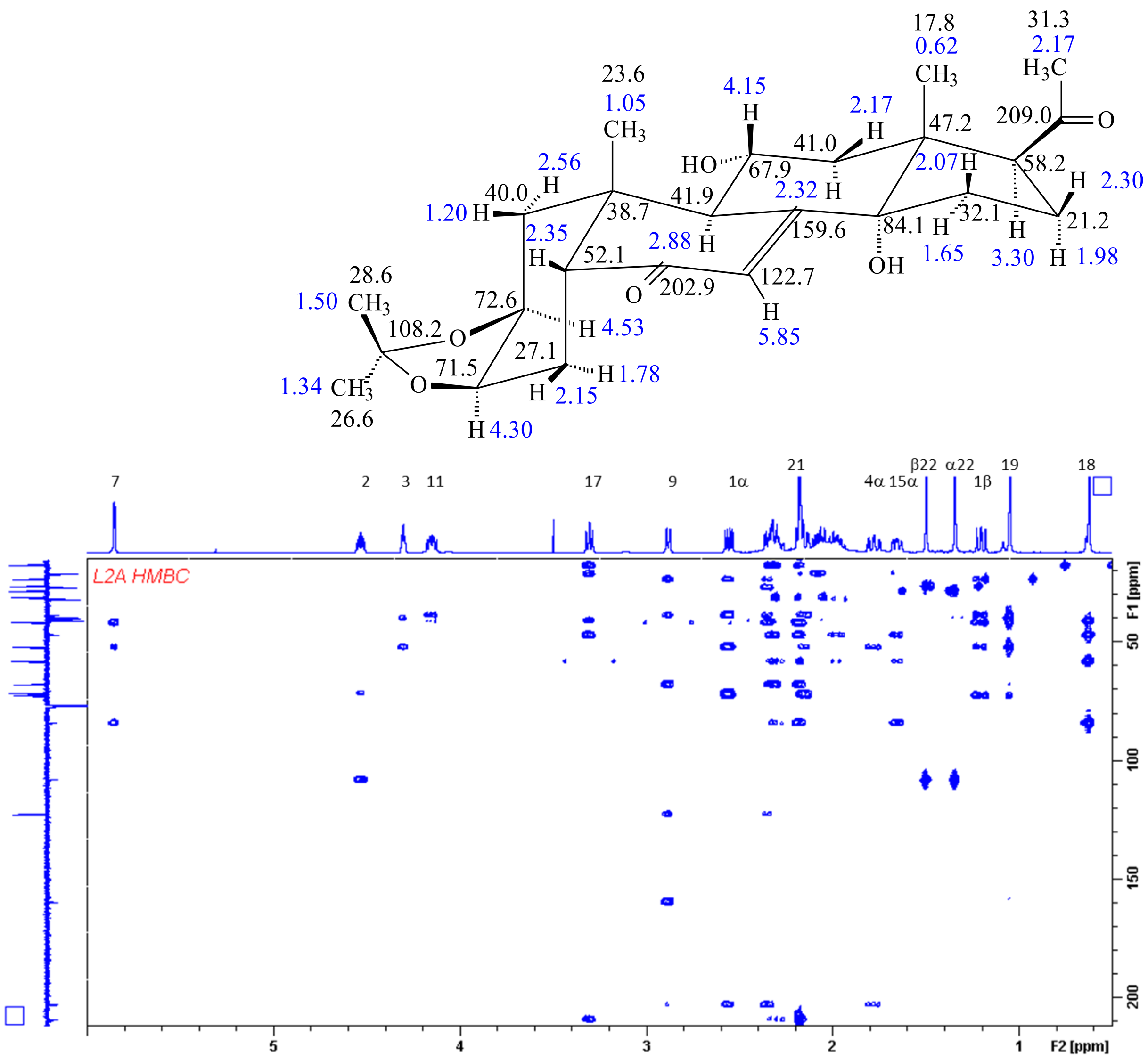

S27. Compound **14** ^{1}H NMR

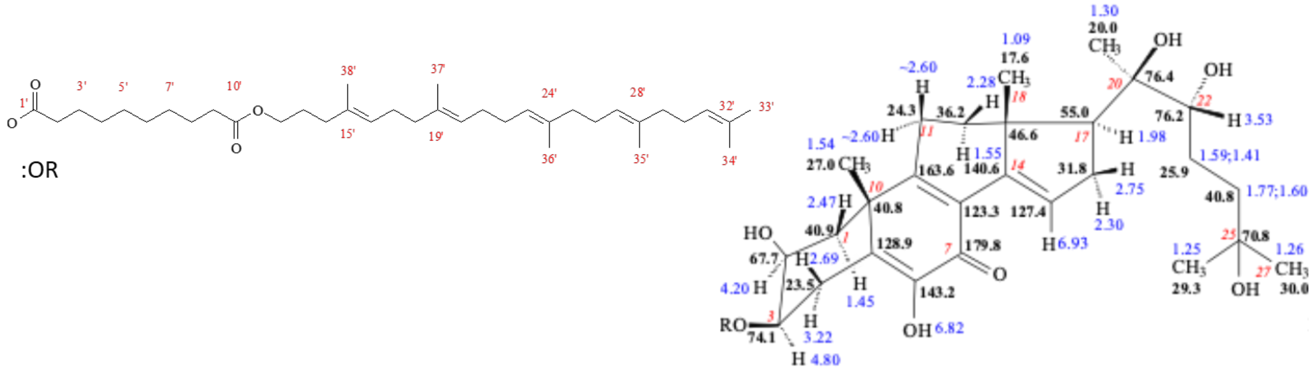

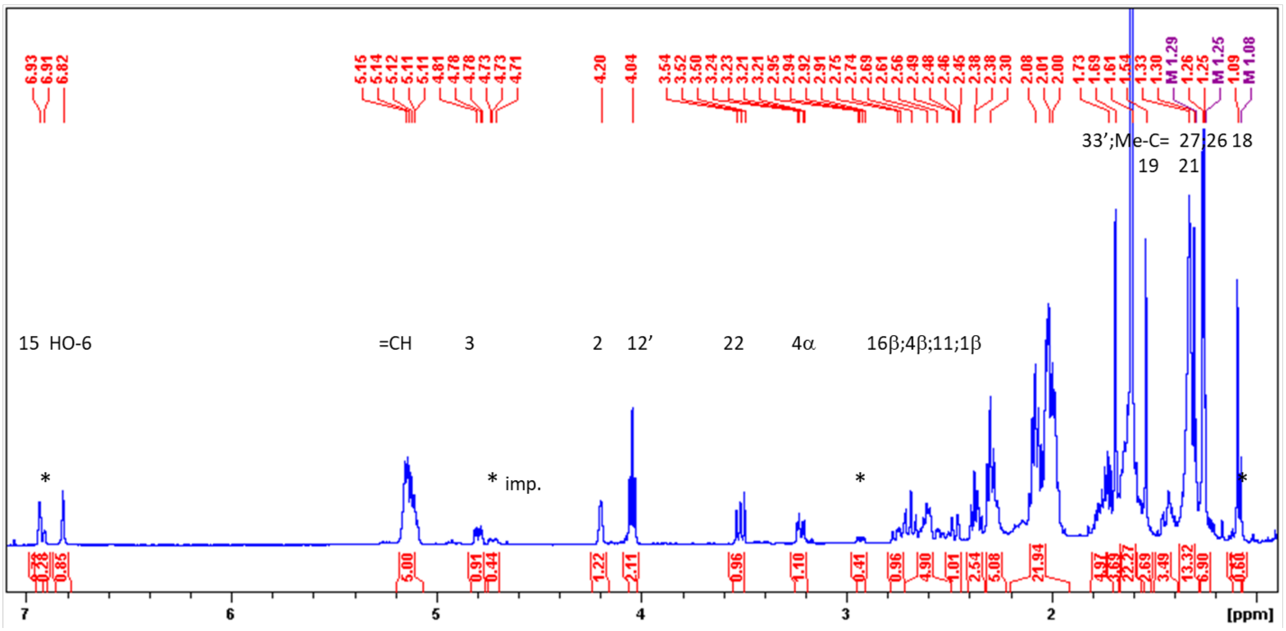

S28. Compound **14** ^{13}C APT

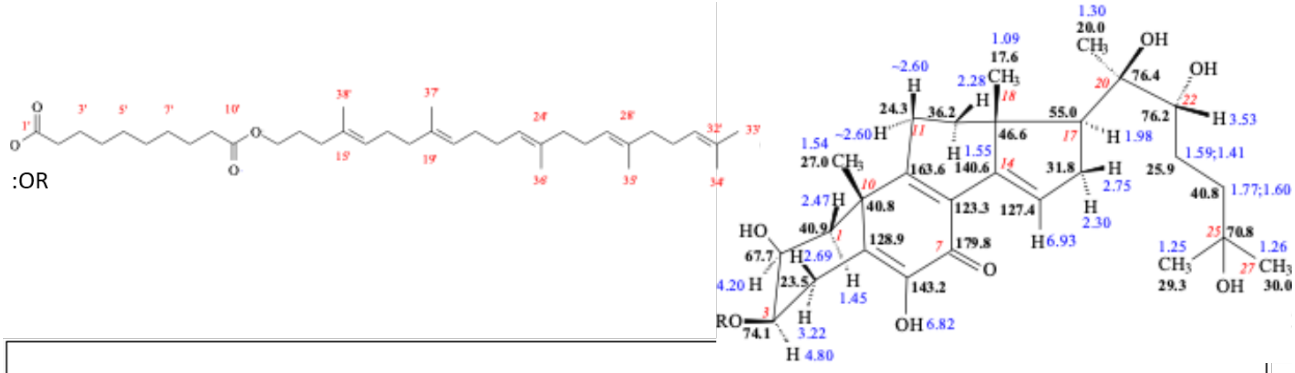

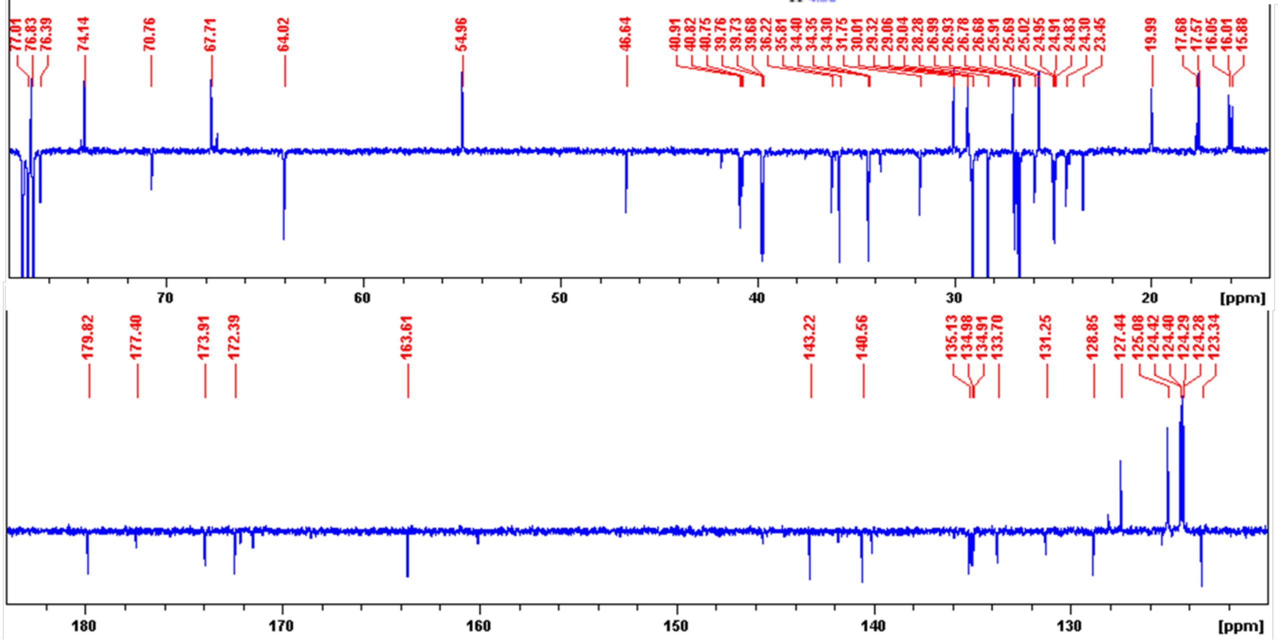

S29. Compound **14** edHSQC CH+CH_3 sections

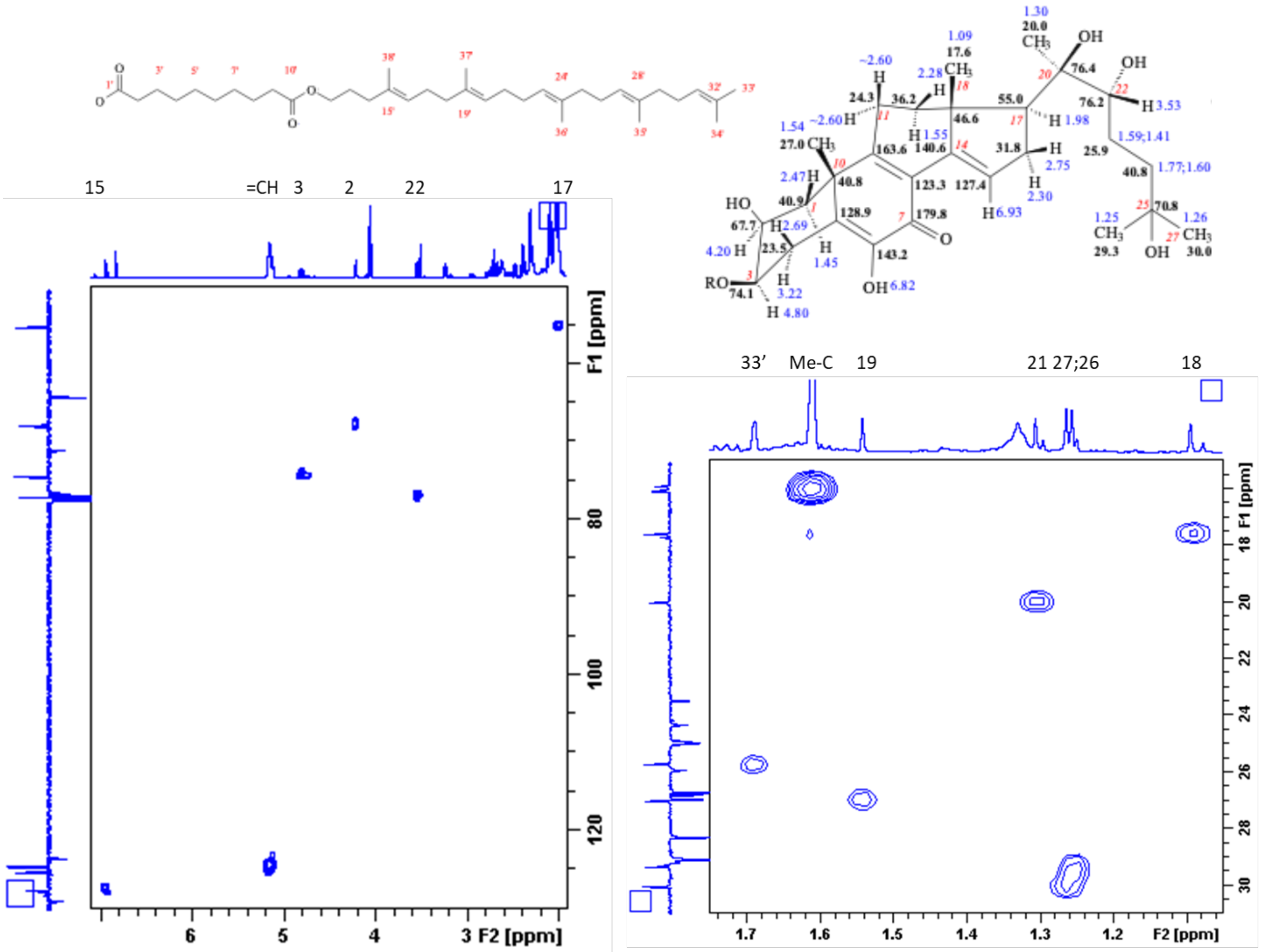

S30. Compound **14** edHSQC CH_2 section

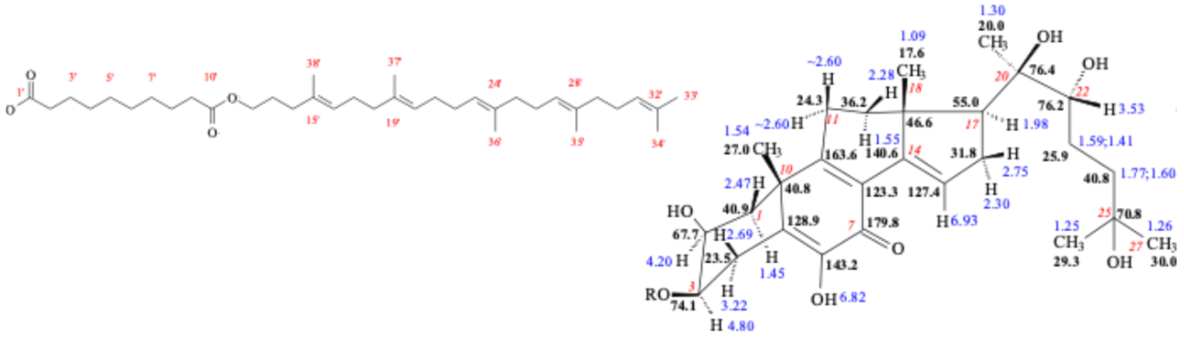

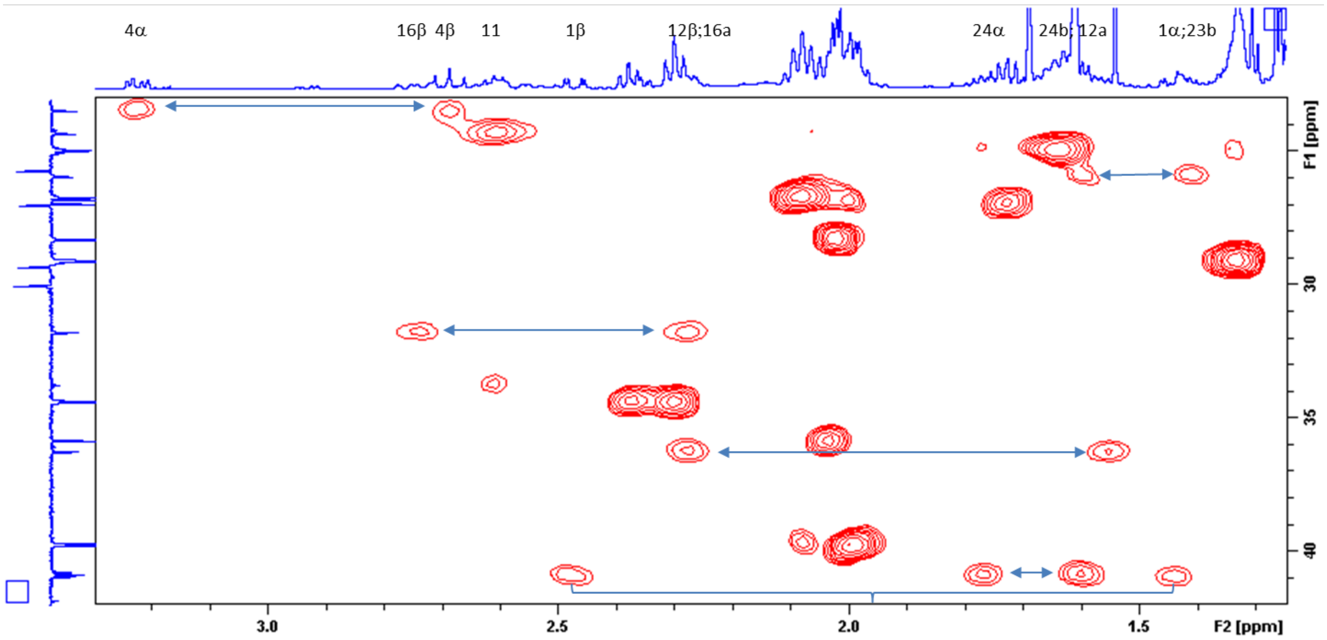

S31. Compound **14** ROESY Me-section + HMBC Me-section

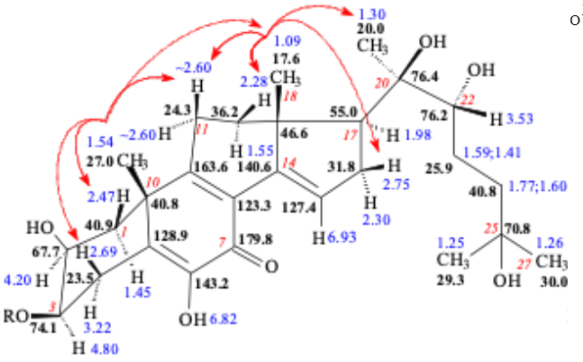

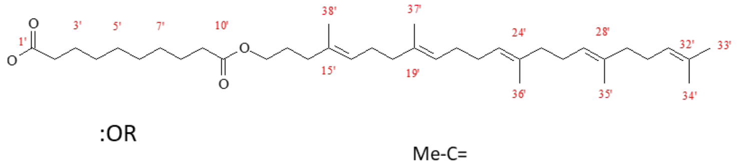

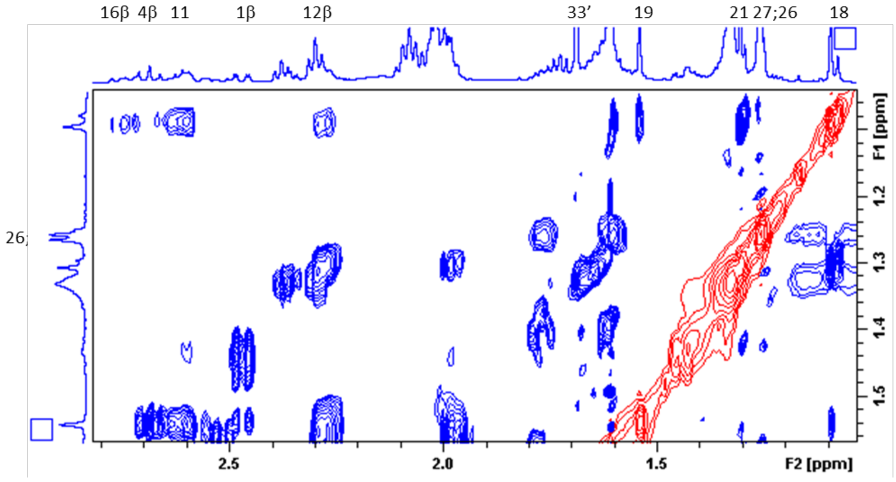

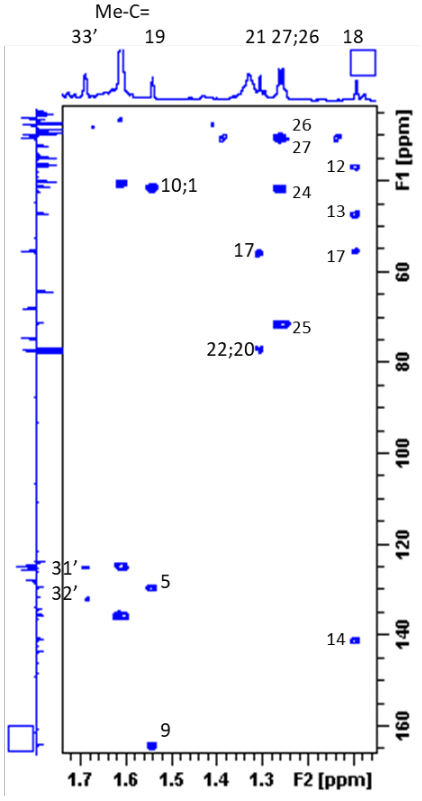

S32. Compound **15** ^{1}H NMR

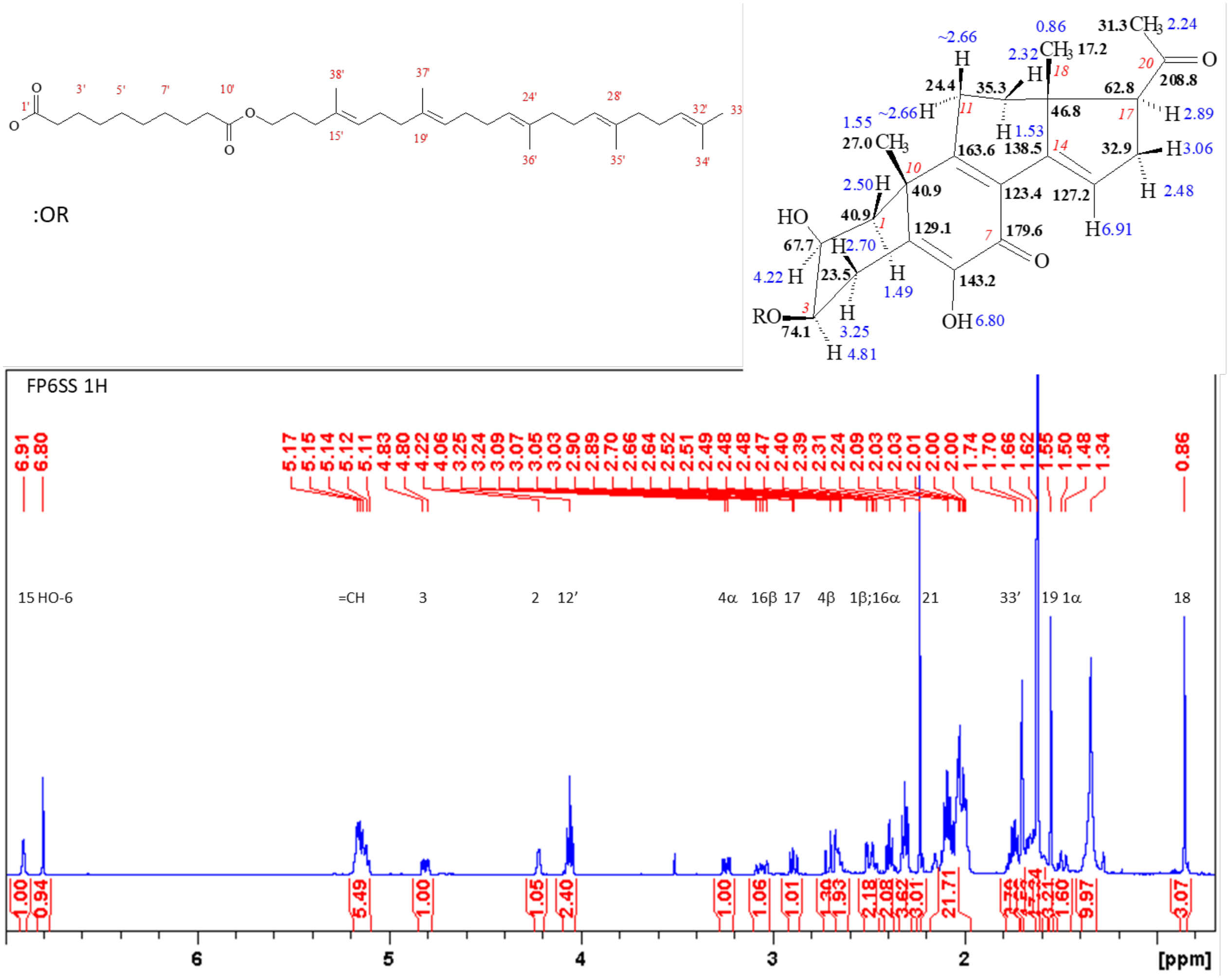

S33. Compound **15** ^{13}C APT

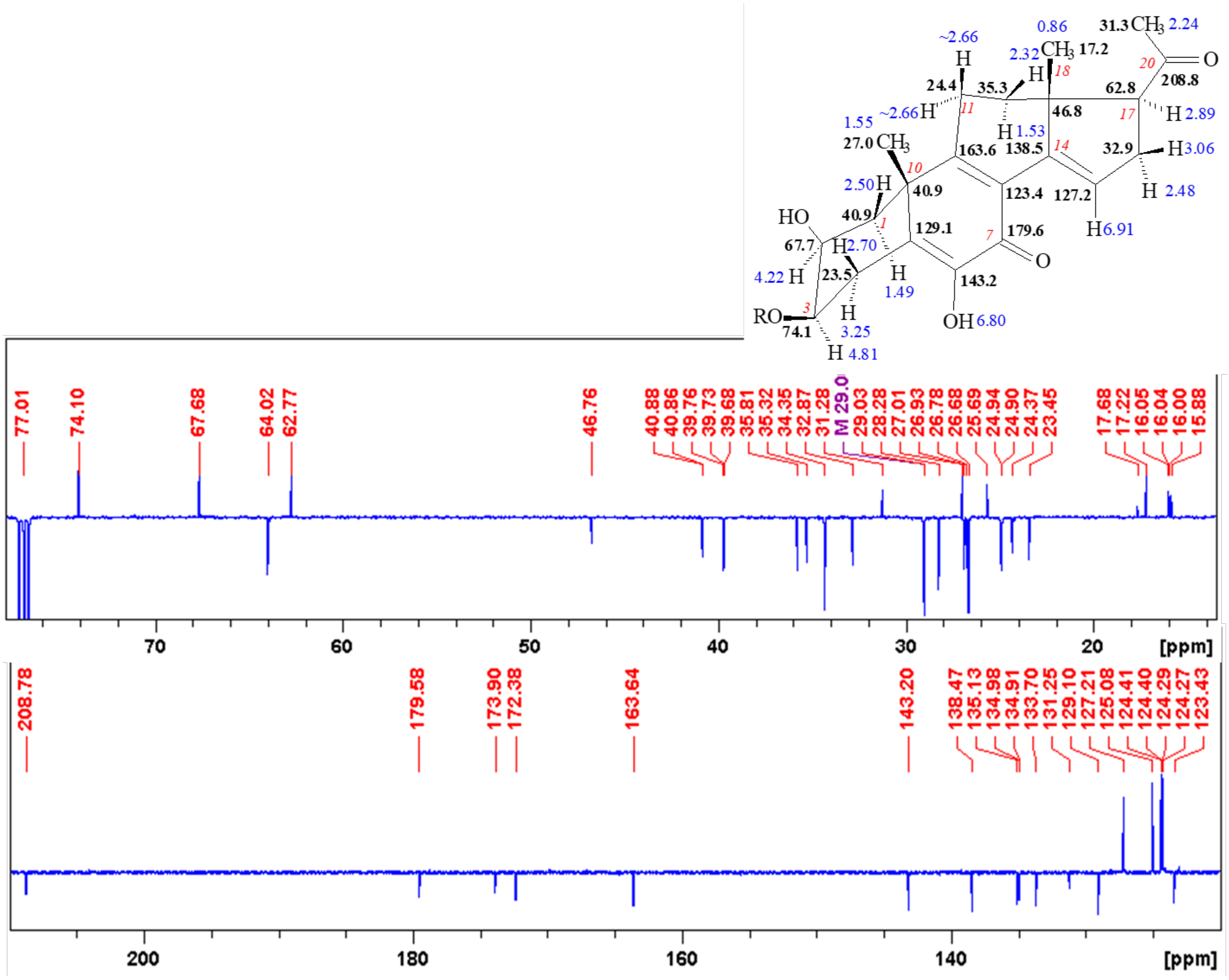

S34. Compound **15** edHSQC

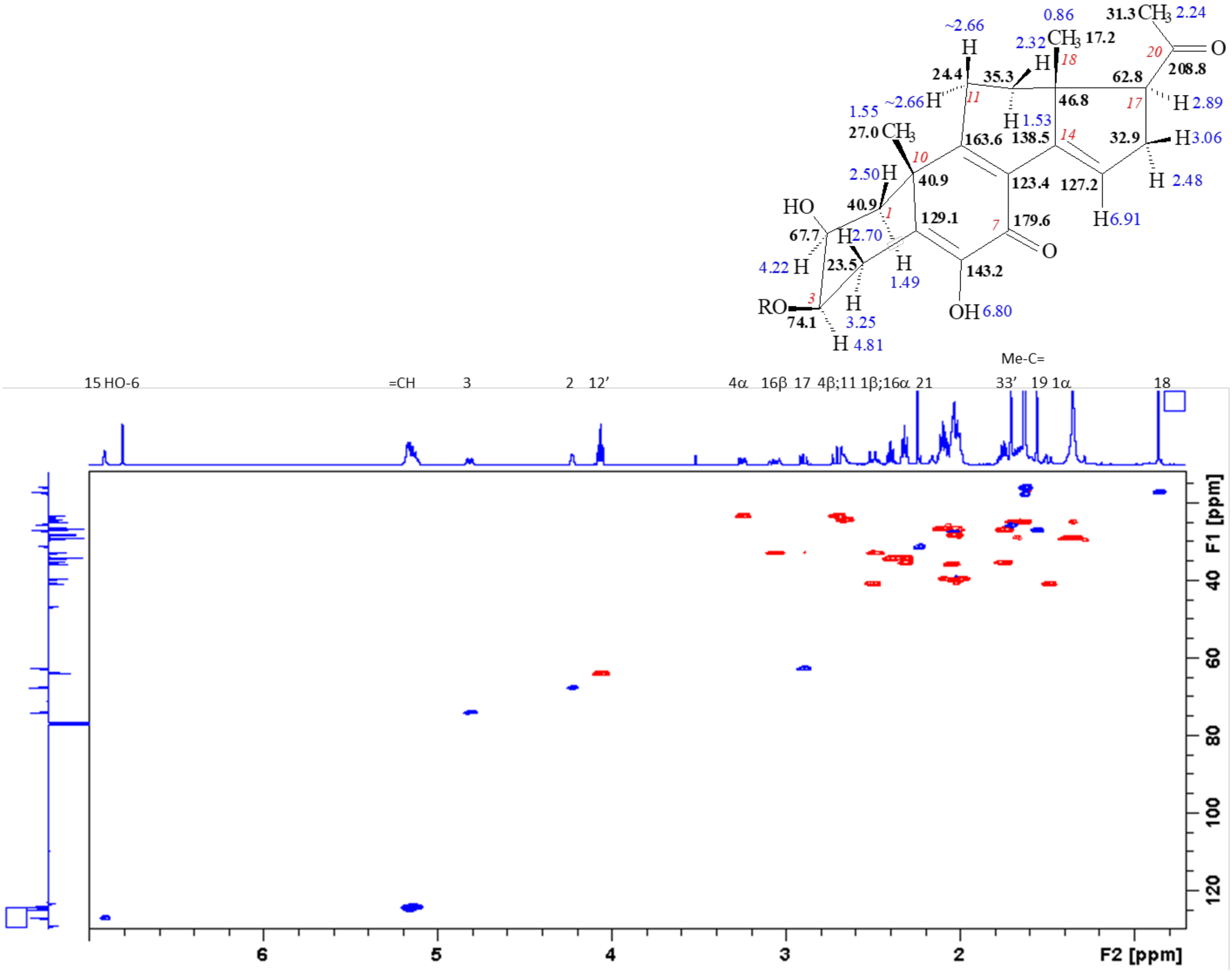

S35. Compound **15** HMBC

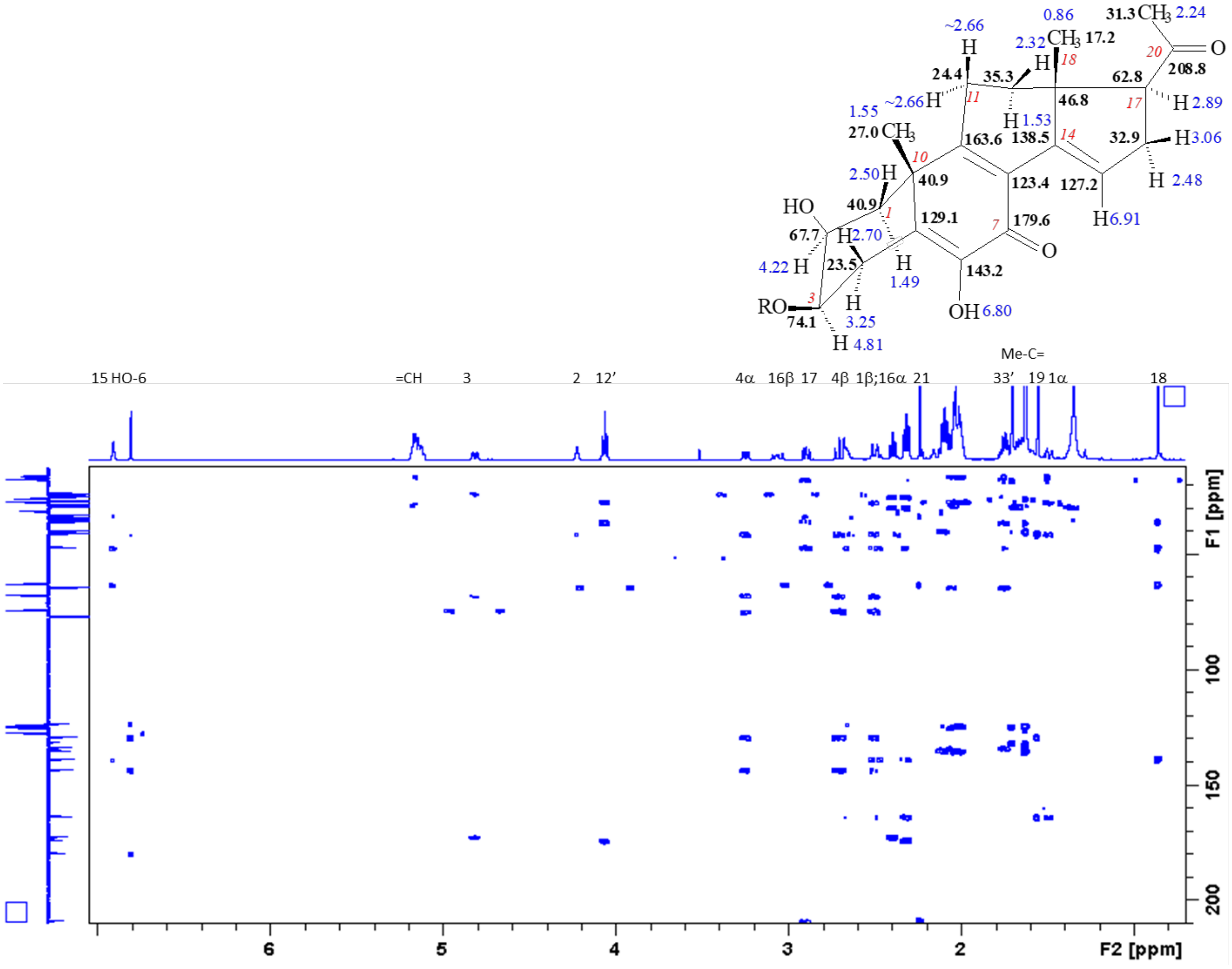

S36. Compound **15** ROESY

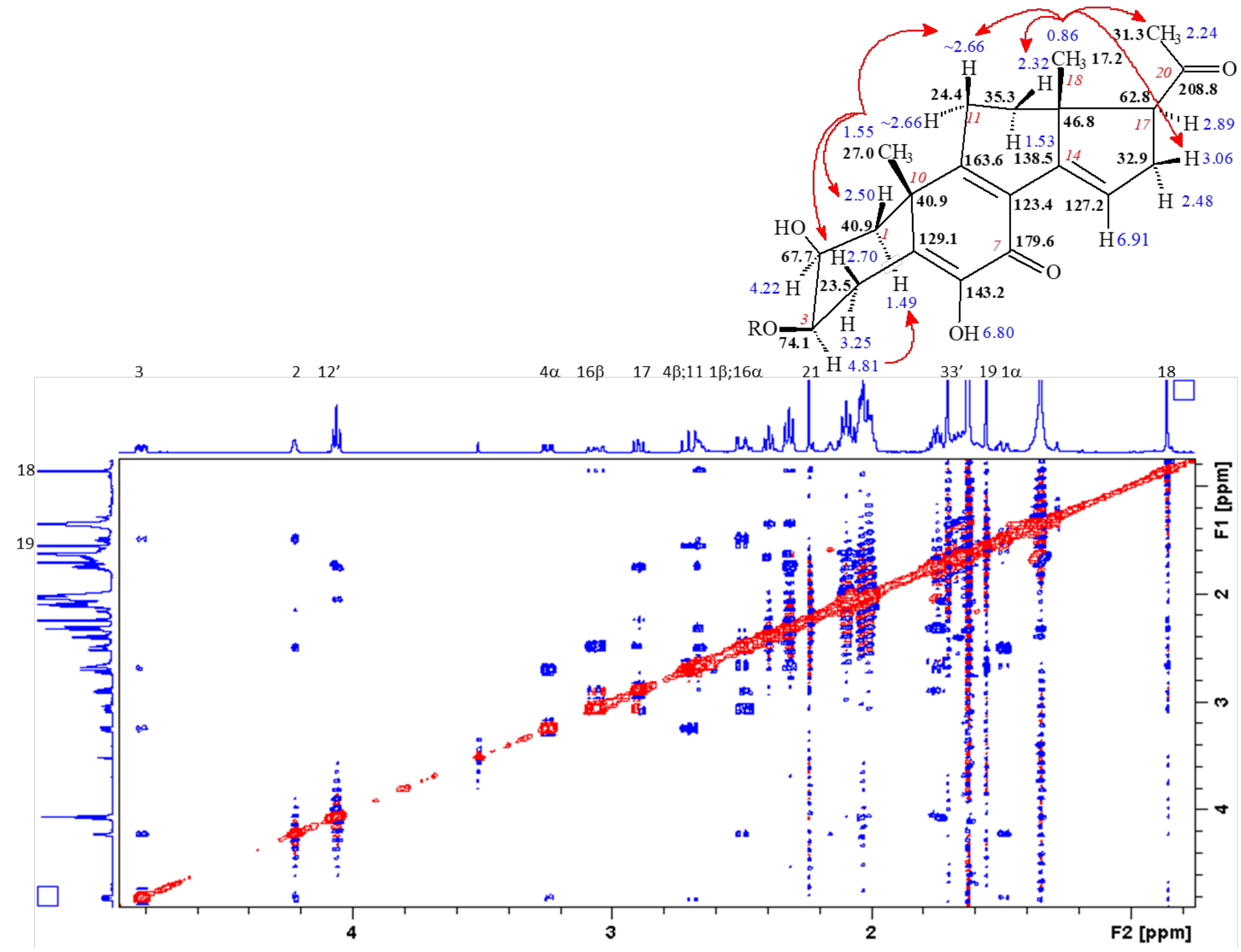

S37. Compound **16** ^{1}H NMR

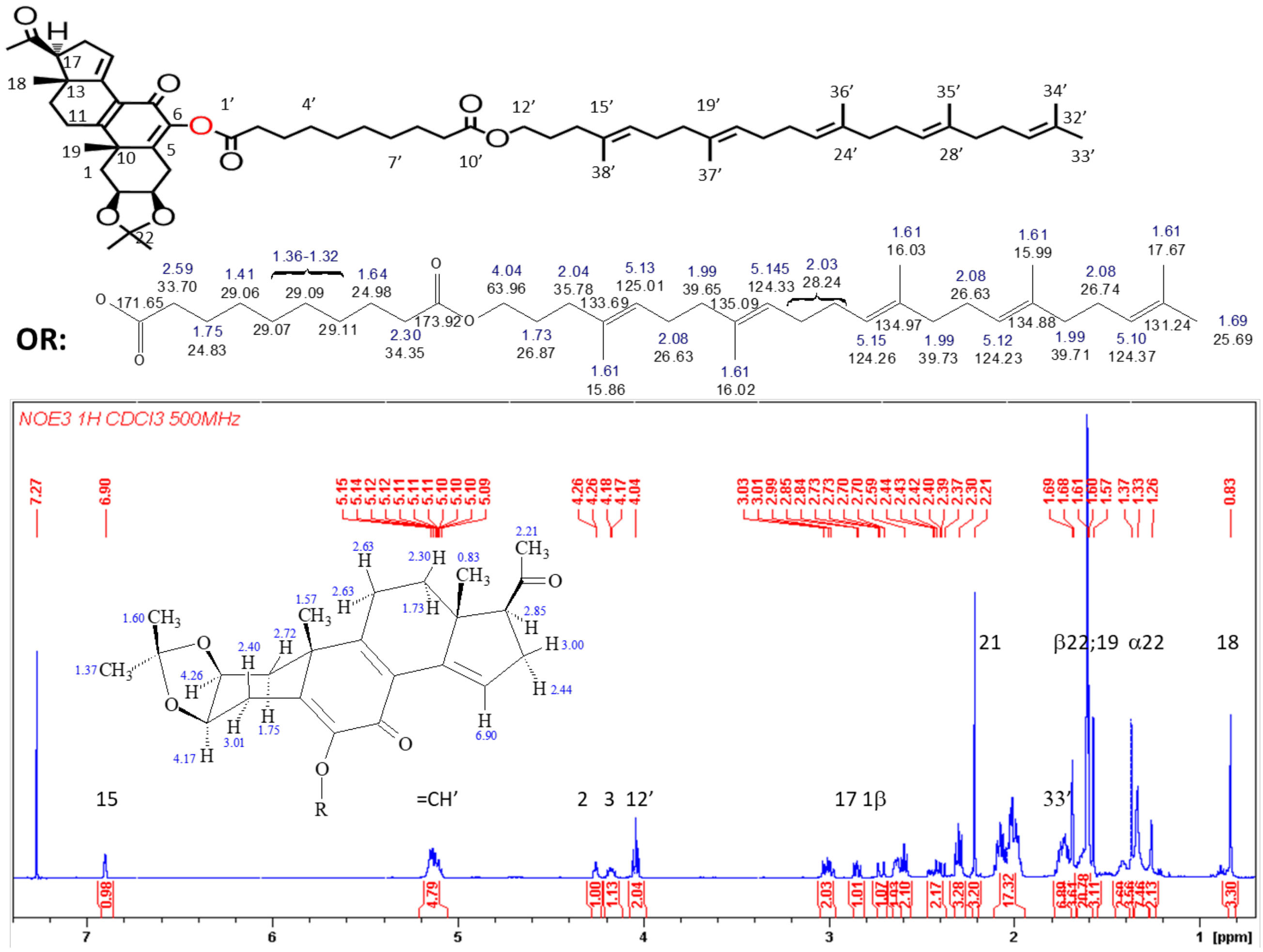

S38. Compound **16** ^{1}H NMR section

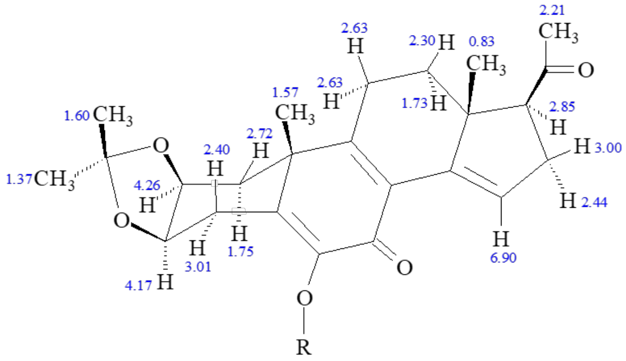

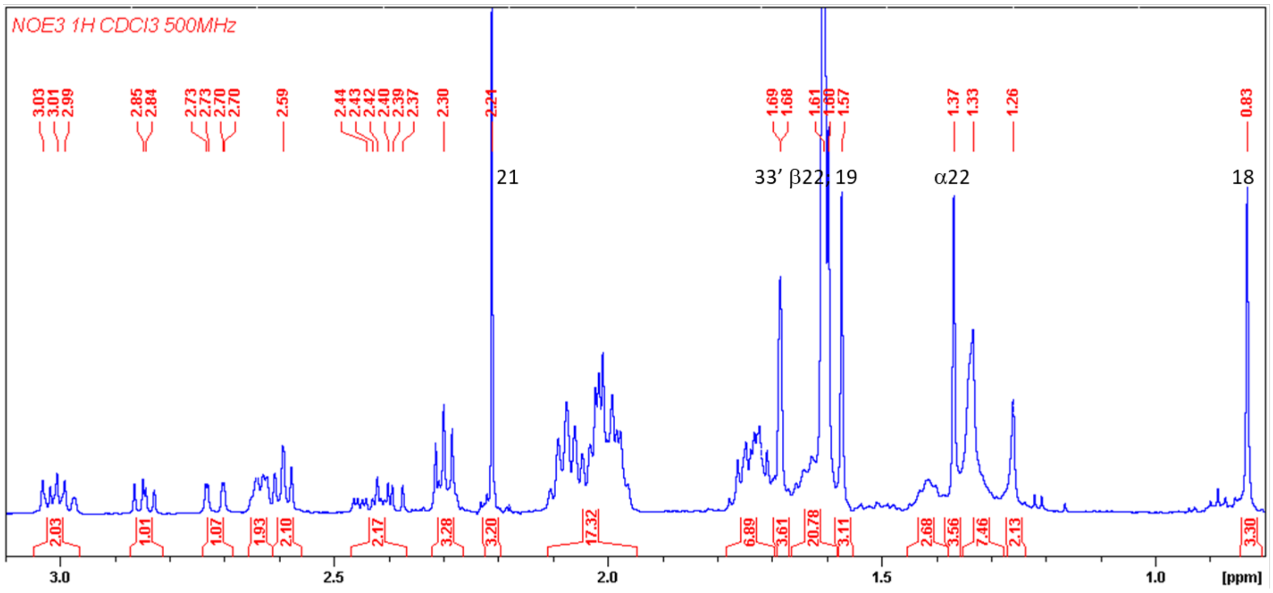

S39. Compound **16** ^{1}H NMR + selTOCSY on (4.04/6.90/4.26) t_{mix}=120ms.

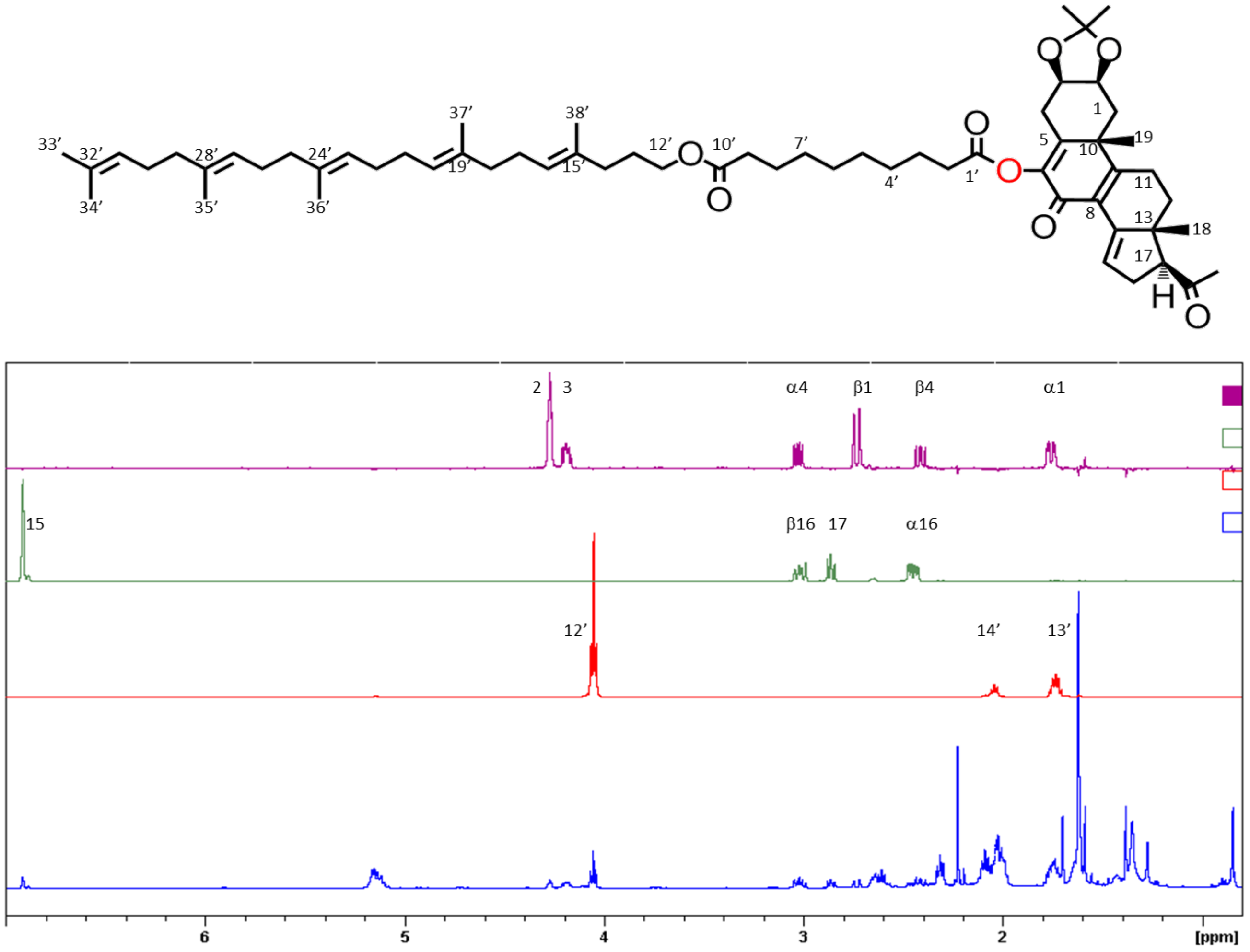

S40. Compound **16** ^{13}C DEPTQ

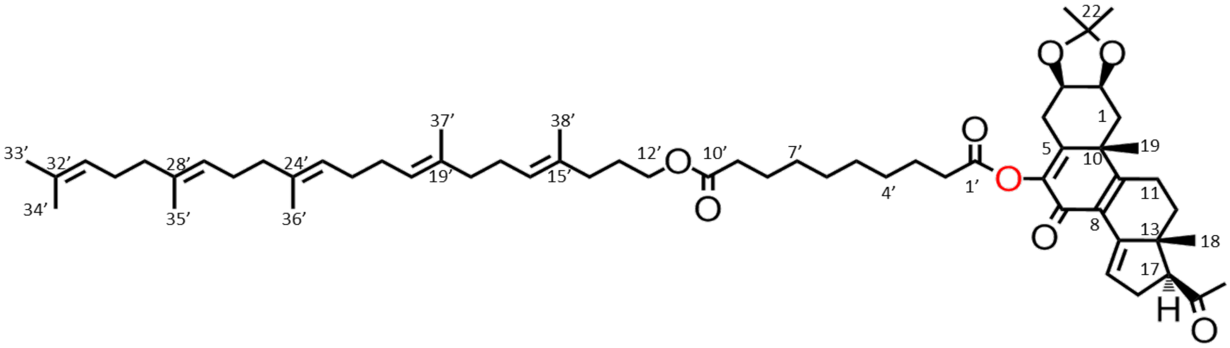

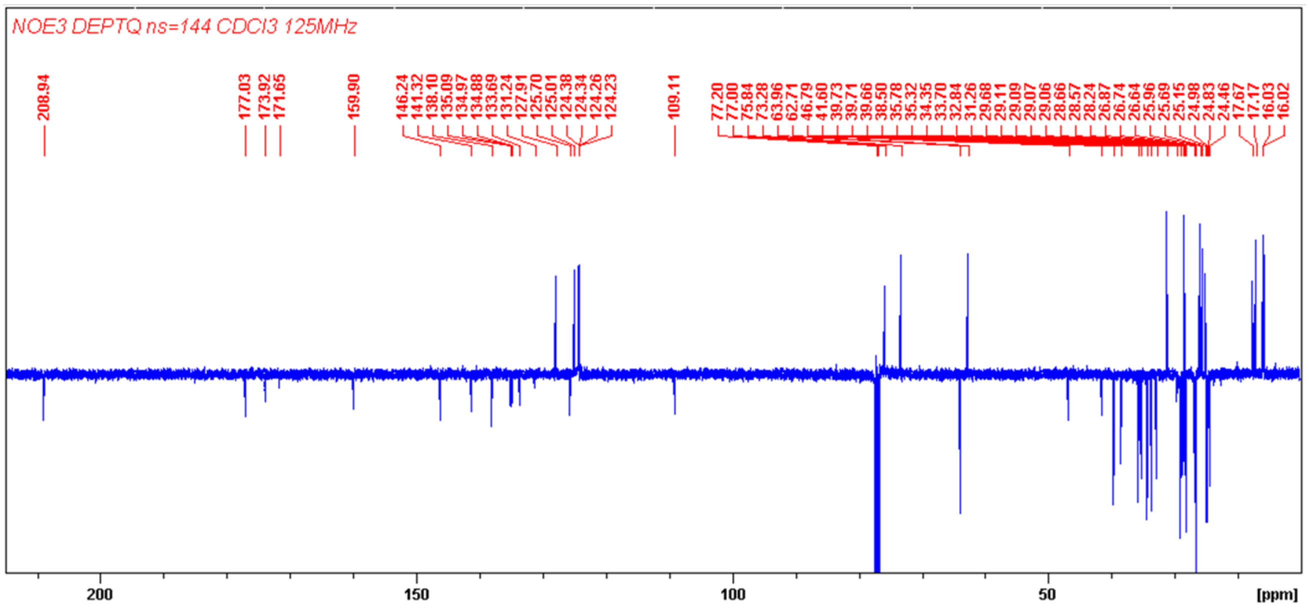

S41. Compound **16** ^{13}C DEPTQ section

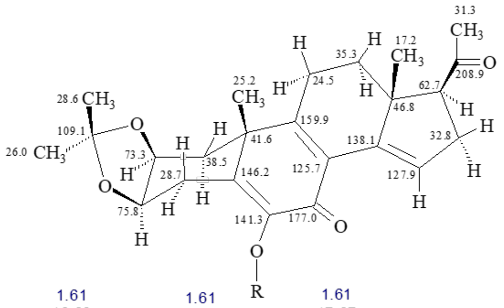

OR:

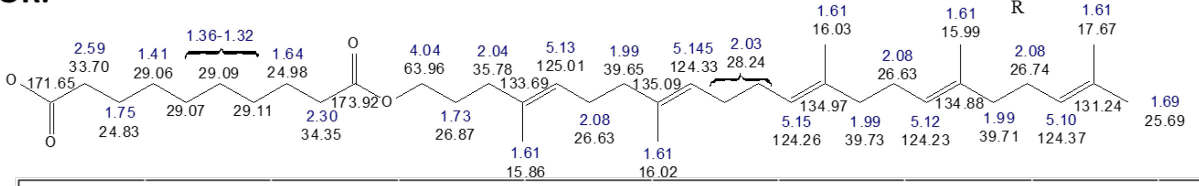

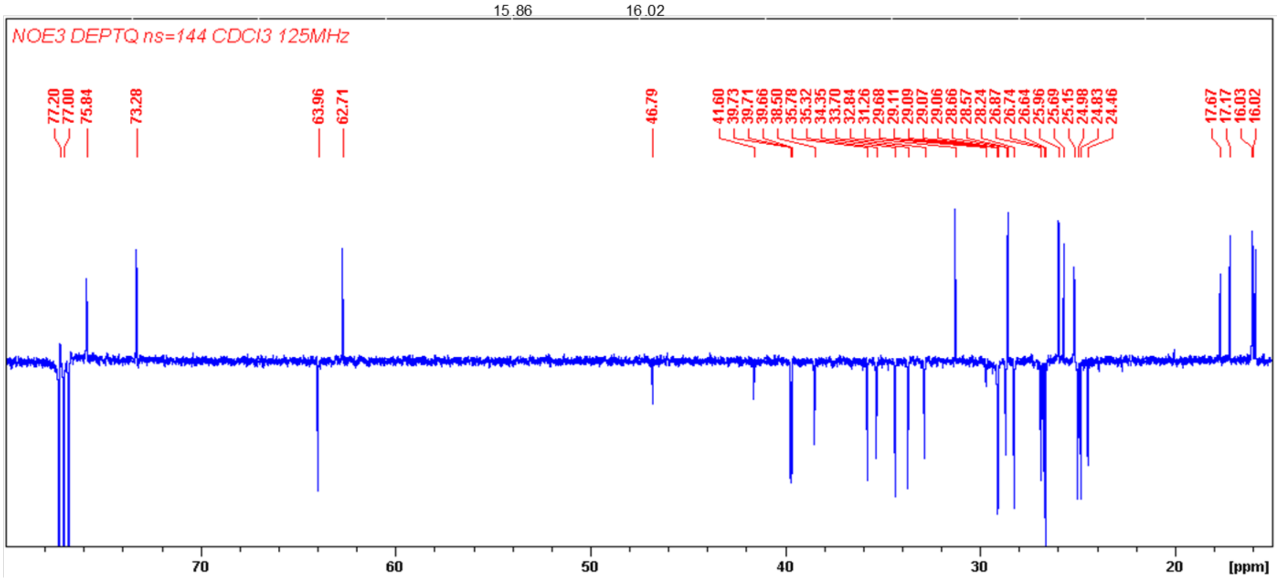

S42. Compound **16** HSQC

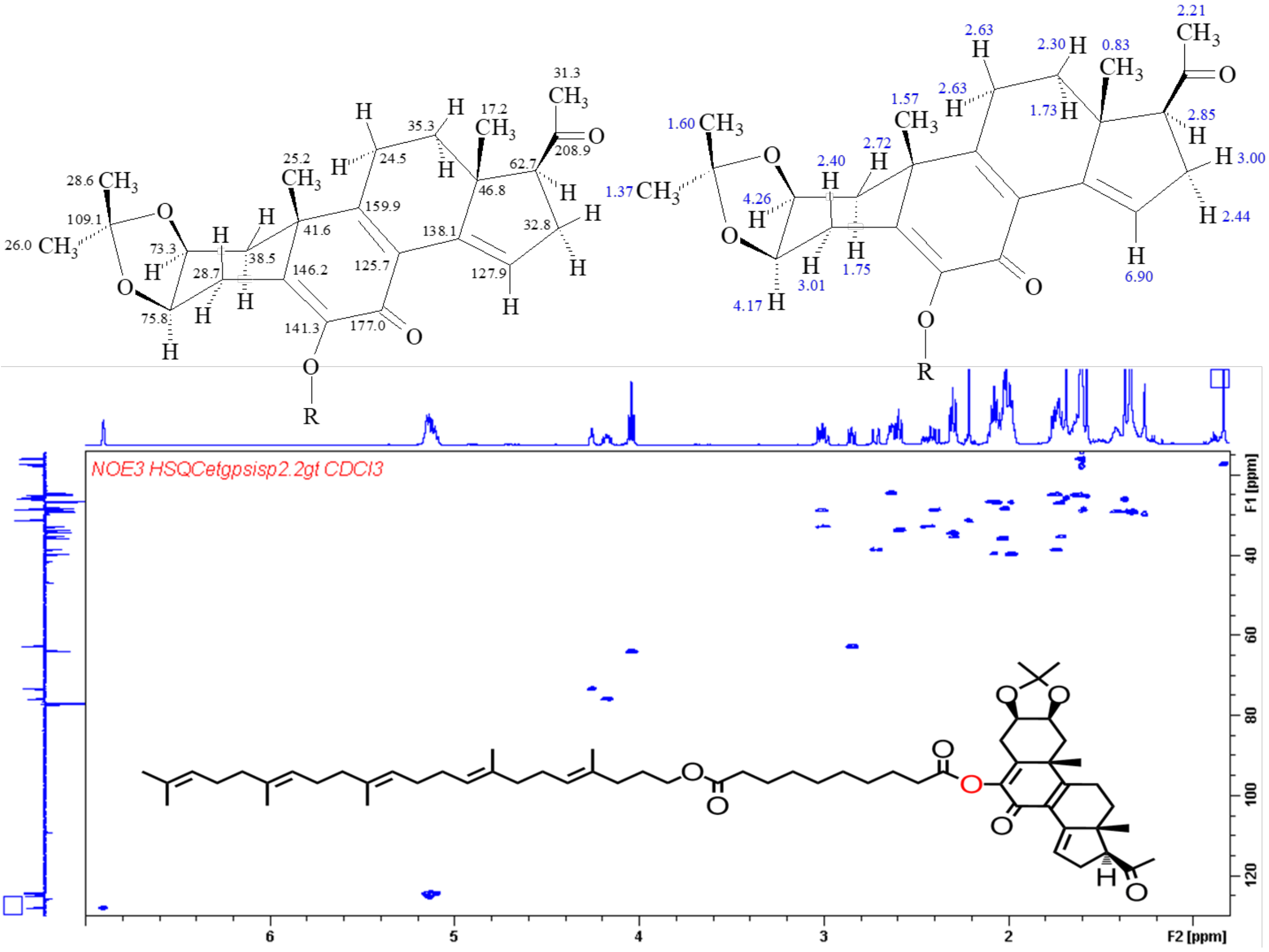

S43. Compound **16** HSQC section

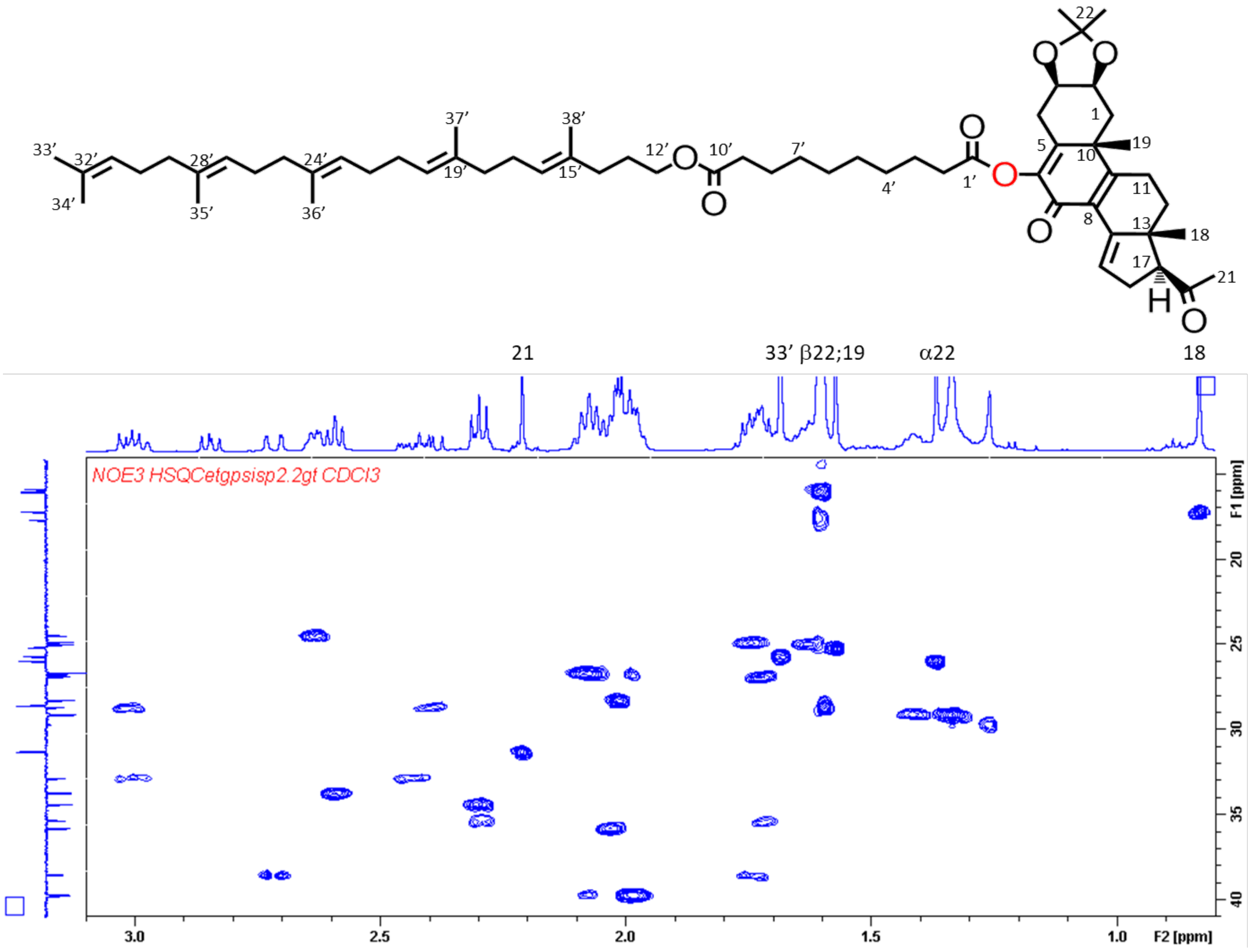

S44. Compound **16** edHSQC CH_2 section

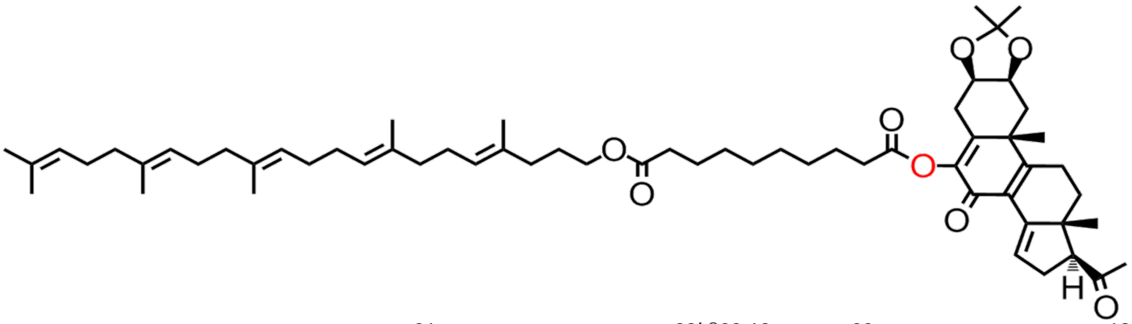

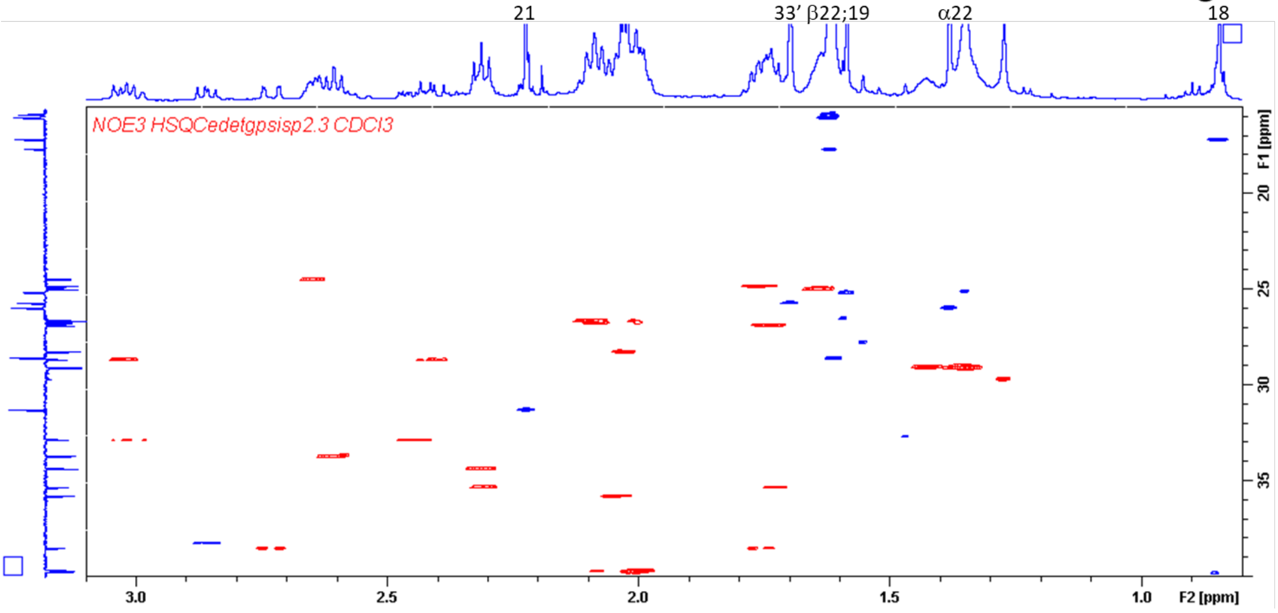

S45. Compound **16** HMBC

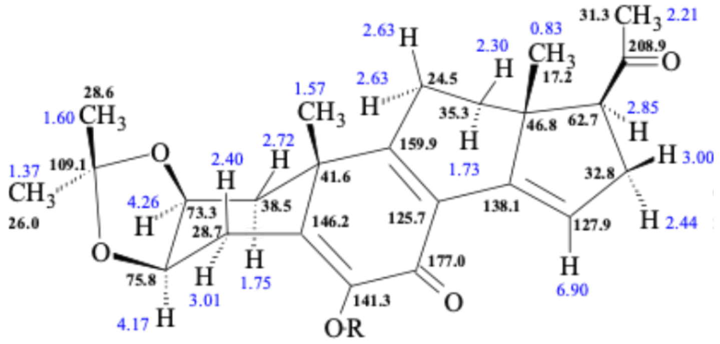

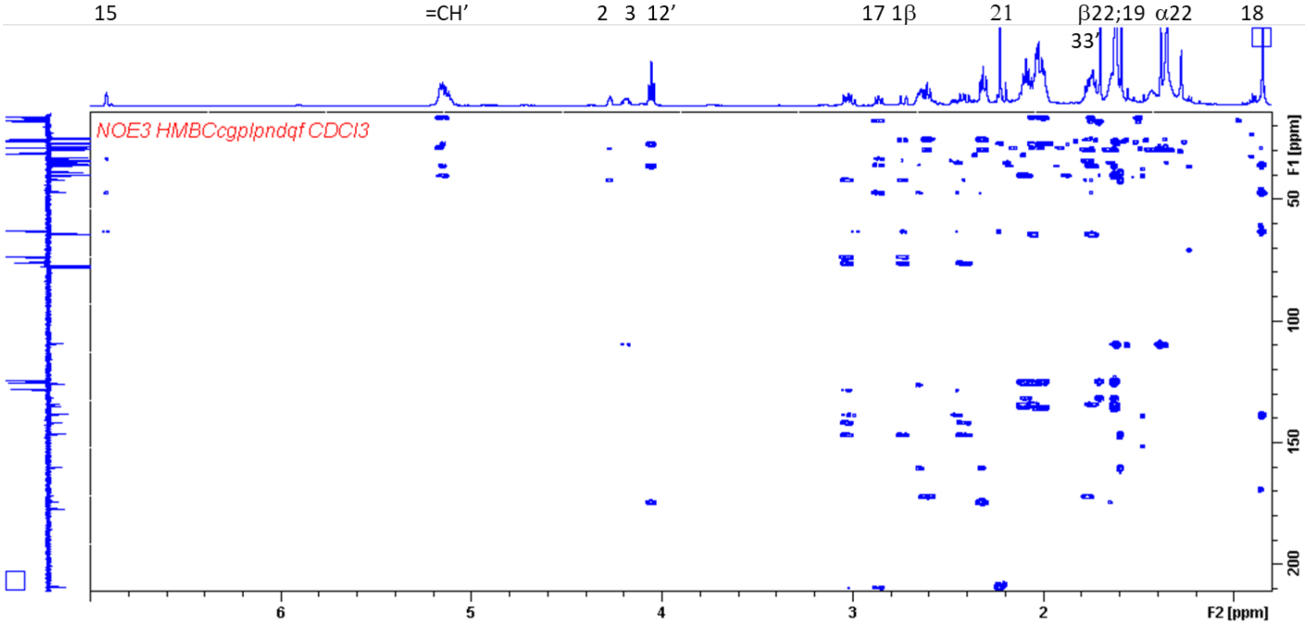

S46. Compound **16** HMBC section

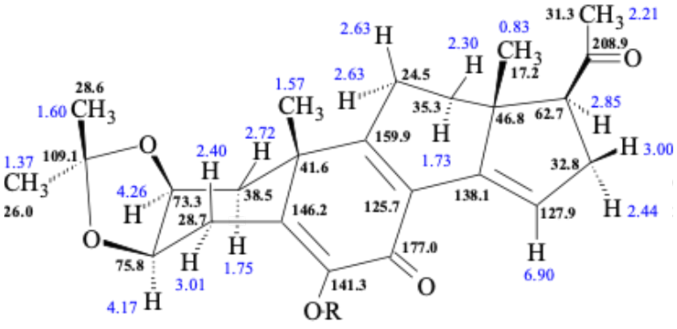

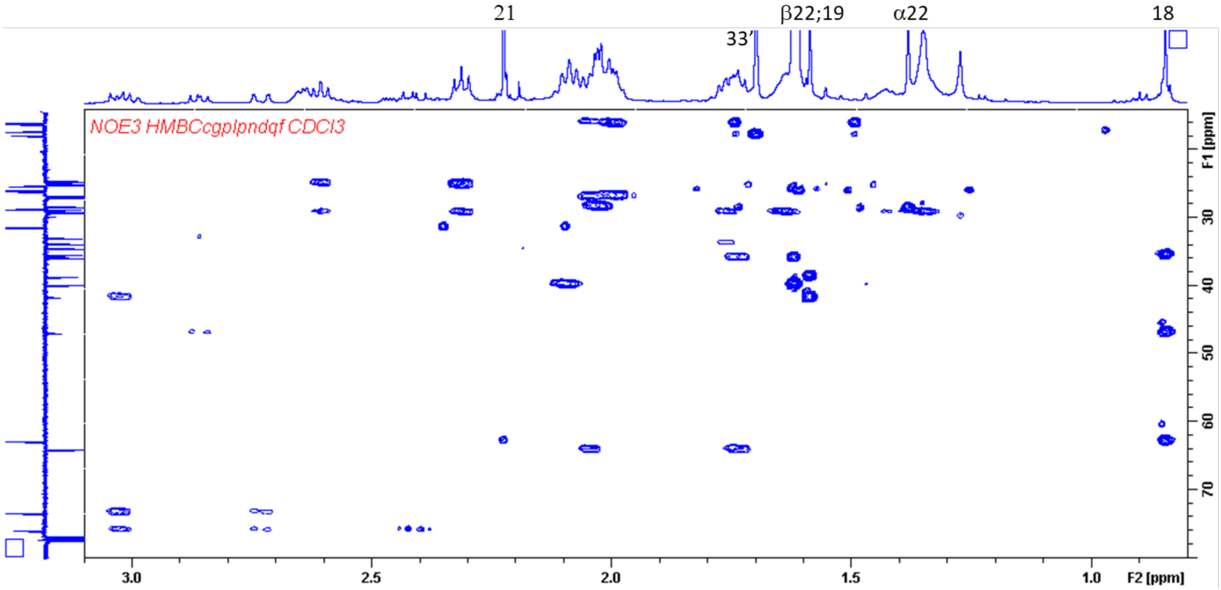

S47. Compound **16** ^{13}C DEPTQ + selINEPT (d4.04t/2.30m/2.59t)

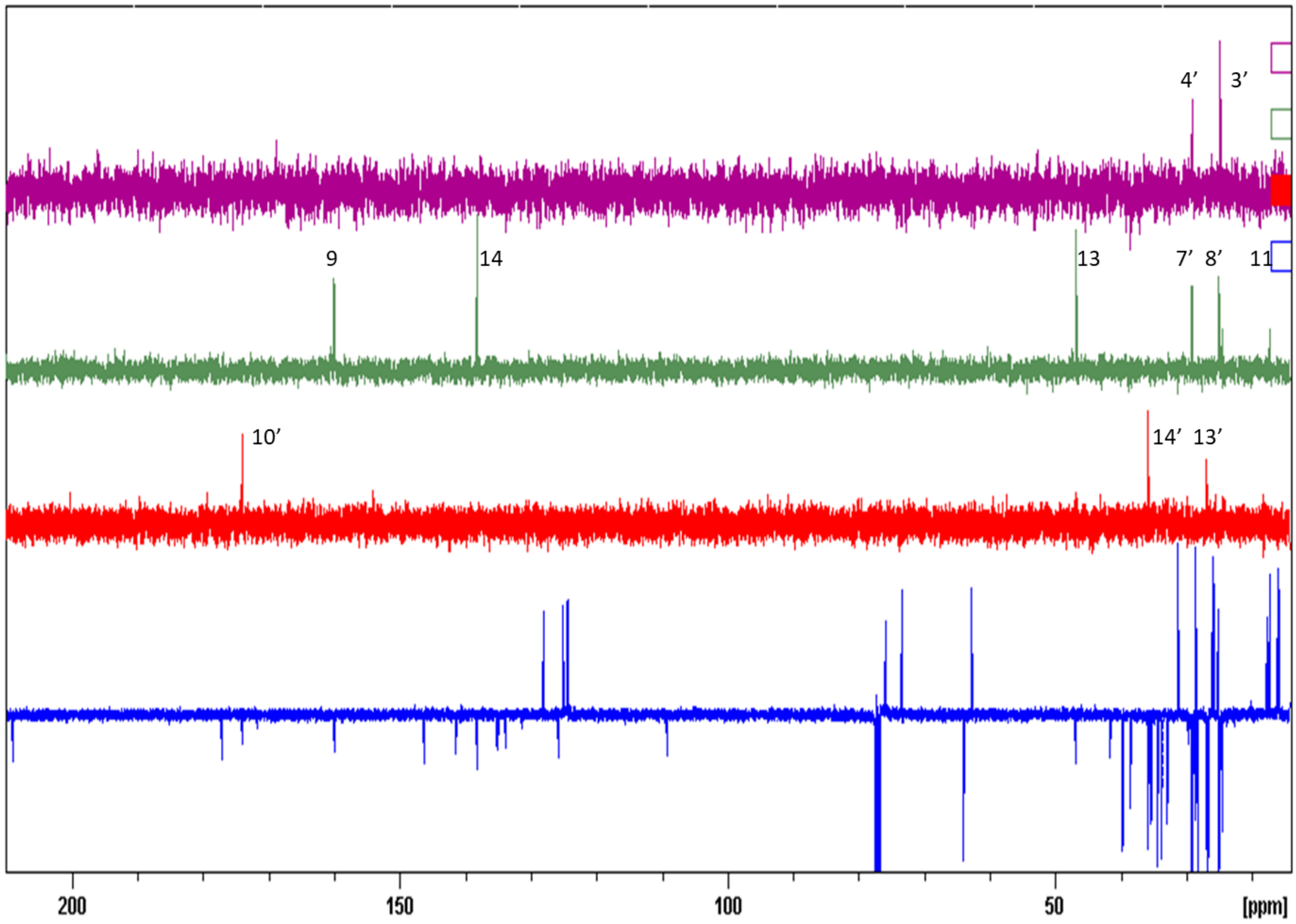

S48. Compound **16** ^{13}C DEPTQ + selINEPT (d4.04t/2.30t/2.59t) section

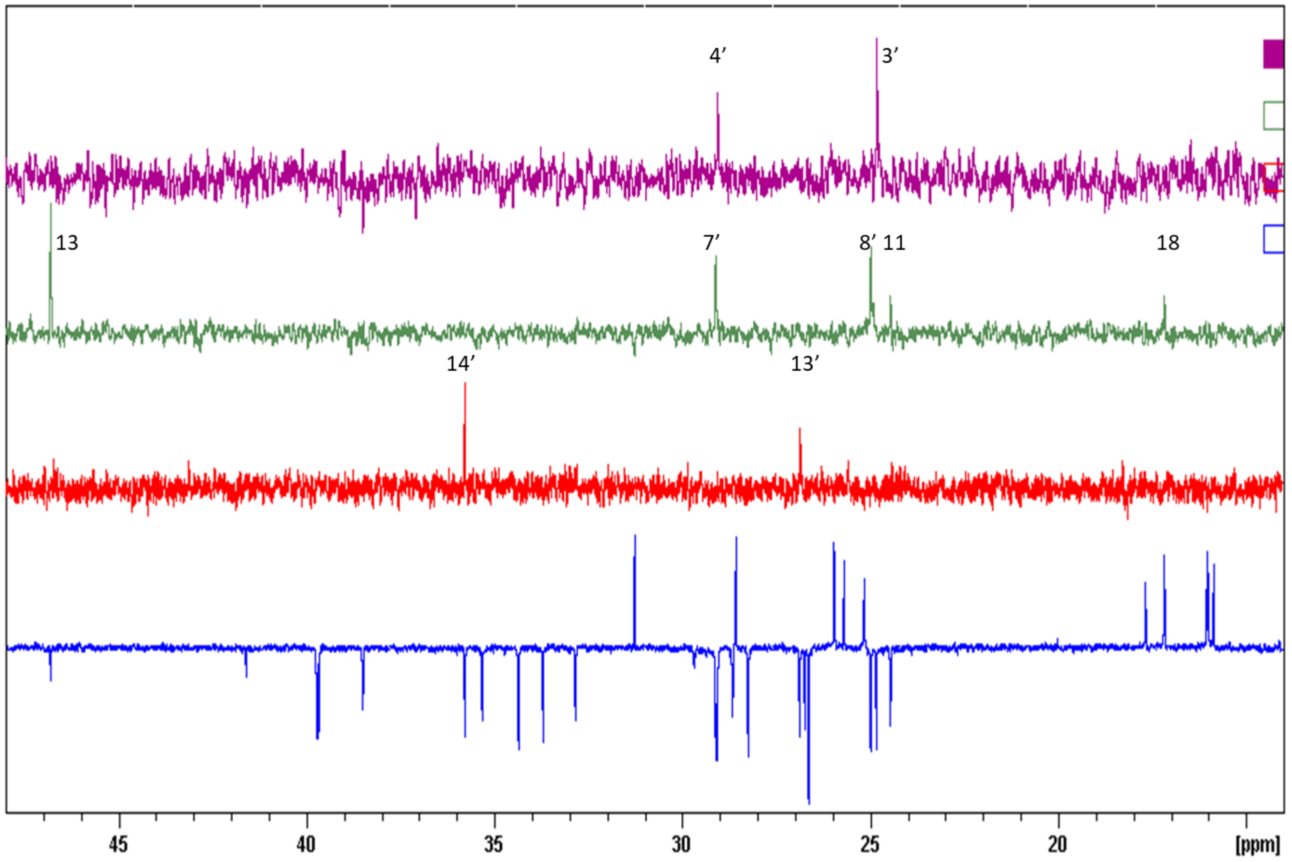

S49. Compound **17** ^{1}H NMR

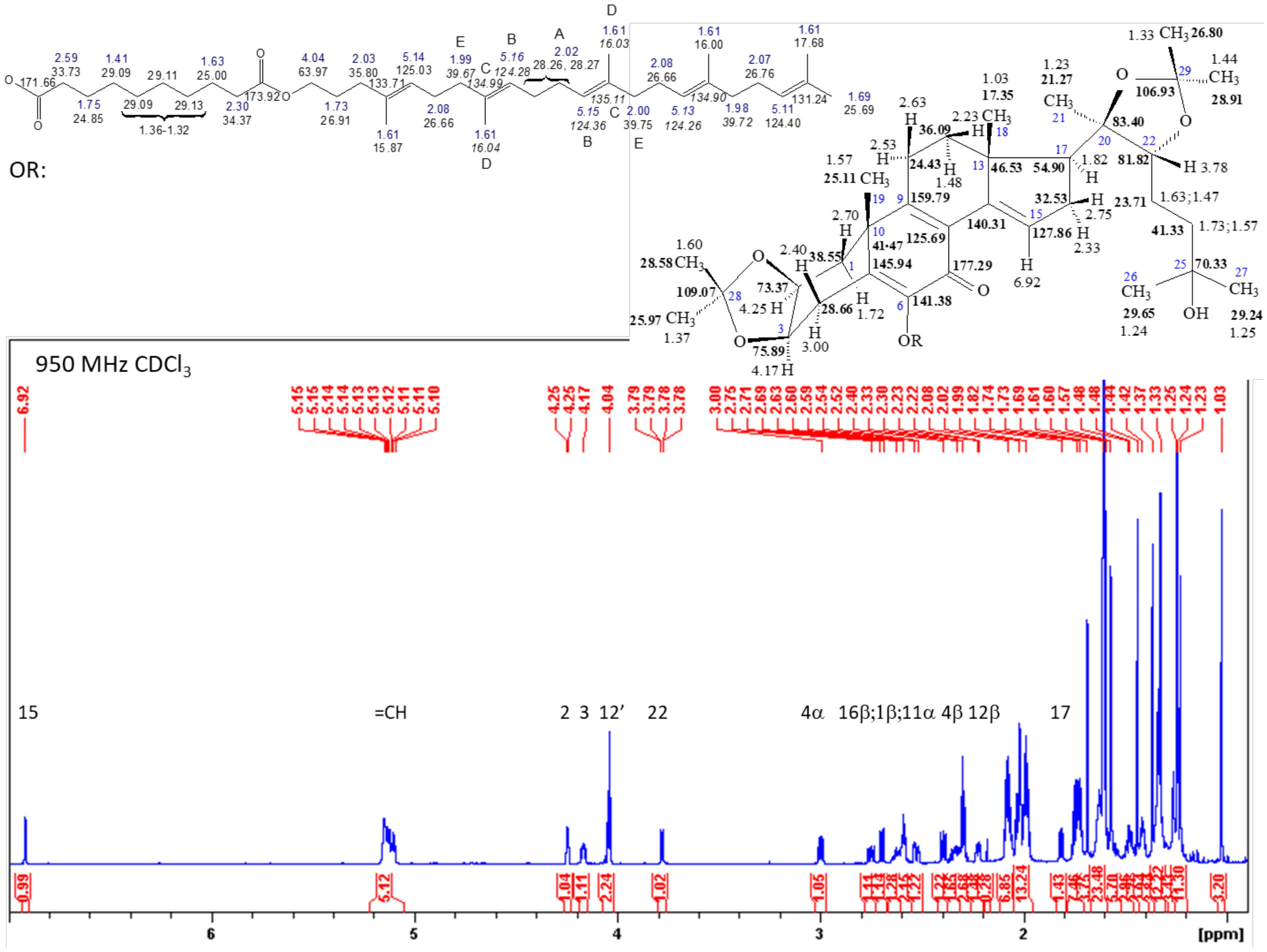

S50. Compound **17** ^{1}H + selTOCSY 15, 12’ and 22

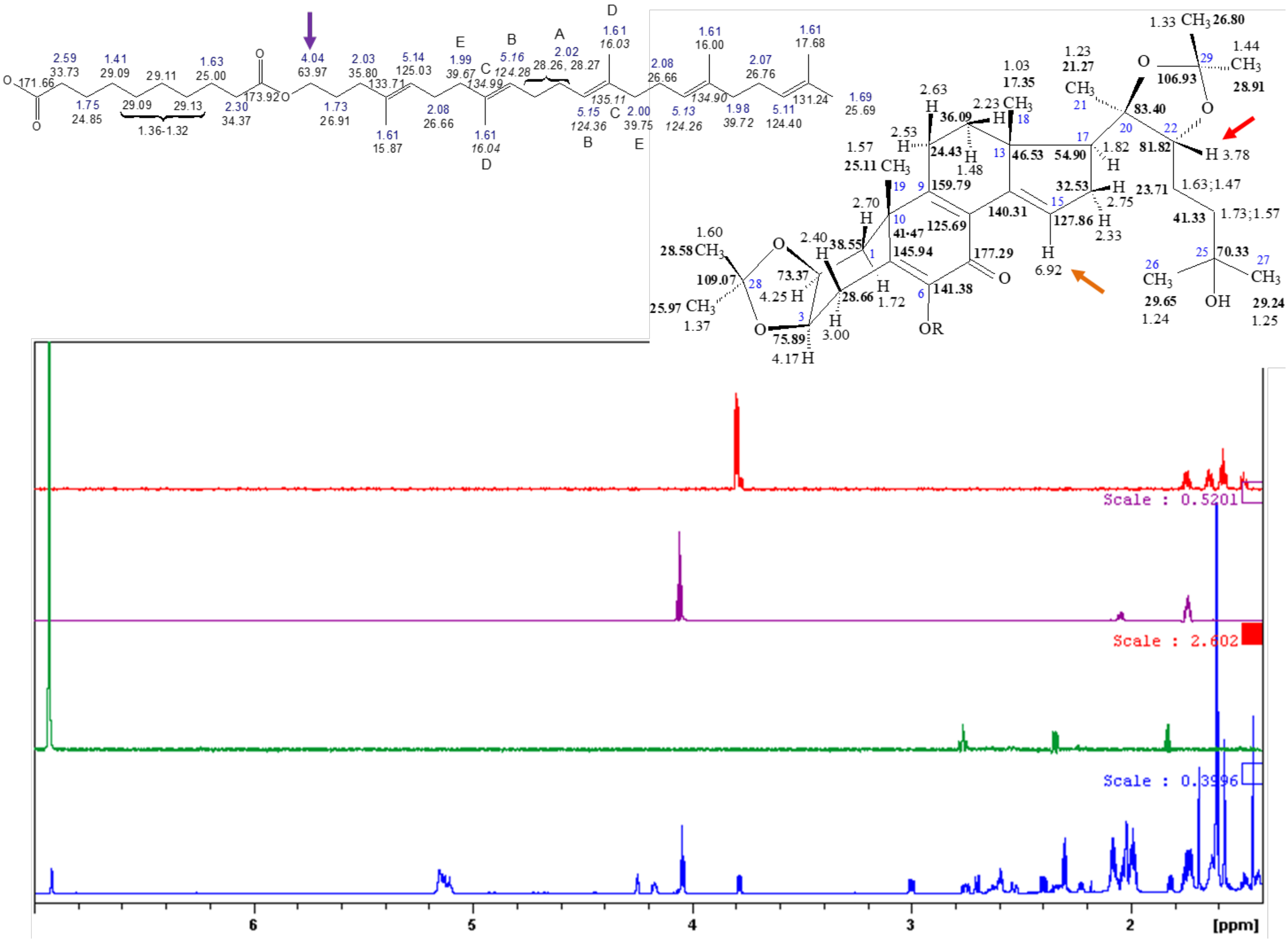

S51. Compound **17** ^{13}C NMR

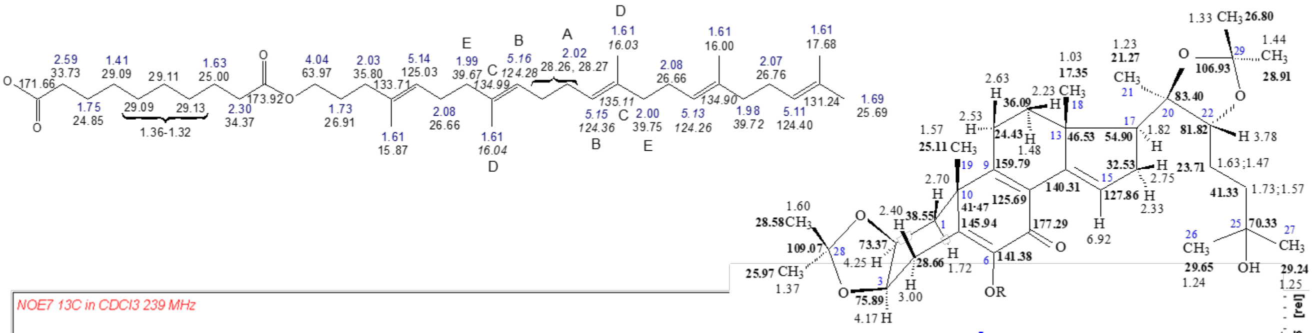

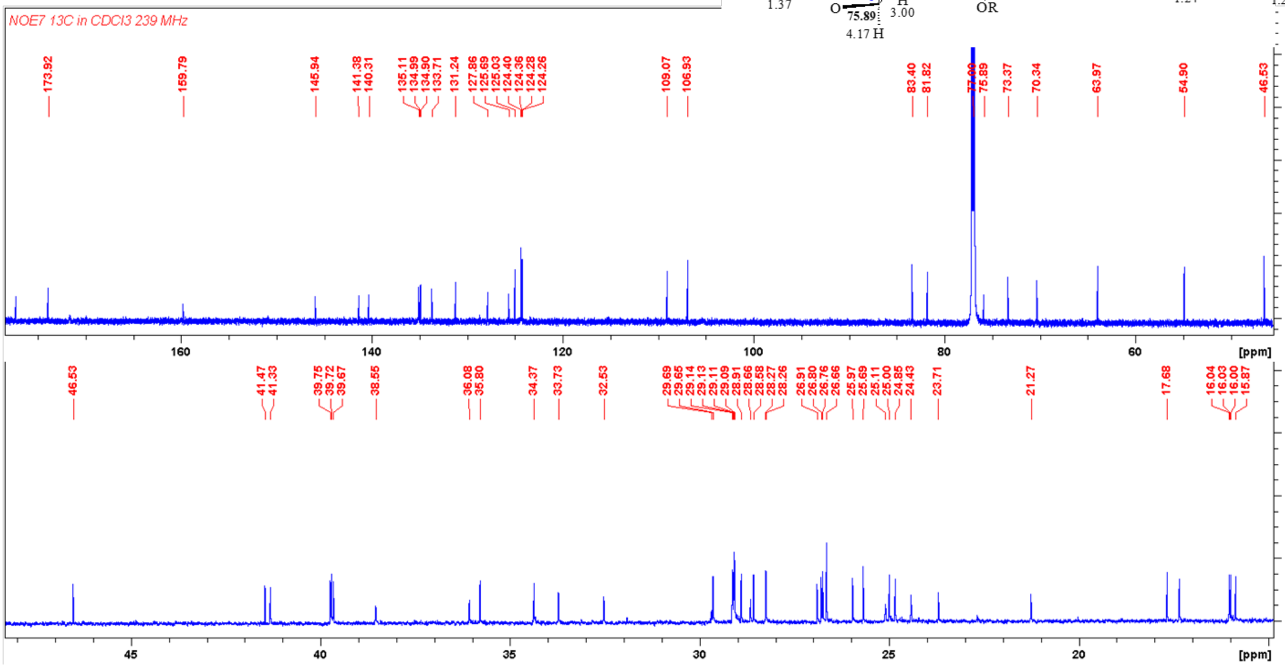

S52. Compound **17** edHSQC + =CH section

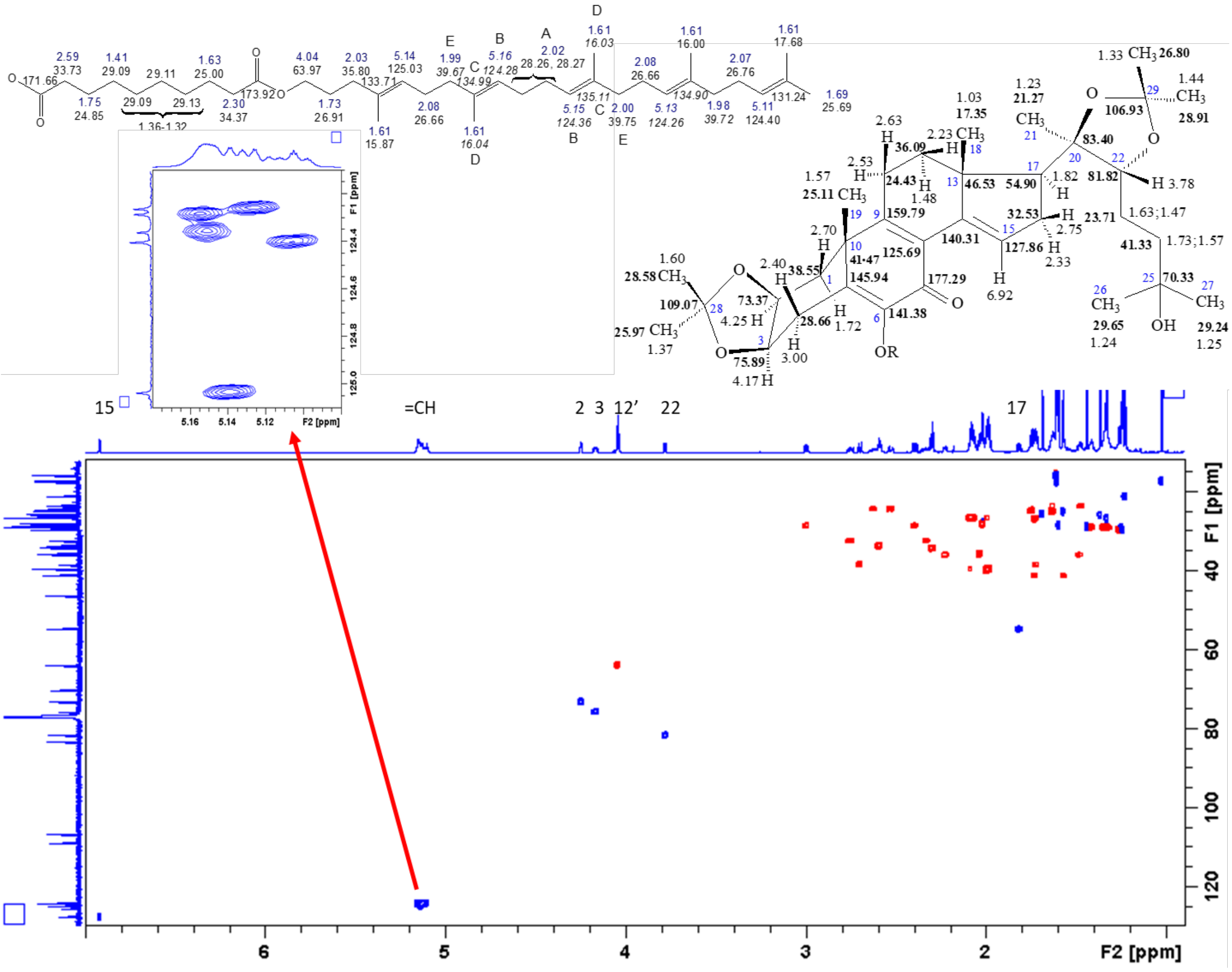

S53. Compound **17** edHSQC CH_2 section

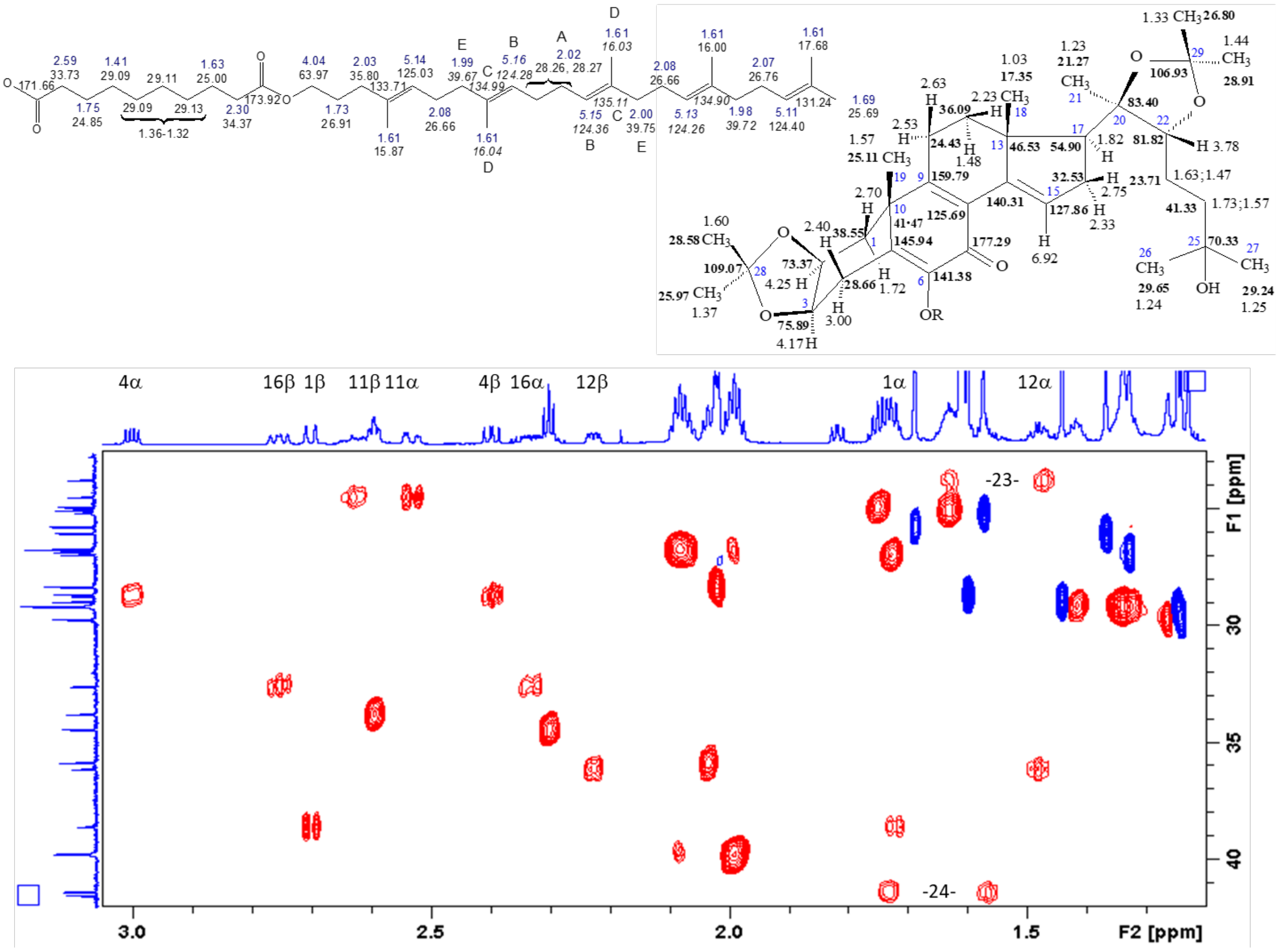

S54. Compound **17** sel. HSQC sections (33–43 and 32–22 ppm)

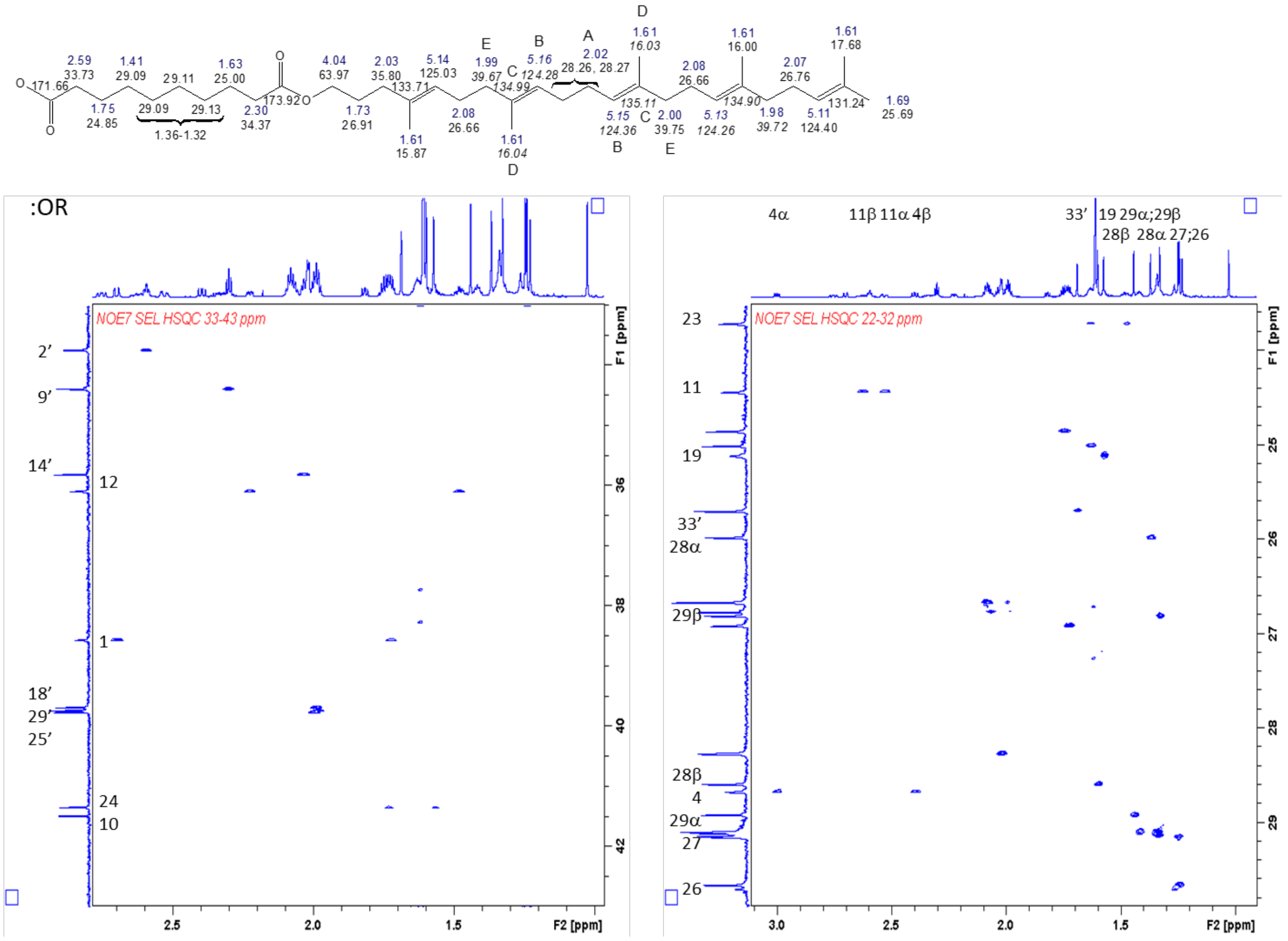

S55. Compound **17** edHSQC and HMBC CH_3 sections

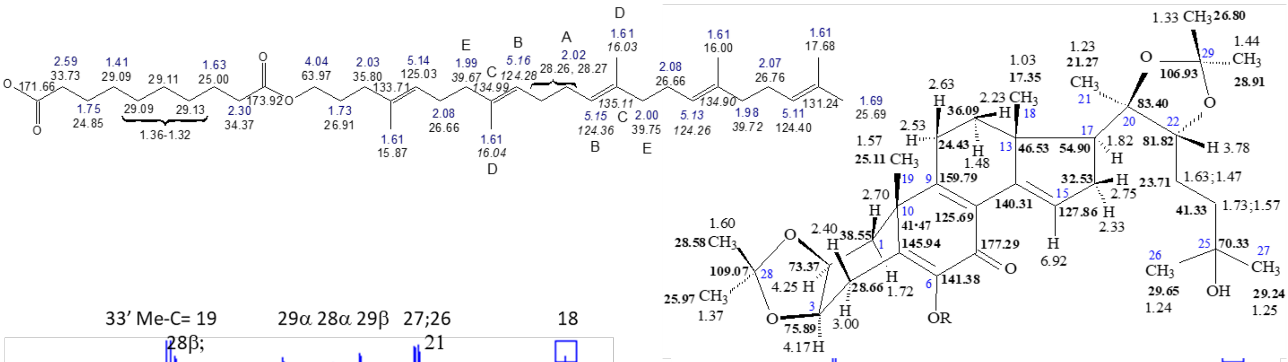

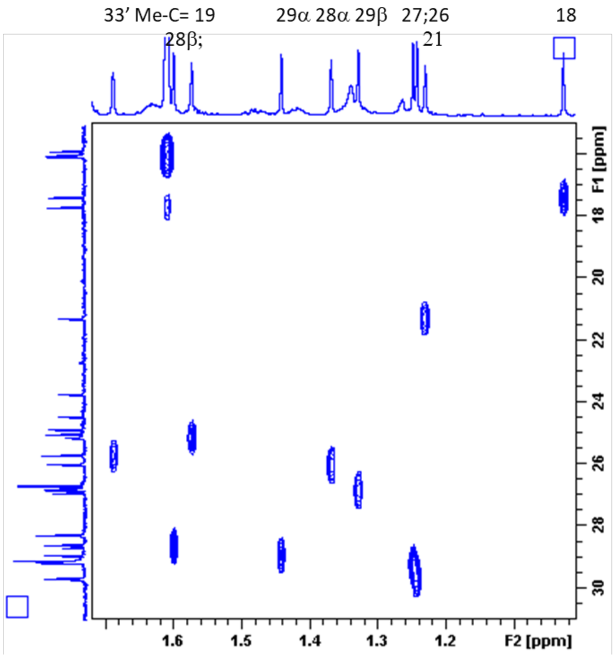

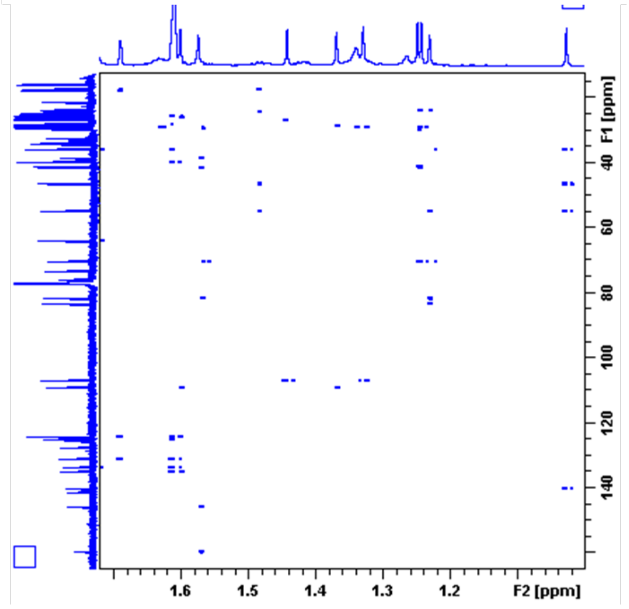

S56. Compound **17** HMBC

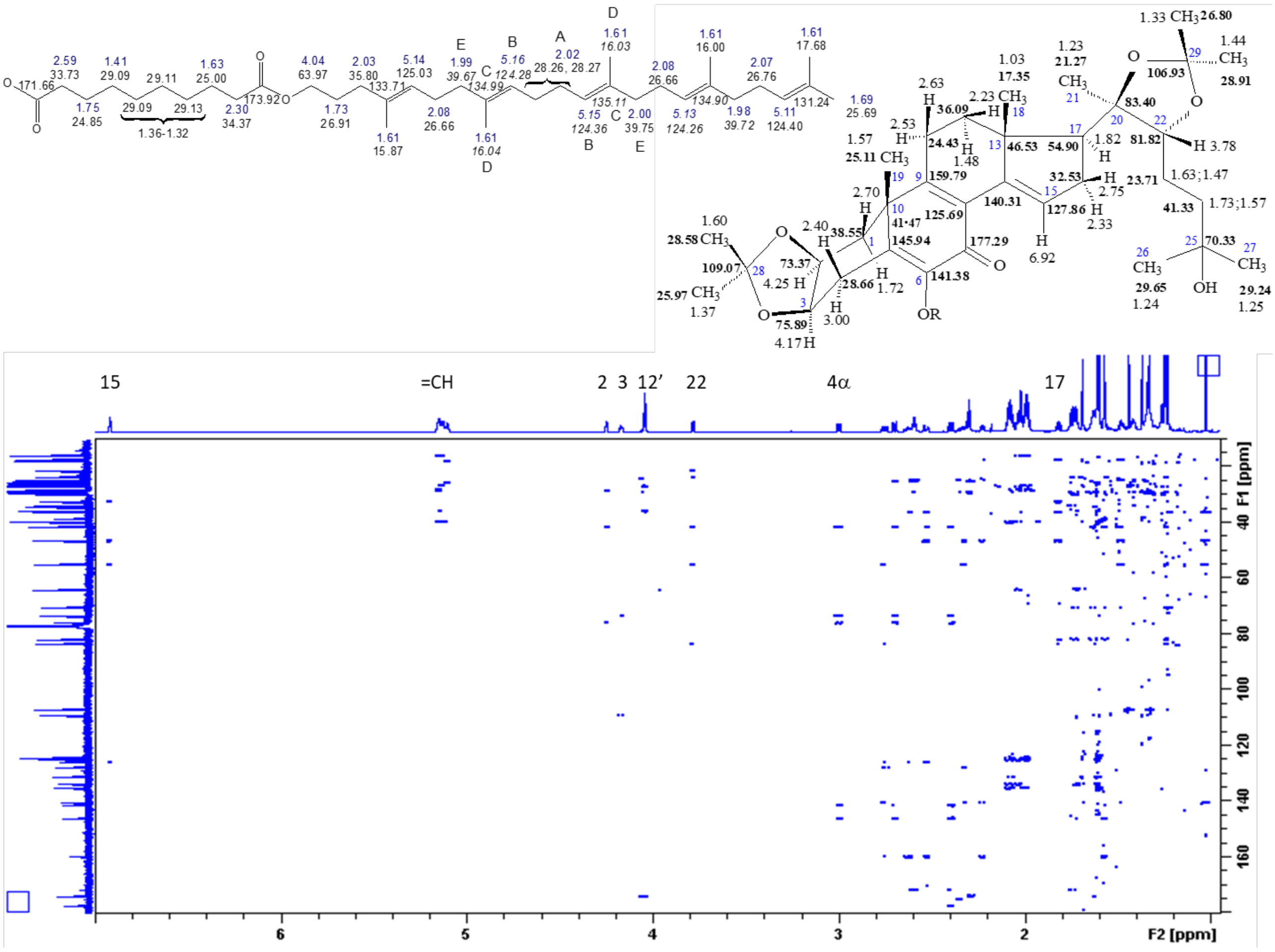

S57. Compound **18** ^{1}H NMR

OR:

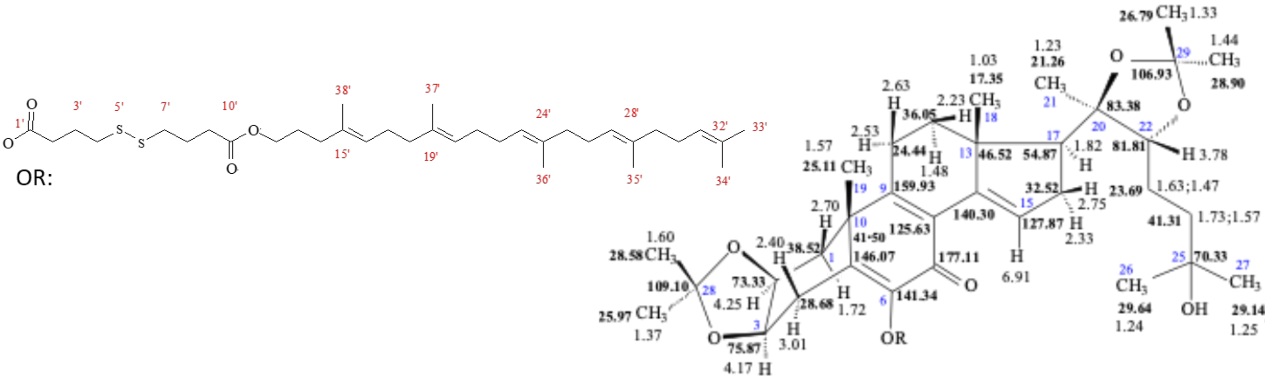

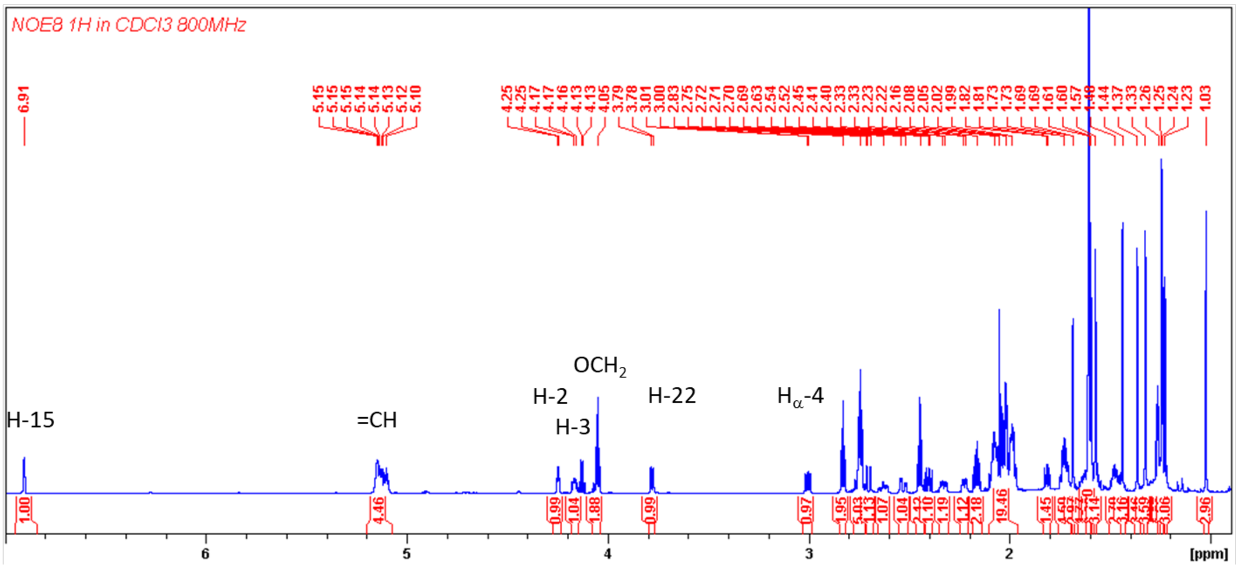

S58. Compound **18** ^{1}H NMR section 3.1–0.9 ppm

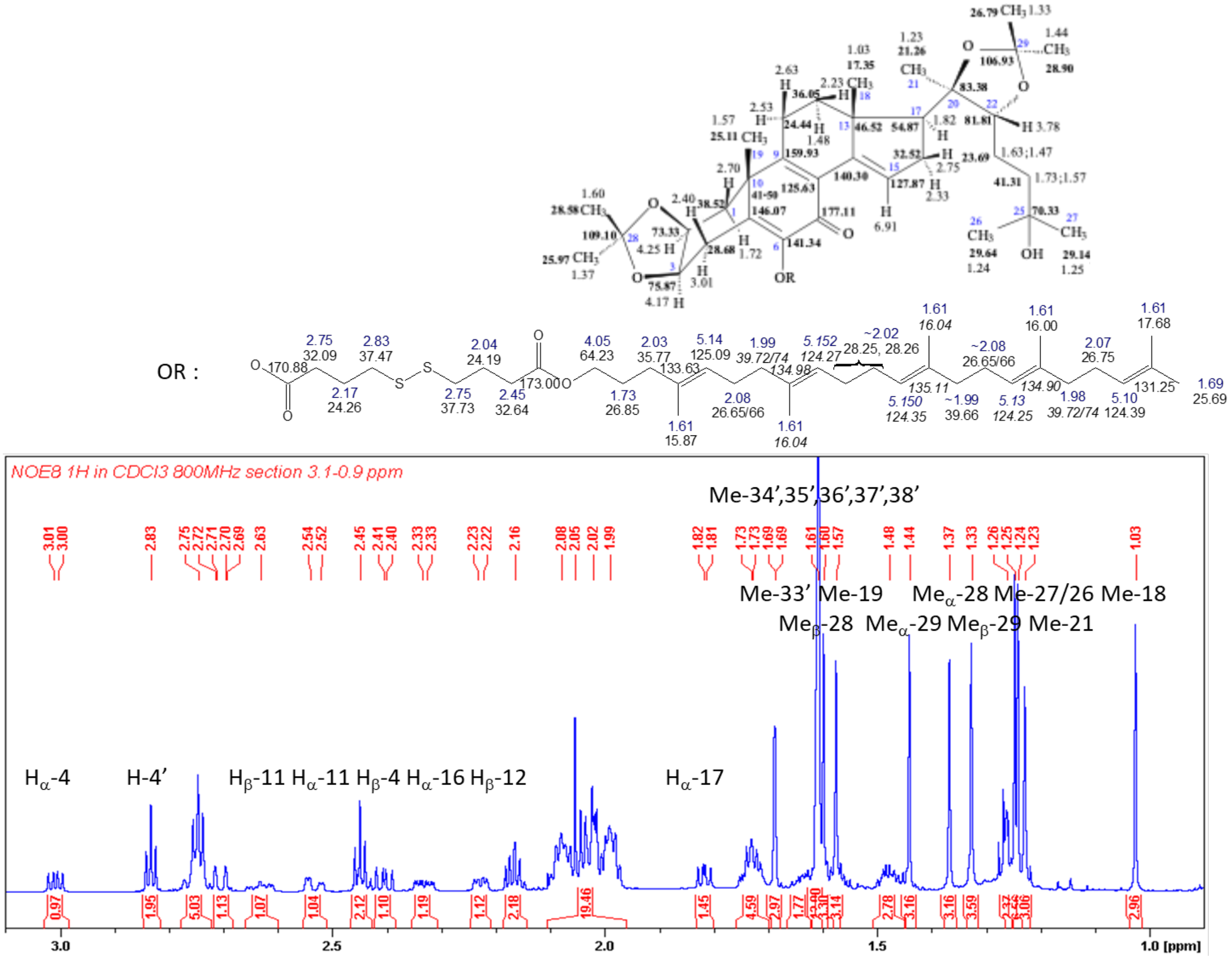

S59. Compound **18** ^{1}H NMR + selTOCSY at 4.05; 1.69 and 2.83 ppm

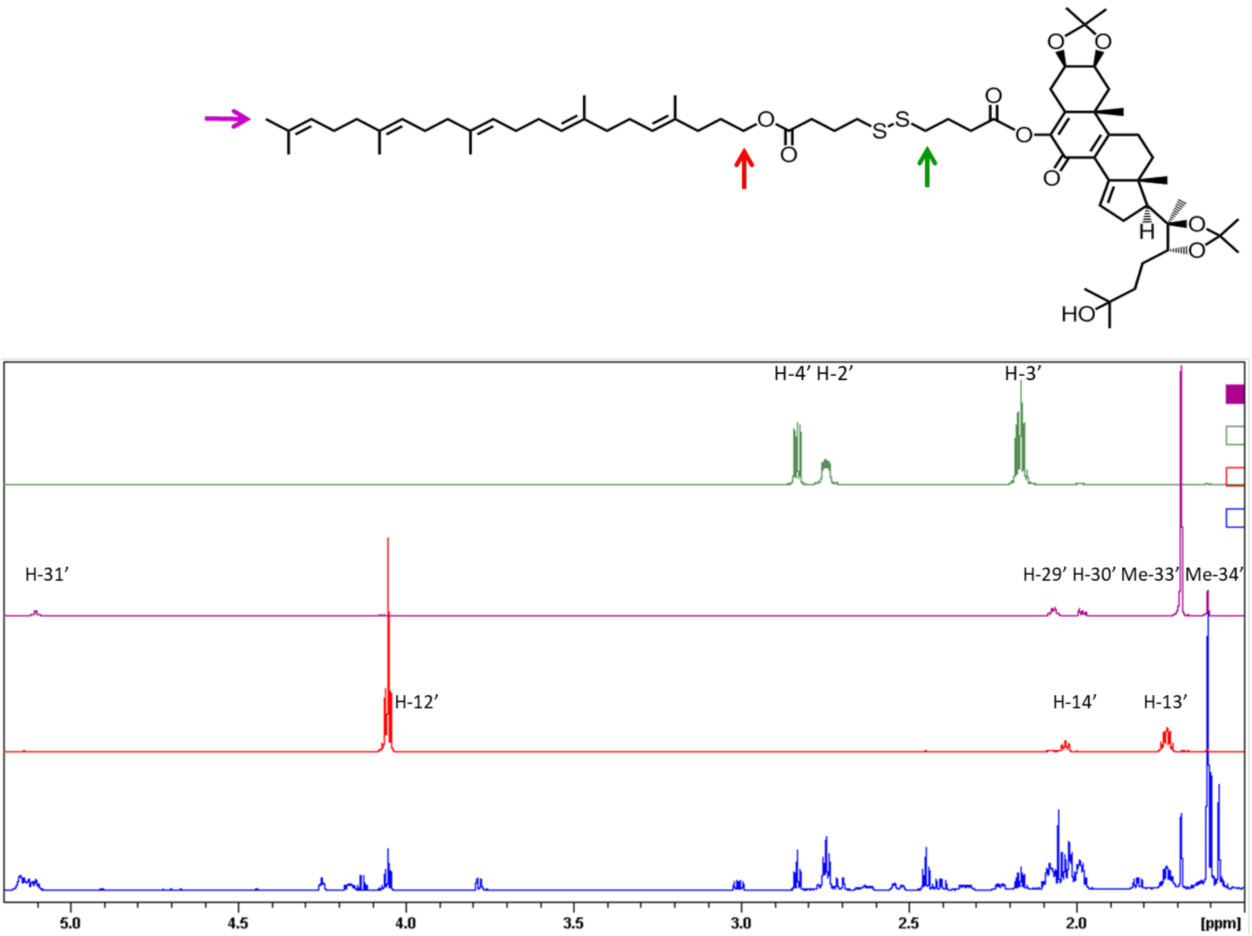

S60. Compound **18** ^{13}C DEPTQ

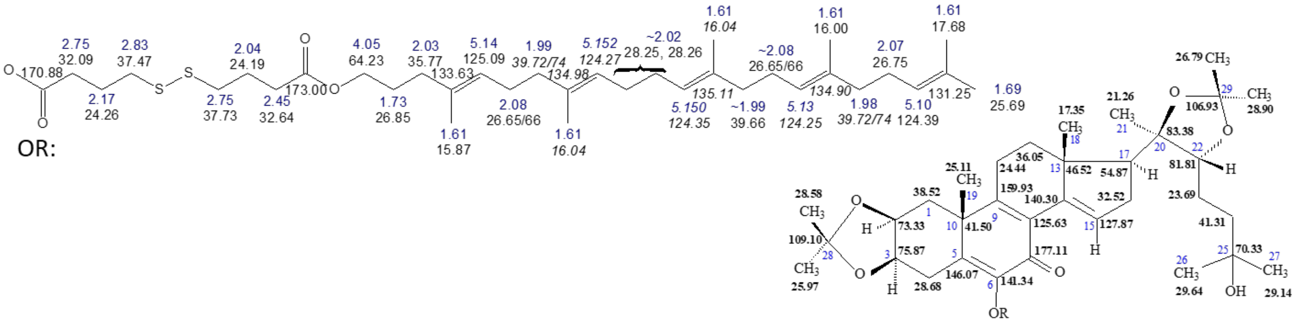

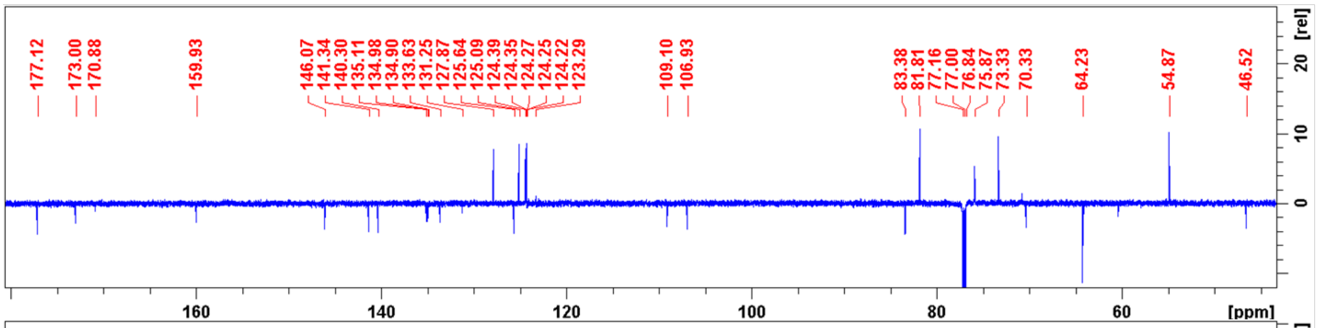

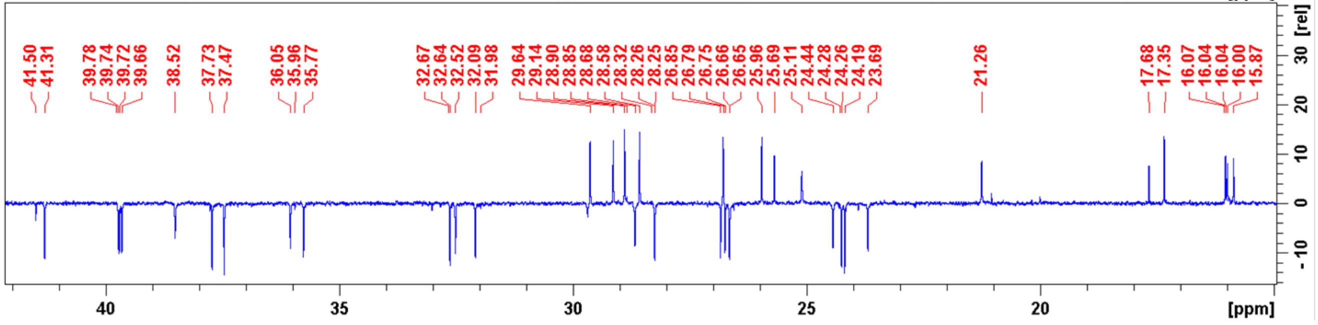

S61. Compound **18** edHSQC sections + band sel. HSQC

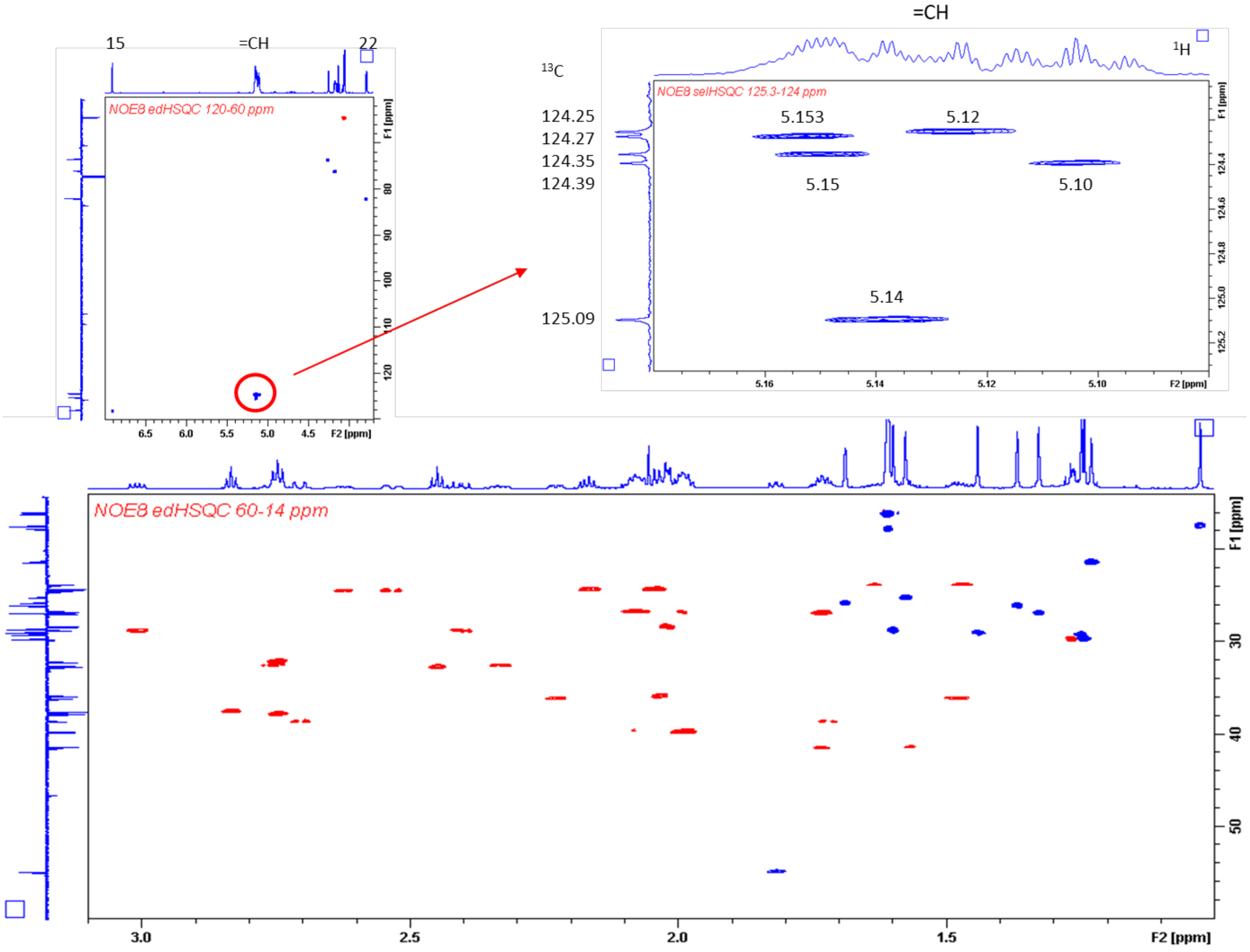

S62. Compound **18** HMBC + band sel. HMBC assignment of C-4’ and C-7’

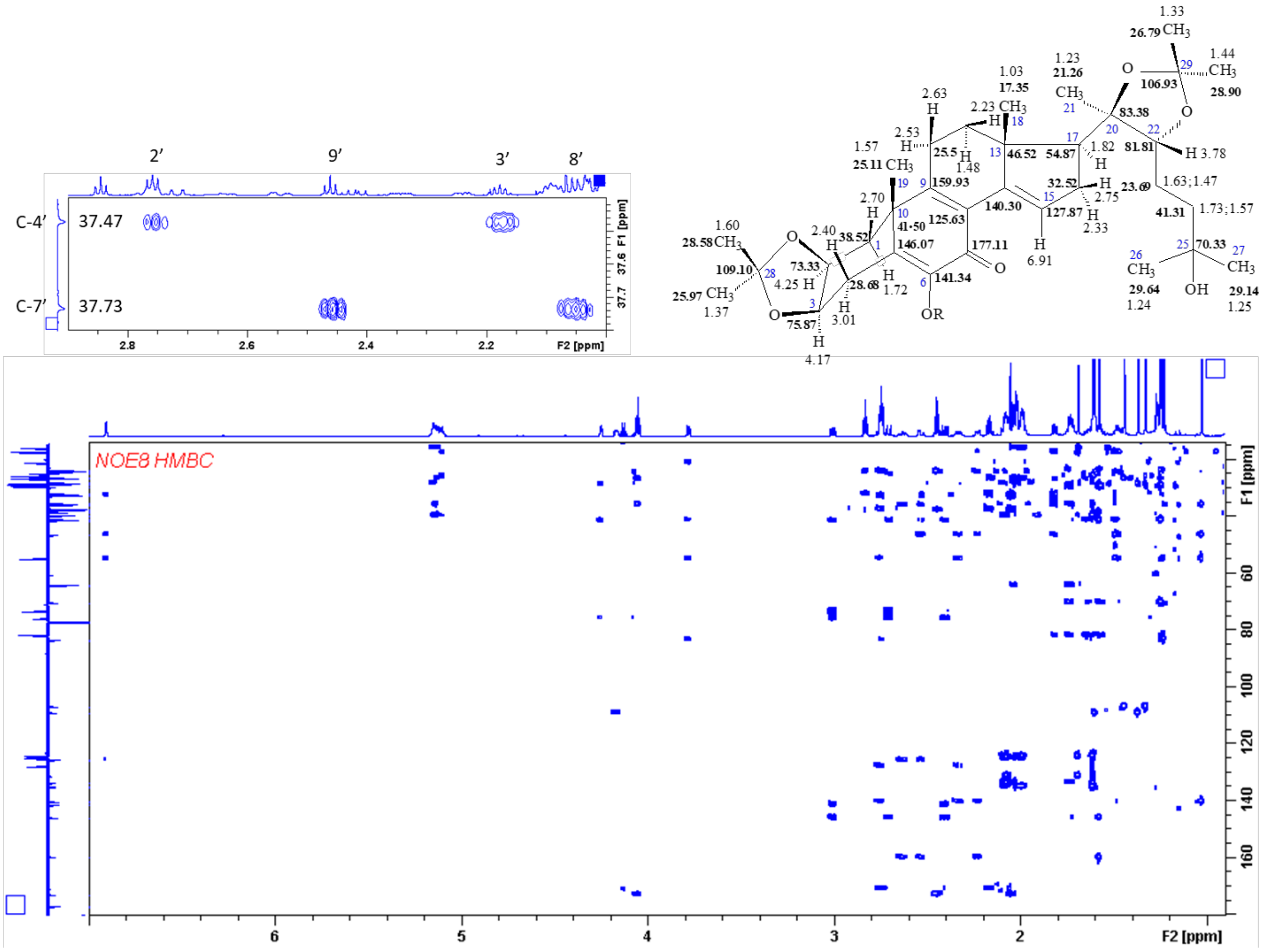

S63. Compound **20** ^{1}H NMR

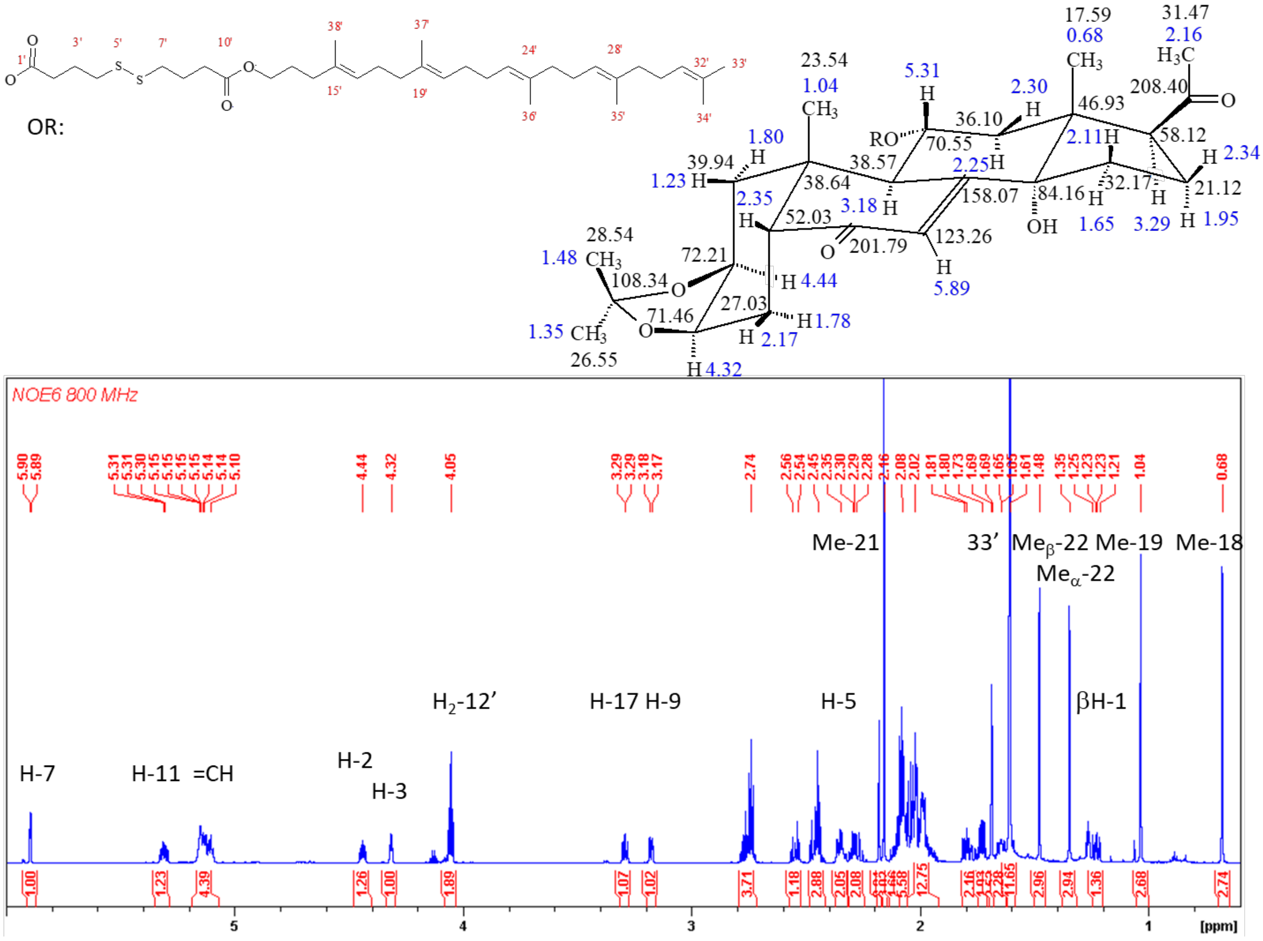

S64. Compound **20** ^{1}H NMR + selTOCSY on (4.05/1.69/2.55) t_{mix}=120ms

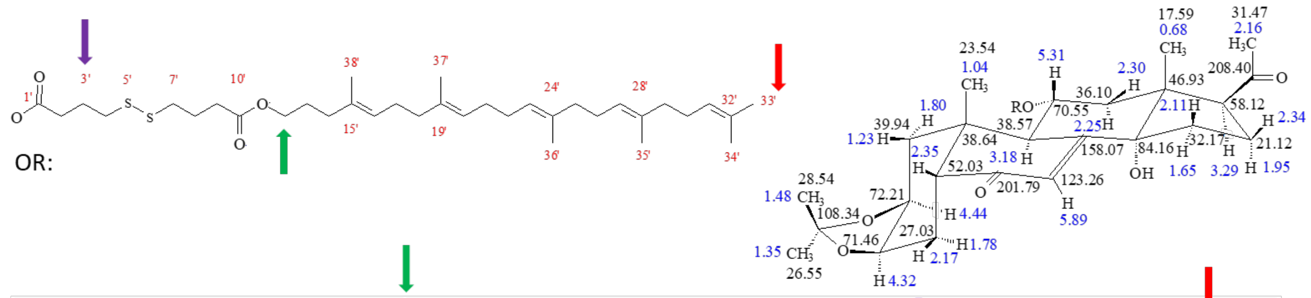

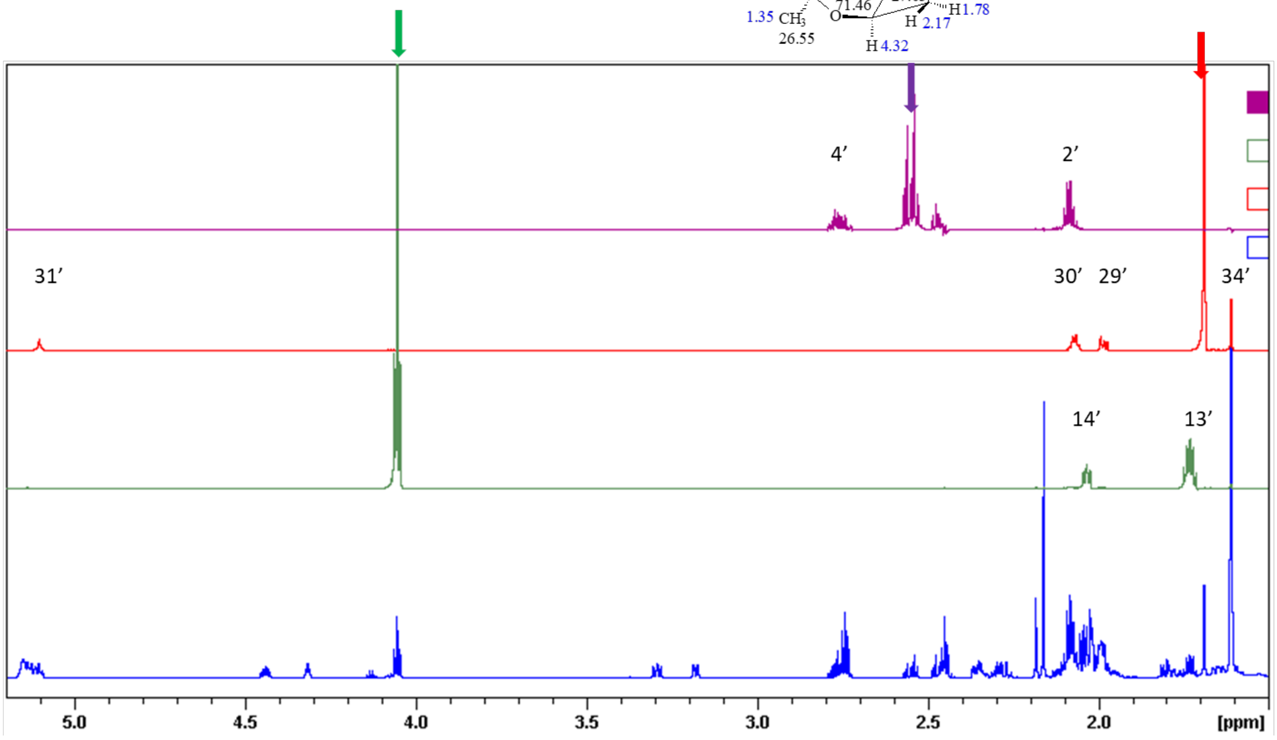

S65. Compound **20** ^{13}C NMR

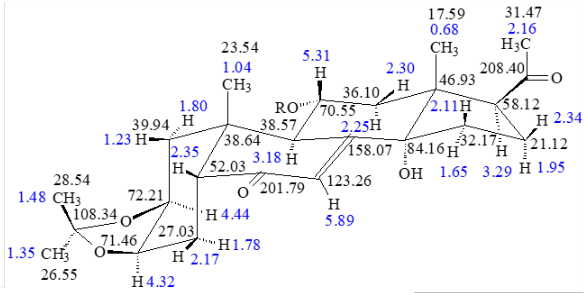

OR:

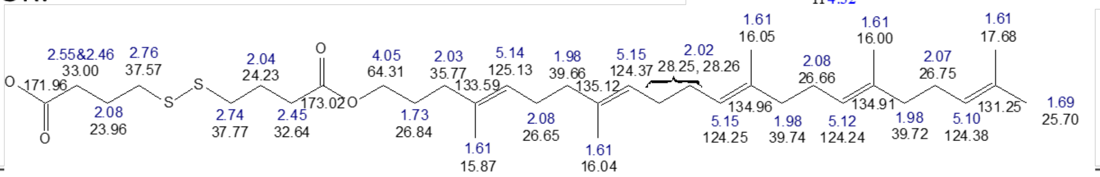

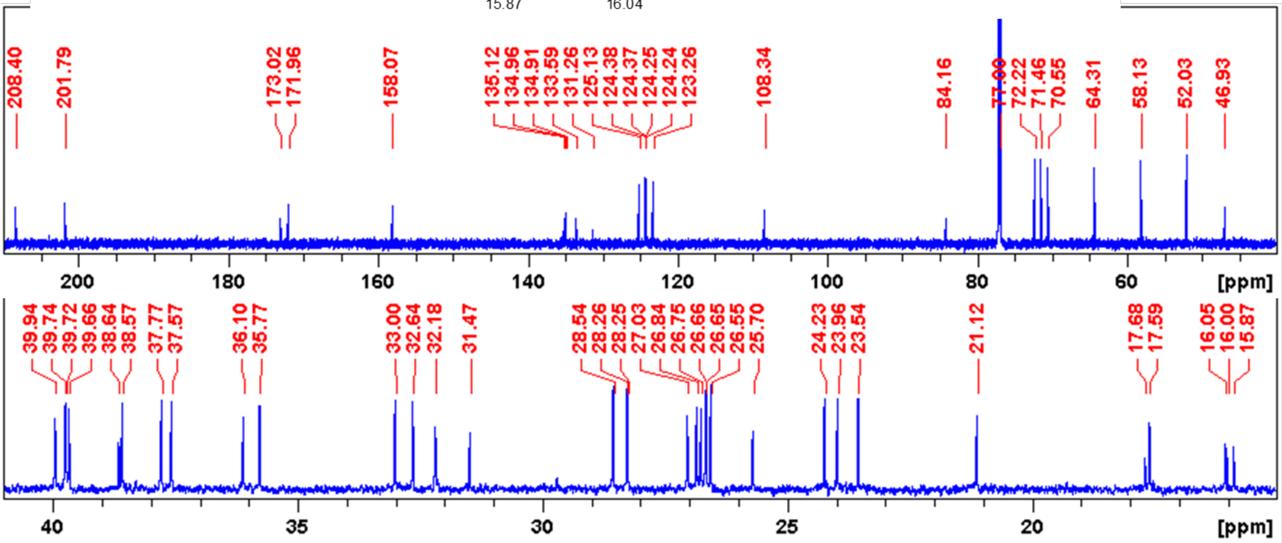

S66. Compound **20** ^{13}C DEPTQ

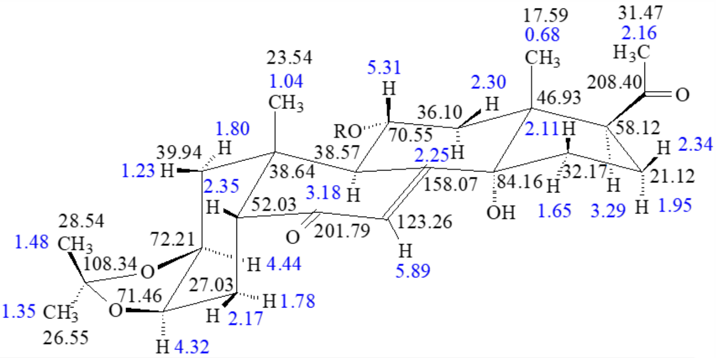

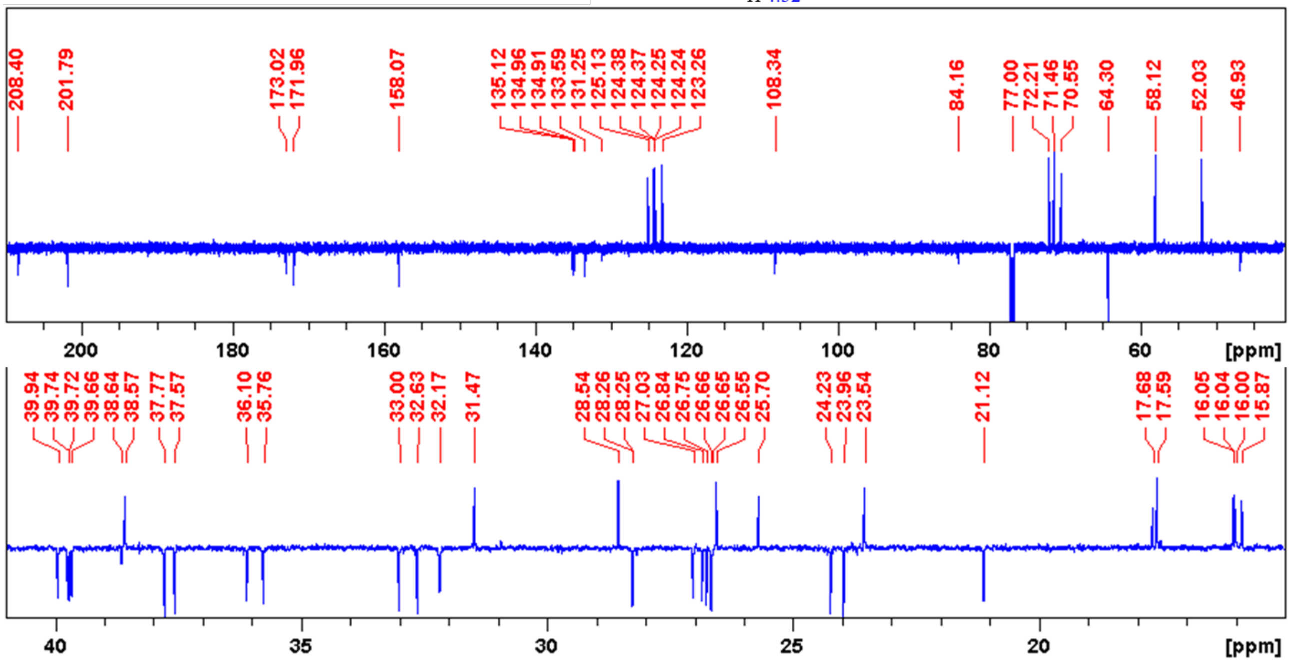

S67. Compound **20** edHSQC+band sel. HSQC =CH section

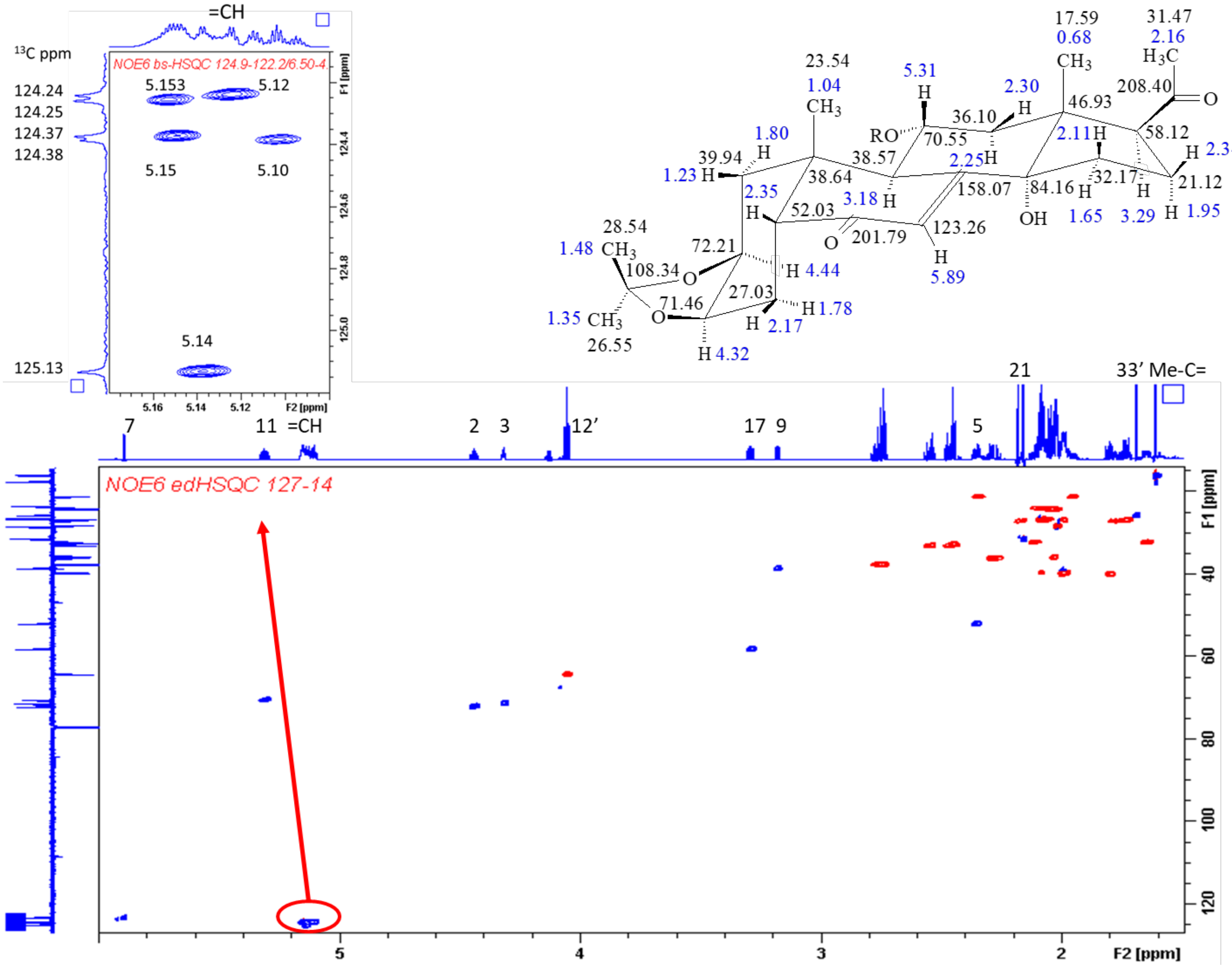

S68. Compound **20** band sel. HSQC section 3.4-0.6/40.5-15 ppm

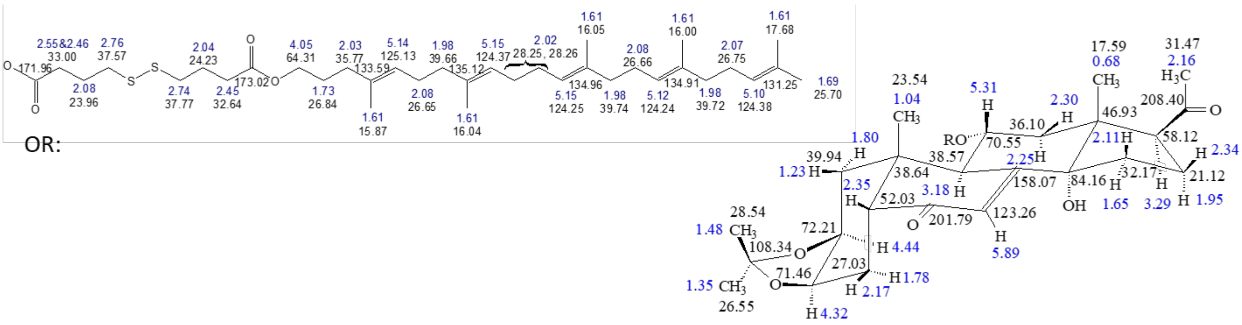

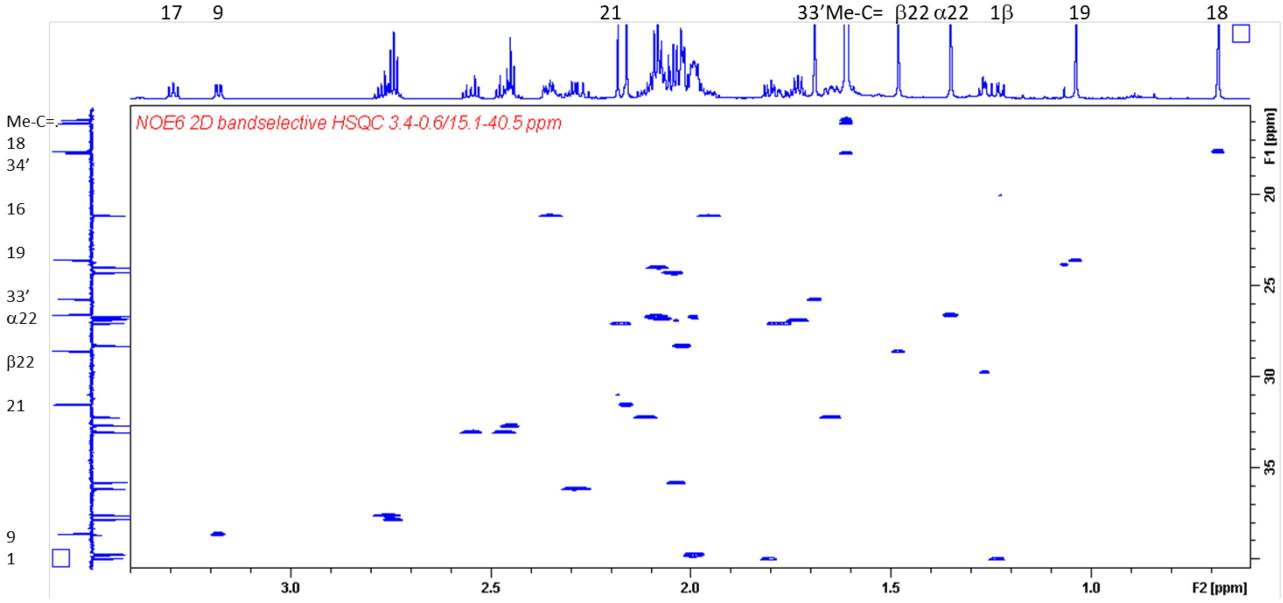

S69. Compound **20** HMBC

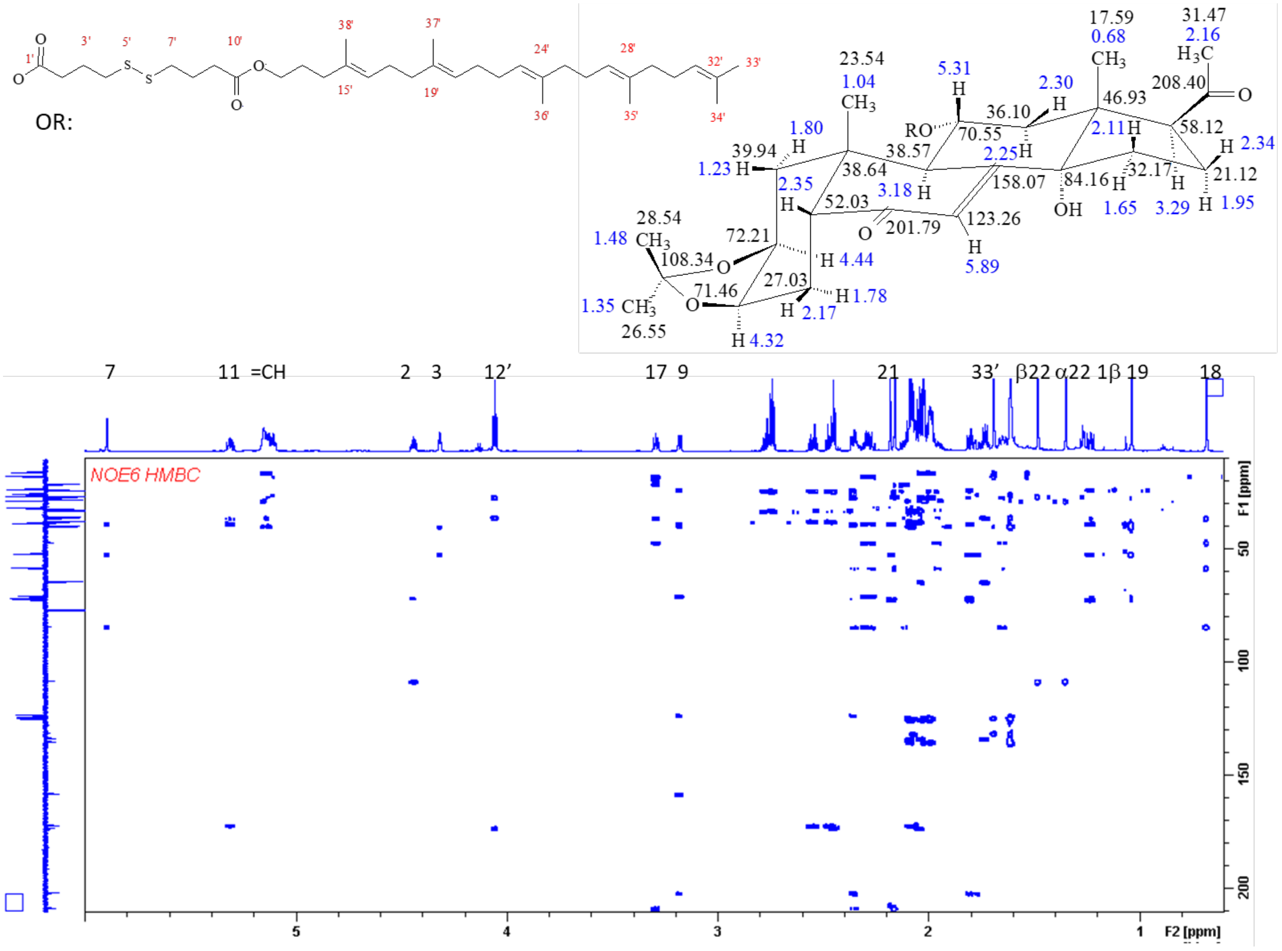

S70. Compound **20** band sel. HMBC 27.2–25.6 and 17.8–15.6 ppm sections

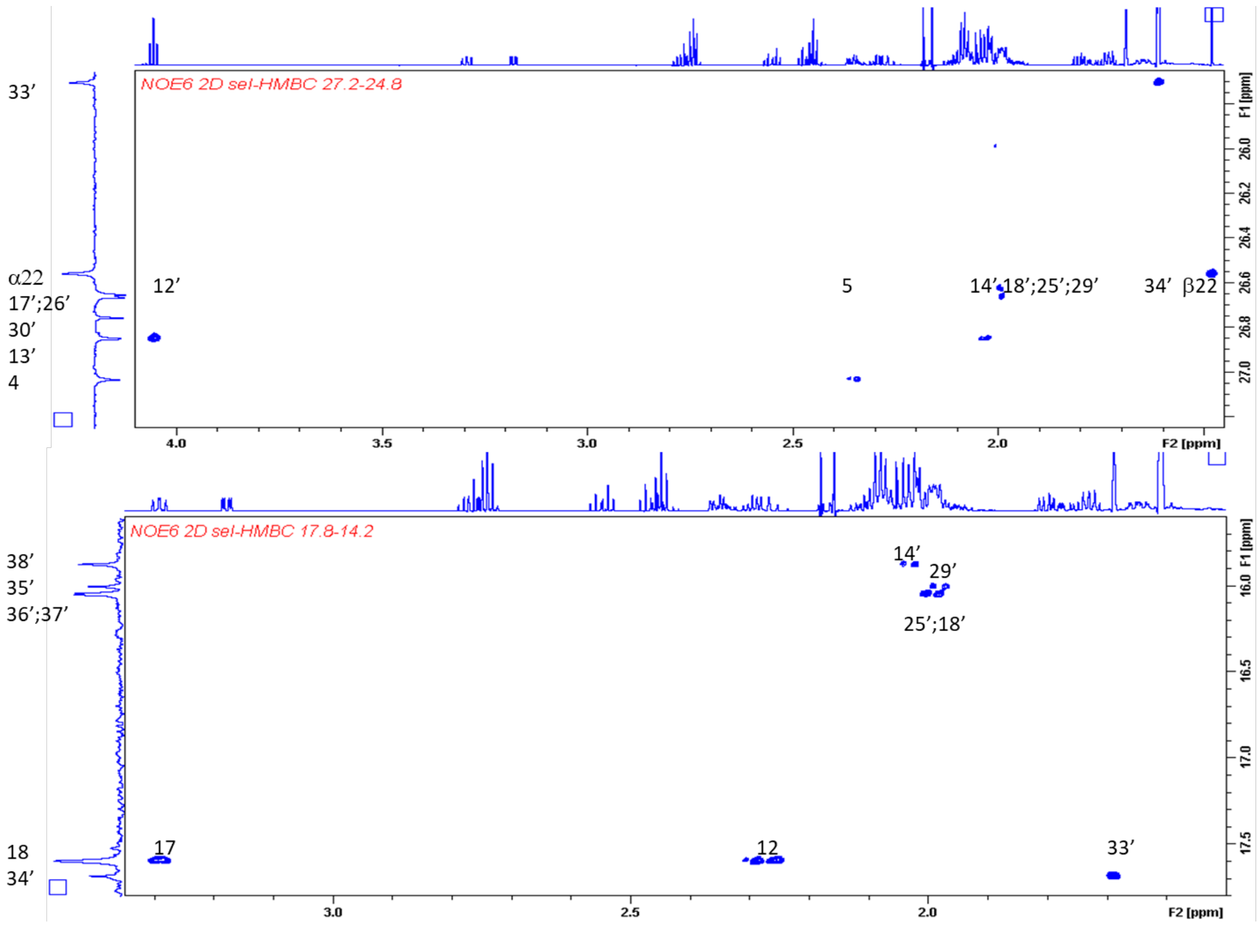

S71. Compound **20** band sel. HMBC 41.6–36.2 and 136–121 ppm sections

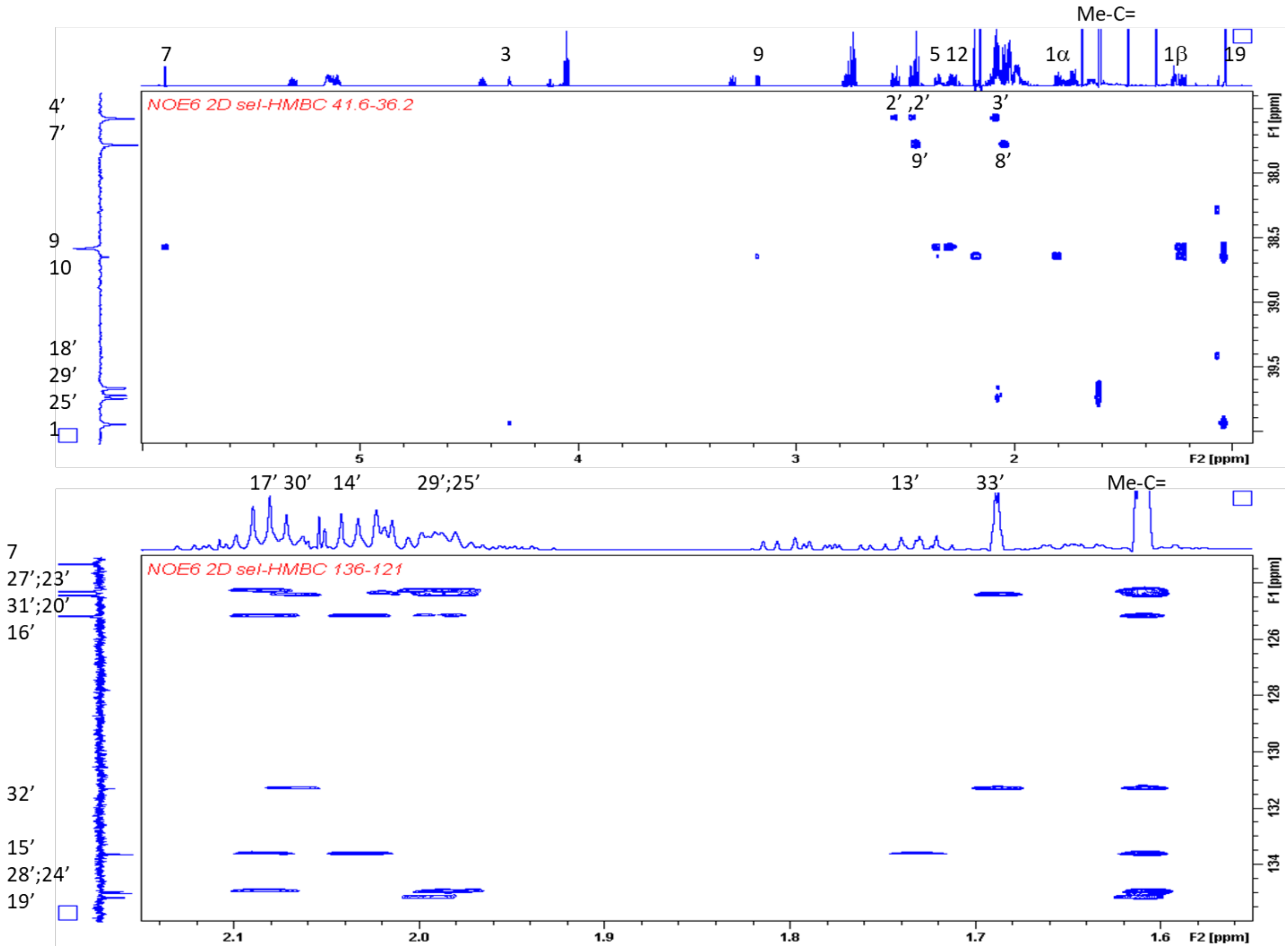

S72. Flash chromatographic, HPLC, and SFC methods for the isolation of compounds. „X g silica": RediSep NP-silica flash columns (TELEDYNE Isco, Lincoln, NE, USA). "XB-C18": Kinetex®, 5 µm, XB-C18, 100 Å, 250 mm x 21.2 mm HPLC column (Phenomenex Inc., Torrance, CA, USA). "Silica (2)": Luna® 5 µm, Silica (2), 100 Å, 250 mm x 21.2 mm HPLC column (Phenomenex Inc., Torrance, CA, USA). "Yield" expresses isolated yield%. Eluent compositions are given in *v/v* %.

Compound (Yield)	Column	Flow rate (ml/min)	Elution	Detection (nm)
**2** (13.7%)	220 g silica	150	*n*-hexane: ethyl acetate (A:B); 20→50% B (30 min)	210
**5** (24.3%)	80 g silica	60	CH_2Cl_2:ethyl-acetate (A:B); 7→10% B (10 min) - 10% B (45 min)	210
**6** (36.4%)	24 g silica	35	*n*-hexane:ethyl acetate (A:B); 3→10% B (40 min) – 10% B (20 min)	210
**8** (48.8%)	40 g silica	40	CH_2Cl_2:CH_3OH (A:B); 2→4% B (70 min)	254 / 300
**9** (49.8%)	40 g silica	40	CH_2Cl_2:CH_3OH (A:B); 0→1% B (10 min) - 1→10% B (25 min)	254 / 300
**10** (55.1%)	24 g silica	35	*n*-hexane:acetone (A:B); 0→15% B (15 min)	254 / 300
**12** (67.6%)	24 g silica	35	CH_3Cl_2:ethyl acetate (A:B); 2→4% B (60 min)	254
**13** (65.7%)	24 g silica	35	CH_2Cl_2:CH_3OH (A:B); 1.5→2% B (15 min) – 2→2.5% B (15 min) – 2.5% B (15 min)	254
**14** (21.8%)	Silica (2)	15	CO_2:ethanol (A:B); 15% B (30min)	254 / 300
**15** (21.3%)	Silica (2)	15	CO_2:ethanol (A:B); 7% B (30 min)	254 / 300
**16** (17.4 %)	Silica (2)	15	CO_2:ethanol (A:B); 4% B (30 min)	254 / 300
**17** (60.1%)	Silica (2)	15	CO_2:ethanol (A:B); 6% B (20 min)	254 / 300
**18** (46.4%)	Silica (2)	15	CO_2:ethanol (A:B); 5% B (20 min)	254 / 300
**19** (56.6%)	Silica (2)	15	CO_2:ethanol (A:B); 8% B (60 min)	254
**20** (72.5%)	Silica (2)	15	CO_2:ethanol (A:B); 7% B (15 min)	254
**21** (66.8%)	Silica (2)	15	CO_2:ethanol (A:B); 4% B (20 min)	254

S73. Average hydrodynamic diameter, polydispersity index, and zeta potential values for nanosuspensions containing self-assembled nanoparticles of compounds **14**–**21**. "Sample age" refers to the total uninterrupted storage time of the suspension that had passed from the occurrence of self-assembly to the time of the measurement. **: $p<0.01$, ***: $p<0.001$ by one-way ANOVA followed by Dunnett's post-hoc test (**14**–**16**) or unpaired T-test (**17**–**21**) as compared to the 2-hours result. Plots of raw light scattering data are presented in Figures SX1-SX44.

Compound	Sample age	Z-average (nm ± SD)	PdI ± SD	Zeta potential (mV ± SD)
**14**	2 h	135.5 ± 1.7	0.197 ± 0.011	-34.2 ± 1.4
	4 weeks	137.5 ± 0.2$^{n.s.}$	0.200 ± 0.007	-46.5 ± 1.8
	16 weeks	138.6 ± 0.5$^{n.s.}$	0.200 ± 0.007	-45.8 ± 2.0
**15**	2 h	156.0 ± 1.4	0.083 ± 0.019	-34.2 ± 0.4
	4 weeks	157.6 ± 0.3$^{n.s.}$	0.068 ± 0.025	-44.6 ± 0.8
	16 weeks	158.9 ± 0.2$^{n.s.}$	0.082 ± 0.002	-42.1 ± 0.9
**16**	2 h	187.8 ± 1.9	0.163 ± 0.007	-42.6 ± 2.2
	4 weeks	190.3 ± 1.4$^{n.s.}$	0.154 ± 0.006	-53.7 ± 2.3
	16 weeks	185.1 ± 1.7$^{n.s.}$	0.127 ± 0.007	-51.8 ± 1.4
**17**	2 h	232.7 ± 4.9	0.254 ± 0.012	-47.6 ± 0.8
	10 weeks	232.1 ± 1.3$^{n.s.}$	0.206 ± 0.035	-35.2 ± 0.5
**18**	2 h	257.0 ± 2.5	0.270 ± 0.028	-41.4 ± 0.7
	10 weeks	232.1 ± 4.5**	0.197 ± 0.011	-34.8 ± 0.7
**19**	2 h	135.4 ± 0.7	0.069 ± 0.006	-40.4 ± 0.1
	10 weeks	140.5 ± 0.8**	0.083 ± 0.015	-31.4 ± 0.5
**20**	2 h	180.8 ± 2.0	0.150 ± 0.009	-40.8 ± 0.6
	10 weeks	172.4 ± 0.7**	0.116 ± 0.011	-32.5 ± 0.8
**21**	2 h	268.5 ± 3.0	0.270 ± 0.012	-40.9 ± 0.5
	10 weeks	228.5 ± 2.7***	0.233 ± 0.021	-28.7 ± 0.2

S74. Particle size distribution plots obtained from light scattering data of the nanosuspension of compound **14** 2 hours after nanoprecipitation. Results of 3 separate measurements are shown by intensity (left) and number (right).

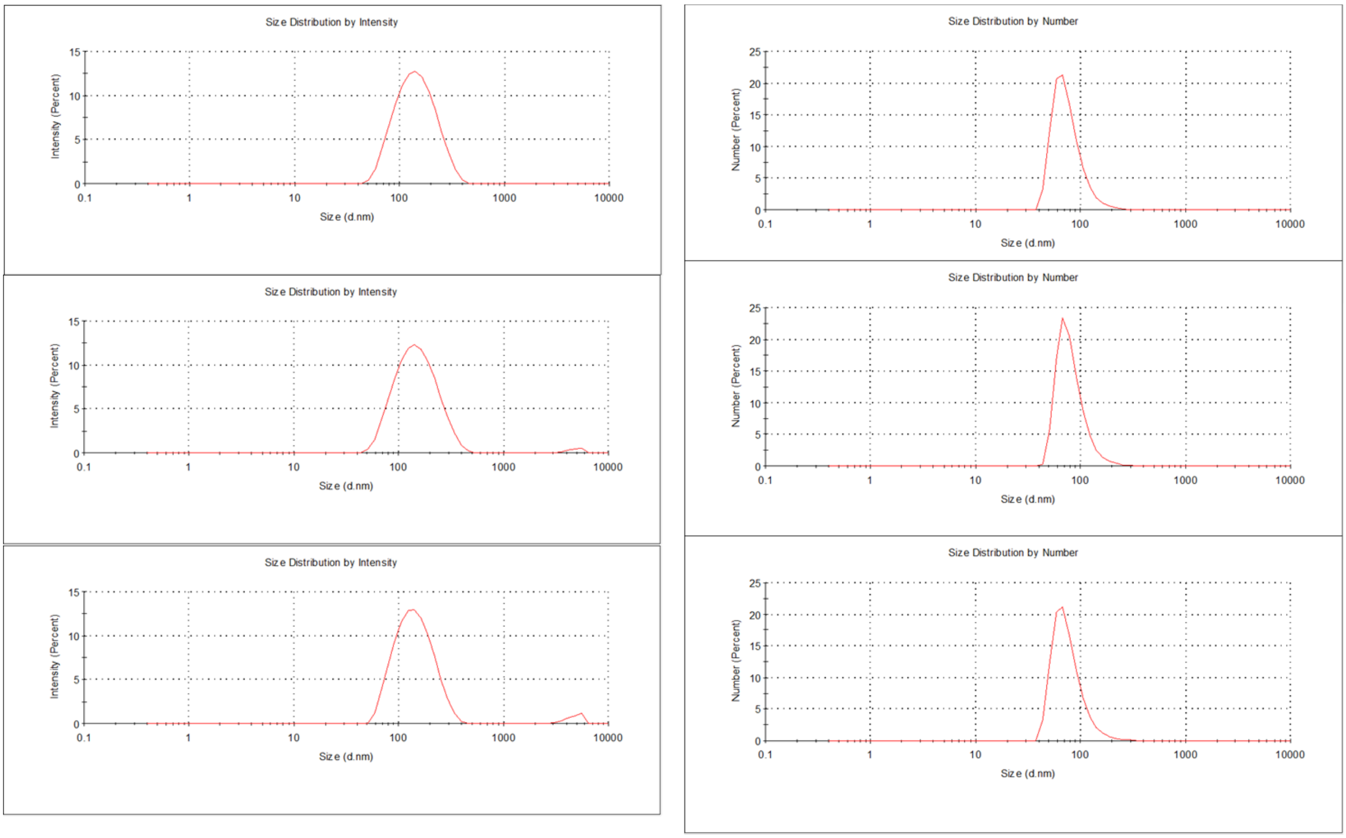

S75. Particle size distribution plots obtained from light scattering data of the nanosuspension of compound **14** 2 weeks after nanoprecipitation. Plots of 3 separate measurements are shown by intensity (left) and number (right).

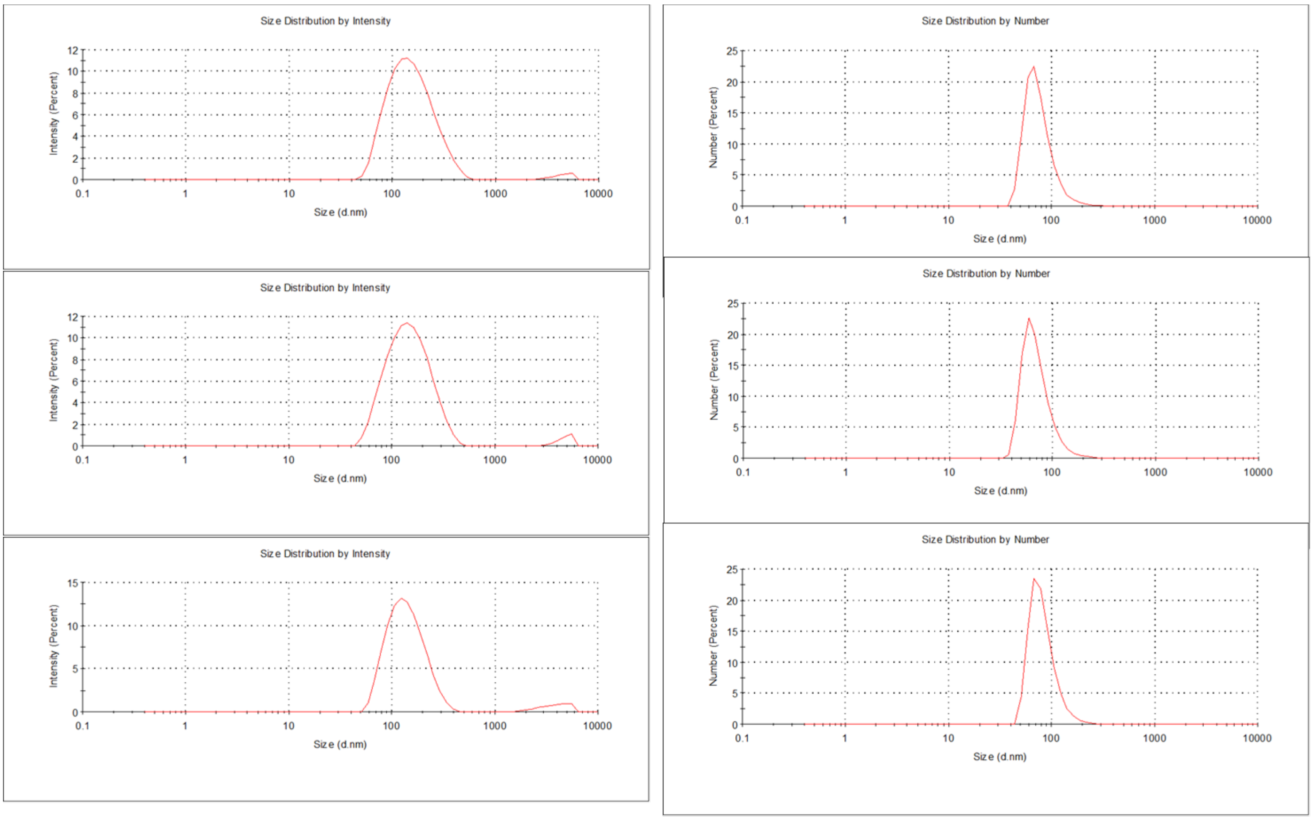

S76. Particle size distribution plots obtained from light scattering data of the nanosuspension of compound **14** 4 weeks after nanoprecipitation. Plots of 3 separate measurements are shown by intensity (left) and number (right).

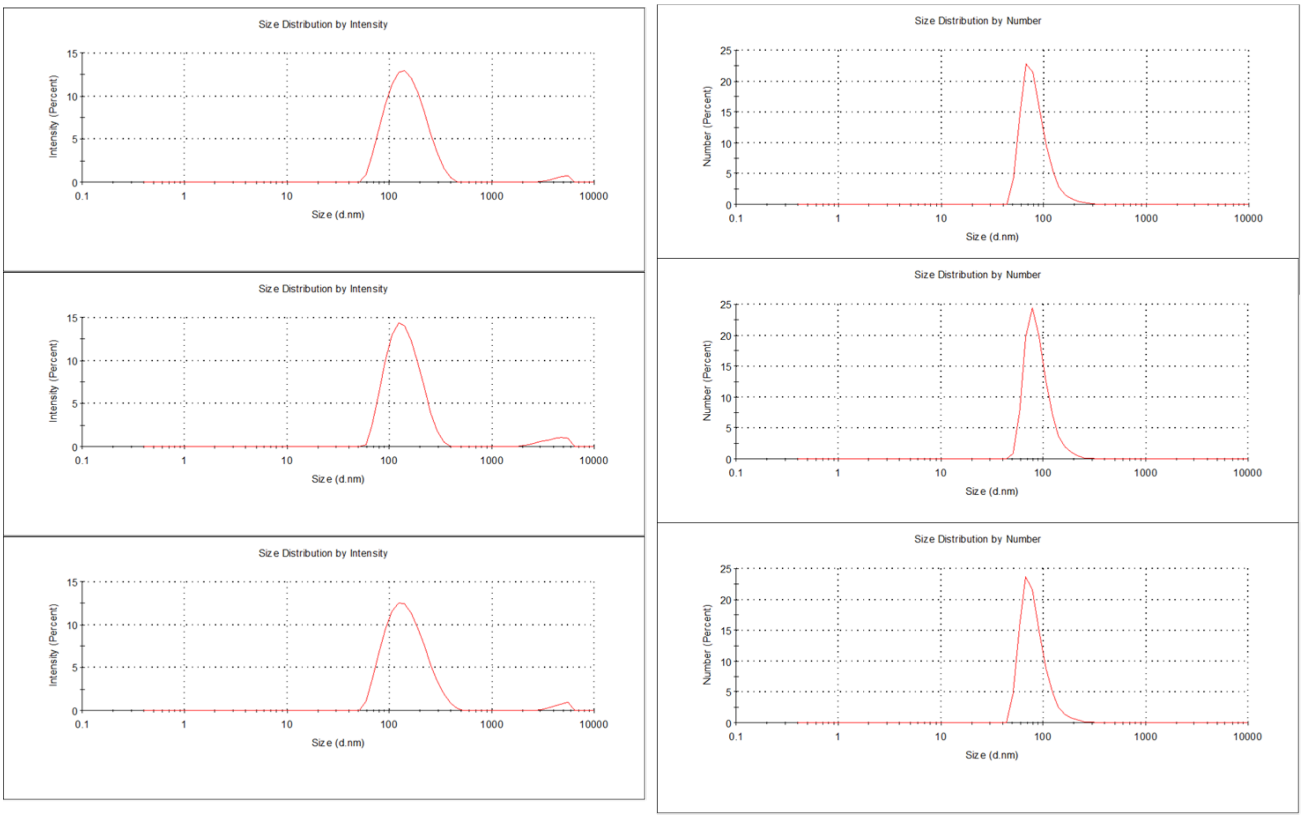

S77. Particle size distribution plots obtained from light scattering data of the nanosuspension of compound **14** 16 weeks after nanoprecipitation. Plots of 3 separate measurements are shown by intensity (left) and number (right).

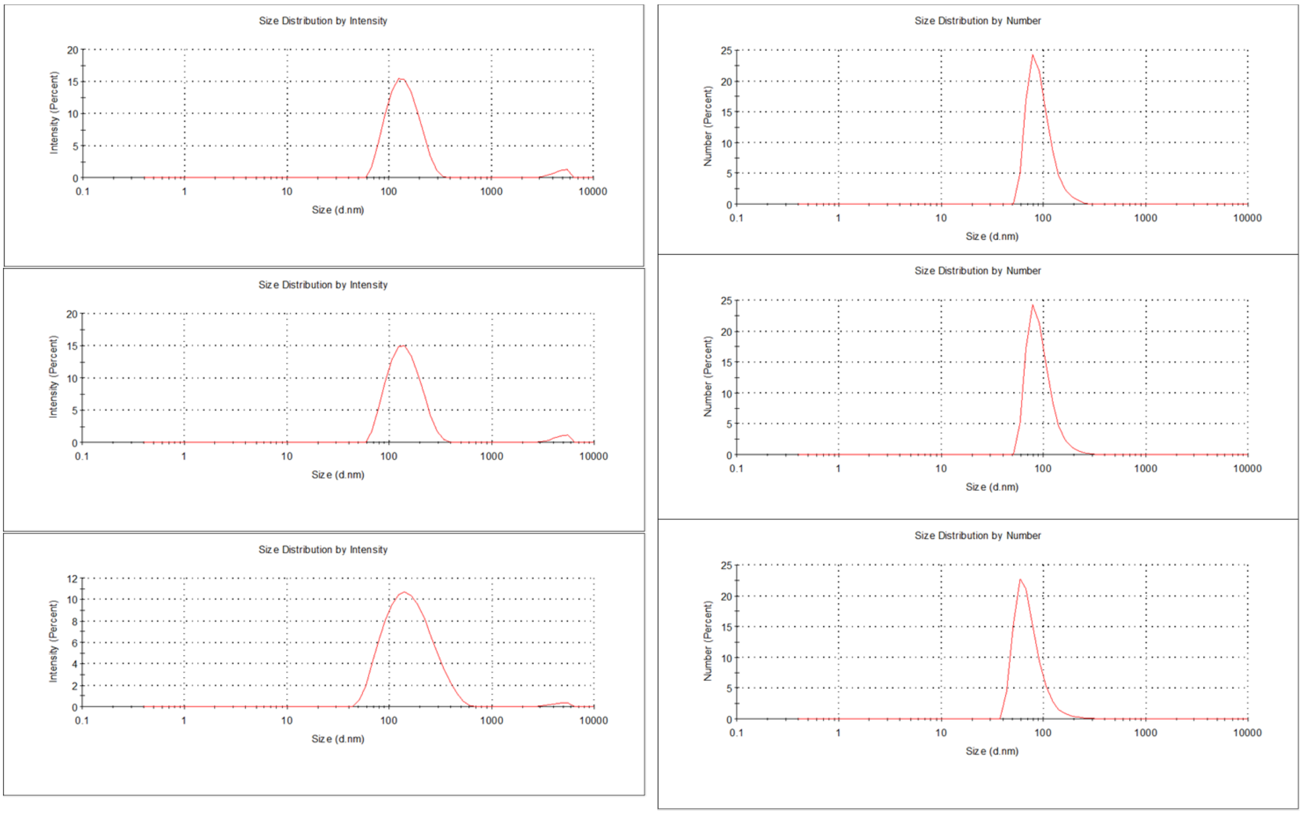

S78. Particle size distribution plots obtained from light scattering data of the nanosuspension of compound **15** 2 hours after nanoprecipitation. Plots of 3 separate measurements are shown by intensity (left) and number (right).

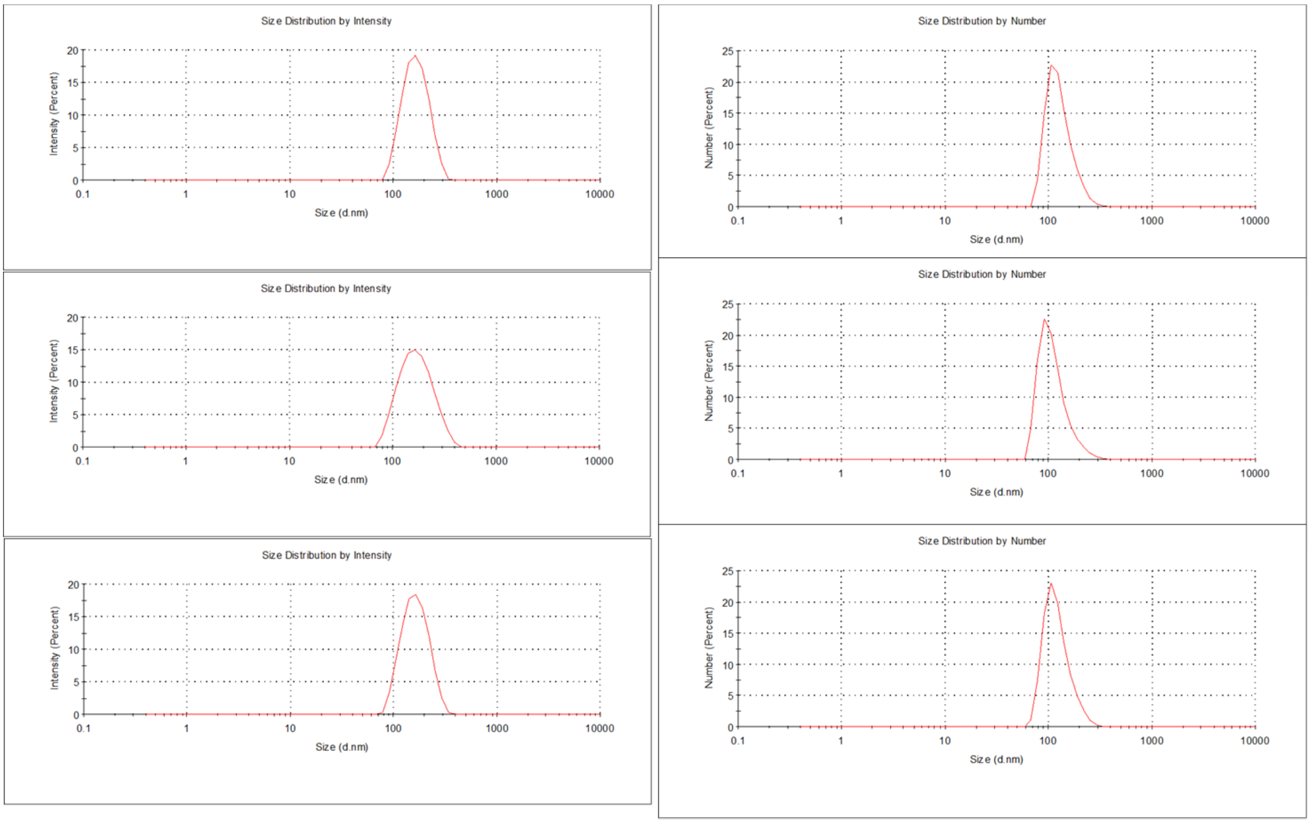

S79. Particle size distribution plots obtained from light scattering data of the nanosuspension of compound **15** 2 weeks after nanoprecipitation. Plots of 3 separate measurements are shown by intensity (left) and number (right).

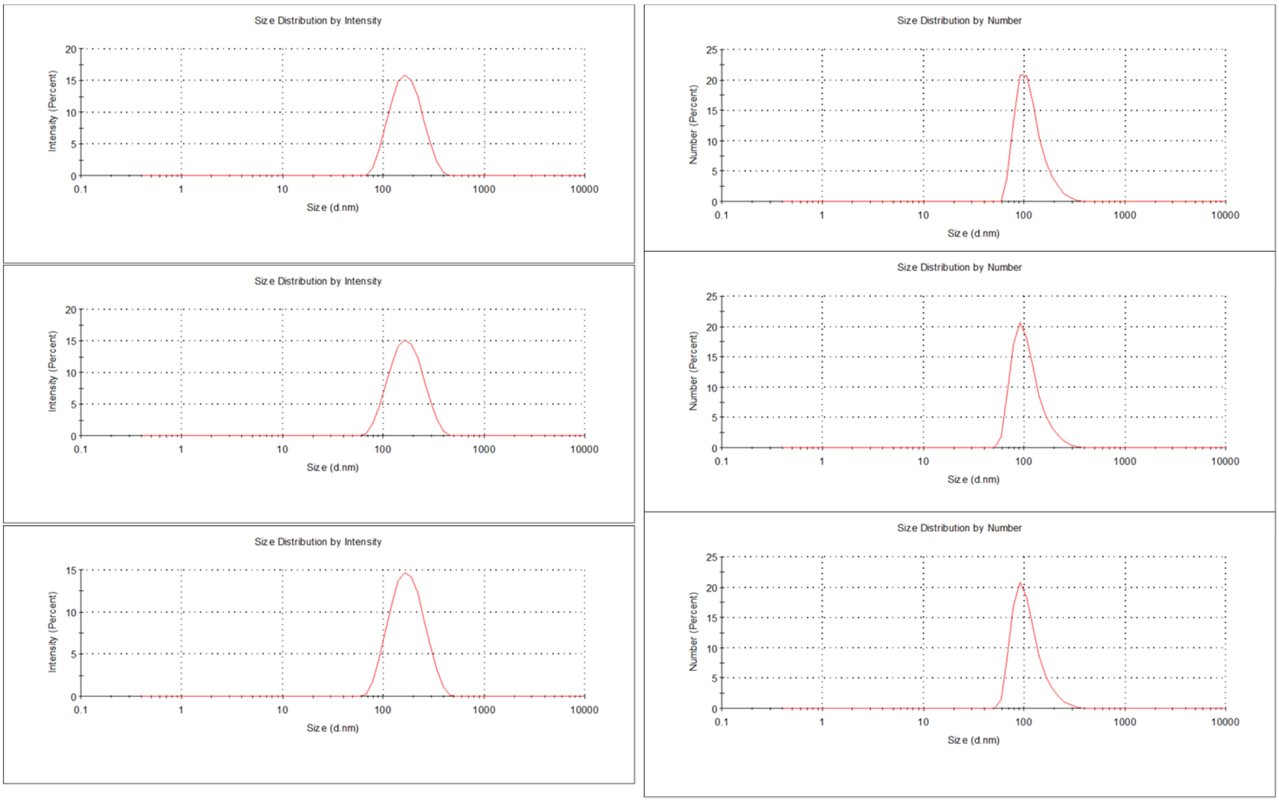

S80. Particle size distribution plots obtained from light scattering data of the nanosuspension of compound **15** 4 weeks after nanoprecipitation. Plots of 3 separate measurements are shown by intensity (left) and number (right).

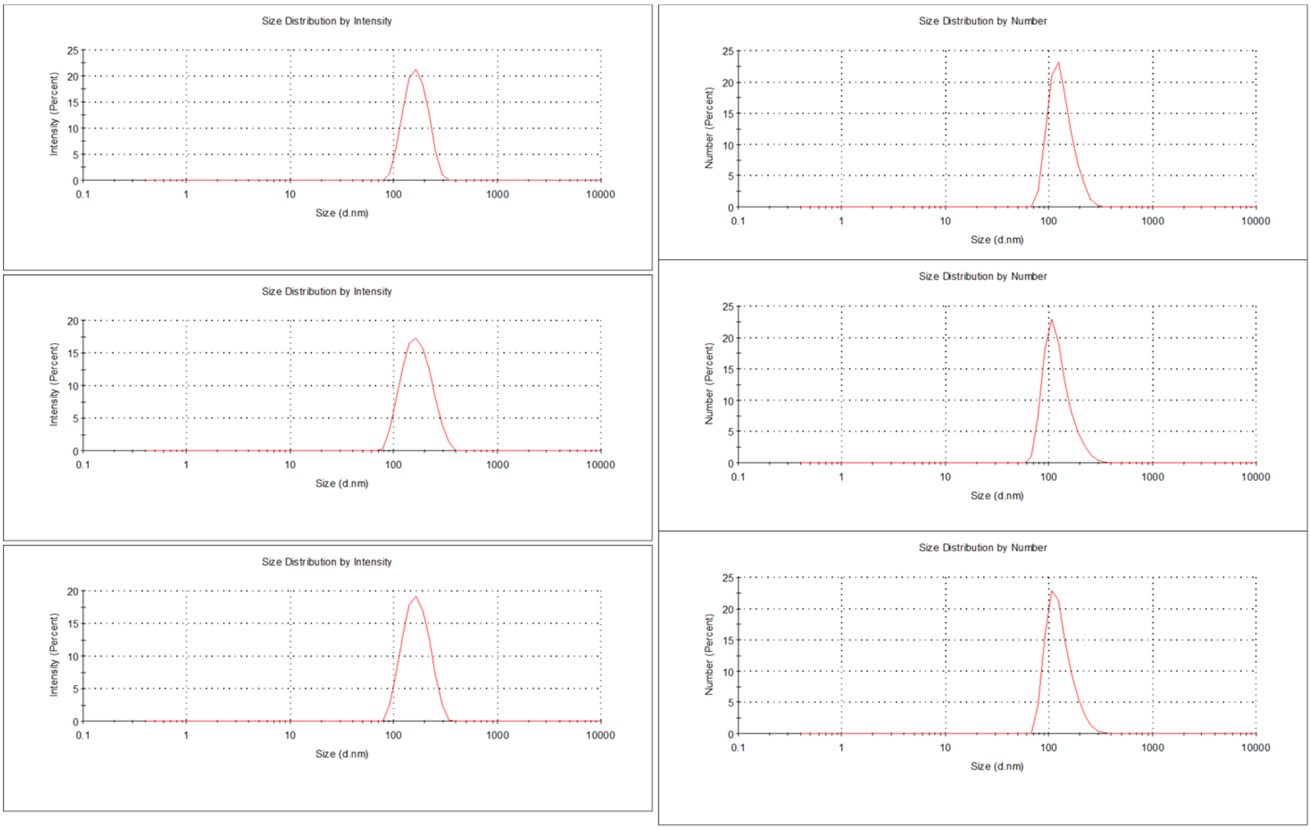

S81. Particle size distribution plots obtained from light scattering data of the nanosuspension of compound **15** 16 weeks after nanoprecipitation. Plots of 3 separate measurements are shown by intensity (left) and number (right).

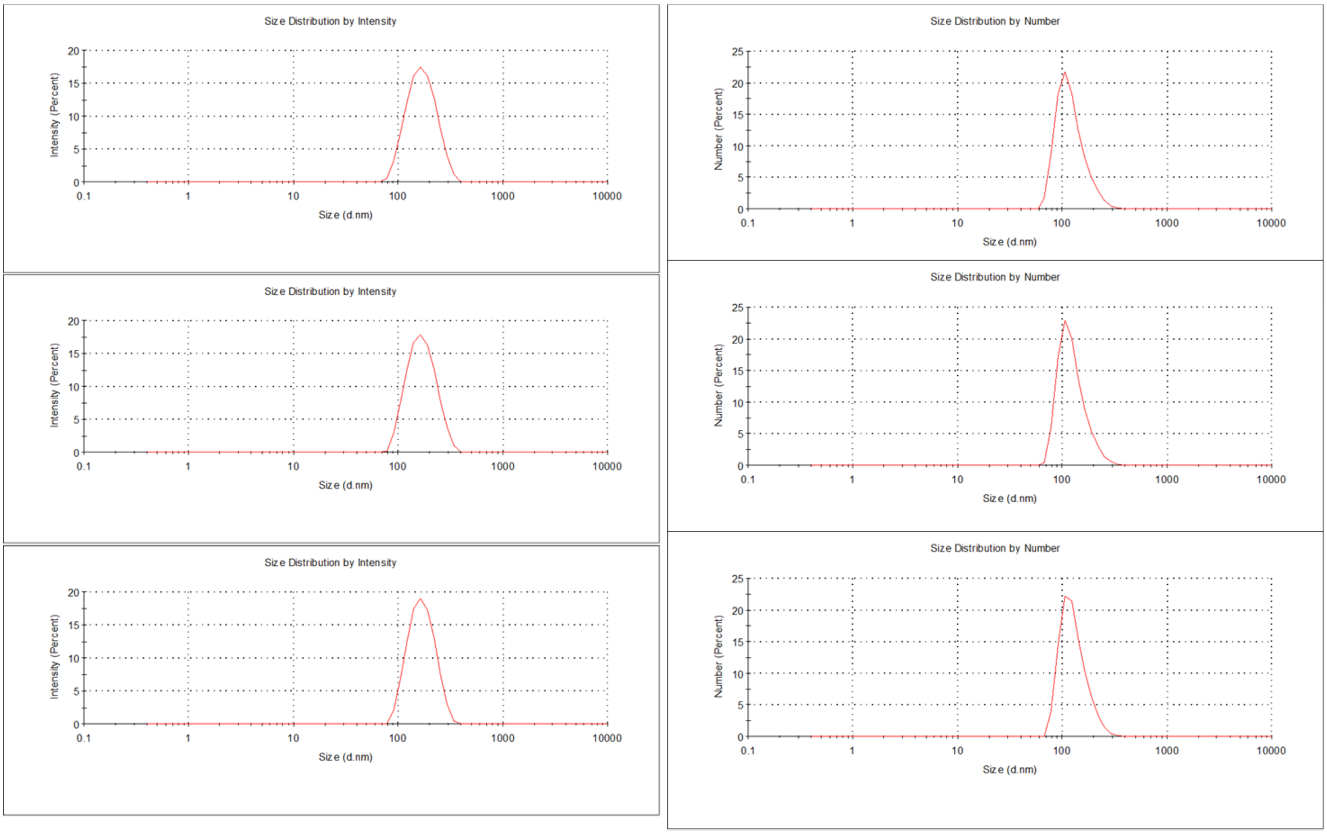

S82. Particle size distribution plots obtained from light scattering data of the nanosuspension of compound **16** 2 hours after nanoprecipitation. Plots of 3 separate measurements are shown by intensity (left) and number (right).

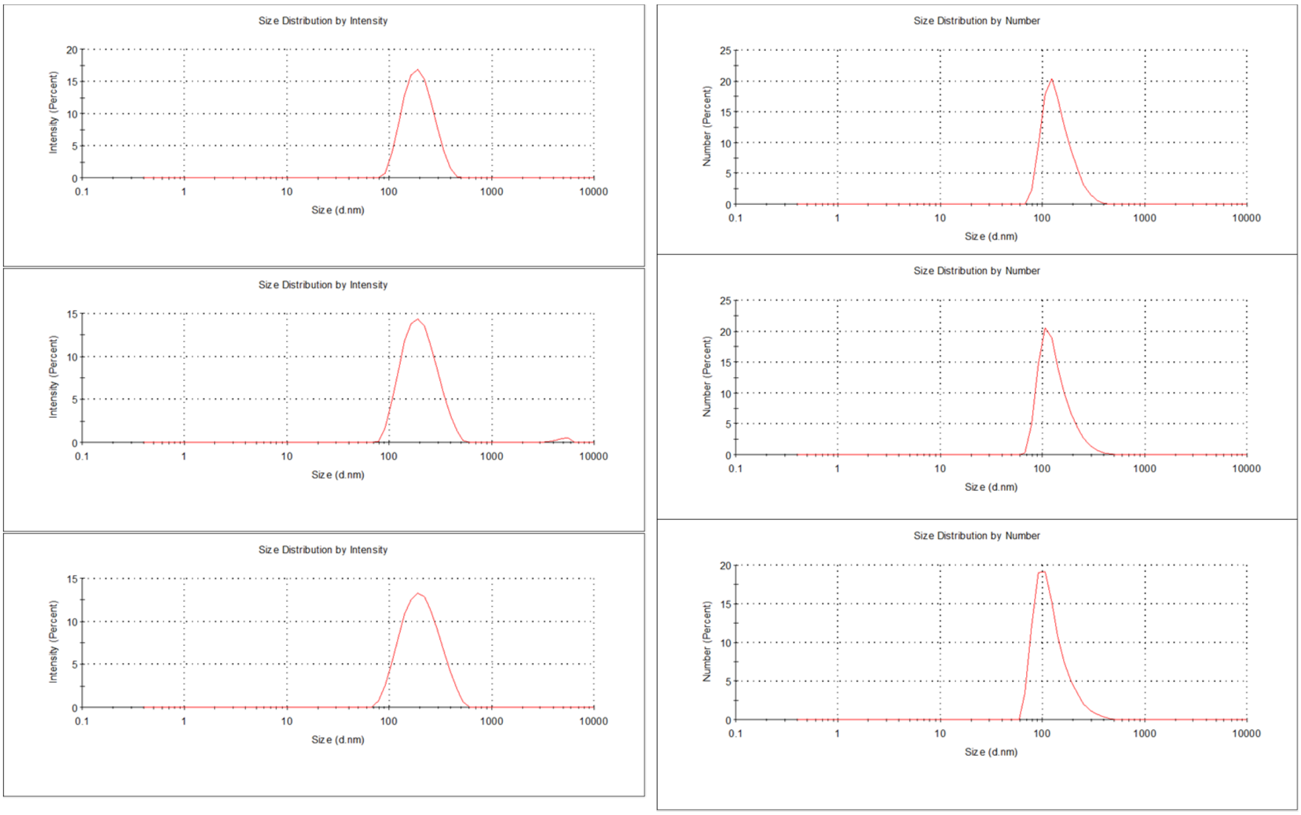

S83. Particle size distribution plots obtained from light scattering data of the nanosuspension of compound **16** 2 weeks after nanoprecipitation. Plots of 3 separate measurements are shown by intensity (left) and number (right).

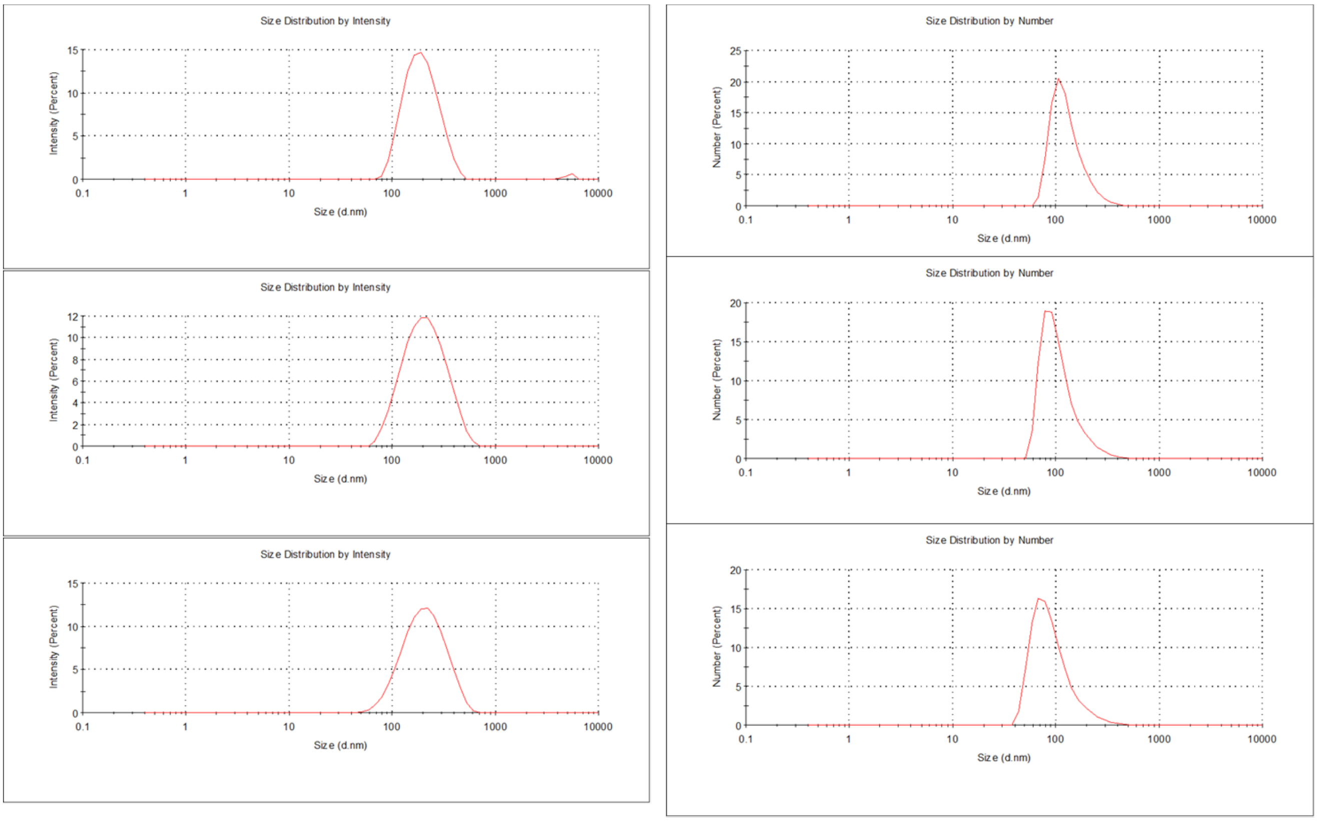

S84. Particle size distribution plots obtained from light scattering data of the nanosuspension of compound **16** 4 weeks after nanoprecipitation. Plots of 3 separate measurements are shown by intensity (left) and number (right).

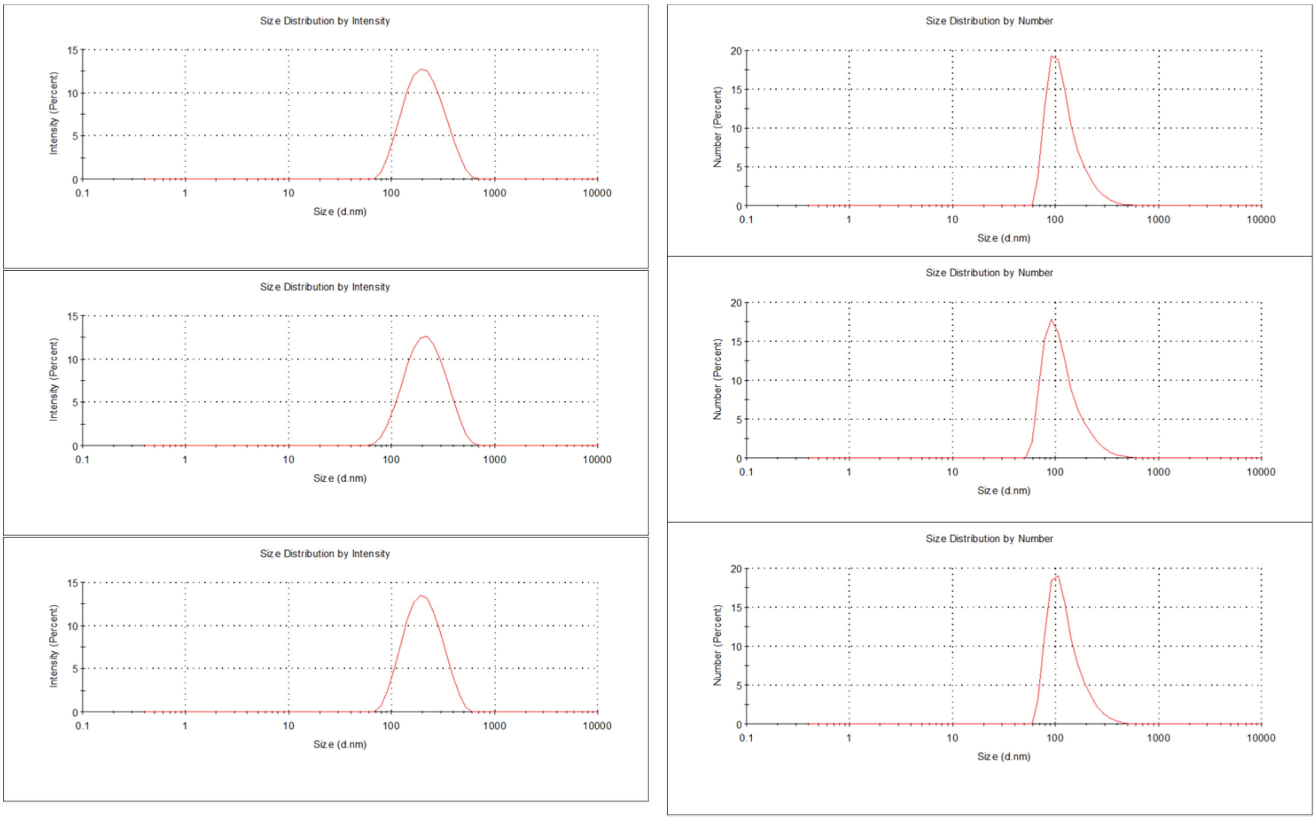

S85. Particle size distribution plots obtained from light scattering data of the nanosuspension of compound **16** 16 weeks after nanoprecipitation. Plots of 3 separate measurements are shown by intensity (left) and number (right).

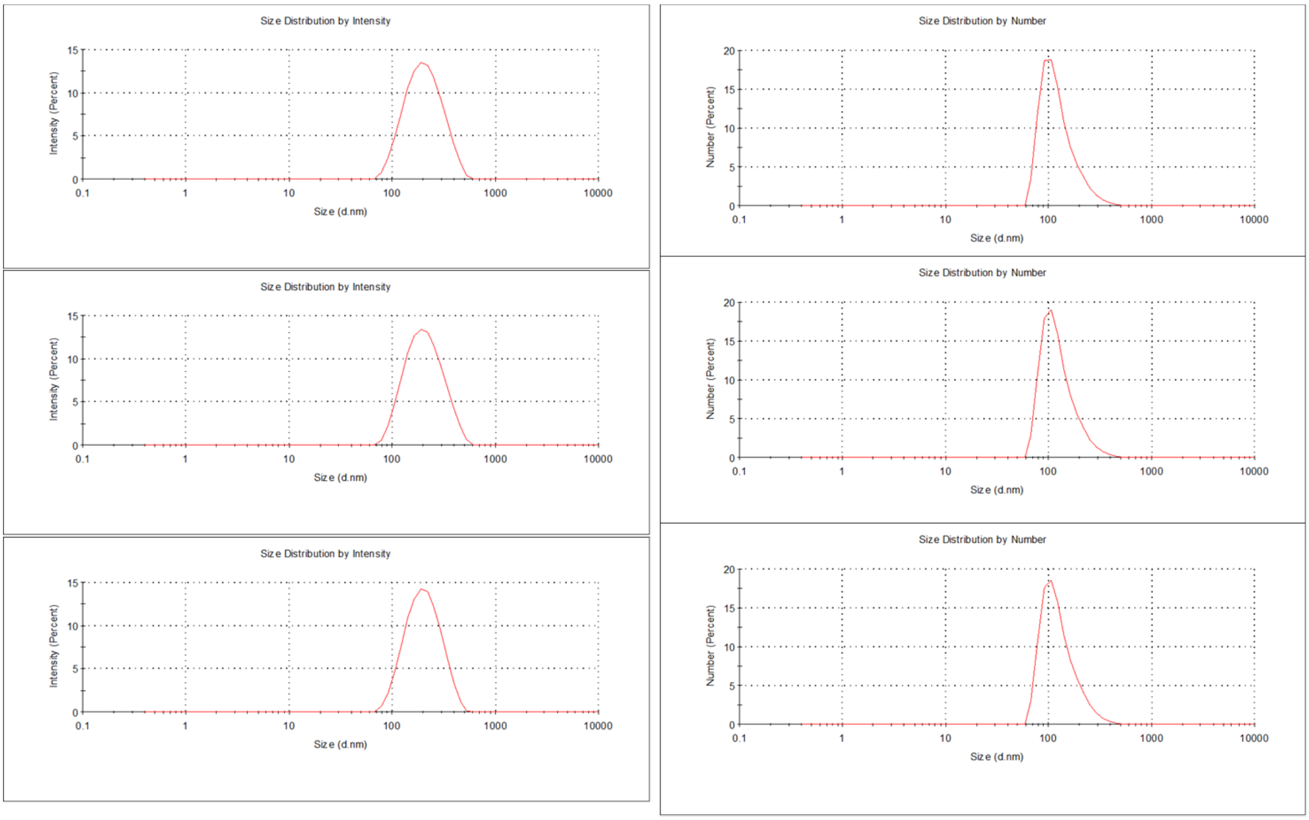

S86. Particle size distribution plots obtained from light scattering data of the nanosuspension of compound **17** 2 hours after nanoprecipitation. Plots of 3 separate measurements are shown by intensity (left) and number (right).

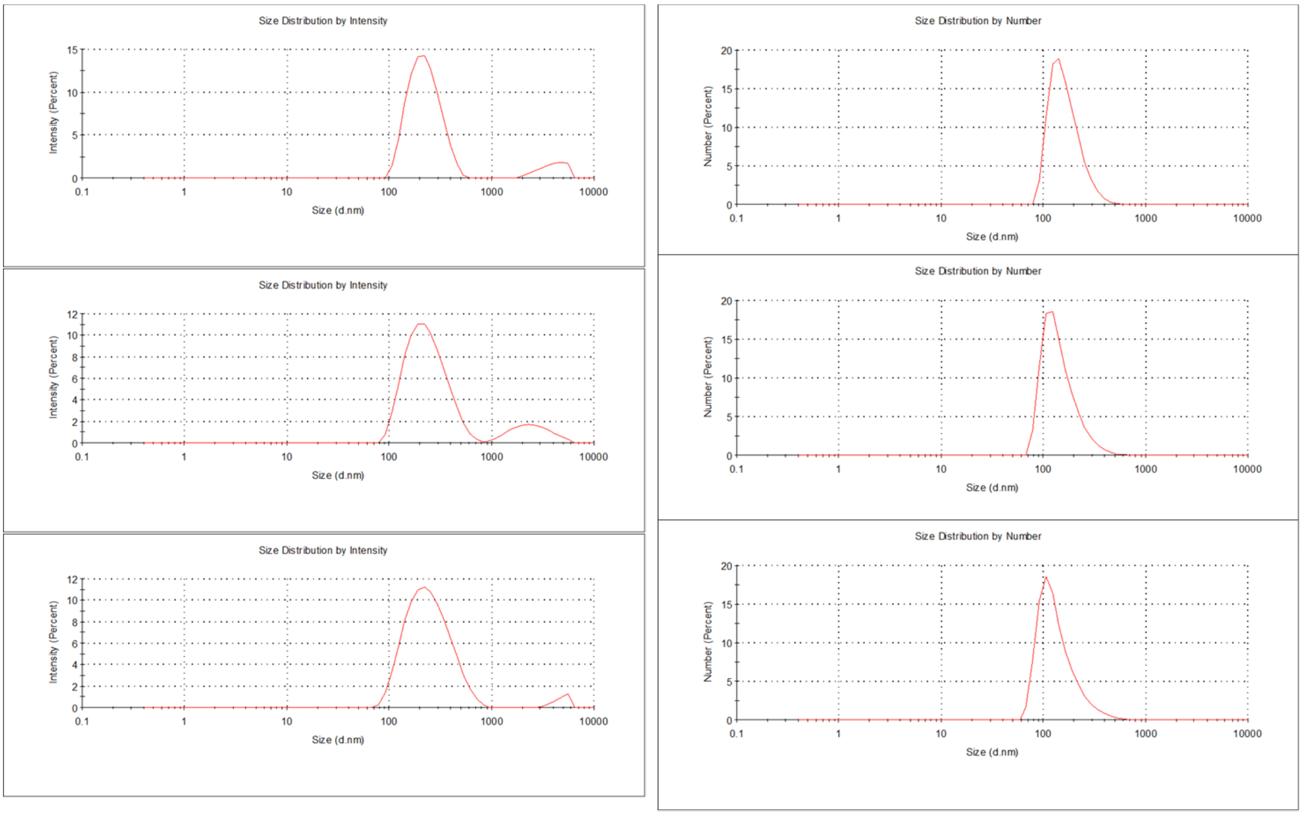

S87. Particle size distribution plots obtained from light scattering data of the nanosuspension of compound **17** 10 weeks after nanoprecipitation. Plots of 3 separate measurements are shown by intensity (left) and number (right).

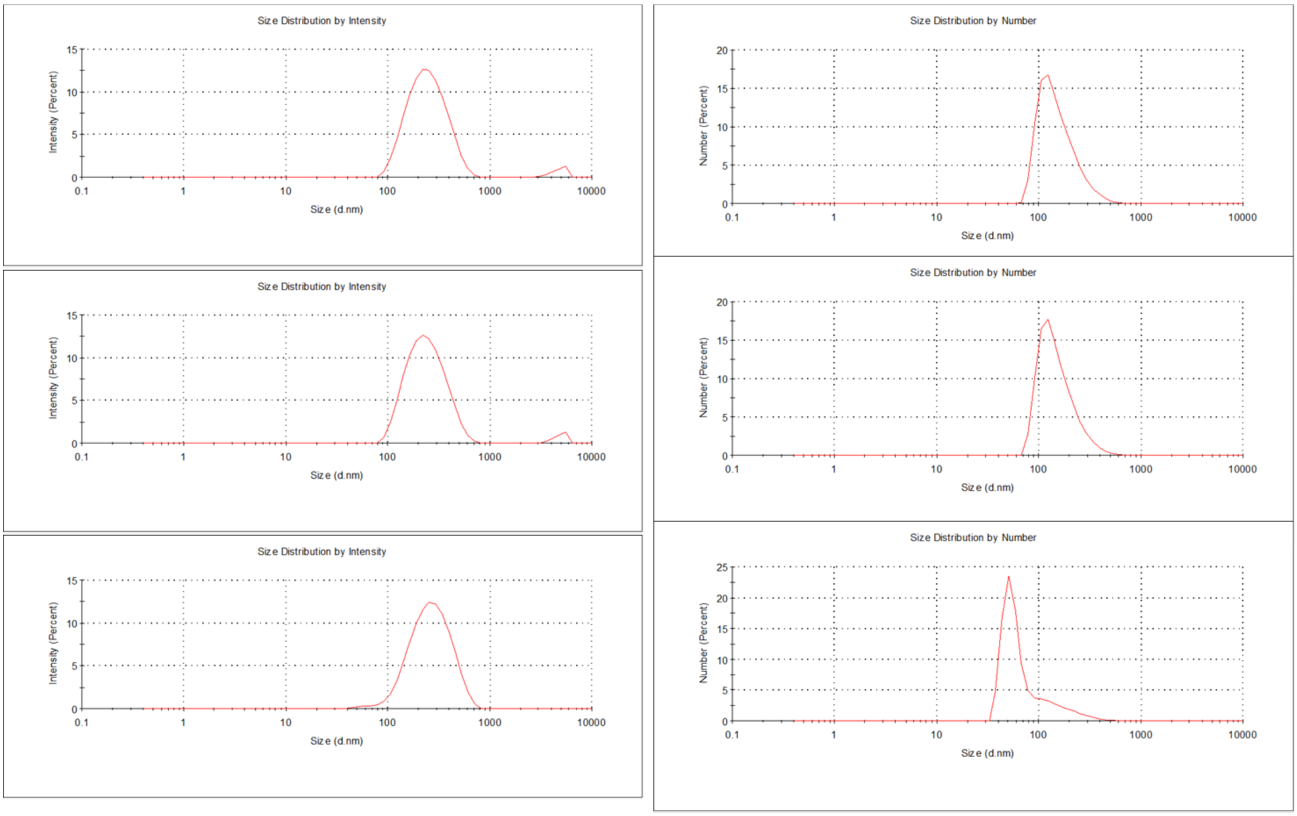

S88. Particle size distribution plots obtained from light scattering data of the nanosuspension of compound **18** 2 hours after nanoprecipitation. Plots of 3 separate measurements are shown by intensity (left) and number (right).

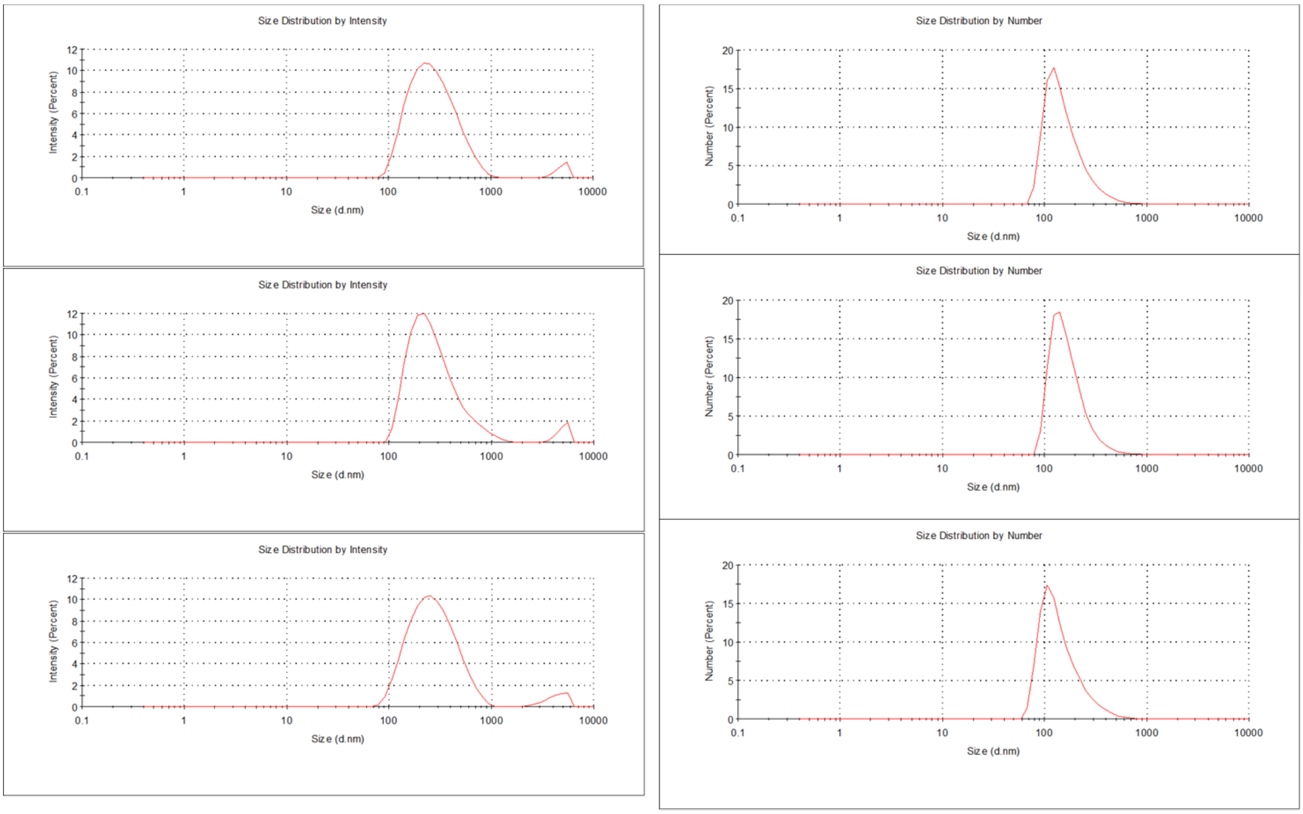

S89. Particle size distribution plots obtained from light scattering data of the nanosuspension of compound **18** 10 weeks after nanoprecipitation. Plots of 3 separate measurements are shown by intensity (left) and number (right).

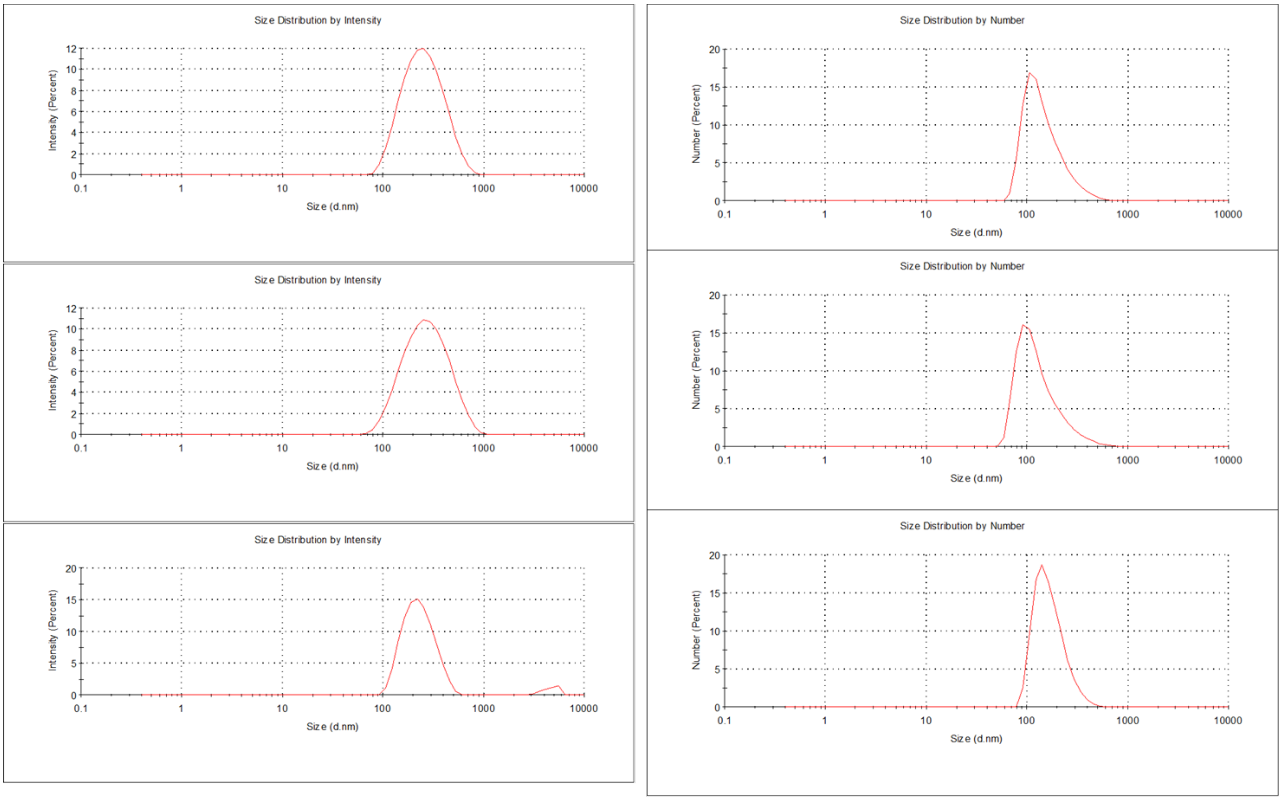

S90. Particle size distribution plots obtained from light scattering data of the nanosuspension of compound **19** 2 hours after nanoprecipitation. Plots of 3 separate measurements are shown by intensity (left) and number (right).

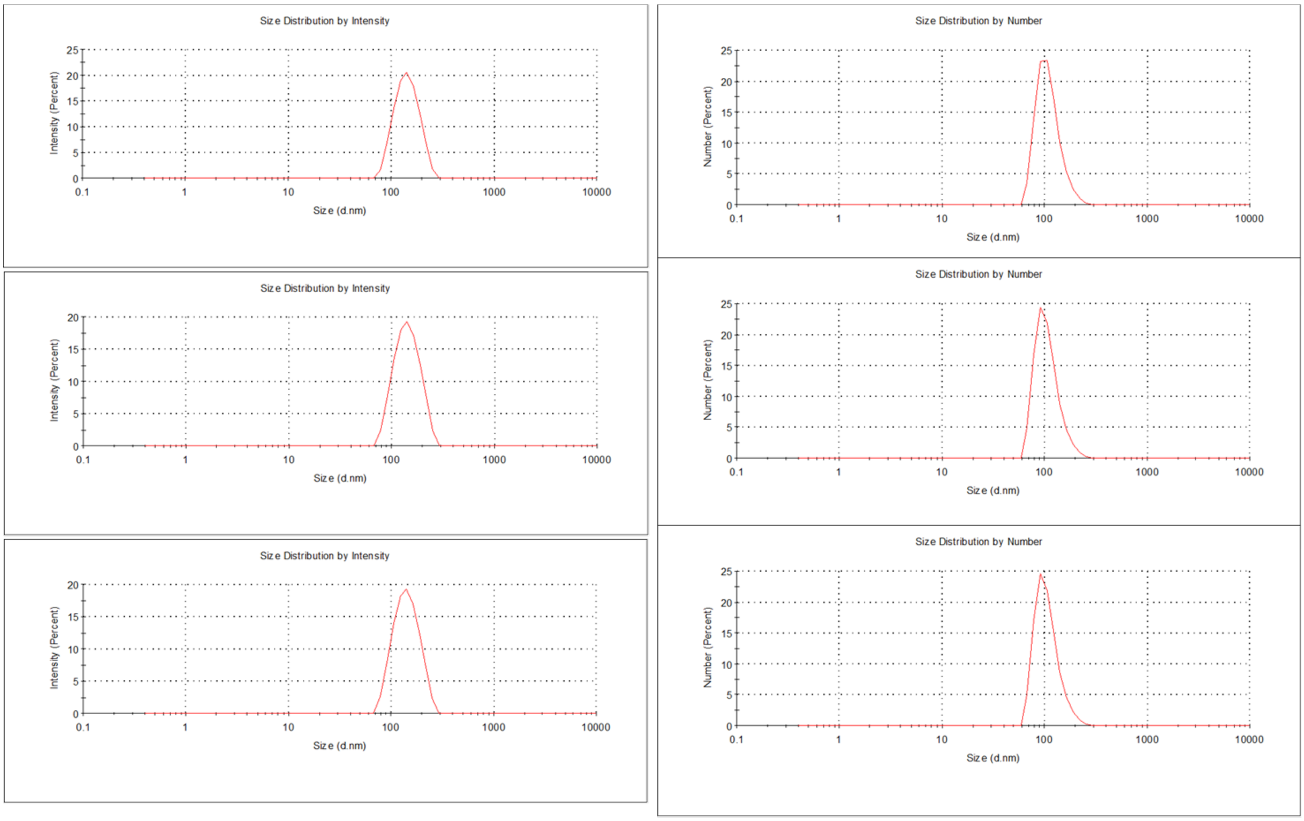

S91. Particle size distribution plots obtained from light scattering data of the nanosuspension of compound **19** 10 weeks after nanoprecipitation. Plots of 3 separate measurements are shown by intensity (left) and number (right).

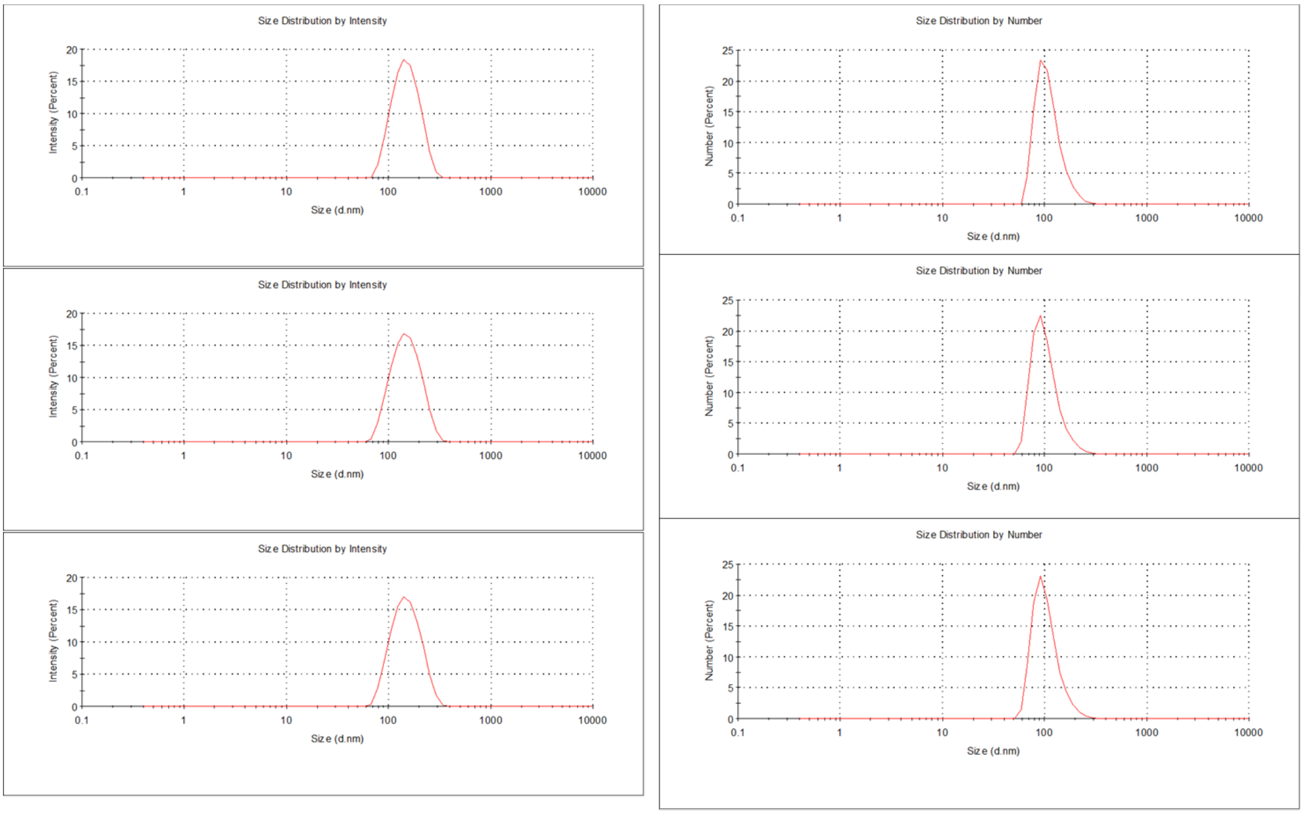

S92. Particle size distribution plots obtained from light scattering data of the nanosuspension of compound **20** 2 hours after nanoprecipitation. Plots of 3 separate measurements are shown by intensity (left) and number (right).

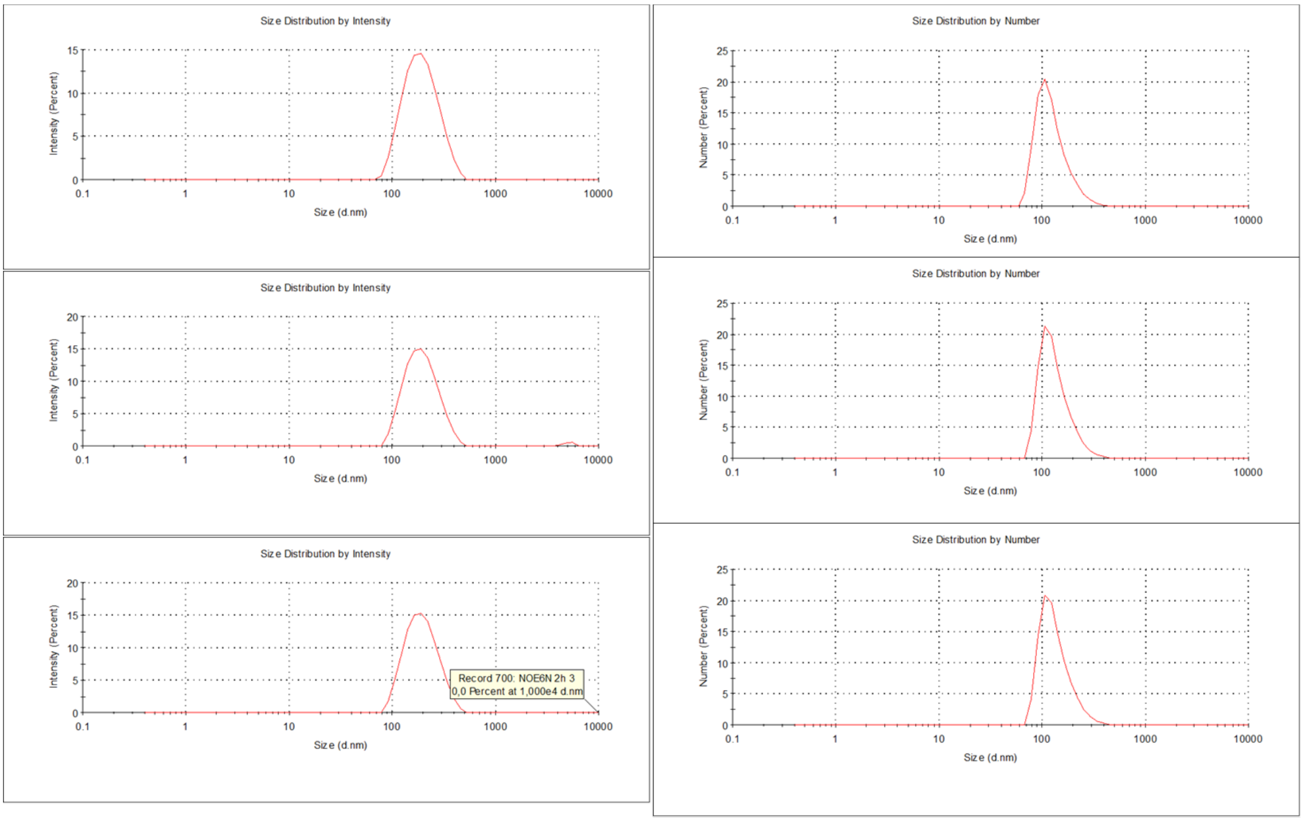

S93. Particle size distribution plots obtained from light scattering data of the nanosuspension of compound **20** 10 weeks after nanoprecipitation. Plots of 3 separate measurements are shown by intensity (left) and number (right).

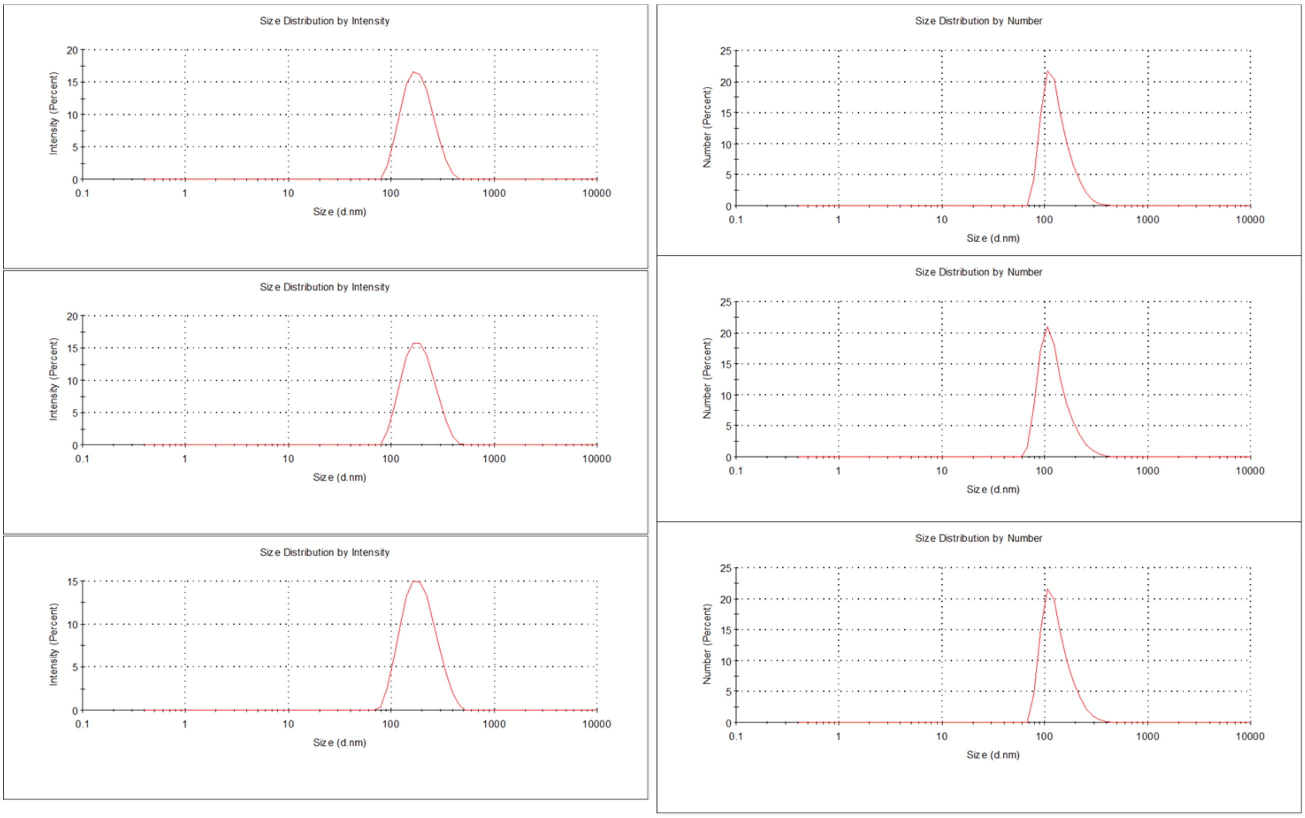

S94. Particle size distribution plots obtained from light scattering data of the nanosuspension of compound **21** 2 hours after nanoprecipitation. Plots of 3 separate measurements are shown by intensity (left) and number (right).

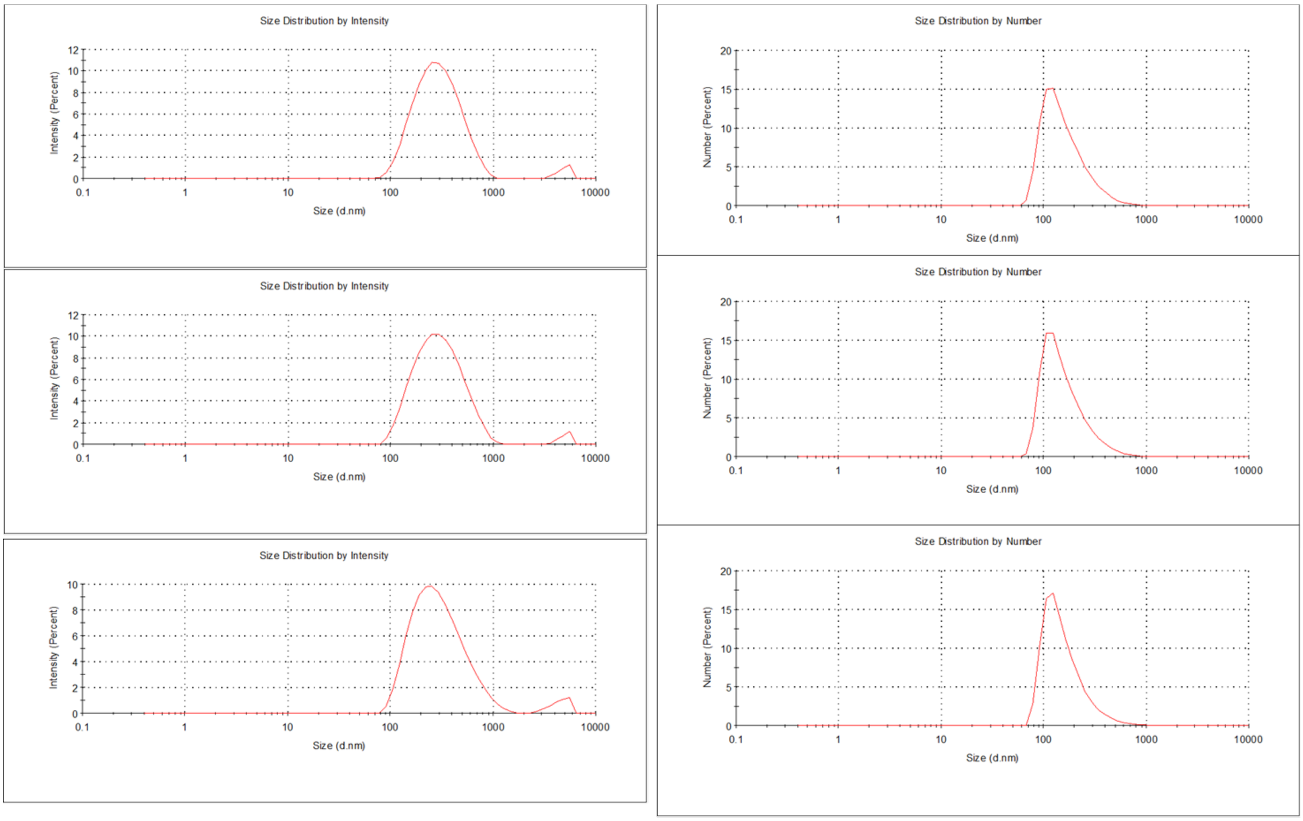

S95. Particle size distribution plots obtained from light scattering data of the nanosuspension of compound **21** 10 weeks after nanoprecipitation. Plots of 3 separate measurements are shown by intensity (left) and number (right).

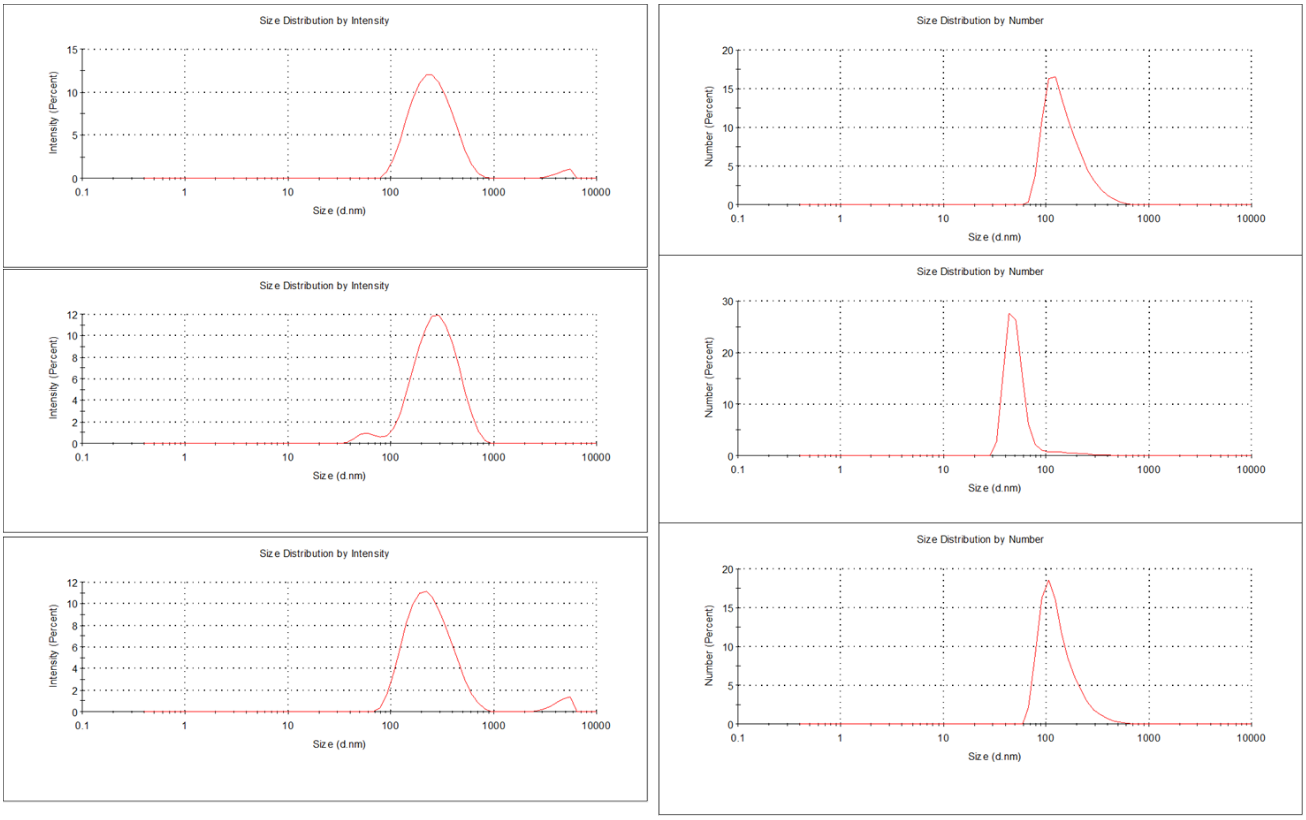

S96. Antiproliferative activity of compounds on SH-SY5Y (**A**) and MRC-5 (**B**) cells without tBHP. The cells were seeded in 96-well plate, incubated with the compounds at 0.5 µM concentration for 48 h. Cell viability was determined by MTT-assay. Data are reported as the means ± S.E.M.

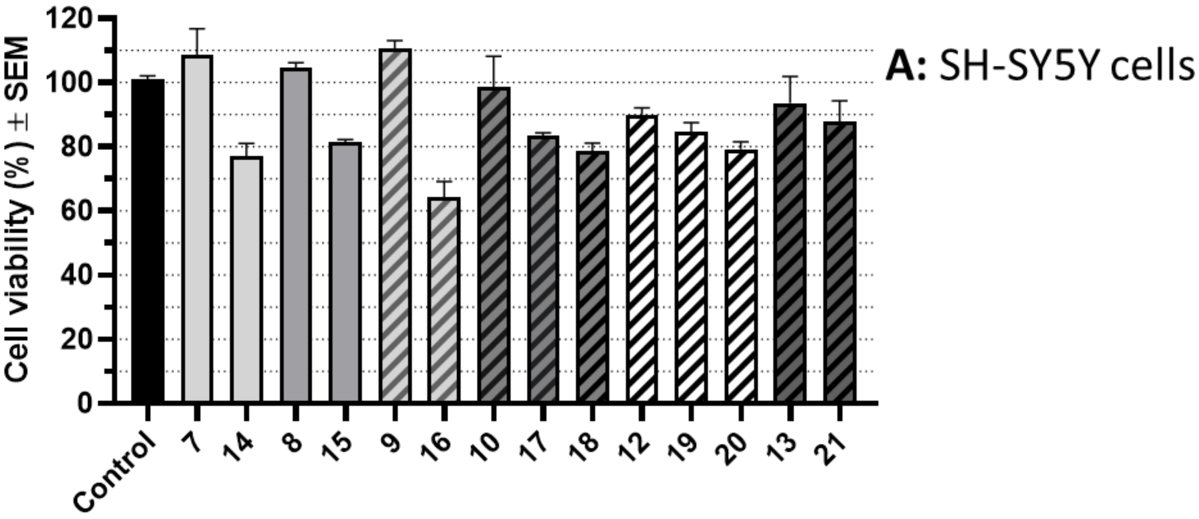

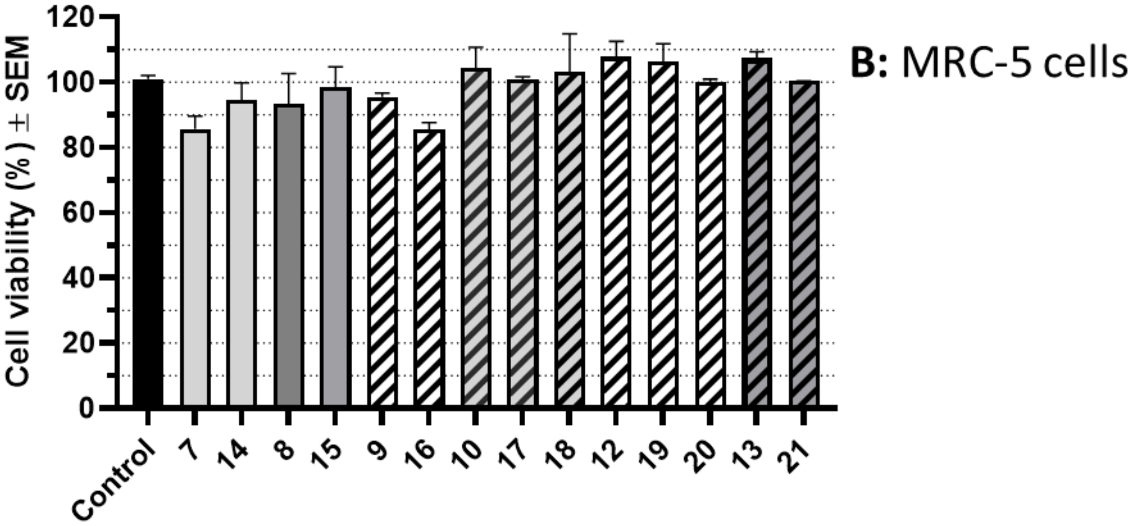

S97. Antiproliferative activity of vincristine as positive control on SH-SY5Y (**A**) and MRC-5 (**B**) cells without tBHP. The cells were seeded in 96-well plate, incubated with vincristine at 0.001 – 20.0 µM concentration for 48 h. Cell viability was determined by MTT-assay. Data are reported as the means ± S.E.M.

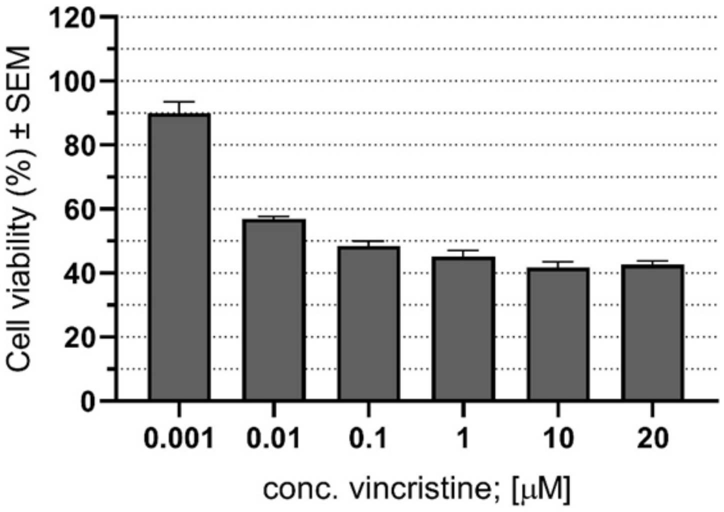

A: SH-SY5Y cells

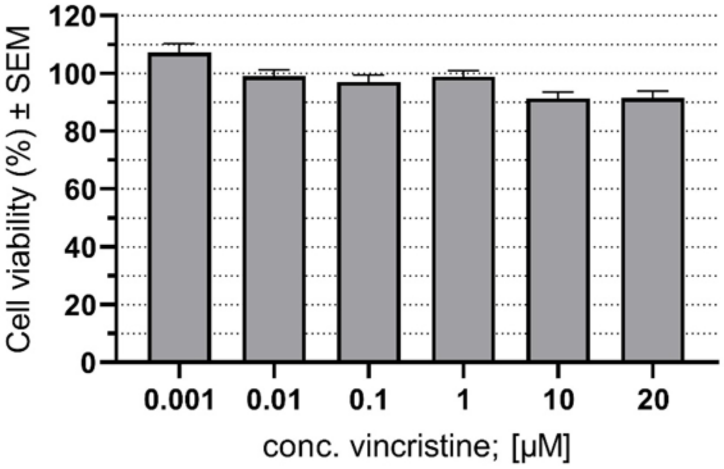

B: MRC-5 cells

S98. Dose-response curve of tBHP on SH-SY5Y cells after 4 h incubation. Cells were seeded in 96-well plate, incubated for 48 h, then tBHP was added in 1.95-1000 µM concentration. Cell viability was determined by MTT-assay. Experimental data points are shown as the mean ± S.E.M.

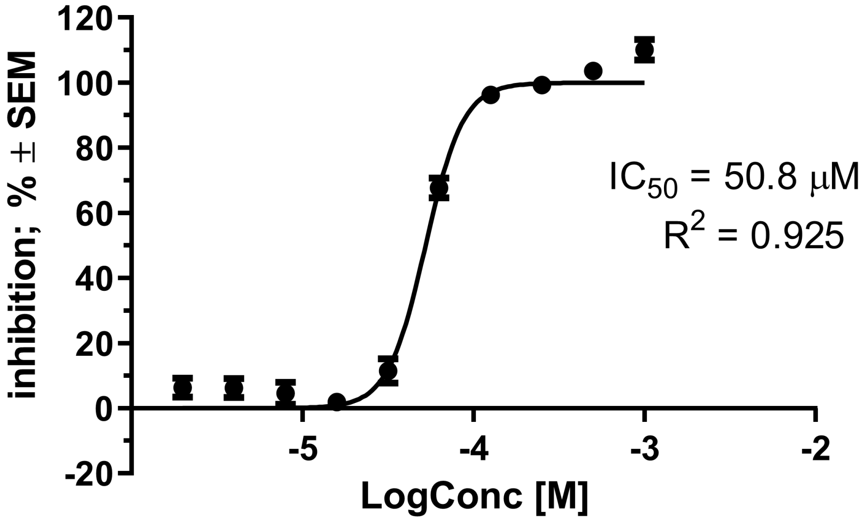

S99. Calculated IC_{50} values of tBHP on SH-SY5Y cells alone or pretreated with different concentrations (0.5–10 µM) of the compounds and incubated for 48 h, then tBHP was added in (1.95–1000 µM) for 4 h. Cell viability was determined by MTT-assay. Data are reported as the means ± S.E.M.

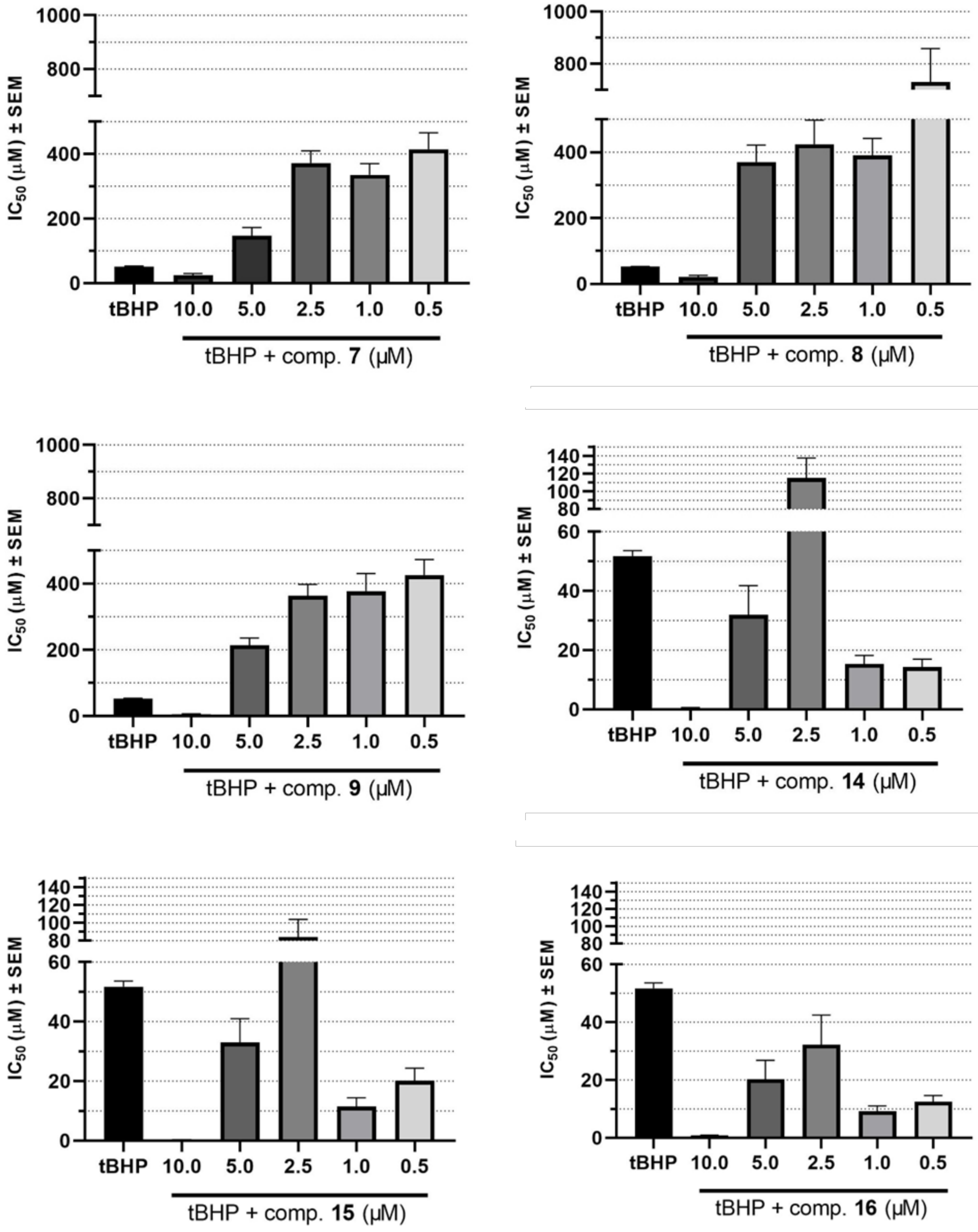

S100. Calculated IC_{50} values and selectivity indices (SI) of tBHP on SH-SY5Y and MRC-5 cells alone or pretreated with 0.5 µM of ecdysteroids (**7**–**13**) or their nanoconjugates (**14**–**21**).

**Compound**	**IC_{50} ± SEM (µM)**		**SI**
	**SH-SY5Y**	**MRC-5**	**IC_{50} $^{(MRC-5)}$ / IC_{50} $^{(SH-SY5Y)}$**
tBHP	50.8 ± 2.5	298.6 ± 75.3	5.90
**7**	517.6 ± 105.1	177.4 ± 37.2	0.34
**8**	760.3 ± 152.6	375.3 ± 82.4	0.49
**9**	427.4 ± 40.3	420.9 ± 96.5	0.98
**10**	71.5 ± 3.9	474.4 ± 137.1	6.64
**12**	65.8 ± 13.1	908.7 ± 112.8	13.80
**13**	56.8 ± 5.3	681.4 ± 123.1	12.00
**14**	25.9 ± 4.2	410.3 ± 84.1	15.85
**15**	30.2 ± 3.0	811.2 ± 76.3	26.83
**16**	19.0 ± 2.8	394.4 ± 71.8	20.73
**17**	11.6 ± 2.2	1357 ± 129.5	117.18
**18**	6.8 ± 0.8	1035 ± 214.8	153.11
**19**	14.4 ± 2.3	1181 ± 41.2	82.24
**20**	10.2 ± 1.1	394.3 ± 99.3	38.54
**21**	10.9 ± 1.5	459.0 ± 181.6	42.19

S101. Transmission electron microscopy (TEM) images of the nanoassemblies of compound **18**.

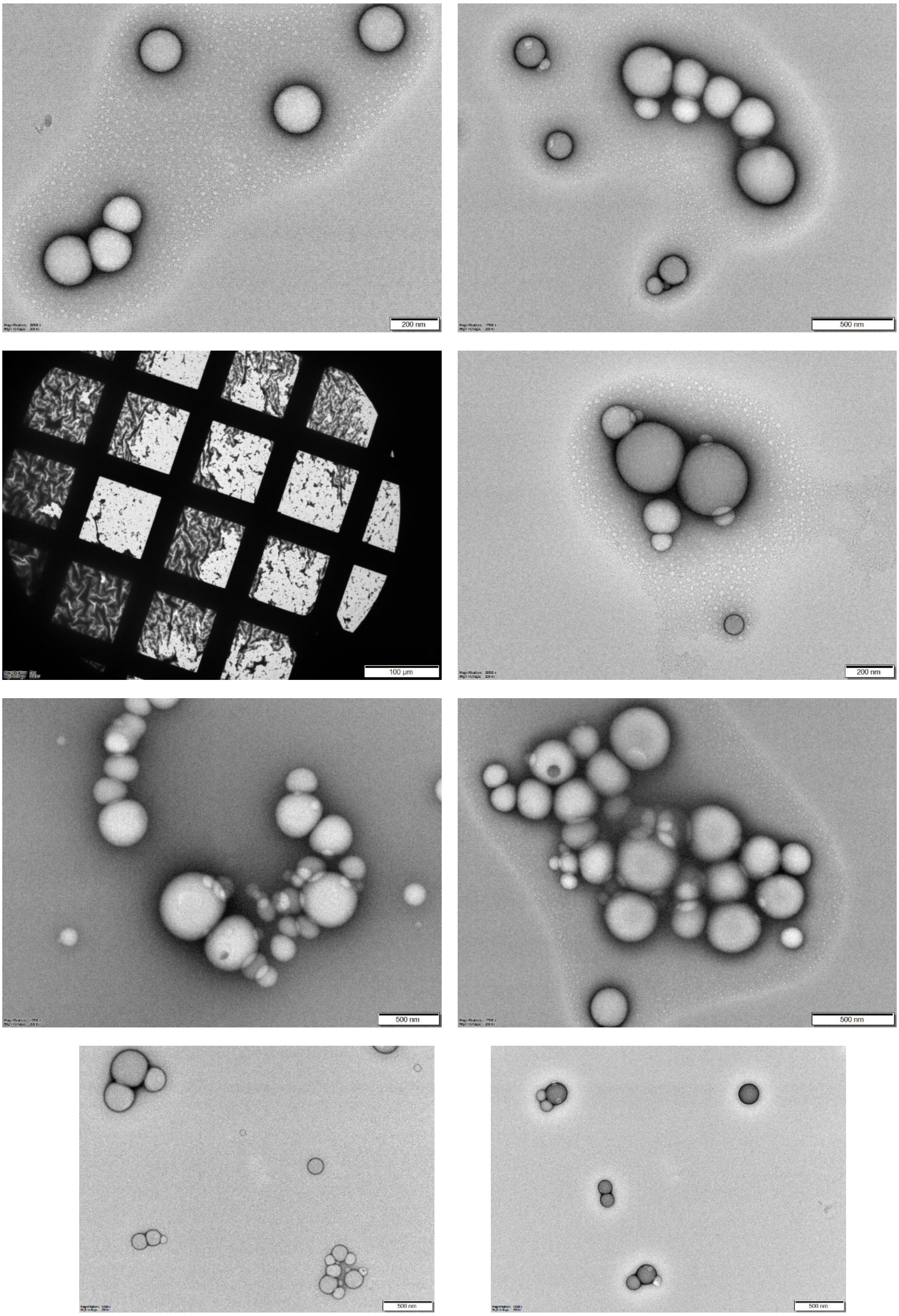

S102. RP-HPLC-PDA max plot chromatogram of ecdysteroid lead compound **9**. Mobile phase: $CH_3CN:H_2O$ – 68:32 v/v%. Flow rate: 1 ml/min. Column: Kinetex®, 5 µm, XB-C18, 100 Å, 250 mm x 4.6 mm column (Phenomenex Inc., Torrance, CA, USA).

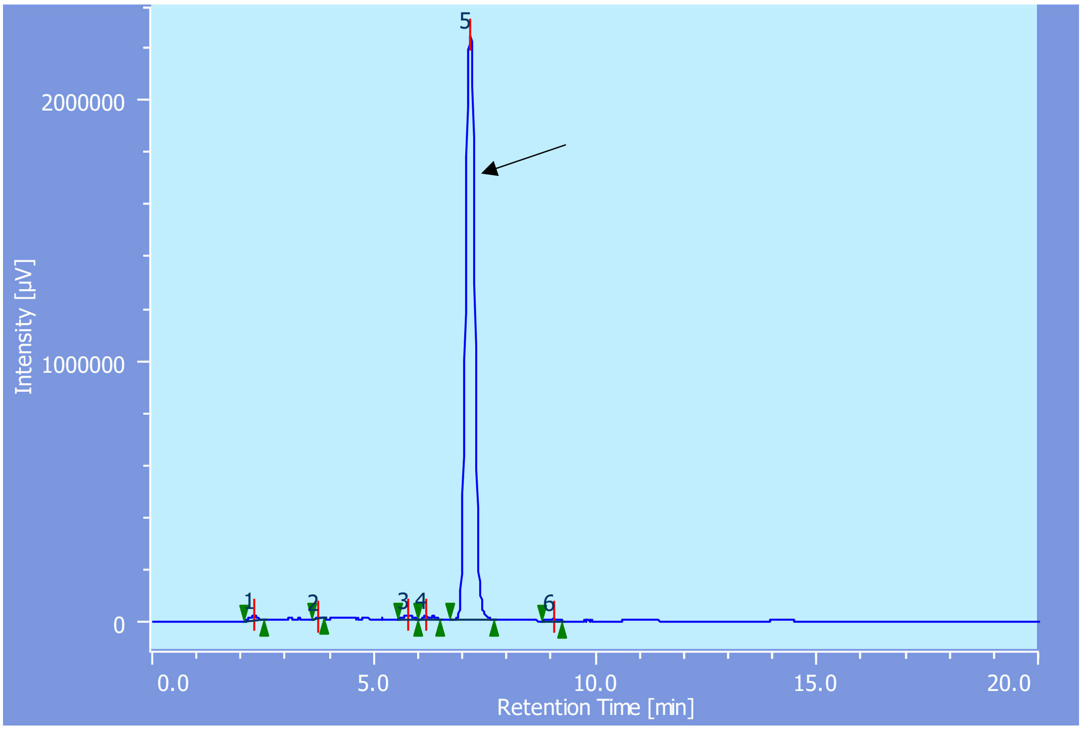

#	Peak Name	CH	tR	Area	Height	Area%	Height%	Quantity	NTP	Resolution	Symmetry Factor	Warning
1	Unknown	7	2.293	221981	19488	0.672	0.850	N/A	762	5.681	0.890	
2	Unknown	7	3.747	38427	5759	0.116	0.251	N/A	6883	7.363	0.940	
3	Unknown	7	5.773	210408	15560	0.637	0.678	N/A	3867	0.864	0.917	
4	Unknown	7	6.160	227590	14456	0.689	0.630	N/A	2198	2.228	2.111	
5	Unknown	7	7.173	32224043	2230414	97.597	97.241	N/A	5516	5.333	0.974	
6	Unknown	7	9.053	95028	8026	0.288	0.350	N/A	12763	N/A	0.842	

S103. SFC-PDA max plot chromatogram of ecdysteroid lead compound **16**. Mobile phase: CO_2:EtOH – 96:04 v/v%. Flow rate: 2 ml/min. Column: Phenomenex Luna® 5 µm, Silica (2), 100 Å, 250 mm x 4.6 mm HPLC column (Phenomenex Inc., Torrance, CA, USA).

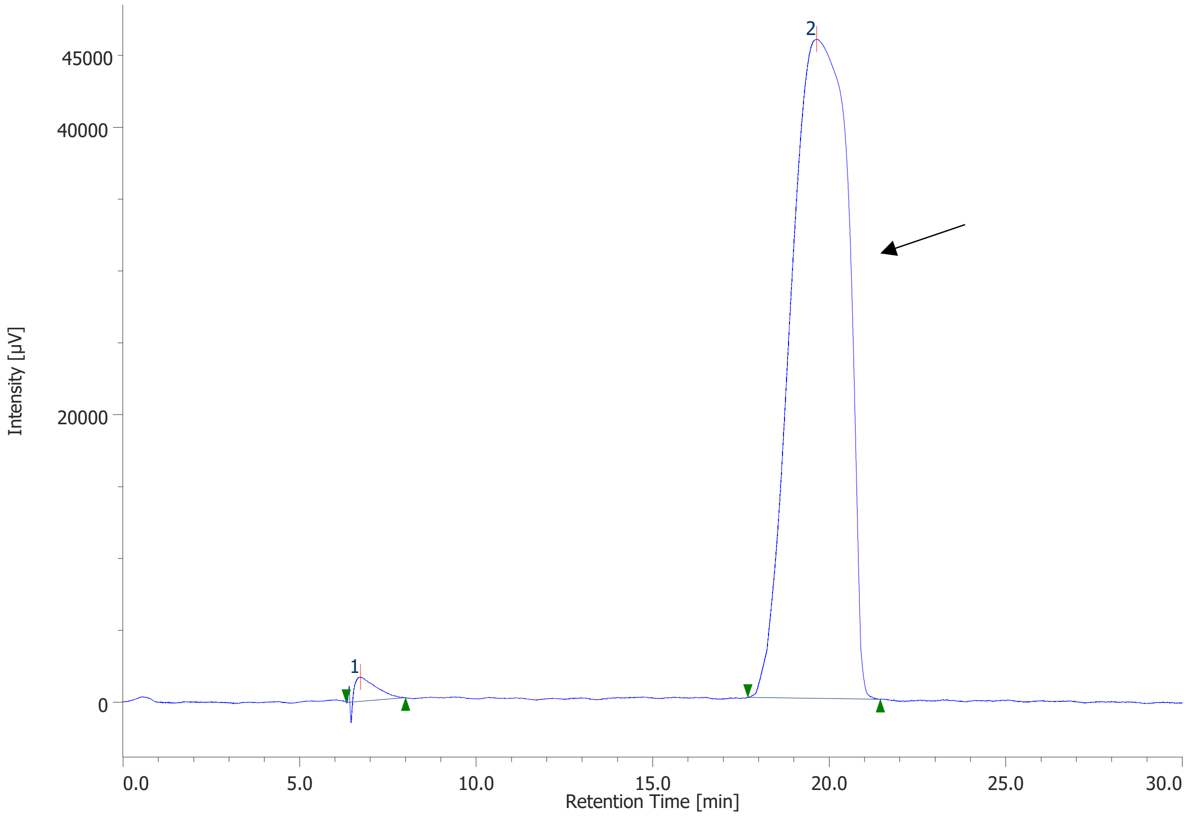

#	Peak Name	CH	tR	Area	Height	Area%	Height%	Quantity	NTP	Resolution	Symmetry Factor	Warning
1	Unknown	7	6.720	72156	1687	1.389	3.550	N/A	380	5.639	2.018	
2	Unknown	7	19.637	5123978	45829	98.611	96.450	N/A	597	N/A	0.932	
